# Supplementary material for: Small molecule branched-chain ketoacid dehydrogenase kinase (BDK) inhibitors with opposing effects on BDK protein levels
Source: Nat Commun. 2023 Aug 9;14:4812. doi: 10.1038/s41467-023-40536-y (PMC10412597; doi:10.1038/s41467-023-40536-y)
Supplement: Supplementary file 1 — Supplementary Information [file 41467_2023_40536_MOESM1_ESM.pdf]

Supplementary Information

Small molecule branched-chain ketoacid dehydrogenase kinase (BDK) inhibitors with opposing effects on BDK protein levels

Rachel J. Roth Flach, Eliza Bollinger, Allan R. Reyes, Brigitte Laforest, Bethany L. Kormos, Shenping Liu, Matthew R. Reese, Luis A. Martinez Alsina, Leanne Buzon, Yuan Zhang, Bruce Bechle, Amy Rosado, Parag V. Sahasrabudhe, John Knafels, Samit K. Bhattacharya, Kiyoyuki Omoto, John C. Stansfield, Liam D. Hurley, LouJin Song, Lina Luo, Susanne B. Breitkopf, Mara Monetti, Teresa Cunio, Brendan Tierney, Frank J. Geoly, Jake Delmore, C. Parker Siddall, Liang Xue, Ka N. Yip, Amit S. Kalgutkar, Russell A. Miller, Bei B. Zhang, Kevin J. Filipski

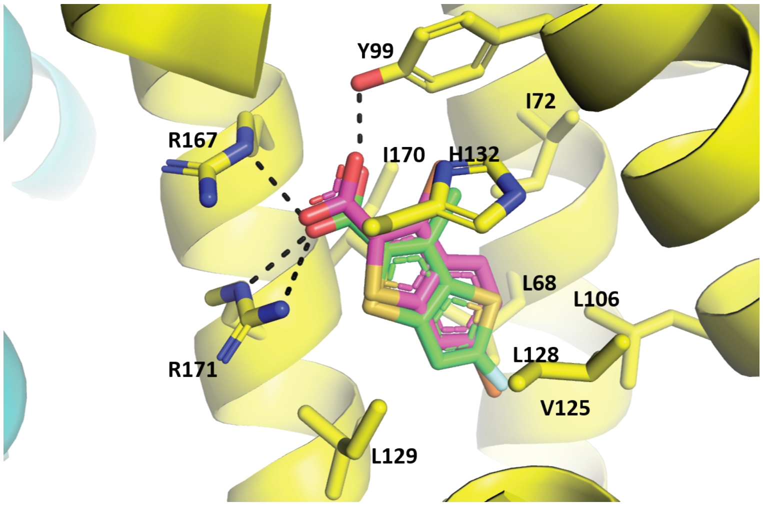

Supplementary Figure 1. Overlay of PF-07208254 and BT2 bound to hBDK from X-ray structures. PF-07208254 is shown in stick model with C atoms colored green. Superimposed is BT2 with C atoms colored in pink. BDK side chains that contact PF-07208254 are shown in sticks. Polar interactions are shown in dash.

Supplementary Figure 1
Related to Table 1

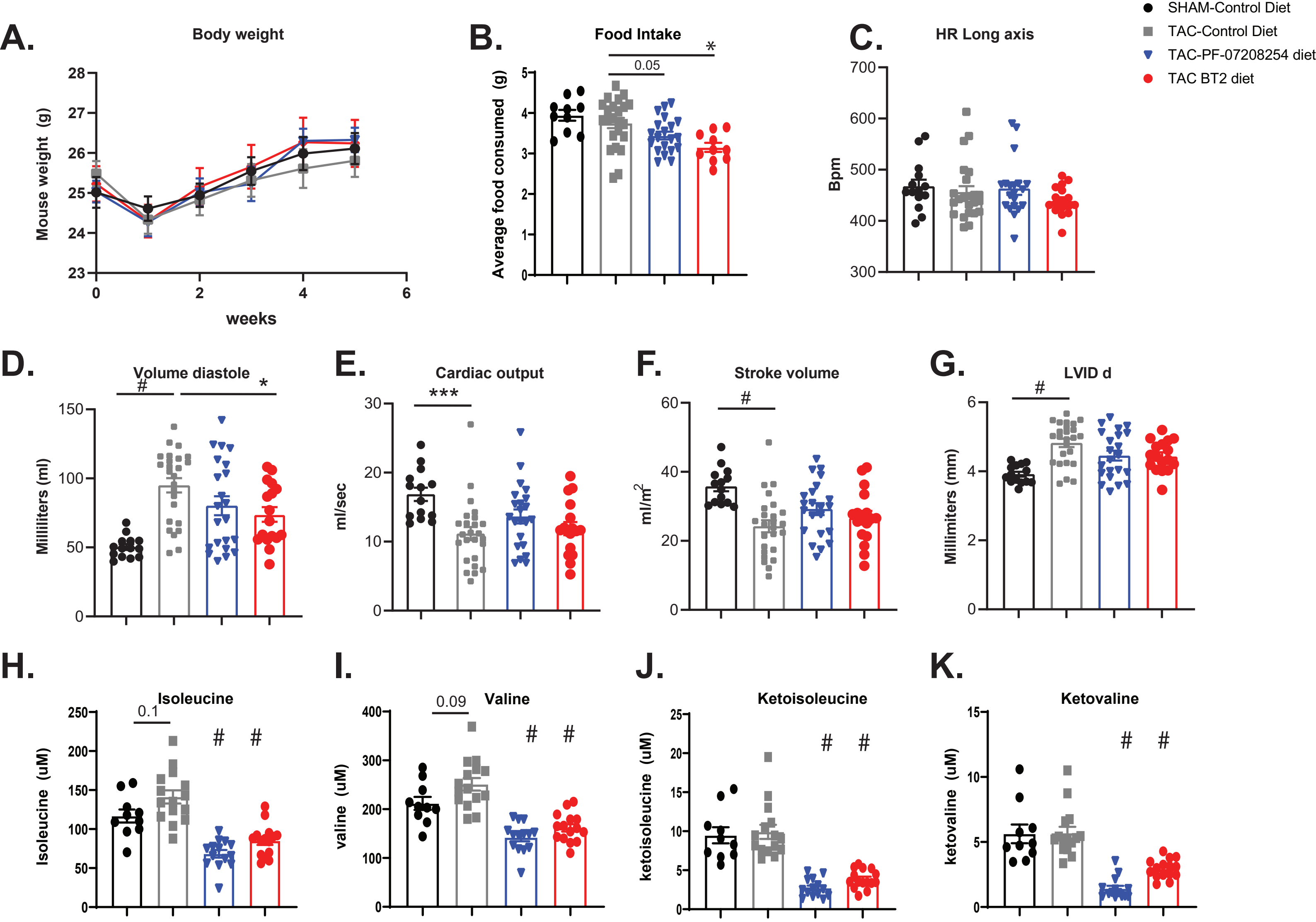

Supplementary Figure 2. PF-07208254 improves cardiac function in a TAC model and reduces BCAA and BCKA. Mice were subjected to TAC surgery as described in Figure **1A. A.** Body weights throughout the study (N=10-23 animals/group; statistics performed using a longitudinal mixed effects model with a random intercept for each mouse and an AR(1) covariance structure). **B.** Average daily food intake (N=10-23 animals/group; *p*=0.010). **C-G.** Echocardiography endpoints (N=14-26 animals/group). **C.** Heart rate (HR). **D.** Volume at diastole (#; *p*<0.0001, *; *p*=0.044). **E.** Cardiac output (**; *p*=0.001), **F.** Stroke volume (#; *p*<0.0001), **G.** Left ventricular internal diameter at diastole (LVID;d) (#; *p*<0.0001). **H-K.** Plasma BCAA and BCKA were measured at day 38 (N=10-14 animals/group). **H.** Isoleucine (#; *p*<0.0001), **I.** Valine (#; *p*<0.0001), **J.** Ketoisoleucine (#; *p*<0.0001), **K.** Ketovaline (#; *p*<0.0001). Statistics performed for B-K by one-way ANOVA with Tukey post-hoc test. Data represent the mean ± SEM. Source data are provided as a Source Data file.

Supplementary Figure 2
Related to Figure 1

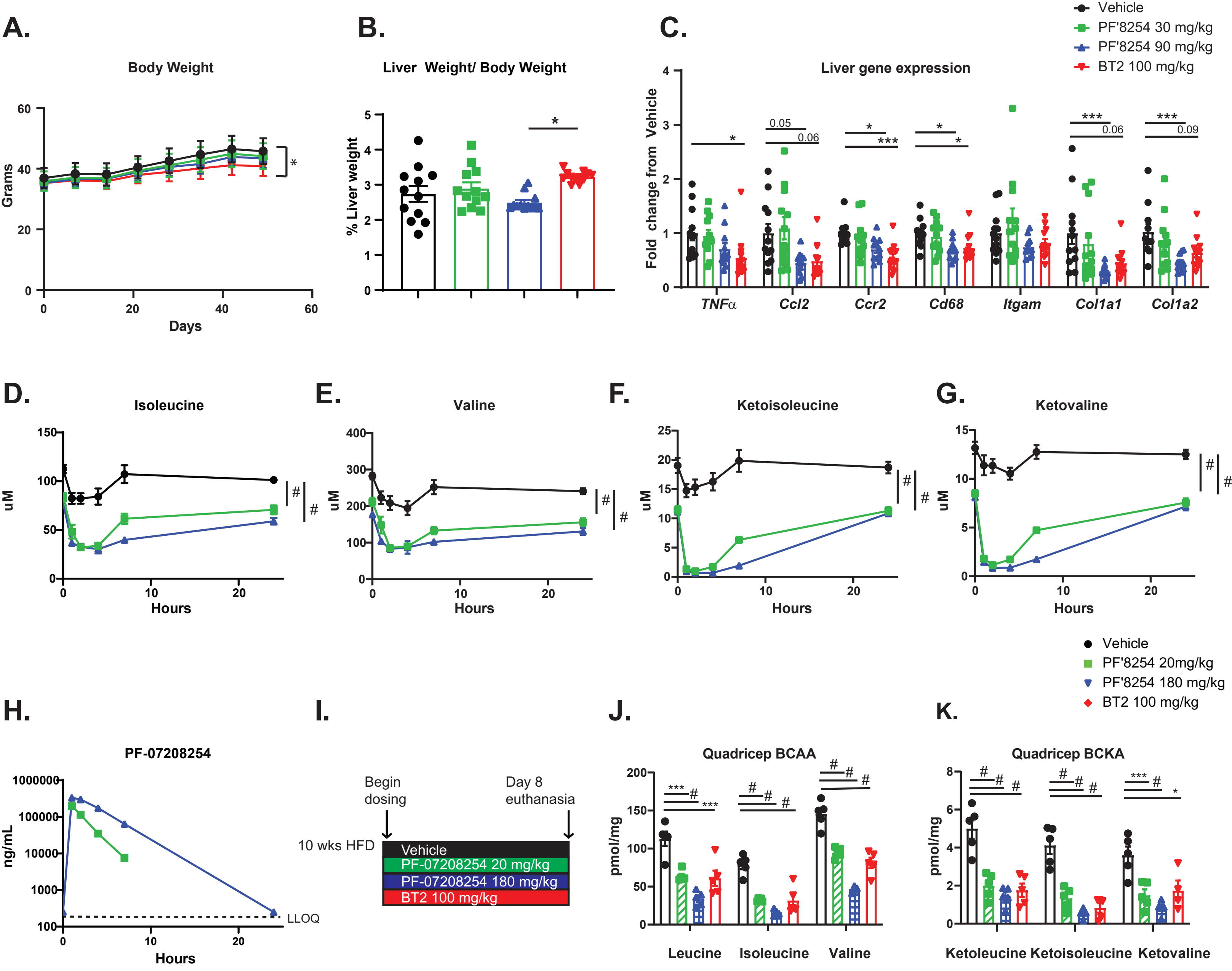

Supplementary Figure 3. PF-07208254 improves metabolic endpoints and lowers BCAA/BCKA in HFD-fed mice. Mice were fed HFD and treated with PF-07208254 or BT2 as in Figure **2A. A.** Body weights throughout the study (N=11-12; a longitudinal mixed effects model with a random intercept for each mouse and an AR(1) covariance structure was fit). **B.** Liver weight was measured as a percentage of body weight (N=12 animals/group; a pairwise Wilcoxon test was performed *; *p*=0.014). **C.** RNA was isolated from livers, and qRT-PCR was performed for several genes (N=12 animals/group; A one-way ANOVA with Tukey HSD test (*ccr2* *; *p*=0.043, ***; *p*=0.001) or a pairwise Wilcoxon test was performed (*tnfa* *; *p*=0.017, *ccl2, cd68* *; *p*=0.015, 0.043, *itgam, col1a1 ***; p*=0.004, *col1a2* ***; *p*=0.002). **D-H.** A PK/PD assessment was performed after 7 weeks of treatment in which mice are dosed with compound and timed bleeds are performed to measure BCAA, BCKA and drug levels (N=6-12 animals/group; a one-way ANOVA with Tukey HSD test was performed for D-G). **D.** Isoleucine (#; *p*<0.0001), **E.** Valine (#; *p*<0.0001), **F.** Ketoisoleucine (#; *p*<0.0001), **G.** Ketovaline (#; *p*<0.0001). **H.** PF-07208254 concentrations. **I-K.** HFD-fed mice were randomized into groups after 10 weeks on diet and treated for 8 days with PF-07208254 or BT2. Quadricep muscle was isolated one hour post final compound dose, and BCAA and BCKA were measured (N=4-5 animals/group; a one-way ANOVA with Tukey HSD test was performed). **I.** Study design. **J.** BCAA (***; *p*=0.001, #; *p*<0.0001). **K.** BCKA (*; *p*=0.019, ***; *p*=0.005, #; *p*<0.0001). Data represent the mean ± SEM. Source data are provided as a Source Data file.

Supplementary Figure 3; Related to Figure 2

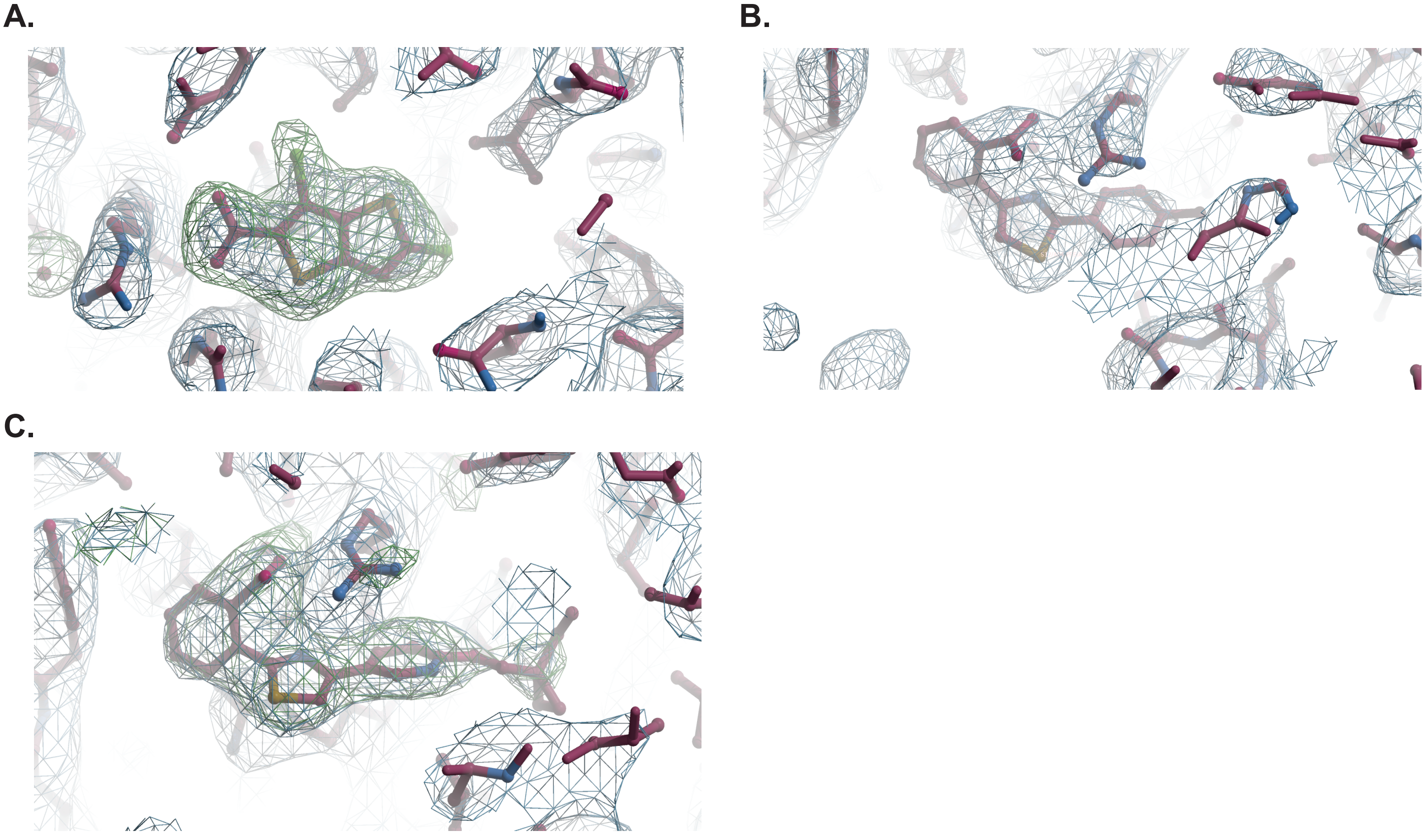

Supplementary Figure 4. OMIT maps for crystal structures. A. OMIT map for PF-07208254. To show model building quality, the final refined model was embedded in initial 2Fo-Fc (blue mesh) and Fo-Fc (green mesh) maps contoured at 1.2 σ and 3.0 σ, respectively, before the ligand was included in calculation. **B.** OMIT map for **S3**. To show model building quality, the final refined model was embedded in initial 2Fo-Fc (blue mesh) map contoured at 1.2 σ, before the ligand was included in calculation. **C.** OMIT map for PF-07247685. To show model building quality, the final refined model was embedded in initial 2Fo-Fc (blue mesh) and Fo-Fc (green mesh) maps contoured at 1.2 σ and 3.0 σ, respectively, before the ligand was included in calculation.

Supplementary Figure 4
Related to Figure 3

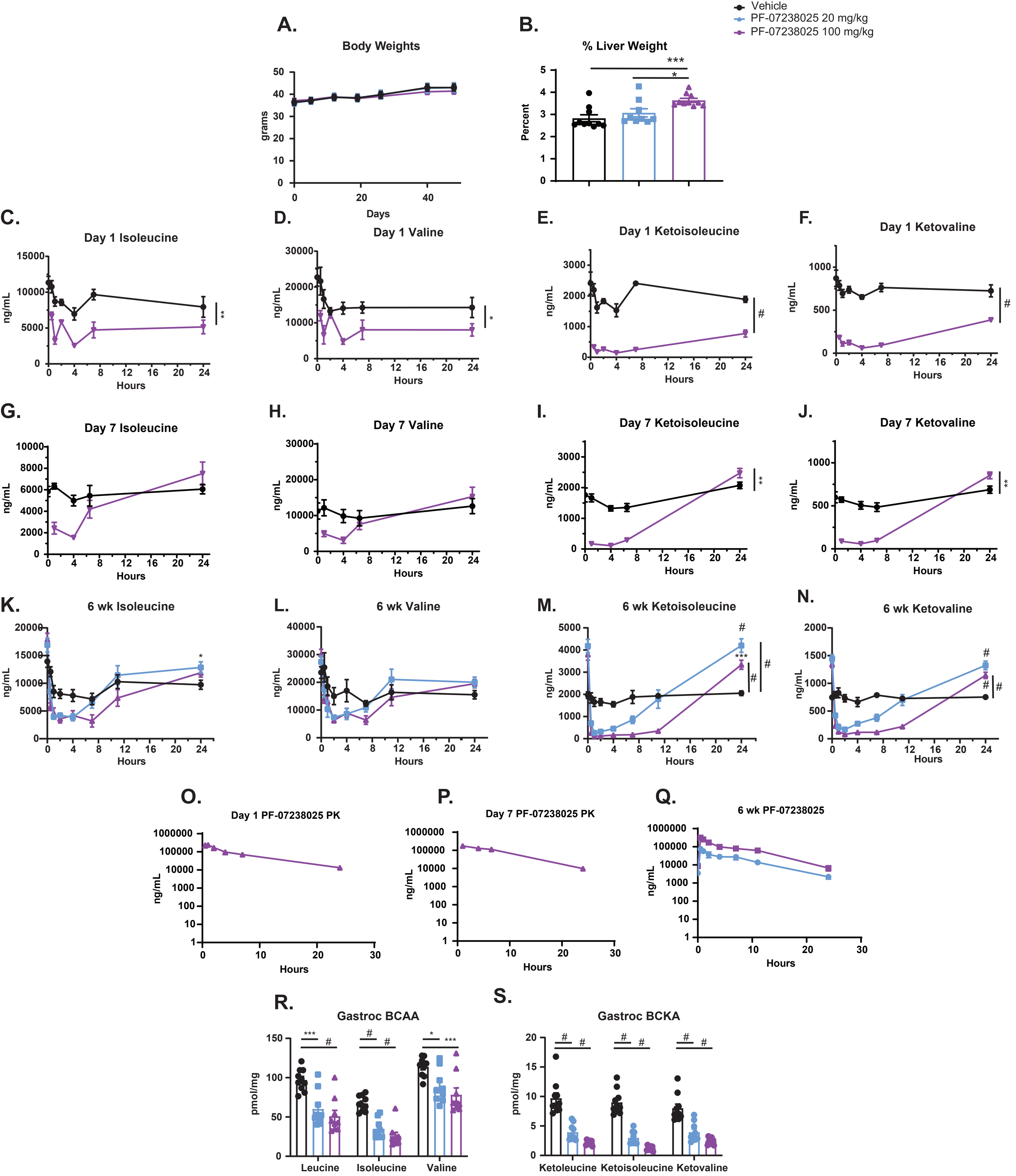

Supplementary Figure 5
Related to Figure 4

Supplementary Figure 5. Thiazole PF-07238025 does not improve metabolic endpoints after day 2, and BCAA/BCKA rebound occurs over time. Mice were fed HFD for 10 weeks, at which time animals were randomized into groups, and treated daily with vehicle or PF-07238025 for 8 weeks as in Figure **4D. A.** Body weights throughout the study duration (N=9-10 animals/group; a longitudinal mixed effects model with a random intercept for each mouse and an AR(1) covariance structure was fit). **B.** % liver weight at euthanasia (N=9-10 animals/group; a one-way ANOVA with Tukey HSD test was performed, *; p=0.03, ***; p=0.001). **C-Q.** PK/PD assessments were performed on day 1 (N=4 animals/group), after 7 days (N=4 animals/group), or 6 weeks of treatment (N=5-10 animals/group), in which mice were dosed with compound, and timed bleeds were performed to measure drug levels, BCAA, and BCKA levels, as in Figure **4K-N.** A one-way ANOVA with Tukey HSD test was performed on AUC and 24 hour time points for each analyte. **C.** Day 1 Isoleucine (**; p=0.0059), **D.** Day 1 Valine (*; p=0.027), **E.** Day 1 Ketoisoleucine (#; p=2.16x10^{-6}), **F.** Day 1 Ketovaline (#; p=0.0001), **G.** Day 7 Isoleucine, **H.** Day 7 Valine, **I.** Day 7 Ketoisoleucine (**; p=0.00055), **J.** Day 7 Ketovaline (***; p=0.0046), **K.** Week 6 Isoleucine (*; p=0.048), **L.** Week 6 Valine, **M.** Week 6 Ketoisoleucine (#; p<0.0001, ***; p=0.001), **N.** Week 6 Ketovaline (#; p<0.0001). **O-Q.** Total PF-07238025 concentrations at **O.** Day 1, **P.** Day 7, and **Q.** Week 6. **R-S.** Gastrocnemius muscle was isolated 1 hour post final compound dose, and **R.** BCAA (isoleucine***; p=0.001, #; p<0.0001, valine***; p=0.002, *; p=0.028) and **S.** BCKA were measured (#; p<0.0001). Data represent the mean ± SEM. Source data are provided as a Source Data file

Supplementary Figure 5
Related to Figure 4

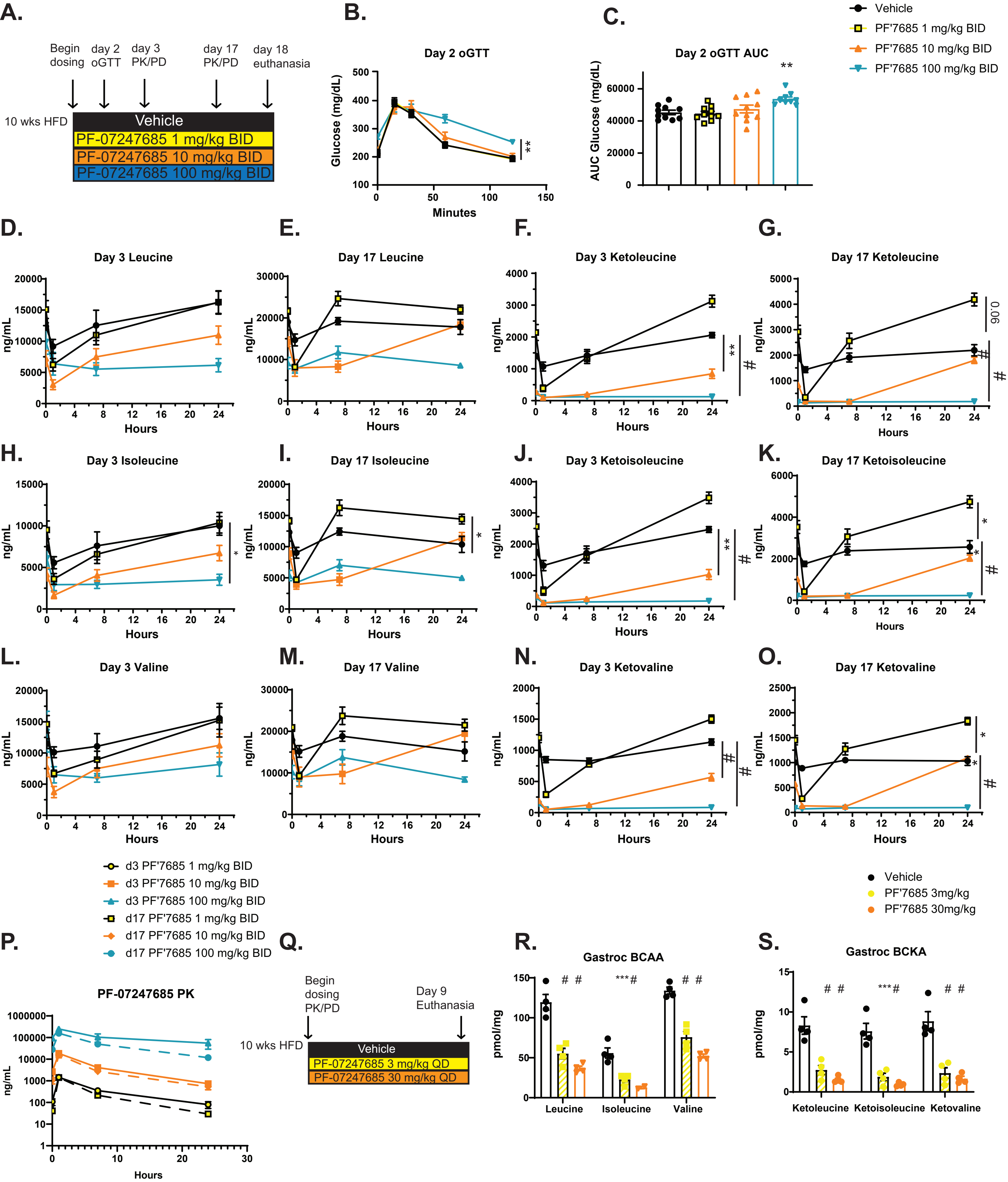

Supplementary Figure 6
Related to Figure 4

Supplementary Figure 6. Thiazole PF-07247685 does not improve glycemia but inhibits BDK and acutely lowers BCKA in plasma and tissues. Mice were fed HFD for 10 weeks, at which time animals were randomized into groups and treated BID with vehicle or PF-07247685 for 18 days. **A.** Study design. **B-C.** An oral glucose tolerance test (oGTT) was performed on day 2 (N=9-10 animals/group; one-way ANOVA with Tukey HSD test was performed on AUC, **; *p*=0.008). **B.** oGTT, **C.** oGTT AUC. **D-P.** PK/PD assessments were performed on days 3 and 17, in which mice were dosed with compound, and timed bleeds were performed to measure drug levels and BCKA levels (N=5 animals/group; one-way ANOVA with Tukey HSD test was performed on AUC in D-O). **D.** Day 3 Leucine, **E.** Day 17 Leucine, **F.** Day 3 Ketoleucine (**; *p*=0.006, #; *p*<0.0001), **G.** Day 17 Ketoleucine (#; *p*<0.0001), **H.** Day 3 Isoleucine (*; *p*=0.02), **I.** Day 17 Isoleucine (*; *p*=0.028), **J.** Day 3 Ketoisoleucine (**; *p*=0.001, #; *p*<0.0001), **K.** Day 17 Ketoisoleucine (*; *p*=0.012, 0.019, #; *p*<0.0001), **L.** Day 3 Valine, **M.** Day 17 Valine, **N.** Day 3 Ketovaline (#; *p*=0.0000), **O.** Day 17 Ketovaline (*; *p*=0.01, 0.023, #; *p*<0.0001), **P.** Total PF-07247685 levels on days 3 and 17. **Q-S.** Mice were fed HFD for 10 weeks, at which time animals were randomized into groups and treated QD with vehicle or PF-07247685 for 9 days. **Q.** Study design. **R-S.** Muscle was harvested one hour post final compound dose, and **R.** BCAA (#; *p*<0.0001, ***; *p*=0.001) and **S.** BCKA (#; *p*<0.0001) were assessed by mass spectrometry (N=2-4 animals/group; one-way ANOVA with Tukey HSD test was performed for statistics). Data represent the mean ± SEM. Source data are provided as a Source Data file.

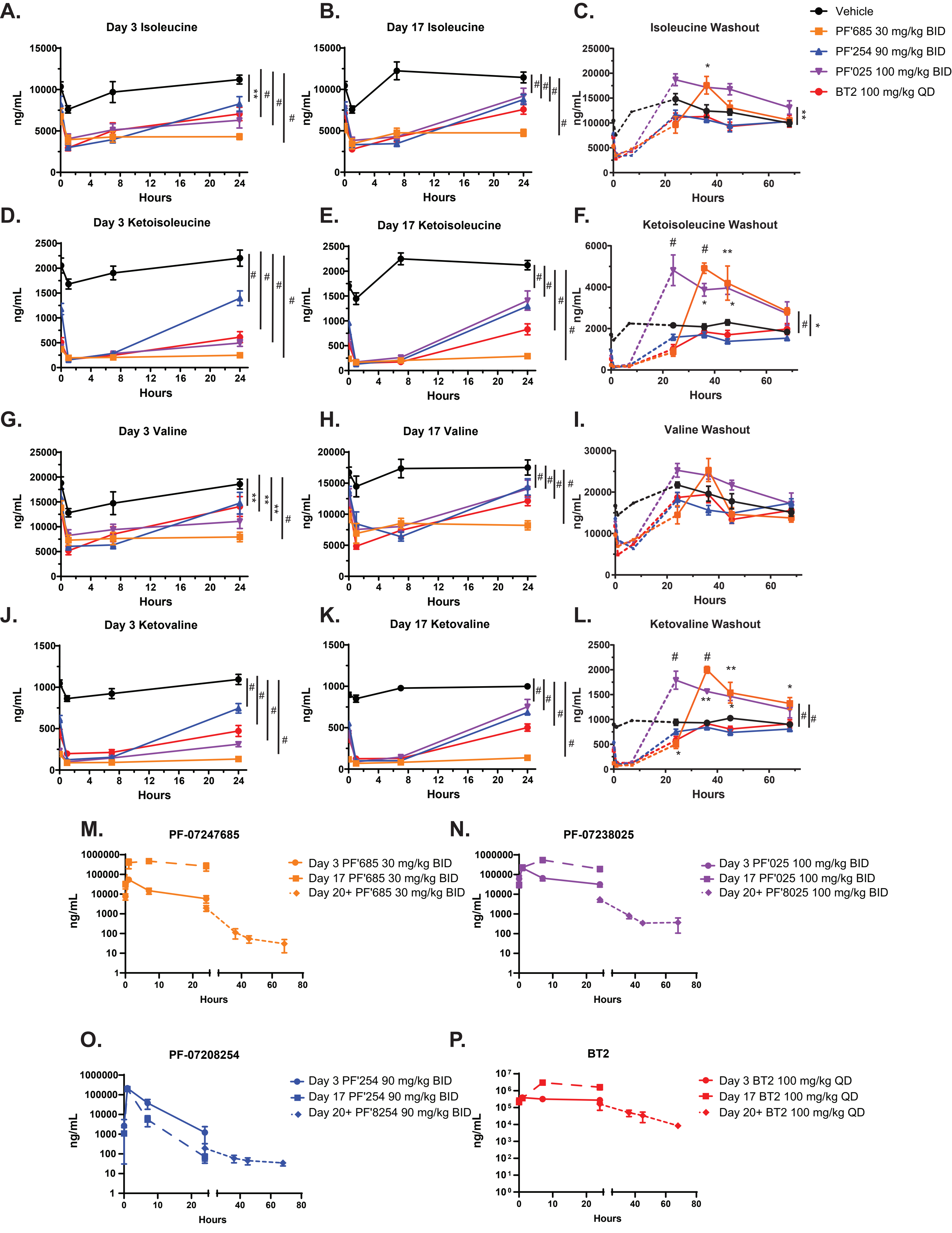

Supplementary Figure 7; Related to Figure 5

Supplementary Figure 7. Additional BCAA/BCKA and drug levels during study described in Figure 5. Mice were fed HFD for 10 weeks, at which time animals were randomized into groups, and treated BID or QD as indicated with vehicle, BT2, PF-07208254, PF-07247685, or PF-07238025 at maximal inhibitory doses. Mice were bled immediately prior to dosing compound, 1 hour post compound dose, 4 hours post compound dose, 7 hours post compound dose (immediately prior to 2nd daily compound dose if BID dosed), and 24 hours after first compound dose on day 3 and day 17 (N=8-9 animals/group). Washout bleeds were performed at 24, 36, 45 and 68 hours post final compound dose on days 20-22 (N=5 animals/group; a one way ANOVA with Tukey post hoc test was used for statistics on A-L for AUC and 24, 36, 45, 68 h time points). **A.** Isoleucine was measured on day 3 (**; *p*=0.008, #; *p*<0.0001), **B.** day 17 (#; *p*<0.0001), and **C.** upon compound washout (*; *p*=0.03, **; *p*=0.001). **D.** Ketoisoleucine was measured on day 3 (#; *p*<0.0001), **E.** day 17 (#; *p*<0.0001), and **F.** upon compound washout (AUC #; *p*<0.0001, *; *p*= 0.010, per time point *p*<0.0001, *; *p*=0.010, 0.018, **; *p*=0.003). **G.** Valine was measured on day 3 (**; *p*=0.001, #; *p*<0.0001), **H.** day 17 (#; *p*<0.0001), and **I.** upon compound washout. **J.** Ketovaline was measured on day 3 (#; *p*<0.0001), **K.** day 17 (#; *p*<0.0001), and **L.** upon compound washout (AUC #; *p*<0.0001, per time point #; *p*<0.0001, **; p=0.001, p=0.002, *; p=0.022, 0.007, 0.03). Dashed lines in panels C,F,I & L represent the 0-7 hour time points from panels B, E, H & K for visualization purposes. Data represent the mean ± SEM. **M-P.** Drug levels throughout study. **M.** PF-07247685, **N.** PF-07238025, **O.** PF-07208254, **P.** BT2. Solid lines represent day 3, dashed lines day 17, dotted lines are washout days 20-21. Data represent the mean ± SD. Source data are provided as a Source Data file.

Supplementary Figure 7; Related to Figure 5

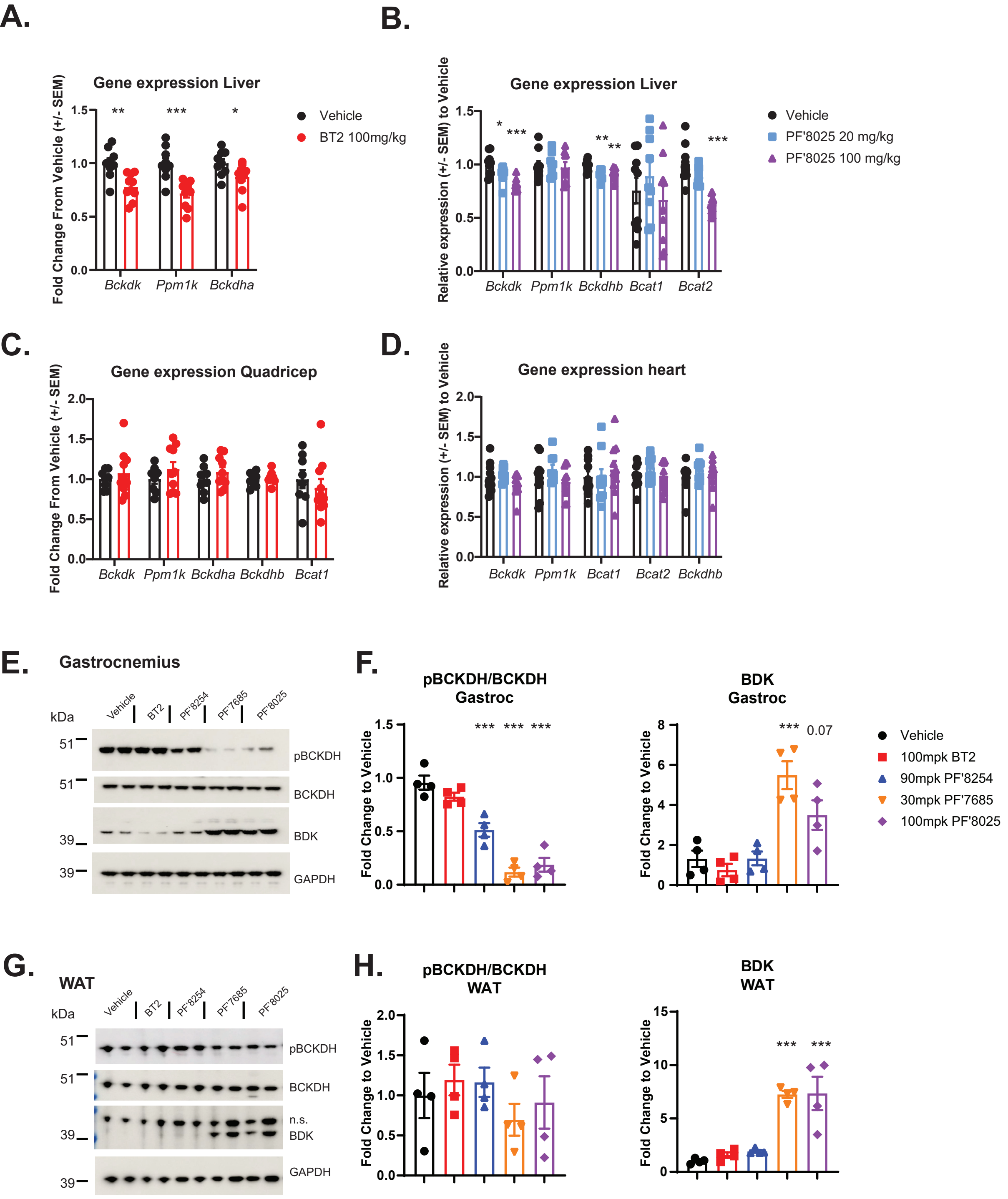

Supplementary Figure 8. BDK mRNA levels not driving BDK protein changes with BT2 or PF-07238025 and BDK protein is upregulated in multiple tissues with thiazoles. A-D. One hour post final compound dose, tissue was harvested. mRNA was isolated, and qPCR was performed from HFD-fed animals that had been treated with BT2 or PF-07238025 (N=8-10 animals/group; a Welch two-sample t-test was performed for statistics). **A.** Liver after BT2 treatment (**; *p*=0.001, ***; *p*=0.00047, *; *p*=0.03). **B.** Liver after PF-07238025 treatment (*; *p*=0.041, **; *p*=0.005, 0.008 ***; *p*<0.0001). **C.** Quadricep after BT2 treatment. **D.** Heart after PF-07238025 treatment. **E-H.** One hour post final compound dose, tissue was harvested and lysed, and Western blots were performed from HFD-fed animals that had been treated with BT2, PF-07208254, PF-07238025 or PF-07247685 (N=4 animals/group; a one-way ANOVA with Tukey HSD test was performed for statistics). **E.** Gastrocnemius tissue, **F.** Densitometry of **E.** (***; p<0.0001) **G.** White adipose tissue (WAT). **H.** Densitometry of **G.** (***; p<0.0001) Data represent the mean ± SEM. *; *p*<0.05; **; *p*<0.01, ***; *p*<0.005. Source data are provided as a Source Data file.

Supplementary Figure 8; Related to Figure 6

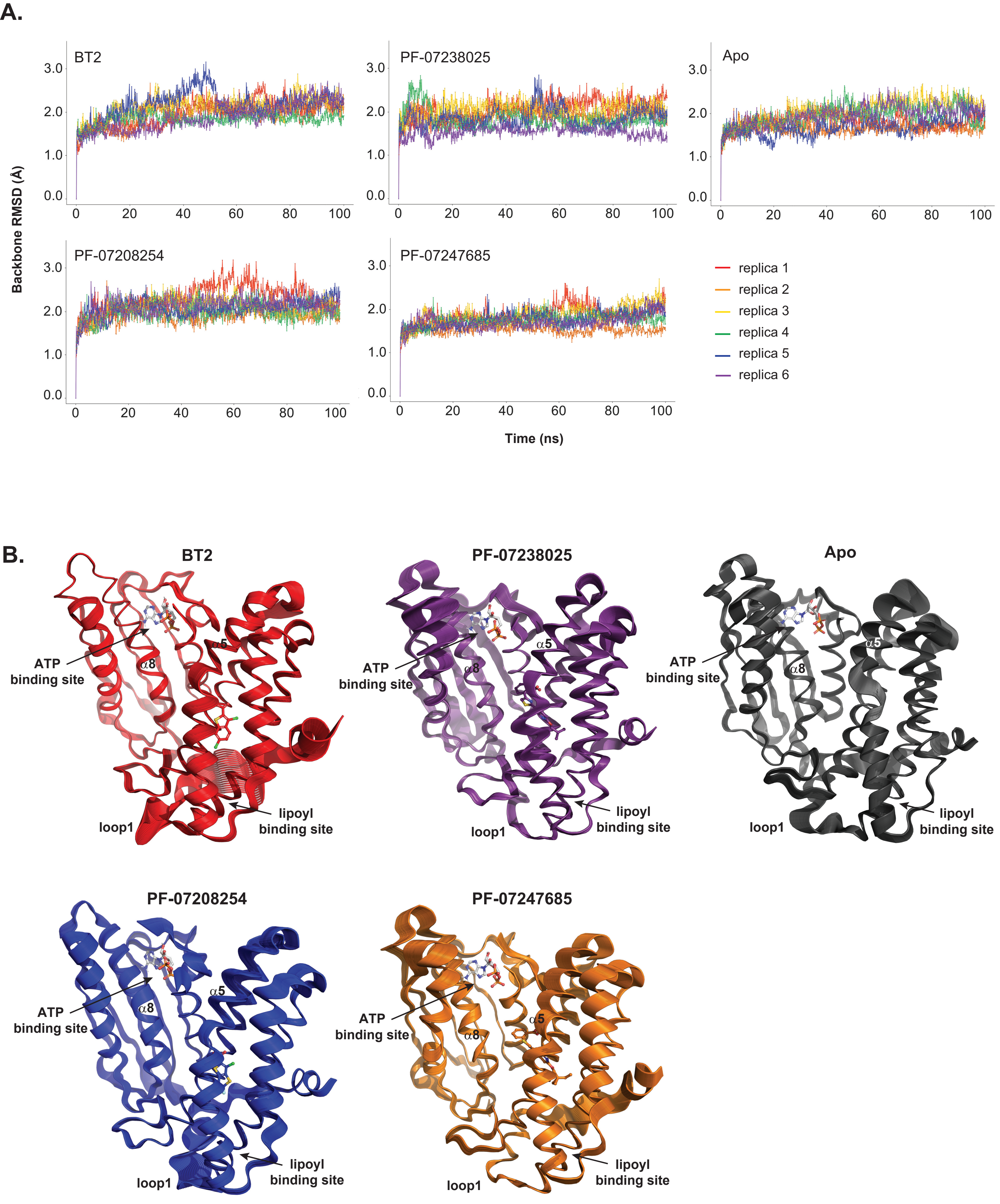

Supplementary Figure 9

Supplementary Figure 9. BDK MD trajectories are stable over the course of the simulations; Loop 1 shows dynamic differences. A. Root-mean-square deviation (RMSD) of BDK backbone atoms with respect to the starting structure over six replicas of 100 ns when bound to BT2, PF-07208254, PF-07238025, PF-07247685 and apo. **B.** Visualizations of the first principal component of BDK bound to destabilizers BT2 (red) and PF-07208254 (blue), stabilizers PF-07238025 (purple) and PF-07247685 (orange), and apo (black).

Supplementary Figure 9

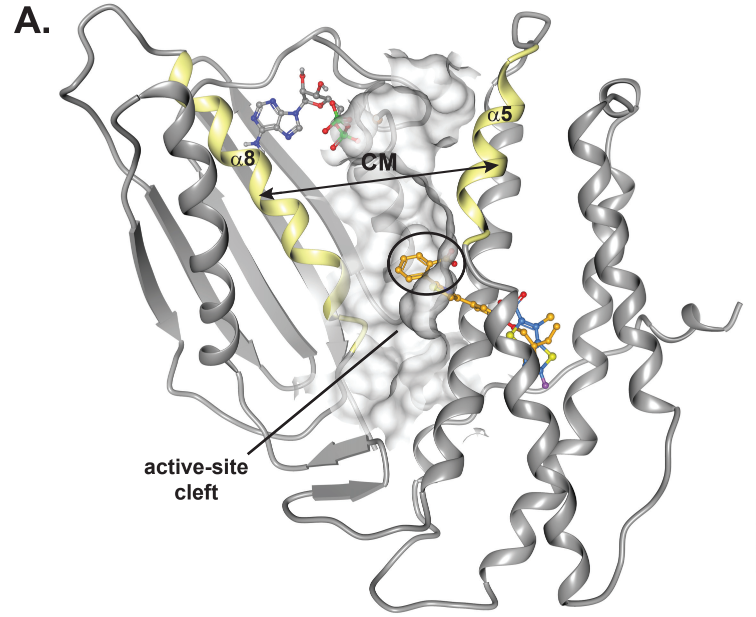

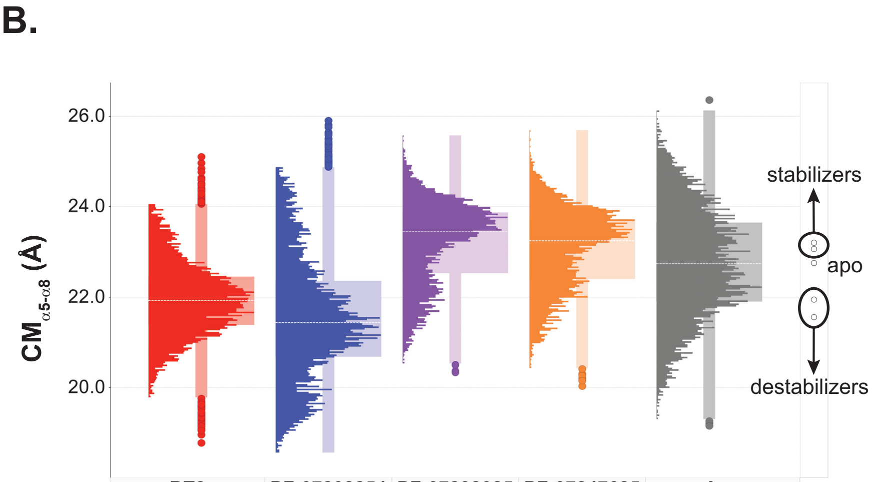

	BT2	PF-07208254	PF-07238025	PF-07247685	Apo
Count	6000	6000	6000	6000	6000
Median	21.9	21.4	23.4	23.2	22.7
Outliers	88	72	4	6	4

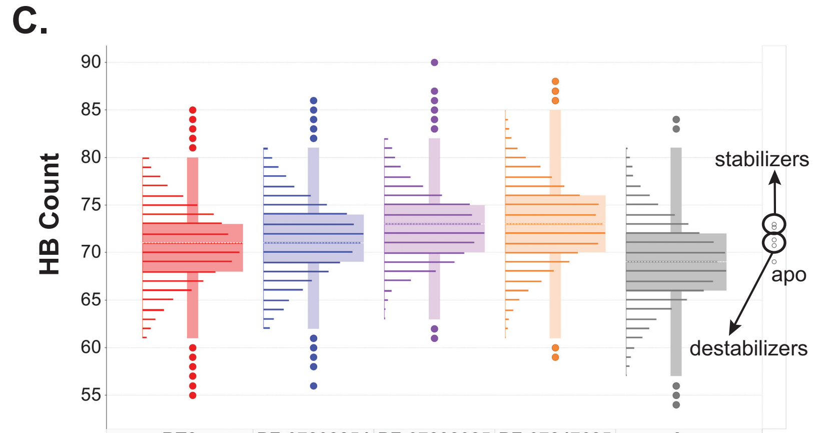

	BT2	PF-07208254	PF-07238025	PF-07247685	Apo
Count	6000	6000	6000	6000	6000
Median	71	71	73	73	69
Outliers	83	71	28	14	12

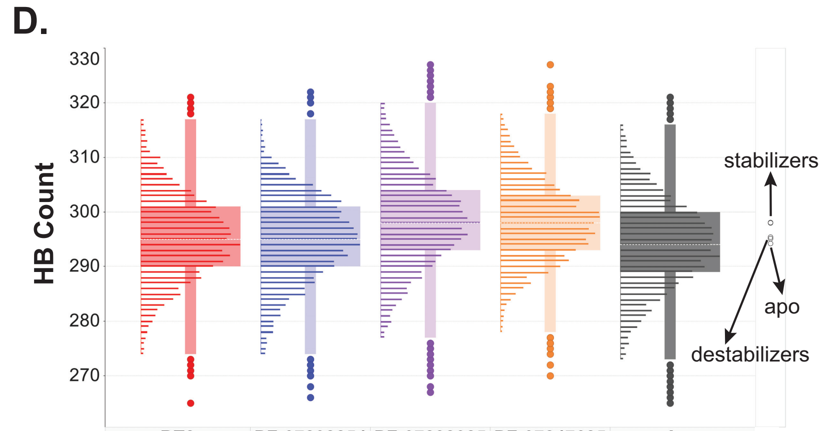

	BT2	PF-07208254	PF-07238025	PF-07247685	Apo
Count	6000	6000	6000	6000	6000
Median	295	295	298	298	294
Outliers	24	29	57	25	36

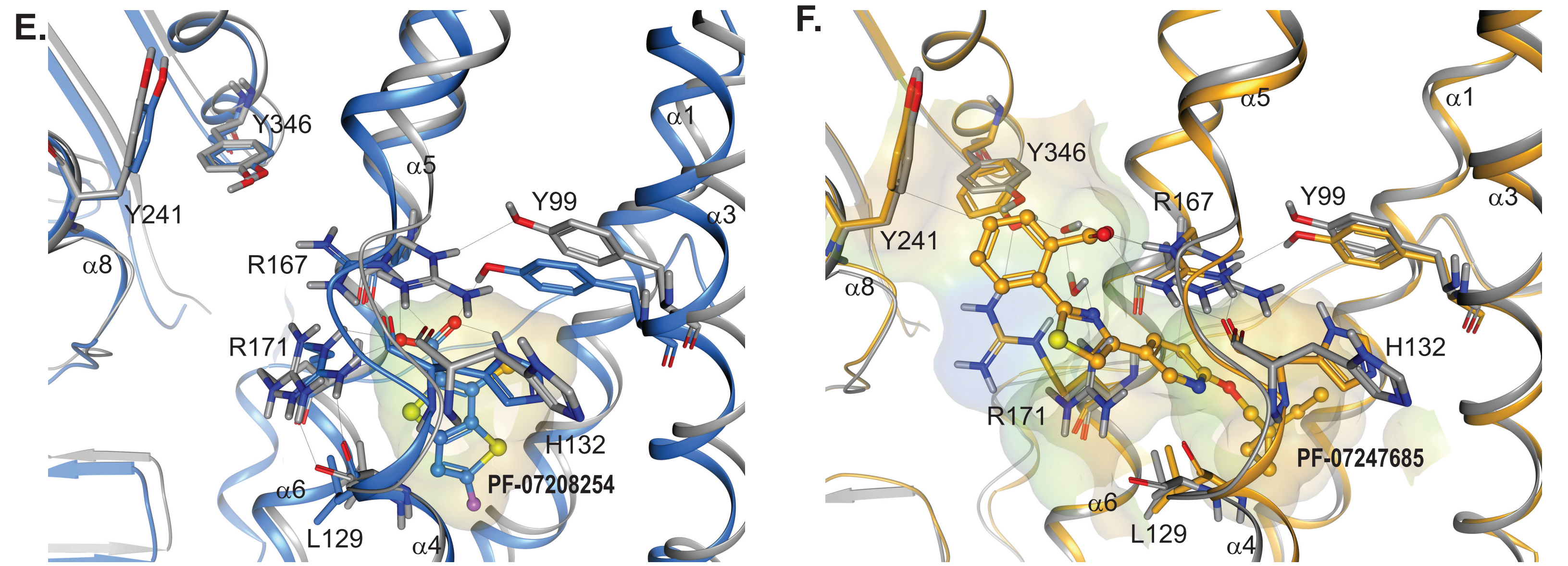

Supplementary Figure 10

Supplementary Figure 10. BDK active-site cleft narrows and fewer hydrogen bonds are observed with binding of destabilizers and widens with binding of stabilizers. A. Illustration of active-site cleft (gray surface) where E1 binds and is phosphorylated by BDK, and center-of-mass (CM) distance between helices α5 and α8 (yellow). Stabilizers PF-07238025 (not shown) and PF-07247685 (orange) protrude into the active-site cleft, physically blocking E1 from binding. **B.** Distribution of BDK $CM_{\alpha5-\alpha8}$ over MD simulations. **C.** Distribution of the number of hydrogen bonds formed by residues in the BDK lipoyl-binding pocket over the course of MD simulations. **D.** Distribution of the number of hydrogen bonds formed by all BDK residues over the course of MD simulations. **E.** Differences in protein-protein and protein-ligand interactions when BDK is bound to a destabilizer PF-07208254, blue) compared to apo structure (gray). **F.** Differences in protein-protein and protein-ligand interactions when BDK is bound to a stabilizer (PF-07247685, orange) compared to apo structure (gray).

Supplementary Figure 10

Supplementary Tables

Supplementary Table 1. Potency of BDK inhibitors

Compound	Structure	BDK in vitro IC_{50} (nM)[a]	BDK SPR K_D (nM)[a]	Human skeletal myocyte IC_{50} (nM)[a]
**S1**	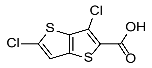	600 ± 51	75 ± 2.0[b]	ND[c]
**S2**	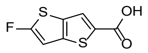	210 ± 10	270 ± 31	930 ± 120
**S3**	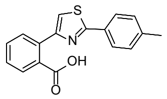	1200 ± 490	5800 ± 4400	>60,000

[a]Potency values are ± standard error with an N ≥ 3 except where noted.
[b]N = 2
[c]Not Determined

Supplementary Table 2. Cerep Bioprint Off-Target Panel

			% Inhibition or IC_{50}/EC_{50}		
**Target**	**Format**	**Single Point Compound Test Concentration (uM) or IC_{50}/EC_{50}**	**PF-07208254**	**PF-07238025**	**PF-07247685**
Abl Kinase	Kinase Inhibition	10		9	11
Abl Kinase	Kinase Inhibition	100	42		
Acetylcholinesterase	Enzyme Inhibition	10		11	-4
Acetylcholinesterase	Enzyme Inhibition	100	-4		
Adenosine A1 Receptor	Agonism	10		-1	0
Adenosine A1 Receptor	Agonism	100	0		
Adenosine A2a Receptor	Agonism	10		1	1
Adenosine A2a Receptor	Agonism	100	0		
Adrenergic Alpha 1a Adrenoceptor	Agonism	10		-1	1
Adrenergic Alpha 1a Adrenoceptor	Agonism	100	8		
Adrenergic Alpha 1a Adrenoceptor	Antagonism	10		13	14
Adrenergic Alpha 1a Adrenoceptor	Antagonism	100	21		
Adrenergic Alpha 2a receptor	Agonism	10		-2	0
Adrenergic Alpha 2a receptor	Agonism	100	-1		
Adrenergic Alpha 2b Adrenoceptor	Agonism	10		0	-1
Adrenergic Alpha 2b Adrenoceptor	Agonism	100	-4		
Adrenergic Alpha 2b Adrenoceptor	Antagonism	10		9	-1
Adrenergic Alpha 2b Adrenoceptor	Antagonism	100	-2		
Adrenergic Beta 1 Adrenoceptor	Agonism	10		3	6
Adrenergic Beta 1 Adrenoceptor	Agonism	100	2		
Adrenergic Beta 1 Adrenoceptor	Antagonism	10		20	27

Adrenergic Beta 1 Adrenoceptor	Antagonism	100	8		
Adrenergic Beta 2 Adrenoceptor	Agonism	10		4	-6
Adrenergic Beta 2 Adrenoceptor	Agonism	100	-5		
Adrenergic Beta 2 Adrenoceptor	Antagonism	10		8	3
Adrenergic Beta 2 Adrenoceptor	Antagonism	100	10		
AMPA Receptor	Ion Channel Binding	10		-13	5
AMPA Receptor	Ion Channel Binding	100	7		
Androgen Receptor	NHR Binding	10		-6	-8
Androgen Receptor	NHR Binding	100	-4		
Angiotensin 1	Agonism	10		-4	-8
Angiotensin 1	Agonism	100	3		
Angiotensin 1	Antagonism	10		-1	5
Angiotensin 1	Antagonism	100	18		
Angiotensin Coverting Enzyme	Enzyme Inhibition	10		-33	-31
Angiotensin Coverting Enzyme	Enzyme Inhibition	100	-4		
Aurora A Kinase	Kinase Inhibition	10		-8	-7
Aurora A Kinase	Kinase Inhibition	100	15		
Cannabinoid CB1	Agonism	10		32	39
Cannabinoid CB1	Agonism	100	55		
Cannabinoid CB1	Agonism	EC_{50}	180,000 nM		
Cannabinoid CB1	Antagonism	10		-1	-4
Cannabinoid CB1	Antagonism	100	-10		
Cholecystokinin 2	Agonism	10		2	-8
Cholecystokinin 2	Agonism	100	0		
Choline Transporter	Transporter Binding	10		-8	-12
Choline Transporter	Transporter Binding	100	-15		
Corticotrophin Releasing Factor 1 (CRF1) Receptor	Agonism	10		-1	4
Corticotrophin Releasing Factor 1 (CRF1) Receptor	Agonism	100	-2		
Corticotrophin Releasing Factor 1 (CRF1) Receptor	Antagonism	10		32	11
Corticotrophin Releasing Factor 1 (CRF1) Receptor	Antagonism	100	39		
Cyclooxygenase 2	Enzyme Inhibition	10		3	4
Cyclooxygenase 2	Enzyme Inhibition	100	0		
Delta Opioid Receptor	Agonism	10		-2	-1
Delta Opioid Receptor	Agonism	100	-2		
Dopamine 2S	Agonism	10		-6	-17
Dopamine 2S	Agonism	100	-29		
Dopamine 2S	Antagonism	10		3	-7
Dopamine 2S	Antagonism	100	-7		
Dopamine D1	Agonism	10		1	4
Dopamine D1	Agonism	100	-4		
Dopamine D1	Antagonism	10		10	5
Dopamine D1	Antagonism	100	-5		
Dopamine Transporter	Transporter Binding	10		-4	-1
Dopamine Transporter	Transporter Binding	100	7		
EGFR Kinase	Kinase Inhibition	10		-1	-6
EGFR Kinase	Kinase Inhibition	100	19		
Endothelin A	Agonism	10		-1	-4
Endothelin A	Agonism	100	4		
GABA Transporter	Transporter Binding	10		-9	-8
GABA Transporter	Transporter Binding	100	0		
GABAa (benzodiazepine site)	Ion Channel Binding	10		-14	13

GABAa (benzodiazepine site)	Ion Channel Binding	100	-9		
GABAa (Cl Channel)	Ion Channel Binding	10		11	19
GABAa (Cl Channel)	Ion Channel Binding	100	19		
GABAa1 Receptor	Ion Channel Binding	10		-28	-21
GABAa1 Receptor	Ion Channel Binding	100	-12		
Glucocorticoid Receptor	NHR Binding	10		-2	0
Glucocorticoid Receptor	NHR Binding	100	4		
Histamine H1	Agonism	10		-4	-5
Histamine H1	Agonism	100	-1		
Histamine H1	Antagonism	10		13	-2
Histamine H1	Antagonism	100	17		
Histamine H2	Agonism	10		2	1
Histamine H2	Agonism	100	0		
Histamine H3	Agonism	10		28	4
Histamine H3	Agonism	100	15		
Kappa Opioid	Agonism	10		-18	-7
Kappa Opioid	Agonism	100	-3		
Kdr Kinase (VEGFR2)	Kinase Inhibition	10		5	-11
Kdr Kinase (VEGFR2)	Kinase Inhibition	100	57		
Kdr Kinase (VEGFR2)	Kinase Inhibition	IC_{50}	76,000 nM		
LCK Kinase	Kinase Inhibition	10		0	-2
LCK Kinase	Kinase Inhibition	100	-4		
L-Type Ca2+ Channel (dihydropyridine site)	Ion Channel Binding	10		-8	-2
L-Type Ca2+ Channel (dihydropyridine site)	Ion Channel Binding	100	1		
L-Type Ca2+ Channel (diltiazem site)	Ion Channel Binding	10		-30	24
L-Type Ca2+ Channel (diltiazem site)	Ion Channel Binding	100	-8		
L-Type Ca2+ Channel (verapamil site)	Ion Channel Binding	10		-3	1
L-Type Ca2+ Channel (verapamil site)	Ion Channel Binding	100	-6		
Melanocortin MC2 Receptor	Agonism	10		0	0
Melanocortin MC2 Receptor	Agonism	100	0		
Melanocortin MC2 Receptor	Antagonism	10		39	18
Melanocortin MC2 Receptor	Antagonism	100	-4		
Monoamine Oxidase A	Enzyme Inhibition	10		13	12
Monoamine Oxidase A	Enzyme Inhibition	100	0		
Mu Opiod	Antagonism	10		-9	-3
Mu Opiod	Antagonism	100	2		
Mu Opioid	Agonism	10		-1	-3
Mu Opioid	Agonism	100	16		
Muscarinic 1	Agonism	10		-1	1
Muscarinic 1	Agonism	100	-1		
Muscarinic 1	Antagonism	10		14	9
Muscarinic 1	Antagonism	100	0		
Muscarinic 2	Agonism	10		21	3
Muscarinic 2	Agonism	100	39		
Muscarinic 2	Antagonism	10		-12	2
Muscarinic 2	Antagonism	100	-7		
Muscarinic 3	Agonism	10		-5	-1
Muscarinic 3	Agonism	100	0		
Muscarinic 3	Antagonism	10		7	3
Muscarinic 3	Antagonism	100	18		

Na+ Channel (site 2)	Ion Channel Binding	10		-2	31
Na+ Channel (site 2)	Ion Channel Binding	100	-2		
Neurokinin Nk1	Agonism	10		-2	-4
Neurokinin Nk1	Agonism	100	-3		
Nicotinic Acetylcholine Receptor	Ion Channel Binding	10		1	-1
Nicotinic Acetylcholine Receptor	Ion Channel Binding	100	-12		
Nicotinic Acetylcholine Receptor (alpha4, beta2 subunit containing)	Ion Channel Binding	10		-2	-4
Nicotinic Acetylcholine Receptor (alpha4, beta2 subunit containing)	Ion Channel Binding	100	-6		
NMDA Receptor (glutamate site)	Ion Channel Binding	10		-2	-5
NMDA Receptor (glutamate site)	Ion Channel Binding	100	3		
NMDA Receptor (PCP binding site)	Ion Channel Binding	10		-2	0
NMDA Receptor (PCP binding site)	Ion Channel Binding	100	-13		
Norepinephrine Transporter	Transporter Binding	10		-11	6
Norepinephrine Transporter	Transporter Binding	100	-15		
P38alpha MAP Kinase	Kinase Inhibition	10		2	3
P38alpha MAP Kinase	Kinase Inhibition	100	35		
Phosphodiesterase 3B	Enzyme Inhibition	10		5	-16
Phosphodiesterase 3B	Enzyme Inhibition	100	-9		
Phosphodiesterase 4D2	Enzyme Inhibition	10		12	-24
Phosphodiesterase 4D2	Enzyme Inhibition	100	-11		
PPAR-gamma	NHR Binding	10		10	21
PPAR-gamma	NHR Binding	100	62		
PPAR-gamma	NHR Binding	IC_{50}	86,000 nM	26,000 nM	50,000 nM
Serotonin 5-HT1a Receptor	Agonism	10		-2	-1
Serotonin 5-HT1a Receptor	Agonism	100	-1		
Serotonin 5-HT1b Receptor	Agonism	10		-3	34
Serotonin 5-HT1b Receptor	Agonism	100	39		
Serotonin 5-HT2a	Agonism	10		0	3
Serotonin 5-HT2a	Agonism	100	-5		
Serotonin 5-HT2b	Agonism	10		4	-5
Serotonin 5-HT2b	Agonism	100	-5		
Serotonin 5-HT3	Ion Channel Binding	10		-9	0
Serotonin 5-HT3	Ion Channel Binding	100	-14		
Serotonin 5-HT4e	Agonism	10		2	0
Serotonin 5-HT4e	Agonism	100	6		
Serotonin Transporter	Transporter Binding	10		-10	2
Serotonin Transporter	Transporter Binding	100	-3		
Src Kinase	Kinase Inhibition	10		5	-8
Src Kinase	Kinase Inhibition	100	11		
Thyrotopin Releasing Hormone 1 (TRH1) Receptor	Agonism	10		-2	4
Thyrotopin Releasing Hormone 1 (TRH1) Receptor	Agonism	100	4		
Thyrotopin Releasing Hormone 1 (TRH1) Receptor	Antagonism	10		20	10
Thyrotopin Releasing Hormone 1 (TRH1) Receptor	Antagonism	100	-5		
Vasopressin 1a	Agonism	10		-1	-1
Vasopressin 1a	Agonism	100	16		

Key compounds were tested against a broad off-target panel to determine selectivity for BDK activity. PF-07238025 and PF-07247685 were tested in single point at 10 µM. Because of its weaker activity PF-07208254 was tested in single point at 100 µM. No targets showed >50%

activity for PF-07238025 and PF-07247685. Three targets (shaded in table) showed >50% activity @ 100 µM for PF-07208254. These were followed up with dose response curves and demonstrated high micromolar activity. Because of the weak activity and large margins between BDK potency and these off-target potencies, pharmacology due to these off-targets in not anticipated within the context of the included experiments.

Supplementary Table 3. Kinase Off-Target Panel

	ATP = Km % Inhibition @ 10 µM			ATP = 1 mM % Inhibition @ 10 µM		
**Target**	**PF-07208254**	**PF-07238025**	**PF-07247685**	**PF-07208254**	**PF-07238025**	**PF-07247685**
ABL Proto-Oncogene 1 Non-receptor Tyrosine Kinase (ABL1)	9.0	7.0	5.1	-0.5	3.8	0.9
Aurora Kinase A (AURKA)	9.4	5.8	8.9	-2.6	0.6	2.2
Bruton agammaglobulinemia Tyrosine Kinase (BTK)	5.9	5.4	-0.7	0.8	-3.6	3.1
Cyclin Dependent Kinase 1 (CDK2/Cyclin-A)	14.6	3.4	1.2	-1.2	2.9	-1.4
Checkpoint Kinase 1 (CHEK1)	4.6	3.8	-3.1	4.3	1.5	4.5
Checkpoint Kinase 2 (CHEK2)	6.7	-2.7	3.8	-0.7	1.0	-0.2
Casein Kinase 1 delta (CKI-delta)	9.1	2.3	6.0	NT	NT	NT
MAP Kinase Interacting serine/threonine Kinase 1 (MKNK1)	15.1	-2.3	0.5	NT	NT	NT
NUAK family, SNF1-like kinase, 1 (NUAK1)	10.3	1.4	-18.2	NT	NT	NT
DNA-activated, catalytic polypeptide protein kinase (PRKDC)	8.3	-4.7	5.4	NT	NT	NT
CSK tyrosine Kinase	2.9	4.8	4.3	NT	NT	NT
C-terminal Src Kinase (v-src sarcoma (Schmidt-Ruppin A-2) viral oncogene homolog (avian) (SRC)	5.6	6.6	17.3	-4.8	7.8	-1.0
Epidermal Growth Factor Receptor (erythroblastic leukemia viral (v-erb)	-0.9	3.9	-2.6	-7.2	-2.3	1.2
EPH Receptor A2 (EPHA2)	0.5	7.0	0.6	-5.6	4.7	5.6
ERBB-4 receptor protein tyrosine kinase (Tyrosine kinase-type cell surface receptor HER4)	-11.4	1.4	7.2	NT	NT	NT
Fibroblast Growth Factor Receptor 1 (FGFR1)	-0.5	6.4	8.8	-0.5	3.3	2.1
Glycogen Synthase Kinase 3 Beta (GSK3B)	9.7	4.0	11.0	0.7	1.6	3.7
Tyrosine-protein kinase HCK (Haemopoietic cell kinase)	9.9	5.6	4.5	NT	NT	NT
IkB kinase beta subunit (IKK BETA)	4.8	1.0	-0.0	NT	NT	NT
Insulin Receptor (INSR)	3.1	5.9	3.4	0.0	4.4	-0.4
Interleukin 1 Receptor Associated Kinase 4 (IRAK4)	6.2	3.4	-0.9	-2.1	5.7	2.9
Janus Kinase 3 (JAK3)	0.9	0.4	0.8	-4.1	-1.7	-0.0
Kinase Insert Domain Receptor (a type III receptor tyrosine kinase) (KDR, VEGFR2)	1.1	4.1	2.0	5.6	3.0	0.1
LCK Proto-Oncogene Src Family Tyrosine Kinase, lymphocyte-specific protein kinase (LCK)	20.6	8.1	5.9	9.9	8.5	3.0
Mitogen-Activated Protein Kinase 1 (MAPK1)	9.4	0.3	7.0	2.8	5.2	2.4

Mitogen-Activated Protein Kinase-Activated Protein Kinase 2 (MAPKAPK2)	3.4	-0.3	2.2	-1.5	4.4	3.3
MAP/Microtubule Affinity Regulating Kinase 1 (MARK1)	2.1	6.0	4.3	8.4	2.4	7.5
MET Proto-Oncogene Receptor Tyrosine Kinase (hepatocyte growth factor receptor) (MET)	-3.1	2.8	-0.3	-10.2	-2.7	-2.2
Misshapen-like Kinase 1 (zebrafish)	-3.3	19.7	21.9	NT	NT	NT
Mitogen and stress-activated protein kinase-1 (MSK1)	2.7	1.5	-1.4	NT	NT	NT
Serine/Threonine Kinase 3 (STE20 homolog, yeast) (STK3)	5.5	-9.3	-1.8	-2.3	-1.7	-7.6
NEK6	7.1	6.5	1.2	NT	NT	NT
Ribosomal protein S6 kinase	2.5	3.5	2.7	NT	NT	NT
p21 protein (Cdc42/Tac)-activated kinase 4 (PAK4)	6.0	4.2	8.3	0.9	2.8	3.7
Proto-oncogene kinase PIM-1	44.5	-2.6	-2.6	NT	NT	NT
Pim-2 Proto-Oncogene Serine/Threonine Kinase (PIM2)	22.3	-8.4	0.1	-5.2	1.3	0.9
Protein Kinase cAMP-dependent, Catalytic Subunit Alpha (PRKACA)	2.7	3.2	2.3	-2.4	2.6	0.3
AKT Kinase (Protein Kinase B), v-akt murine thymoma viral oncogene homolog 1 (AKT1)	6.7	2.2	2.4	-0.8	-0.6	-0.8
MAP kinase activated protein kinase 5 (MAPKAPK5)	6.6	1.4	2.3	NT	NT	NT
Rho Associated, Coiled-Coil Containing Protein Kinase 1 (ROCK1)	3.6	-0.8	2.4	0.8	-1.1	-0.9
Rho kinase (ROCK2)	-5.8	-3.5	5.5	NT	NT	NT
Ribosomal protein S6 kinase II alpha 1 (Rsk1)	3.3	1.2	2.4	NT	NT	NT
Ribosomal protein S6 kinase II alpha 3 (Rsk2)	-0.7	-11.1	-1.6	NT	NT	NT
Mitogen-activated protein kinase 12 (MAPK12/ERK6/P38 gamma)	13.3	5.6	4.6	NT	NT	NT
Mitogen-activated protein kinase 13 (MAPK13/P38 gamma)	3.6	0.1	-0.7	NT	NT	NT
Serum/Glucocorticoid Regulated Kinase 1 (SGK1)	-0.1	-11.1	0.7	1.9	-12.3	-5.1
Serine kinase SRPK1	2.8	30.4	12.6	NT	NT	NT
TEK Receptor Tyrosine Kinase, endothelial (TEK)	0.3	3.6	0.6	0.7	3.6	8.6
Neurotrophic Receptor Tyrosine Kinase, type 1 (NTRK1)	5.6	9.6	14.5	5.8	15.5	10.0
Eukaryotic elongation factor-2 kinase	2.3	0.9	-1.5	NT	NT	NT
Mitogen-activated protein kinase kinase kinase 9 (MAPK3K9) (mixed lineage kinase 1)	3.2	-1.1	6.3	NT	NT	NT
RAC-beta serine/threonine kinase (AKT2 kinase)	0.2	1.5	5.5	NT	NT	NT
TAO Kinase 2 (TAOK2)	-2.5	-0.4	-4.7	1.4	-1.0	-1.8
Aurora kinase B (AURKB)	3.0	9.9	17.3	NT	NT	NT
Calcium/Calmodulin Dependent Protein Kinase II Alpha (CAMK2A)	6.8	-0.5	2.8	-0.3	-0.8	3.5
Casein Kinase 1 Alpha 1 (CSNK1A1)	9.2	1.6	3.1	-0.6	3.8	2.1
Casein Kinase 2 Alpha prime polypeptide (CSNK2A2)	24.5	2.1	7.5	7.3	0.9	1.9
Dual-specificity tyrosine-(Y)-phosphorylation regulated kinase (DYRK)	11.8	7.1	2.1	NT	NT	NT
FKBP-rapamycin associated protein (FRAP, mTOR)	5.1	-0.1	0.4	NT	NT	NT

Mitogen-Activated Protein Kinase Kinase Kinase Kinase 4 (MAPK4K4)	11.9	17.2	25.5	-7.5	-0.7	3.2
Serine/Threonine-Protein Kinase MASK (MST4)	16.7	5.9	2.9	3.7	0.1	-2.9
Myosin Light Chain Kinase 2 (MYLK2)	-3.6	1.1	1.4	-0.1	-1.6	1.7
Phosphorylase B kinase gamma catalytic chain skeletal muscle isoform	1.8	0.8	2.2	NT	NT	NT
PLK1 kinase	1.8	3.0	10.1	NT	NT	NT
Phosphatidylinositol 4-kinase, catalytic, beta (PI4KB)	7.9	3.1	7.1	NT	NT	NT
Phosphatidylinositol-3-kinase, class 3 (PIK3C3)	7.3	4.0	3.0	NT	NT	NT
Phosphatidylinositol-3-kinase, catalytic, delta polypeptide (PIK3CD)	6.4	12.2	-7.0	NT	NT	NT
Sphingosine kinase 1 (SPHK1)	-15.9	-86.4	-82.5	NT	NT	NT
3-phosphoinositide dependent protein kinase-1 (PDPK1)	-3.2	1.3	-0.6	-3.1	0.8	2.0
Phosphatidylinositol-3-kinase, class 2, alpha polypeptide (PIK3C2A)	2.6	-11.1	-4.2	NT	NT	NT
Phosphatidylinositol-3-kinase, catalytic, beta polypeptide (PIK3CB)	3.1	-3.0	-3.5	NT	NT	NT
Phosphatidylinositol-3-kinase, catalytic, gamma polypeptide (PIK3CG)	4.8	-1.5	-4.6	NT	NT	NT
Phosphatidylinositol-3-kinase, class 2, alpha polypeptide (PIK3C2A)	1.6	-6.3	5.5	NT	NT	NT
Mitogen-Activated Protein Kinase 14 (MAPK14)	3.0	0.5	3.5	4.0	-3.7	7.1
MAP/microtubule affinity-regulating kinase 3 (MARK3)	5.0	3.1	2.1	NT	NT	NT
Protein kinase, DNA-activated, catalytic polypeptide (PRKDC)	8.3	-4.7	5.4	NT	NT	NT
Protein kinase C, alpha (PRKCA)	3.8	-2.0	14.8	NT	NT	NT
Protein kinase C, beta II isoform (PRKCB2)	-1.7	2.6	9.4	1.9	-1.7	4.0

	**ATP = 10 uM<br>% Inhibition @ 10 µM**		
**Target**	**PF-07208254**	**PF-07238025**	**PF-07247685**
Phosphatidylinositol 4-kinase, catalytic, alpha (PI4KA)	8.2	2.6	-3.1
Phosphatidylinositol-3-kinase, class 2, beta polypeptide (PIK3C2B)	3.5	-6.7	2.0
Sphingosine kinase 2 (SPHK2)	-11.8	-9.4	3.9
	**ATP = 100 uM<br>% Inhibition @ 10 µM**		
Mitogen-activated protein kinase 8 (MAPK8 / JNK1)	-0.6	-0.2	2.1
Mitogen-activated protein kinase 9 (MAPK9 / JNK2)	-3.0	5.4	4.3
MEK1	4.1	10.1	11.2
Calcium/calmodulin-dependent protein kinase I (CAMK1)	-7.9	-3.7	-5.1
	**Lantha Binding<br>% Inhibition @ 10 µM**		
TTK protein kinase (TTK)	16.2	4.1	3.6

Calcium/calmodulin-dependent protein kinase kinase 2, beta (CAMKK2)	7.8	1.8	1.8
Myosin light chain kinase (MYLK)	3.3	1.0	1.3
MAP kinase interacting serine/threonine kinase 2 (MKNK2)	11.8	-2.1	1.2
TGF-beta activated kinase 1/MAP3K7 binding protein 1 (MAP3K7IP1)	2.6	3.6	-1.9
Mitogen-activated protein kinase kinase kinase 11 (MAP3K11)	6.8	-3.5	-2.2
Protein kinase N2 (PKN2)	8.1	4.1	-1.7
Protein kinase, AMP-activated, alpha 1 beta 1 gamma 2 complex (PRKA A1)	5.9	-1.8	0.5
Dual-specificity tyrosine-(Y)-phosphorylation regulated kinase 2 (DYRK)	29.2	13.3	1.9
Mitogen-activated protein kinase 15 (MAPK15)	18.3	-4.9	2.4
Transforming growth factor, beta receptor II (70/80kDa) (TGFBR2)	15.9	4.7	15.1

Key compounds were tested against multiple Invitrogen kinase panels, which included 96 kinases, to determine kinase selectivity. PF-07208254, PF-07238025, and PF-07247685 were tested at 10 µM compound concentration for % inhibition of select protein and lipid kinases at the ATP concentrations noted in the table. Several other kinases were probed for inhibition of binding in a Lantha screen format assay as noted in the table. All % inhibition values were < 30% with the exceptions (shaded in table) of PF-07208254 showing 44.5% inhibition of PIM-1 at K_m ATP and PF-07238025 showing 30.4% inhibition of Serine Kinase SRPK1 at K_m ATP. In a follow-up dose response, PF-07208254 demonstrated an IC_{50} = 14.4 µM for PIM-1. Because of this weak activity and large margins between BDK potency (PF-07208254 BDK IC_{50} = 110 nM) and PF-07238025 BDK IC_{50} = 4.5 nM), and these off-target potencies, pharmacology due to these off-targets is not anticipated within the context of the included experiments.

X-ray Crystal Structures:

Supplementary Table 4. Data collection and refinement statistics (molecular replacement)

	PF-07208254	S3	PF-07247685
**Data collection**			
Space group	$P6_422$	$P4_122$	$P4_122$
Cell dimensions			
a, *b*, *c* (Å)	132.27, 132.27, 121.70	117.8, 117.8,154.8	118.370,118.370,156.230
α, β, γ (°)	90, 90, 120	90, 90, 90	90, 90, 90
Resolution (Å)	114.5-2.55 (2.70-2.55) *	117.8-2.79 (3.08-2.79)	47.7-3.15(3.23-3.15)
R_{merge}	0.15(2.49)	0.112(2.74)	0.113(2.07)
$I / \sigma I$	13.5(1.2)	17.3(1.7)	17.7(1.6)
Completeness (spherical, %)	86.2(28.3)	63.1(12.7)	93.3(54.7)
Completeness (ellipsoidal, %)	90.4(39.9)	86.0(73.5)	96.0(89.2)
Redundancy	18.0(13.9)	12.8(12.5)	13.1(13.2)

**Refinement**			
Resolution (Å)	114.5-2.54	117.8(2.79)	47.7(3.15)
No. reflections	18,176	17,480	18,537
R_{work} / R_{free}	0.231/0.270	0.248/0.296	0.240/0.279
No. atoms			
Protein	2,564	5,042	5,163
Ligand/ion	42	124	121
Water	28	12	13
B-factors[¥]			
Protein	80.7	97.2/163	99.9/142
Ligand	81.3	79.8/155	95.3/119
Solvent	63.9	64.02/132	74.5
Wilson Plot	83.56	92.4	102.3
R.m.s. deviations			
Bond lengths (Å)	0.008	0.009	0.008
Bond angles (°)	0.91	0.92	0.91

*One crystal was used for each data set. Values in parentheses are for the highest-resolution shell. Anisotropic resolution cutoff was used during data merging and scaling, and both spherical and ellipsoidal completeness are reported.

[¥]For **S3** and PF-07247685, the average B factors of protein and bound inhibitors are listed separately for chain A and B in the asymmetric unit. Chain A is more ordered than chain B, and chain B was tightly restrained to chain A during model building and refinements. There is only one chain in the PF-07208254 crystal.

Supplementary Methods

Chemical Synthesis:

BT2 was obtained from Enamine in 95% purity (Monmouth Junction, NJ, USA); Catalog number EN300-00845. Compound **S1** can be synthesized according to a literature procedure, and showed 98.2% purity as determined by LCMS.[11] Compound **S2** is commercially available from multiple vendors including Aurora Fine Chemicals LLC (San Diego, CA); catalog number 182.043.609. Material used herein was 96.5% pure as determined by LCMS. Compound **S3** is commercially available from multiple vendors including Aurora Fine Chemicals LLC (San Diego, CA); catalog number 100.479.432. Material used herein was 95.7% pure as determined by LCMS. The synthesis of all other included compounds is below.

Methods:

Reactions were performed in air or, when oxygen- or moisture-sensitive reagents or intermediates were employed, under an inert atmosphere (nitrogen or argon). When appropriate, reaction apparatuses were dried under dynamic vacuum using a heat gun, and anhydrous solvents (Sure-Seal™ products from Aldrich Chemical Company, Milwaukee, Wisconsin or DriSolv™ products from EMD Chemicals, Gibbstown, NJ) were employed. Commercial solvents and reagents were used without further purification. When indicated, reactions were heated by microwave irradiation using Biotage Initiator or Personal Chemistry Emrys Optimizer microwaves or the like. Reaction progress was monitored using thin-layer chromatography (TLC), liquid chromatography-mass spectrometry (LCMS) and high-performance liquid chromatography (HPLC) analyses. TLC was performed on pre-coated silica gel plates with a fluorescence indicator (254 nm excitation wavelength) and visualized under UV light and/or with I_2, $KMnO_4$, $CoCl_2$, phosphomolybdic acid, and/or ceric ammonium molybdate stains. LCMS data were acquired on an Agilent 1100 Series instrument with a Leap Technologies autosampler, Gemini C18 columns, acetonitrile/water gradients, and either trifluoroacetic acid, formic acid, or ammonium hydroxide modifiers or similar equipment. The column eluent was analyzed using a Waters ZQ mass spectrometer scanning in both positive and negative ion modes from 100 to 1200 Da. Other similar instruments were also used. HPLC data were acquired on an Agilent 1100 Series instrument using Gemini or XBridge C18 columns, acetonitrile/water gradients, and either trifluoroacetic acid or ammonium hydroxide modifiers and comparable equipment. Purifications were performed by medium performance liquid chromatography (MPLC) using Isco CombiFlash Companion, AnaLogix IntelliFlash 280, Biotage SP1, or Biotage Isolera One instruments and pre-packed Isco RediSep or Biotage Snap silica cartridges and the like. Chiral purifications were performed by chiral supercritical fluid chromatography (SFC) using Berger or Thar instruments and similar instruments; ChiralPAK-AD, -AS, -IC, Chiralcel-OD, or –OJ columns; and CO_2 mixtures with methanol, ethanol, isopropanol, or acetonitrile, alone or modified using trifluoroacetic acid or $iPrNH_2$. UV detection was used to trigger fraction collection.

Mass spectrometry data are reported from LCMS analyses. Mass spectrometry (MS) was performed via atmospheric pressure chemical ionization (APCI), electrospray Ionization (ESI), electron impact ionization (EI) or electron scatter (ES) ionization sources. High resolution mass spectrometry (HRMS) was performed on a Sciex TripleTOF 5600+ (Sciex, Ontario, Canada) with DuoSpray ionization source. The LC instrument includes an Agilent (Agilent Technologies, Wilmington, DE) 1200 binary pump, Agilent 1200 autosampler, Agilent 1200 column compartment, and Agilent 1200 DAD. The instrument acquisition and data handling were done with Sciex Analyst TF version 1.7.1. Prior to acquisition, instrument was calibrated with less than 5 ppm accuracy. During acquisition, a calibration run was performed initially and after every 5 injections using the Sciex positive polarity tuning mix. Elution Conditions: Column: Waters XSelect

HSS T3, 2.1 x 50mm, 2.5 µm particle size; Column Temperature 60 °C; Solvent A: Water (0.1% formic acid); Solvent B: Acetonitrile (0.1% formic acid); Gradient: Initial 5% B, hold for 0.10 min, 5-95% B in 2.8 min, 95-5% B in 0.20 min, 3.5 min total runtime; Flow rate 0.8 mL/min. TOF Conditions: ESI in Positive Mode; The spray chamber: Gas 1 and 2 at 60, curtain gas at 40, temperature 600 °C, IonSpray voltage 5500 V, declustering potential 100, collision energy 10. The acquisition was done in TOF MS mode with range of 100-2000 amu with accumulation time of 0.20 sec. ESI in Negative Mode; The spray chamber: Gas 1 and 2 at 60, curtain gas at 40, temperature 600 °C, IonSpray voltage -4500 V, declustering potential -100, collision energy -10. The acquisition is done in TOF MS mode with range of 100-2000 amu with accumulation time of 0.20 sec. Proton, carbon, and fluorine nuclear magnetic spectroscopy (^{1}H NMR, ^{13}C NMR, ^{19}F NMR) chemical shifts are given in parts per million downfield from tetramethylsilane and were recorded on 101, 300, 400, 500, or 600 MHz Varian spectrometers. Chemical shifts are expressed in parts per million (ppm, δ) referenced to the deuterated solvent residual peaks. The peak shapes are described as follows: s, singlet; d, doublet; t, triplet; q, quartet; quin, quintet; m, multiplet; br s, broad singlet; app, apparent. Analytical SFC data were acquired on a Berger analytical instrument as described above. Optical rotation data were acquired on a PerkinElmer model 343 polarimeter using a 1 dm cell. Silica gel chromatography was performed primarily using a medium pressure Biotage or ISCO systems using columns pre-packaged by various commercial vendors including Biotage and ISCO.

Unless otherwise noted, chemical reactions were performed at room temperature (about 23 degrees Celsius). HPLC, UPLC, LCMS, and SFC retention times were measured using the methods noted in the procedures. Products were generally dried under vacuum before being carried on to further reactions or submitted for biological testing. Mass spectrometry data is reported from liquid chromatography-mass spectrometry (LCMS), atmospheric pressure chemical ionization (APCI) instrumentation. Chemical shifts for nuclear magnetic resonance (NMR) data are expressed in parts per million (ppm, δ) referenced to residual peaks from the deuterated solvents employed.

Synthesis of 3-chloro-5-fluorothieno[3,2-b]thiophene-2-carboxylic acid (**PF-07208254**)

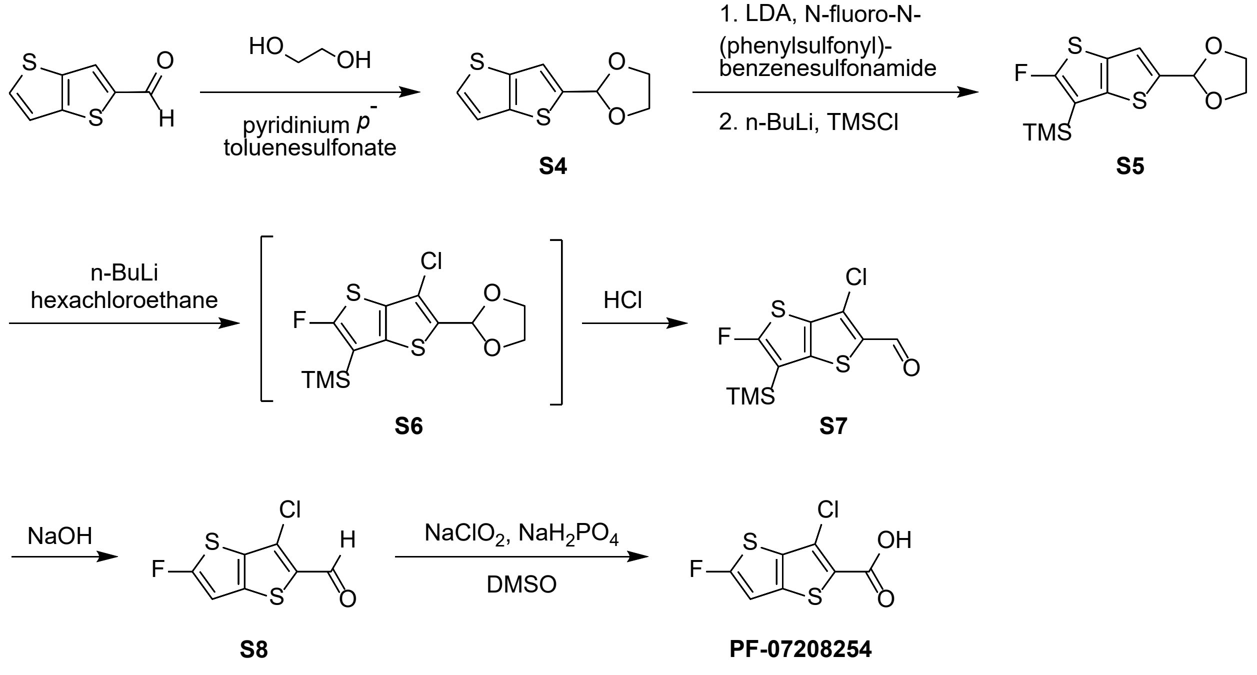

Step 1. Synthesis of 2-(thieno[3,2-b]thiophen-2-yl)-1,3-dioxolane (**S4**)

To a solution of thieno[3,2-*b*]thiophene-2-carbaldehyde (180 g, 1.07 mol) in cyclohexane (3 L, 0.36 M) was added pyridinium *p*-toluenesulfonate (26.9 g, 0.107 mol) and ethylene glycol (597 mL, 10.7 mol). The reaction mixture was heated to 90 °C for 40 hours, in an apparatus equipped with a Dean-Stark trap. The resulting mixture was diluted with sodium chloride (aq., 2 L) and the resulting organic layer was separated. The aqueous layer was extracted with ethyl acetate (3 x 1 L) and the combined organic layers were dried over sodium sulfate, filtered, and concentrated *in vacuo* to afford **S4** as a light brown solid (230 g). This material was used for next step directly. ^{1}H NMR (400 MHz, DMSO-d_6) δ 7.68 (d, *J* = 5.3 Hz, 1H), 7.57 (s, 1H), 7.43 (dd, *J* = 5.3, 0.7 Hz, 1H), 6.10 (s, 1H), 4.07 – 4.00 (m, 2H), 4.00 – 3.93 (m, 2H); HRMS (m/z): $[M+H]^+$ calcd. for $C9H9O2S2^+$, 213.0038; found, 213.0038; 2.01 min; 96%. HPLC: 8.649 min, 95% pure.

Step 2. Synthesis of [5-(1,3-dioxolan-2-yl)-2-fluorothieno[3,2-b]thiophen-3-yl](trimethyl)silane (**S5**).

A solution of **S4** (120.0 g, 564 mmol) in a mixture of toluene (1.5 L) and tetrahydrofuran (0.9 L) was cooled to −78 °C. Lithium diisopropylamide (2.0 M solution in tetrahydrofuran / heptane / ethylbenzene, 367 mL, 1.3 eq.) was added in a drop-wise manner at a rate such that the internal temperature of the reaction mixture remained below −72 °C. The reaction mixture was stirred at this temperature over 1 hour followed by dropwise addition of a *N*-fluoro-*N*-(phenylsulfonyl)benzenesulfonamide (231.7 g, 734 mmol) solution in tetrahydrofuran (5 volumes) at −78 °C. The reaction mixture was stirred at this temperature for 1 hour followed by addition of sodium hydroxide (15%, 20 volumes). Once at ambient temperature, the aqueous phase was extracted with isopropyl acetate (5 volumes). The combined organic layers were dried over sodium sulfate, filtered, and concentrated *in vacuo* to afford 120 g of a crude brown solid. This material was used for next step directly.

This solid (120 g) was dissolved in tetrahydrofuran (2.3 L), cooled to −78 °C, and treated in a drop-wise manner with *n*-butyllithium (2.5 M solution in hexanes; 417 mL, 2 eq.) at a rate that maintained the internal reaction temperature below −72 °C. After the reaction mixture had been stirred at −78 °C for 1 hour, a solution of trimethylsilyl chloride (113.2 g, 2 eq.) in tetrahydrofuran (1 volume) was added, while again maintaining the internal reaction temperature below −72 °C. The reaction mixture was then stirred at −78 °C for 1 hour, whereupon an aliquot was partitioned between diethyl ether and saturated aqueous sodium bicarbonate solution. GCMS analysis of the organic layer of this aliquot indicated conversion to **S5**: GCMS *m/z* 302.1 $[M^+]$. The reaction mixture was quenched at −78 °C by addition of saturated aqueous sodium bicarbonate solution (10 volumes), and the resulting mixture was allowed to warm to room temperature. The aqueous phase was extracted with isopropyl acetate (5 volumes). The organic layer was concentrated and purified by silica gel (3 g/g), eluted with heptanes : methyl *tert*-butyl ether (10:1). The organic phase was concentrated to 2-3 volumes, then slurried with heptanes (3 volumes). The mixture was filtered, and the residue dried under nitrogen to give **S5** as a yellowish solid (150 g, 88% yield). ^{1}H NMR (400 MHz, methanol-d_4) δ 7.24 (s, 1H), 6.00 (s, 1H), 4.04 – 3.96 (m, 2H), 3.95 – 3.86 (m, 2H), 0.29 (s, 9H); ^{19}F NMR (400 MHz, methanol-d_4) δ -113.92 (s, 1H).

Step 3. Synthesis of 3-chloro-5-fluoro-6-(trimethylsilyl)thieno[3,2-b]thiophene-2-carbaldehyde (**S7**)

A tetrahydrofuran (1.8 L, 15 volumes) solution of **S5** (150 g, 1 eq.) was cooled down to −78 °C followed by dropwise addition of *n*-BuLi (2.5 M solution in hexanes, 260 mL, 1.3 eq), at a rate that maintained the internal temperature below −72 °C. After completion of the addition, the reaction mixture was allowed to stir at −78 °C for 1 hour, whereupon a solution of hexachloroethane (165.7 g, 1.4 eq.) in tetrahydrofuran (5 volumes) was added dropwise, in a manner that maintained the internal reaction temperature below −72 °C. The reaction mixture was stirred for a further 1 hour at −78 °C. The reaction mixture, containing **S6**, was quenched with HCl (4 N, 5 volumes) and stirred at room temperature for 6 hours. The aqueous mixture was extracted with isopropyl acetate (5 volumes). The organic phase was concentrated to 2-3 volumes, then slurried with isopropyl alcohol (3 volumes). The mixture was filtered, and the residue was dried under nitrogen to give crude material as a yellowish solid. The crude product was slurried with a tetrahydrofuran : water co-mixture (1/1; 20 volumes) and the residue was then dried under nitrogen to afford **S7** as a yellowish solid (87 g, 60% yield). ^{1}H NMR (400 MHz, DMSO-d_6) δ 9.87 (s, 1H), 0.26 (s, 9H); ^{13}C NMR (101 MHz, DMSO-d_6) δ 182.36, 174.04, 171.07, 134.64 (d, J = 4.8 Hz), 127.40, 115.98, 115.75, -0.51 (d, J = 1.8 Hz); ^{19}F NMR (400 MHz, DMSO-d_6) δ -100.08 (s, 1H); HRMS (m/z): [M+H]$^+$ calcd. for C10H11ClFOS2Si$^+$, 292.9688; found, 292.9688; 3.01 min; 100%. HPLC: 11.806 min, 100% pure.

Step 4. Synthesis of 3-chloro-5-fluorothieno[3,2-b]thiophene-2-carbaldehyde (**S8**)

To a methanol (30 mL) : water (30 mL) solution of **S7** (2.96 g, 10.1 mmol) was added an aqueous solution of sodium hydroxide (1 M, 0.20 mL). The resulting solution was stirred at 50 °C for 1 h. After that time additional water was added to yield a thick slurry. The beige precipitated solid was filtered, rinsed with a methanol : water (1:1 ratio; 12 mL) solution to yield **S8** (2.17 g 89% yield) as a beige solid after drying at 50 °C over 16 h. ^{1}H NMR (400 MHz, DMSO-d_6) δ 9.99 (s, 1H), 7.49 (d, J = 1.7 Hz, 1H); ^{13}C NMR (400 MHz, DMSO-d_6): δ 182.08, 169.83, 166.85, 139.70, 134.83, 126.90, 105.75; ^{19}F NMR (400 MHz, DMSO-d_6) δ -111.67 (s, 1H); HRMS (m/z): [M+H]$^+$ calcd. for C7H3ClFOS2$^+$, 220.9292; found, 220.9290; 2.26 min; 94%; HPLC: 9.393 min, 90% pure; LC/MS: 0.817 min / 220.7 [M+H].

Step 5. Synthesis of 3-chloro-5-fluorothieno[3,2-b]thiophene-2-carboxylic acid (**PF-07208254**)

A solution of sodium chlorite (20.5 g, 227 mmol) and sodium dihydrogen phosphate (27.5 g, 229 mmol) in water (100 mL) was added slowly, in a drop-wise manner, to a 0 °C solution of **S8** (10.0 g, 45.3 mmol) in a mixture of dimethyl sulfoxide (56 mL) and 2-methyltetrahydrofuran (100 mL). The reaction mixture was then allowed to warm to room temperature and was stirred at that temperature until the starting material had been completely consumed, as assessed by LCMS analysis (approximately 2 hours). The reaction mixture was then poured in portions into a cold (0 °C) saturated aqueous solution of sodium thiosulfate pentahydrate (300 mL), at a rate that maintained the temperature of the resulting mixture below 15 °C. After stirring at 10 °C for 20 minutes, the mixture was diluted with ethyl acetate (200 mL). The aqueous layer was extracted with ethyl acetate (2 x 200 mL), and the combined organic layers were washed with saturated aqueous sodium chloride solution, dried over magnesium sulfate, filtered, and concentrated *in vacuo*. The residue was stirred in a mixture of heptane and ethyl acetate (9:1, 50 mL) for about 1 hour. The resulting solid was collected via filtration and washed with a mixture of heptane and ethyl acetate (9:1, 2 x 20 mL), providing a white solid (10.58 g). This was stirred in dichloromethane for 20 minutes and filtered; the filter cake was washed with dichloromethane (2 x 20 mL) to afford *3-chloro-5-fluorothieno[3,2-*b*]thiophene-2-carboxylic acid* (**PF-07208254**) as a white solid. Yield: 10.0 g, 42.2 mmol, 93%. ^{1}H NMR (400 MHz, DMSO-d_6) δ 13.7 (br s, 1H), 7.40

(d, *J* = 1.7 Hz, 1H), ^{19}F NMR (400 MHz, DMSO-d_6) δ -116.0 (d, 1H); ^{13}C NMR (400 MHz, DMSO-d_6): δ 168.6, 165.64, 161.79, 127.86, 125.20, 122.73, 105.03.; HRMS (m/z): [M+H]$^+$ calcd. for C7H3ClFO2S2$^+$, 236.9242; found, 236.9242; 2.01 min; 100%; HPLC: 4.668 min. (99% pure).

Synthesis of 2-{2-[6-(2-methylpropoxy)pyridin-3-yl]-1,3-thiazol-4-yl}benzoic acid (**PF-07238025**)

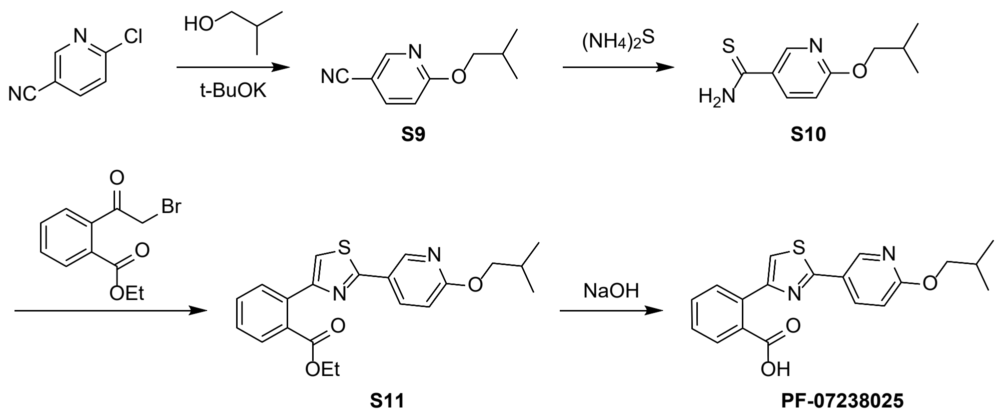

Step 1. Synthesis of 6-(2-methylpropoxy)pyridine-3-carbonitrile (**S9**)

A solution of 2-methylpropan-1-ol (32.1 g, 433 mmol) in tetrahydrofuran (100 mL) was added over 3 minutes to a 0 °C suspension of potassium *tert*-butoxide (48.6 g, 433 mmol) in tetrahydrofuran (600 mL). After the reaction mixture had been stirred for 15 minutes, a slurry of 6-chloropyridine-3-carbonitrile (40 g, 290 mmol) in tetrahydrofuran (200 mL) was added in several portions, at a rate that maintained the reaction temperature below 20 °C. When the reaction mixture had cooled to 15 °C, the ice bath was removed and the reaction mixture was allowed to stir at room temperature. After 1 hour, water (700 mL) was added, and the resulting mixture was extracted with ethyl acetate (2 x 800 mL). The combined organic layers were washed with saturated aqueous sodium chloride solution, dried over magnesium sulfate, filtered, and concentrated in vacuo; stirring of the resulting oil caused it to begin to solidify. Heptanes (800 mL) were added, and the mixture was stirred for 20 minutes, whereupon it was filtered to provide **S9**, and the filtrate was concentrated in vacuo to provide a pale-yellow oil. Some of this solid **S9** was used to seed the pale-yellow oil which further solidified upon being subjected to high vacuum to yield in total 45.9 g (260 mmol, 90% yield) of **S9** as a yellow solid. ^{1}H NMR (400 MHz, DMSO-d_6) δ 8.68 (dd, *J* = 2.4, 0.8 Hz, 1H), 8.14 (dd, *J* = 8.7, 2.4 Hz, 1H), 7.01 (dd, *J* = 8.8, 0.8 Hz, 1H), 4.12 (d, *J* = 6.7 Hz, 2H), 2.12 – 1.97 (m, 1H), 0.97 (d, *J* = 6.7 Hz, 6H); ^{13}C NMR (101 MHz, DMSO-d_6) δ 165.94, 152.66, 142.48, 117.87, 112.06, 102.18, 73.00, 27.76, 19.34. HRMS (m/z): [M+H]$^+$ calcd. for C10H13N2O$^+$, 177.1022; found, 177.1022; 2.24 min; 99%; HPLC: 8.798 min / 100% pure.

Step 2. Synthesis of 6-(2-methylpropoxy)pyridine-3-carbothioamide (**S10**)

This experiment was carried out in two equal batches. Aqueous ammonium sulfide solution (40%, 56 g, 330 mmol) was added to a solution of **S9** (28.8 g, 163 mmol) in methanol (350 mL), and the reaction mixture was allowed to stir at room temperature overnight. After the two reactions had been combined, they were concentrated in vacuo to approximately one-half of the original volume,

and treated with water (500 mL). The resulting precipitate was collected via filtration, washed with water and air-dried for 1 hour using house vacuum. The yellow solid was then stirred for 10 minutes in heptane (700 mL), and again collected via filtration. The filter cake was washed with heptane (2 x 300 mL) to afford **S10** as a solid. Combined yield: 56.36 g, 268.0 mmol, 82%. ^{1}H NMR (400 MHz, DMSO-d_6) δ 9.82 (br s, 1H), 9.50 (br s, 1H), 8.71 (d, *J* = 2.6 Hz, 1H), 8.23 (dd, *J* = 8.8, 2.6 Hz, 1H), 6.90-6.77 (m, 1H), 4.10 (d, *J* = 6.7 Hz, 2H), 2.04 (hept., 1H), 0.97 (d, *J* = 6.7 Hz, 6H). ^{13}C NMR (101 MHz, DMSO-d_6) δ 197.18, 165.63, 146.65, 139.29, 128.97, 110.03, 72.62, 27.89, 19.47. HRMS (m/z): $[M+H]^+$ calcd. for C10H15N2OS$^+$, 211.0900; found, 211.0900; 1.72 min; 98%; HPLC: 7.421 min / 100% pure.

Step 3. Synthesis of ethyl 2-{2-[6-(2-methylpropoxy)pyridin-3-yl]-1,3-thiazol-4-yl}benzoate (**S11**)

A mixture of ethyl 2-(bromoacetyl)benzoate (35.2 g, 130 mmol) and **S10** (27.3 g, 130 mmol) in ethanol (350 mL) was heated to 75 °C for 1.5 hours, whereupon it was allowed to cool to room temperature and concentrated in vacuo. The resulting oil was subjected to silica gel chromatography (Eluent: 20% ethyl acetate in heptane); the product was combined with the product of a similar reaction carried out using **S10** (23.3 g, 111 mmol), and the combined waxy solids were granulated into a powder. After this had been stirred in heptane (500 mL) for 30 minutes, the solid was collected via filtration, washed with heptane, and resuspended in fresh heptane (500 mL). This mixture was stirred at room temperature over the weekend, and then filtered; the filter cake was washed with heptane to provide **S11** as a colorless solid (58.5 g).

The impure column fractions were combined and chromatographed on silica gel (Eluent: 20% ethyl acetate in heptane) to yield additional **S11** as an oil, which solidified to a waxy solid upon drying at high vacuum. This material was suspended in heptane (40 mL) and the collected solid was granulated to a powder and again suspended in heptane (60 mL). After the mixture had been stirred 1 hour, the solid was collected by filtration, washed with heptane (20 mL), suspended in fresh heptane (60 mL), and stirred at room temperature overnight. Filtration provided a filter cake, which was washed with heptane (20 mL) to afford additional **S11** as a colorless solid (12.6 g). Combined yield: 71.1 g, 186 mmol, 77%. ^{1}H NMR (400 MHz, chloroform-*d*) δ 8.72 (br d, *J* = 2.3 Hz, 1H), 8.19 (dd, *J* = 8.7, 2.5 Hz, 1H), 7.76 (dd, *J* = 7.7, 1.4 Hz, 1H), 7.64 (dd, *J* = 7.7, 1.3 Hz, 1H), 7.53 (ddd, *J* = 7.6, 7.5, 1.4 Hz, 1H), 7.45 (ddd, *J* = 7.5, 7.5, 1.3 Hz, 1H), 7.31 (s, 1H), 6.81 (br d, *J* = 8.7 Hz, 1H), 4.18 (q, *J* = 7.2 Hz, 2H), 4.13 (d, *J* = 6.7 Hz, 2H), 2.19 – 2.04 (m, 1H), 1.09 (t, *J* = 7.2 Hz, 3H), 1.04 (d, *J* = 6.7 Hz, 6H); HPLC: 11.082 min, 97% pure; LC/MS (*m/z*): 383.1 [M+H], 1.090 min.

Step 4. Synthesis of 2-{2-[6-(2-methylpropoxy)pyridin-3-yl]-1,3-thiazol-4-yl}benzoic acid (**PF-07238025**)

An aqueous solution of sodium hydroxide (3.0 M; 100 mL, 300 mmol) was added to a suspension of **S11** (58.5 g, 153 mmol) in ethanol (500 mL), and the reaction mixture was heated to 75 °C for 2 hours. After the reaction mixture had cooled, it was diluted with water (500 mL), and acidified to a pH of approximately 5 to 6 by addition of 1 M hydrochloric acid. The resulting mixture was allowed to stir for 30 minutes at room temperature, whereupon the precipitate was collected via filtration and washed with water to afford **PF-07238025** as a colorless solid. Yield: 51.3 g, 145 mmol, 95%. ^{1}H NMR (400 MHz, DMSO-d_6) δ 12.79 (s, 1H), 8.74 (d, *J* = 2.5 Hz, 1H), 8.21 (dd, *J* = 8.7, 2.5 Hz, 1H), 7.91 (s, 1H), 7.75 (br d, *J* = 7.7 Hz, 1H), 7.62 (br d, *J* = 7.6 Hz, 1H), 7.57 (ddd, *J* = 7.6, 7.5, 1.3 Hz, 1H), 7.48 (ddd, *J* = 7.5, 7.5, 1.0 Hz, 1H), 6.97 (d, *J* = 8.7 Hz, 1H), 4.12 (d, *J* = 6.7 Hz, 2H), 2.14 – 1.99 (m, 1H), 0.99 (d, *J* = 6.7 Hz, 6H). ^{13}C NMR (101 MHz, DMSO-d_6) δ

170.73, 164.99, 163.50, 154.93, 145.43, 137.45, 133.16, 130.76, 129.60, 128.86, 128.65, 123.59, 116.52, 111.62, 72.59, 27.91, 19.51; HRMS (m/z): $[M+H]^+$ calcd. for $C19H19N2O3S^+$, 355.1111; found, 355.1111; 2.39 min; 98%; HPLC: 9.149 min / 100% pure.

Synthesis of 2-(4-{6-[(1-ethylcyclopropyl)methoxy]pyridin-3-yl}-1,3-thiazol-2-yl)benzoic acid (**PF-07247685**)

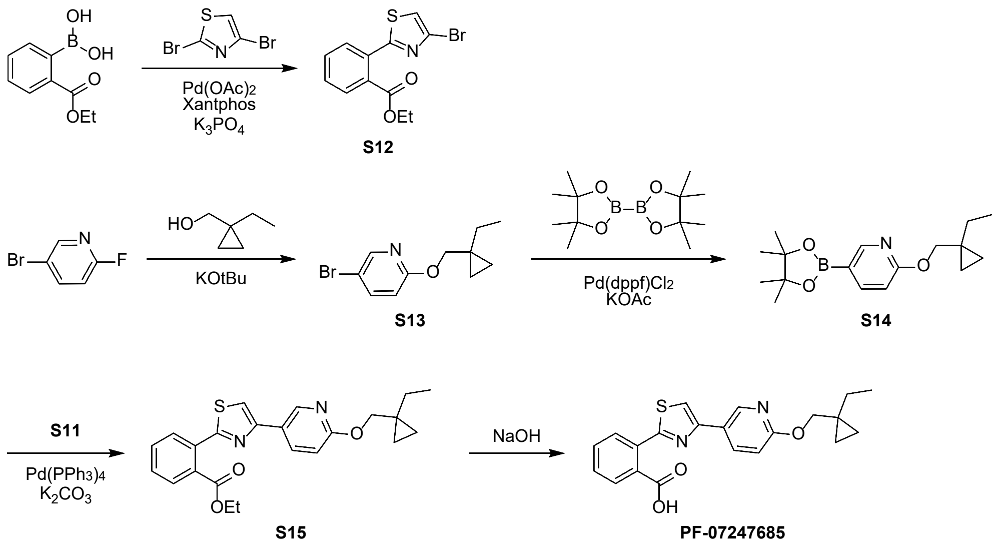

Step 1. Synthesis of ethyl 2-(4-bromo-1,3-thiazol-2-yl)benzoate (**S12**)

A reaction vessel containing a mixture of 2,4-dibromo-1,3-thiazole (10.0 g, 41.2 mmol), [2-(ethoxycarbonyl)phenyl]boronic acid (8.78 g, 45.3 mmol), palladium(II) acetate (462 mg, 2.06 mmol), 4,5-bis(diphenylphosphino)-9,9-dimethylxanthene (Xantphos; 1.19 g, 2.06 mmol), potassium phosphate monohydrate (28.4 g, 123 mmol), and tetrahydrofuran (206 mL) was evacuated and charged with nitrogen; this evacuation cycle was carried out a total of 3 times. After the reaction mixture had been heated at 70 °C for 3 days, it was cooled and partitioned between water and ethyl acetate. The aqueous layer was extracted twice with ethyl acetate, and the combined organic layers were washed with saturated aqueous sodium chloride solution, dried over sodium sulfate, filtered, and concentrated in vacuo. Silica gel chromatography was carried out twice: #1 (Eluent: dichloromethane); #2 (Gradient: 0% to 20% ethyl acetate in heptane). The purified material was triturated in heptane, while it still contained a small amount of ethyl acetate from concentration of the column fractions; this afforded **S12** as a yellow solid. Yield: 8.67 g, 27.8 mmol, 67%. LCMS 0.89 min / *m/z* 313.9 (bromine isotope pattern observed) $[M+H]^+$. ^1H NMR (400 MHz, chloroform-*d*) δ 7.88 – 7.79 (m, 1H), 7.68 – 7.60 (m, 1H), 7.63 – 7.51 (m, 2H), 7.33 (s, 1H), 4.29 (q, *J* = 7.2 Hz, 2H), 1.24 (t, *J* = 7.1 Hz, 3H); ^{13}C NMR (101 MHz, DMSO-d_6) δ 168.09, 167.16, 132.28, 131.93, 131.25, 131.00, 130.12, 129.65, 124.83, 120.29, 61.53, 14.16; HRMS (m/z): $[M+H]^+$, Br isotopic pattern, calcd. for $C12H11BrNO2S^+$, 355.9688; found, 355.9688; 2.26 min; 99%; HPLC: 8.780 min / 97% pure.

Step 2. Synthesis of 5-bromo-2-[(1-ethylcyclopropyl)methoxy]pyridine (**S13**)

Potassium *tert*-butoxide (38.3 g, 341 mmol) was added to a 0 °C solution of (1-ethylcyclopropyl)methanol (25.0 g, 250 mmol) in tetrahydrofuran (630 mL). The reaction mixture

was stirred for 15 minutes, whereupon a solution of 5-bromo-2-fluoropyridine (40.0 g, 227 mmol) in tetrahydrofuran (60 mL) was added as a single portion. The ice bath was then removed, and the reaction mixture was allowed to stir for 6 hours. After slow addition of water, the resulting mixture was extracted three times with ethyl acetate, and the combined organic layers were washed with saturated aqueous sodium chloride solution, dried over sodium sulfate, filtered, and concentrated in vacuo. Chromatography on silica gel (Gradient: 0% to 10% ethyl acetate in heptane) afforded **S13** as a colorless oil. Yield: 56.1 g, 219 mmol, 96%. ^{1}H NMR (400 MHz, chloroform-*d*) δ 7.92 (br d, *J* = 2.6 Hz, 1H), 7.40 (dd, *J* = 8.8, 2.6 Hz, 1H), 6.45 (dd, *J* = 8.8 Hz, 1H), 3.86 (s, 2H), 1.25 (q, *J* = 7.4 Hz, 2H), 0.72 (t, *J* = 7.5 Hz, 3H), 0.28 (d, *J* = 2.1 Hz, 2H), 0.26 – 0.16 (m, 2H); ^{13}C NMR (101 MHz, DMSO-d_6) δ 163.03, 147.51, 141.94, 113.33, 111.60, 71.38, 27.11, 20.83, 11.06, 10.36; HRMS (m/z): [M+H]$^+$, Br isotopic pattern, calcd. for C11H15BrNOS$^+$, 256.0332; found, 256.0333; 2.74 min; 96%; HPLC: 10.631 min / 96% pure.

Step 3. Synthesis of 2-[(1-ethylcyclopropyl)methoxy]-5-(4,4,5,5-tetramethyl-1,3,2-dioxaborolan-2-yl)pyridine (**S14**)

A reaction vessel containing a mixture of **S13** (56.1 g, 219 mmol), 4,4,4',4',5,5,5',5'-octamethyl-2,2'-bi-1,3,2-dioxaborolane (66.7 g, 263 mmol), [1,1'-bis(diphenylphosphino)ferrocene]dichloropalladium(II) (16.0 g, 21.9 mmol), and potassium acetate (KOAc; 64.5 g, 657 mmol) in toluene (706 mL) was evacuated and charged with nitrogen. This evacuation cycle was repeated twice, whereupon the reaction mixture was heated at 100 °C overnight. It was then filtered; the filtrate was washed with toluene and concentrated under reduced pressure, providing **S14** as a black gum. This material was progressed directly to the following step.

Step 4. Synthesis of ethyl 2-(4-{6-[(1-ethylcyclopropyl)methoxy]pyridin-3-yl}-1,3-thiazol-2-yl)benzoate (**S15**)

A reaction vessel containing a mixture of **S12** (57.0 g, 183 mmol), **S14** (from the previous step; ≤219 mmol), potassium carbonate (75.7 g, 548 mmol), and tetrakis(triphenylphosphine)palladium(0) (21.1 g, 18.3 mmol) in 1,4-dioxane (550.0 mL) and water (220 mL) was evacuated and charged with nitrogen. This evacuation cycle was repeated twice, whereupon the reaction mixture was heated to 90 °C for 2 hours. It was then cooled and partitioned between saturated aqueous sodium bicarbonate solution and ethyl acetate. After the aqueous layer had been extracted twice with ethyl acetate, the combined organic layers were washed with saturated aqueous sodium chloride solution, dried over sodium sulfate, filtered, and concentrated in vacuo. Purification was carried out first using silica gel chromatography (Gradient: 10% to 20% ethyl acetate in heptane), and then via a second chromatography on silica gel (Eluent: 10% dichloromethane, followed by 20% methanol in dichloromethane) to yield **S15**. Yield: 42.8 g, 105 mmol, 57%. ^{1}H NMR (400 MHz, chloroform-*d*) δ 8.70 (dd, *J* = 2.5, 0.8 Hz, 1H), 8.14 (dd, *J* = 8.6, 2.5 Hz, 1H), 7.83 – 7.76 (m, 1H), 7.75 – 7.69 (m, 1H), 7.63 – 7.51 (m, 2H), 7.49 (s, 1H), 6.85 (dd, *J* = 8.7, 0.8 Hz, 1H), 4.25 (d, *J* = 7.2 Hz, 1H), 4.21 (d, *J* = 13.1 Hz, 3H), 1.59 (s, 3H), 1.53 (t, *J* = 7.4 Hz, 2H), 1.29 (s, 3H), 1.12 (t, *J* = 7.1 Hz, 3H), 1.00 (t, *J* = 7.4 Hz, 3H), 0.90 (t, *J* = 6.9 Hz, 2H), 0.56 (t, *J* = 3.0 Hz, 2H), 0.49 – 0.42 (m, 2H). LC/MS (1.08 min / calc'd. 408.52, found 409.3 (M+H)).

Step 5. Synthesis of 2-(4-{6-[(1-ethylcyclopropyl)methoxy]pyridin-3-yl}-1,3-thiazol-2-yl)benzoic acid (**PF-07247685**)

To a solution of **S15** (42.8 g, 105 mmol) in ethanol (1.1 L) was added an aqueous solution of sodium hydroxide (4.0 M; 131 mL, 524 mmol). The reaction mixture was heated at 80 °C for 2 hours, whereupon it was concentrated in vacuo to approximately one-half of the original volume and then added to a mixture of 1 M hydrochloric acid and ethyl acetate. The aqueous layer was extracted twice with ethyl acetate, and the combined organic layers were washed sequentially with water and with saturated aqueous sodium chloride solution, dried over sodium sulfate, filtered, and concentrated under reduced pressure. The residue was dissolved in heptane containing a small amount of ethyl acetate, and a small quantity of inorganic material was removed via filtration. The filtrate was diluted with ethyl acetate, washed twice with water and once with saturated aqueous sodium chloride solution, dried over sodium sulfate, filtered, and concentrated in vacuo. The residue was dissolved in a small amount of ethyl acetate; upon swirling of the flask, a solid began to form. heptane was added until the mixture became cloudy, and swirling was continued while additional material precipitated. When it appeared that solid was no longer forming, heptane was added, and the mixture was stirred for 2 hours. Filtration, followed by washing of the filter cake with heptane, provided **PF-07247685** as a white solid. Yield: 34.9 g, 91.7 mmol, 87%. ^{1}H NMR (400 MHz, DMSO-d_6) δ 13.05 (s, 1H), 8.77 (dd, J = 2.5, 0.7 Hz, 1H), 8.25 (dd, J = 8.6, 2.5 Hz, 1H), 8.18 (s, 1H), 7.82 – 7.76 (m, 1H), 7.72 – 7.56 (m, 3H), 6.94 (dd, J = 8.6, 0.7 Hz, 1H), 4.17 (s, 2H), 1.46 (q, J = 7.4 Hz, 2H), 0.93 (t, J = 7.4 Hz, 3H), 0.56 – 0.46 (m, 2H), 0.48 – 0.38 (m, 2H). ^{13}C NMR (101 MHz, DMSO-d_6) δ 170.13, 166.04, 163.81, 152.54, 145.20, 137.35, 133.84, 131.59, 131.11, 130.40, 129.97, 129.14, 124.17, 115.06, 111.17, 71.11, 27.18, 20.99, 11.16, 10.42.; HRMS (m/z): $[M+H]^+$ calcd. for $C21H21N2O3S^+$, 381.1267; found, 381.1267; 2.44 min; 100%; HPLC: 9.024 min / 100% pure.

X-ray Crystal Structure Methods:

Protein Purification and crystallization: Based on previously published BDK purification strategies[2, 3] an MBP-TEV-humanBDK-His construct was co-expressed with GroEL/ES (pGro7) in E. coli BL21 (DE3) cells. Harvested cells were resuspended in 100 mM potassium phosphate, 1 mM TCEP, 500 mM KCl, 0.1 mM EDTA, 10% glycerol, 1% Tween-20, 0.25% Triton X-100, pH 7.5 supplemented with Roche EDTA-free protease inhibitor tablets and benzonase. After lysis by Microfluidizer (Microfluidics), cellular debris was pelleted by centrifugation at 40,000 xg. The supernatant was applied to a 14 mL column of Amylose Resin High Flow (New England Biolabs), washed with 50 mM HEPES, 1 mM TCEP, 2 mM $MgCl_2$, 250 mM KCl, 300 mM arginine, 10% glycerol, pH 7.5 and eluted with buffer containing 10 mM maltose. Due to phosphorylation of the protein at the TEV cleavage site, the pooled protein was dephosphorylated with lambda phosphatase (NEB) while simultaneously removing the MBP by TEV protease. Cleaved, dephosphorylated BDK was applied to a Superdex 200 column (Cytiva Life Sciences) equilibrated in 50 mM HEPES, 1 mM TCEP, 2 mM $MgCl_2$, 1 M NaCl, 250 mM KCl, 300 mM arginine, 10% glycerol, pH 7.5. Fractions containing BDK were pooled and concentrated to at least 30 mg/mL. Crystals of dephosphorylated BDK diffracted poorly. To restore phosphorylation levels closer to pre-dephosphorylation levels, two cycles of autophosphorylation were performed. For each cycle, human BDK protein (25 mg/mL) was incubated overnight with 25 mM ATP and 65 mM $MgCl_2$, followed by another round of SEC to remove nucleotide. Autophosphorylation was verified by mass spectrometry.

Phosphorylated human BDK (20 mg/mL) was incubated with 25 mM ADP and 1 mM ligand and crystals were grown by sitting drop vapor diffusion. For the thiophene PF-07208254, crystals were grown with a well solution of 0.1 M MES pH 5.4, 0.2 M magnesium chloride, and 10% PEG-3350. Crystals were cryoprotected by gradually increasing the glycerol concentration to 25% before flash freezing in liquid nitrogen. For the thiazoles **S3** and PF-07247685, crystals were grown with a well solution of 0.2 M ammonium sulfate and 17-22% PEG-3350 and cryoprotected with 20% glycerol.

Data collection, structure determinations and refinements:

Crystal data sets were collected at beamline 17-ID at IMCA CAT (Advanced Photon Source, Argonne National Laboratory, Chicago). The wavelength used was 1 Å. Crystals were kept at 100 K using cryo stream. Data were processed using the program autoPROC[44]. Compared to the reported rat BDK complex crystals, our human BDK complex crystals diffracted weaker and showed strong anisotropical diffractions. Data sets were scaled and merged using anisotropic resolution cut off, and both spherical and ellipsoidal data completeness were reported.

The initial human BDK structure complex with PF-07208254 was determined using published rat BDK/BT2 complex structure (PDB ID, 4E01). Structure refinements were carried out using the program Buster[55]. Protein manual building was carried out using program Coot[66] and automatic ligand fitting was done using the program Rhofit[77]. There is one human BDK/PF-07208254 complex in the crystallographic asymmetric unit, and two human BDK/**S3** and BDK/PF-07247685 complexes in the crystallographic asymmetric unit, respectively. In the latter two crystals, complex B is significantly more disordered with weaker electron densities for both the protein and ligands. The percentages of residues in Ramachandran plot favored regions are 96.5%, 95.5%, 95.8% respectively, and in unfavored regions are 0.32%, 0.32% and 0.16% respectively.

Computational Methods:

Structure Preparation. Structure preparation was performed using Protein Preparation Wizard defaults in Schrodinger 2021-2[8-128-12] unless otherwise stated. X-ray co-crystal structures of rat BCKDK (rBDK) with BT2, PF-07208254, PF-07238025 and PF-07247685 in the allosteric binding site and apo were internally generated (2.1 – 2.9 Å resolution) and used as initial coordinates for MD simulations (human structures were not available at the time). Rat and human BDK protein sequences are 95.6% identical and the 3-dimensional structures are highly similar between the two species (RMSD = 0.63 Å over all heavy atoms). Most of the species differences occur in the N-terminus, which is unresolved in the X-ray crystal structures. There are no residue differences between rat and human within 9 Å of BT2, PF-07208254, PF-07238025 or PF-07247685; therefore, it is expected that the conclusions drawn from analysis of MD results based on the rat structures will translate to human. Two loops were not resolved in the structures. Loop 1 residues R50 – Q52 (numbering based on the signal peptide processed, mature protein) were built using Prime[12-1412-14]. The ATP lid (loop 12, residues A306 – G335) was not modeled due to its size and was capped at residues T305 and F336. ADP, two crystallographic ions, Mg^{2+} and K^+, and one crystallographic water molecule that forms a hydrogen bond network between ADP, D285 sidechain and G289 backbone were present in the ATP binding site for all simulations. Consistent zero-order bonds to Mg^{2+} and K^+ were employed. Two additional crystallographic waters were retained in the structures of PF-07238025 and PF-07247685, one of which forms a hydrogen bond network from the carboxylic acid of the ligand to the L164 backbone, Y346 sidechain, and water #2, and the other of which forms a hydrogen bond network to the thiazole N of the ligand,

the R171 sidechain, and water #1. Protonation states were optimized using PROPKA at pH 7.0 and checked for consistency across all systems.

System Preparation, MD Simulations, Analysis. Desmond System Builder in Schrodinger 2021-2[15-1715-17] was used to prepare the systems for simulation. The OPLS4 force field[1818] was used following customization using the Force Field Builder panel for missing ligand torsion parameters along with the TIP3P water model[1919]. An orthorhombic periodic box was set up with 12 Å of water on all sides of the system and neutralizing ions added. Default Desmond minimization and equilibration protocols were employed. Recent work has suggested that analysis of multiple MD replicas leads to more reliable conclusions than those drawn based on a single long simulation[2020-23]; therefore following equilibration, six independent NPT production simulations of 100 ns each were run for all five systems using Desmond, starting with the same equilibrated structure and using new initial randomized velocities. The independent trajectories were combined for subsequent analysis, giving a total production time of 600 ns for each system. Schrodinger 2021-2 software was used to perform RMSD, RMSF, essential dynamics, CM, and hydrogen bond analyses. BDK lipoyl-binding pocket residues were identified based on overlay with the asymmetric PDK3-L2 complex (PDB ID: 1Y8P)[2124] and selection of residues within 6 Å of the L2 group.

Supporting computational details and analysis.

All MD trajectories stabilized by 50 ns based on the backbone root-mean-square deviation (RMSD) from the initial structures (Supplementary Figure 9A). The dynamics of loop 1 and the lipoyl-binding pocket residues upon inhibitor binding seem to play a role in destabilization or stabilization of BDK on the BCKDH complex as described in the main text. As detailed in Tso et al [2325] there is a network of interactions connecting helices α4 and α5 to the lipoyl-binding pocket, and the inhibitor (*S*)-CPP perturbs the lipoyl-binding pocket residues. In support of this, the MD simulations suggest there is an overall increase in dynamic flexibility of the BDK lipoyl-binding pocket when bound to destabilizers BT2 and PF-07208254, and an overall decrease in dynamic flexibility of the lipoyl-binding pocket when bound to stabilizers PF-07238025 and PF-07247685. This observation can be explained by an increase in the overall number of hydrogen bonds formed over the course of the MD simulations when BDK is bound to stabilizers PF-07238025 and PF-07247685, 73 and 298 on average for the lipoyl-binding pocket and overall, respectively, compared to when BDK is bound to destabilizers BT2 and PF-07208254, 71 and 295 on average (Supplementary Figure 10C-D).

The width of the active-site cleft seems to play an important role in the ability of BDK to phosphorylate E1 as described in the main text. When destabilizers BT2 or PF-07208254 are bound to BDK, the width of the active-site cleft is narrowed as measured by $CM_{\alpha5\text{-}\alpha8}$, preventing E1 from positioning itself for phosphorylation (Supplementary Figure 10A-B). In the apo structure, Y99, L129, H132, R167, and R171 form an intricate hydrogen bond (HB) network: Y99 forms a HB with R167 via sidechain interactions, R167 sidechain forms a HB with the H132 backbone, and R171 sidechain forms a HB with the backbone of L129 (Supplementary Figure 10E-F). Upon binding of a destabilizer, BT2 or PF-07208254, this HB network is rearranged, as previously described by Tso et al.[25, 26] The acid moiety of the inhibitor binds in between Y99 and R167, forming HB interactions with the sidechains and forcing the R167 sidechain toward the active-site cleft. The HB between the R167 sidechain and H132 backbone is maintained, which pulls the loop in between helices α4 and α5 toward the active-site cleft, and places the H132 sidechain into position to interact with the acid moiety of the inhibitor (Supplementary Figure 10E). The

importance of H132 was previously shown in the study of a p.His162Gln variant by Maguolo et al.,[2527] and the current results further highlight the importance of this residue and its interactions. The R171 sidechain maintains its HB with the L129 backbone and forms an additional HB interaction with the acid of the ligand. These changes in interactions induced by ligand binding cause a subtle movement in the positions of helix α5 and α3, leading to a narrowing of the active-site cleft.

In contrast, when stabilizers PF-07238025 or PF-07247685 are bound to BDK, the active-site cleft widens as measured by $CM_{\alpha5\text{-}\alpha8}$. Upon binding of a stabilizer, PF-07238025 or PF-07247685, the R167 sidechain flips in order to make 2 HBs to the acid moiety of the inhibitor (Supplementary Figure 10F). This causes H132 to adjust to maintain the HBs between its backbone and the R167 sidechain, pulling the loop between helices α4 and α5 away from the active-site cleft. A HB can still be maintained between Y99 and R167 sidechains with the inhibitor bound as in the apo structure. The sidechain of R171 is forced to change conformation upon binding of a stabilizer and forms a HB with the sidechain of Y346 in addition to participating in a water-mediated HB network with the acid and thiazole nitrogen of the inhibitor. The terminal phenyl of the inhibitor, which is positioned in the active-site cleft and blocks E1 from binding, interacts with Y241 through van der Waals (VDW) interactions and pushes the sidechain slightly away, causing the cleft to widen further.

Western Blot antibodies: The following antibodies and concentrations were used for Western blotting. pBCKDH phospho S292 (Bethyl A304-672A 1:1000), total BCKDH (Yenzym 037 1:1000), BDK (abcam ab128935 1:1000), GAPDH (CST 14C10 1:10000), α-tubulin (CST 2144 1:10,000). Secondary antibodies were anti-rabbit IgG HRP-Conjugated-(CST 7074P2 1:10000) Anti-mouse IgG (CST HRP-linked Antibody #7076).

In vivo compound measurements: Compounds were quantitatively measured in plasma and tissue samples using liquid chromatography–tandem mass spectrometry (LC-MS/MS). All standards were made in blank mouse plasma and were typically prepared at 1-50,000 ng/mL. Tissues were homogenized using a 1:4 dilution in 60:40 isopropanol:water and 2 hours of shaking in the bead beater using zirconia beads. For tissue analysis, a mixed matrix approach was used where an equal volume of blank tissue homogenate was added to plasma standards, and blank plasma was added to tissue samples. Samples were prepared by protein precipitation using acetonitrile containing 150 ng/mL indomethacin, 25 ng/mL diclofenac and 5 ng/mL tolbutamide as internal standards, vortexed and centrifuged, then transferred and diluted with water for LC-MS/MS analysis. Chromatography was performed on a Waters Acquity iClass UPLC System (Milford, MA). Separation was achieved with an Acquity UPLC HSS T3 column (2.1x50 mm, 1.8 µm), and a gradient of 0.1% formic acid in water (Mobile Phase A) and 0.1% formic acid in acetonitrile (Mobile Phase B) at a flow rate of 0.600 mL/min. The gradient increased from 5%B to 95%B over 2.0 minutes and the entire run time was 3 minutes. Data was collected on an AB Sciex API6500 mass spectrometer (Foster City, CA, USA) using Turbo IonSpray™ electrospray ionization (ESI) and multiple reaction monitoring (MRM) mode. Typical source conditions (heated capillary temperature, gas1, gas2, and curtain gas) were set at 500C, 40, 60 and 20, respectively. Data acquisition and quantitative processing was carried out with Analyst software version 1.6.2 (Applied Biosystems/MDS Sciex, Canada).

MRM transitions were optimized for each compound. BT2 (Negative Ion Mode): Q1 245.0; Q3 201.0; DP -60; CE -15; IS=Diclofenac. PF-07208254 (Negative Ion Mode): Q1 235.0; Q3 191.0; DP -65; CE -18; IS=Tolbutamide. PF-07238025 (Positive Ion Mode): Q1 355.1; Q3 281.1; DP 60; CE 34; IS=Indomethacin. PF-07247685 (Positive Ion Mode): Q1 381.2; Q3 281.1; DP 80; CE 45; IS=Indomethacin. IS Transitions: Diclofenac (Negative Ion Mode): Q1 294.1; Q3 250.0; DP -40; CE -15. Tolbutamide (Negative Ion Mode): Q1 269.0; Q3 170.0; DP -60; CE -30. Indomethacin (Positive Ion Mode): Q1 358.3; Q3 139.2; DP 46; CE 26.

Plasma BCAA/BCKA quantification: Plasma BCAA and BCKA were analyzed using a standardized method at Pfizer or at Q^2 solutions, Ithaca, NY. Two methods were used for BCAA/BCKA quantitation. *Pfizer (Groton, CT):* A surrogate analyte approach was utilized with $^{13}C_5\,D_8\,^{15}N$ valine, D_{10} leucine, $^{13}C_6\,D_{10}\,^{15}N$ isoleucine, $^{13}C_4\,D_4$ ketovaline, D_7 ketoleucine and $^{13}C_6$ ketoisoleucine as surrogate analytes and $^{13}C_5$ valine, D_7 leucine, $^{13}C_6$ isoleucine, $^{13}C_5$ ketovaline and D_3 ketoleucine as internal standards (ketoleucine and ketoisoleucine shared the same IS). Surrogate standard curves were typically prepared at 10 – 50,000 ng/mL in control mouse plasma. Samples and standards were subjected to protein precipitation with methanol containing internal standards, vortexed and centrifuged to obtain supernatant. An aliquot of the supernatant was transferred to a new 96-well block, dried down under nitrogen, and reconstituted in water containing 0.1% acetic acid for injection. Chromatography was performed on a Shimadzu Nexera UPLC System (Kyoto, Japan). Separation of structural isomers was achieved with an Acquity UPLC HSS T3 column (2.1x100 mm, 1.8 µm, Waters Corporation, Milford MA) using 0.1% acetic acid in water (Mobile Phase A) and methanol (Mobile Phase B) at a flow rate of 0.400 mL/min. The gradient increased from 3%B to 85%B over 6.1 minutes and the entire run time was 10 minutes. Data was collected on an AB Sciex Triple TOF 6600 mass spectrometer (Foster City, CA, USA) using negative ion electrospray ionization (ESI) and TOF MS with a mass range of 60-1060. Extracted ion chromatograms were integrated at a mass tolerance of 10 ppm. Surrogate standard calibration curves were obtained by fitting the normalized intensities of the surrogate standards vs. their concentrations using a linear fit with $1/x^2$ weighting factor. The endogenous concentrations were obtained by converting normalized intensities to concentrations according to the corresponding surrogate standard curve fitting equations. Data acquisition was performed with Analyst software version 1.6.2, peak integrations were performed in MultiQuant and either Multiquant or Watson LIMS 7.5 was used for regression and quantitation.

Tissue BCAA/BCKA quantitation: Mouse muscle tissue BCAA/BCKA were analyzed using a standardized method at Q^2 solutions, Ithaca, NY. Briefly, mouse muscle tissue was pounded to powder under liquid N_2 and homogenized and sonicated in working internal standard solution (WISS) containing Leucine D_{10}, Isoleucine D_{10}, Valine D_8, Ketoleucine D_3, Ketoisoleucine D_8, Ketovaline $^{13}C_4\,D_4$ in 4:1 methanol/water at a ratio of 50 mg tissue/1 mL WISS. Samples were spun, and the supernatant was collected and transferred to a deep well injection plate followed by drying under nitrogen at 40 °C. Residue was resuspended in 50 µL water containing 0.2% acetic acid. Samples were run on a Dionex Ultimate 3000 with a Waters HSS T3 1.8 µm, 2.1 x 100 mm column. The mobile phase was A:1000:2 water/acetic acid and B: methanol. Gradient separation began at 1.8% B for 2 minutes then increased to 5.5% B over 0.5 minutes, then to 6.5% B over 2.75 minutes, then to 100% B over 1.75 minutes and held for 1 minute followed by a return to 1.8%B and held for 1 minute. Data was acquired on a Thermo Q-Exactive mass spectrometer within a scan range of 100 – 160 m/z. A surrogate analyte approach was utilized for quantitation with $^{13}C_5\,^{15}N$ valine, $^{13}C_6$ leucine, $^{13}C_6$ isoleucine, $^{13}C_5$ ketovaline, $^{13}C_6$ ketoleucine and $^{13}C_6$ ketoisoleucine as surrogate analytes. Surrogate standard curves were typically prepared at 25 – 4000 nM for amino acids and 0.8 – 64 nM for keto acids in pooled mouse tissue extract. The mono-isotopic mass was monitored for all analytes and standards except for leucine, isoleucine and valine, for which the M+1 isotopologue was measured and a correction factor

based on the natural isotope abundance was applied. Surrogate standard calibration curves were obtained by fitting the normalized intensities of the surrogate standards vs. their concentrations using a linear fit. The endogenous concentrations were obtained by converting normalized intensities to concentrations according to the corresponding surrogate standard curve fitting equations.

Transverse Aortic Constriction: Two rounds of equivalent surgeries were performed. The afternoon prior to surgery a subcutaneous dose of SR Meloxicam was administered to ensure adequate pre-operative analgesia coverage. Meloxicam was slowly released over 72 hours. All surgical instruments were sterilized with vaporized hydrogen peroxide. The surgeon and all surgical personnel donned a bonnet, masks, gowns, and gloves. Instruments were sterilized between each animal using a hot bead sterilizer and sterile tip technique. On the day of surgery, anesthesia was induced by administering an anesthetic cocktail IP (Ketamine 25 mg/mL @ 100 mg/kg + xylazine 2 mg/mL @ 8 mg/kg). Once at an acceptable plane of anesthesia, the animal was prepped for surgery. A pre-operative dose of LRS was administered subcutaneously at 20 mL/kg. Nails were clipped, and fur was shaved from the chin to the xyphoid process. The skin was prepped with 2 consecutive scrubs of Chloraprep swabs. The animal was intubated using polyethylene 60 tubing and placed on a ventilator. End tidal volume was set at 250 µL/min, and the respiration was set at 140 breaths per minute. A final scrub with a Chloraprep swab was applied. Isoflurane was administered at rate of 0.5 to 2% throughout the procedure.

A toe pinch was performed to ensure an adequate plane of anesthesia for surgery. A midline skin incision was made above the 1st and 2nd rib, approximately 0.5-1 cm in length. A local analgesic block (Bupivacaine @ 1 mg/kg + Buprenorphine @ 0.003 mg/kg) was administered to the intercoastal muscles along the 1st and 2nd ribs. Using blunt dissection, a 3 mm muscle incision was made between the 1st and 2nd rib. The thymus was gently manipulated and placed on either side of the incision to expose the aortic arch beneath. A retractor was placed on each side of the incision to secure the thymus and create a clear surgical window of the aortic arch. The tissue of the aortic arch was gently isolated, between the brachiocephalic artery and the left common artery. A 6-0 Silk suture was passed under the aortic arch, and a 4 mm stainless steel pin (0.38 mm diameter) was placed across the aorta. The suture was tied around the needle and secured using a surgeon's knot. Immediately, the stainless steel pin was removed from under the suture and the suture remained in place to produce a defined constriction of the vessel. For sham surgery, the aortic arch was isolated, and a suture was passed under the aortic arch, no ligation was made, and the suture was removed. The retractor was carefully removed, and the ribs were closed with 2 simple interrupted sutures using 6-0 Prolene. To prevent atelectasis, before completely closing the muscle incision, positive pressure was applied to the exhaust tubing of the ventilator to assist in evacuating any excess air from the chest cavity. A small amount of saline was applied to the muscle tissue, and the skin was closed with 5-0 Prolene using 2 single external horizontal mattress pattern sutures. The mouse was slowly weaned off the ventilator. An intraperitoneal dose of Antisedan (0.5 mg/mL @ 1 mg/kg) was administered to reverse the effects of xylazine. When the animal was removed from the ventilator, they were placed in a warm oxygen chamber until they were ambulatory. Once ambulatory the mice were returned to a clean warm cage and a subcutaneous dose of diluted buprenorphine was administered. During day 0 PM post-operative assessments, a subcutaneous dose of LRS (20 mL/kg) and diluted buprenorphine was administered. Another dose of subcutaneous LRS and diluted buprenorphine was administered during day 1 AM post-operative assessments. During day 1 PM post-operative assessments through day 2 PM assessments, diluted buprenorphine was administered subcutaneously. Animals were given Nutra Gel support for 4 days post surgery. After the 72-hour post-op period animals were monitored daily until post-op day 7. Food consumption was monitored throughout the study to ensure similar food consumption across groups. Three animals

that did not consume at least 1.5 g/day compound chow for 3 consecutive days and consequently had very low plasma levels of PF-07208254 for a sustained time period were excluded from the study. Animals that lost 20% of body weight or greater from the start of surgery were humanely euthanized. At euthanasia, hearts were excised, excess blood within the chamber was drained, hearts were weighed, the atria were removed, and the ventricles were immediately snap frozen in liquid nitrogen. Lungs were blotted briefly on a napkin and weighed.

Echocardiography: Ultrasounds were acquired in a blinded fashion at 4 weeks post-surgery on the Vevo3100 using the MX550D probe. Prior to the imaging studies, animals were anesthetized with 2% isoflurane in an induction chamber. Once asleep, animals were placed onto a heated platform set to 34 °C to maintain body temperature. Heart rates were monitored and maintained above 400 bpm for all measurements. Electrode gel was applied to the four paws and taped to the ECG electrodes on the heated platform. Hair was removed from the chest with Nair, and isoflurane was set to 0.5-1.0% for systolic function acquisition. Warm echo gel was placed on the shaved chest, and the MX550D probe was used for recordings. For recording of the pasternal long axis (LV long axis view), the "notch" of the MX550D transducer was placed towards the head of the animal and rotated approximately 30-45 degrees clockwise. When heart structures were visualized clearly, a B-mode image of the heart was recorded. The MX550D probe was turned 90 degrees clockwise to the left side of the mouse to obtain a pasternal short axis view (LV short axis view). Once the ventricular structures were clear, the M-mode cursor was set in the middle of the ventricle and an M-mode image of the ventricle was recorded. After scanning was finished, residual echo gel was removed, and the mouse was returned to the cage for recovery. Echocardiography images were analyzed using Vevo Lab 3.20 software. At least three beats were measured, and the mean was calculated. Images were analyzed blindly after acquisition.

Hek293 Cell experiments: Human embryonic kidney (HEK293) cells were purchased from American Type Culture Collection (ATCC) (CRL-1573™) and were cultured at 37 °C in Eagle's Minimal Essential Medium (EMEM) supplemented with 10% Hi-FBS (Gibco) and 100 units/mL Penicillin/Streptomycin (ThermoFisher Scientific). BT2 (100 and 300 µM), PF-07208254 (3, 10, 30 or 300 µM), PF-07238025 (0.2, 0.6, 2 or 6 µM), PF-07247685 (0.01, 0.03, 0.1 or 0.3 µM) or DMSO (0.01%) was added to HEK293 cells and incubated for 48 hours at 37 °C. Whole cell lysates were lysed in 200 µL of Cell Signaling lysis buffer containing HALT protease inhibitors (ThermoFischer Scientific) 48 hours post-treatment. Cells were lysed by sonication. Samples were then spun at 16,260 x *g* for 10 minutes, and the supernatant was placed in new tubes. Total protein quantification was calculated using Pierce BCA assay. All samples were boiled at 95 °C for 5 minutes with Invitrogen LDS buffer and Reducing Agent at a concentration of 2 mg/mL of total protein based on the BCA assay results. 20 µg of each sample was loaded into NuPage 4-12% Bis-Tris gels and samples were run at 160V for 2 hours using the BioRad system. The proteins were then transferred to a PVDF membrane using the iBlot2 system. Membranes were blocked in 5% milk in TBS plus 0.05% Tween 20. The membranes were exposed using the Amersham 800 imager, and Imagequant software and Microsoft Excel 365 was used to analyze densitometry of each band. Primary antibodies used are detailed above.

In vitro BDK FRET activity assay: Initially, 5 µL of 2X BDK enzyme (5 nM final) in assay buffer (20 mM Tris·HCl (pH 7.5), 100 mM KCl, 5 mM $MgCl_2$, 0.5 mM DTT, 0.02% (vol/vol) Tween-20, and 0.01% BSA) was added to the compound plates and pre-incubated for 15 min at room temperature. Final enzyme concentration was optimized to reflect the linear range of enzyme activity with less than 30% substrate conversion. This was followed by addition of 5 µL of 2X LBD-Tev-PhosphoPep+Lipoyl substrate/ATP mix in assay buffer to initiate the reaction. Plates were

covered with a lid and allowed to incubate at room temperature for 90 min. Final concentration of LBD-E1 substrate was 50 nM and final concentration of ATP was 15 µM. Following incubation, the enzyme reactions were stopped by adding 5 µL of 4X stop/primary antibody solution containing 100 mM (final) EDTA, 2 nM (final) anti-His Eu antibody, and 1:1000 anti-pE1 antibody in assay buffer. The plates were covered and incubated at room temperature for 30 min. Finally, 5 µL of 4X detection reagent consisting of 20 nM (final) anti-rabbit Ulight antibody and 1X LANCE detection buffer in dH_2O was then added. The plates were centrifuged for 30 seconds at 113 x *g*. After a 60 min incubation, the plates were read on an EnVision® MultiReader in TR-FRET mode (excitation 320 nm: emissions 665 nm). The raw data from the EnVision® MultiReader was expressed as a ratio and was analyzed using proprietary software (Activity Base). The percent effect at each concentration of compound was calculated relative to the values for the uninhibited control wells (100% DMSO) and fully inhibited control wells (600 µM radicicol) on each assay plate. IC_{50} values were determined from the percent effect data using a 4-parameter logistic dose response model (Activity Base).

Human skeletal muscle BDK activity assay: BDK activity and inhibition in human skeletal muscle cells was monitored by measuring BDK dependent phosphorylation of endogenous BCKDHA using a custom AlphaLISA SureFire Ultra assay system. Prior to the assay, the total BCKDHA antibody (Bethyl Laboratories - A303-790A) was biotinylated using the ChromaLink™ One-Shot Antibody Biotinylation Kit (Trilink Technologies B-9007-009K) and a phospho (S293) BCKDHA antibody (Bethyl Laboratories A304-672A) was CaptSure tagged using the Lightning-Link® CaptSure™ Conjugation Kit (TGR BioSciences 6300007). Human skeletal myocytes (Gibco A11440) were previously immortalized via the introduction of hTERT into the cells. Immortalized human skeletal muscle cells were plated at 15,000 cells/well and grown in skeletal muscle growth media containing the media supplement and chick embryo extract (Promocell C-23060 and C-23160, MP92850145) in 384-well CulturPlate plates. After overnight incubation, media was removed, and compound treatments were performed in growth media diluted 10X in PBS for 60 minutes. Cells were washed with PBS and lysed in 1X lysis buffer (Cell Signaling 9803) containing 2 nM biotinylated total BCKDHA Antibody and 1X protease/phosphatase inhibitor cocktail (Cell Signaling 5872). After an hour incubation at room temperature, a 1X immunoassay buffer (Perkin Elmer AL000F) mix containing 1:400 CaptSure tagged Phospho BCKDHA Antibody and 40 µg/µL Anti CaptSure acceptor beads (Perkin Elmer ALSU-ACAB) was added to the lysates and allowed to incubate for 60 minutes. The signal was then generated by the addition of streptavidin donor beads (Perkin Elmer ALSU-ASDB) at 40 µg/µL in 1X immunoassay buffer and incubating at room temperature for an additional 30 minutes. The phospho BCKDHA signal was developed using EnVision® multilabel reader (Perkin Elmer) using Alphascreen settings (excitation 680 nm, emission 570 nm). The fluorescence emission was used to calculate the % effect relative to the HPE (30 µM BT2) and ZPE (DMSO). IC_{50} curves were generated using ActivityBase software.

RNA Isolation: Frozen tissues were placed in Matrix D Lysis Tubes (2 mL) and homogenized in 1 mL of Qiazol and 200 mL of chloroform (Sigma). Samples were then spun for 10 min at 11,292 x *g* for phase separation, and the upper aqueous phase of each sample was mixed in a 1:1 ratio with 70% ethanol. The samples were then washed in Qiagen RNEasy spin columns and reagents according to the manufacturer's protocol. Total RNA was quantified using NanoDrop. qPCR was performed using the RNA to CT one step kit (Invitrogen) according to manufacturer's instructions and the Taqman Probes (Fisher) used are listed in Supplementary Table 5.

Supplementary Table 5. RT-PCR Probes

Gene	Catalog ID

Tnfa	Mm00443258_m1
Ccl2	Mm00441242_m1
Cd68	Mm03047343_m1
Ccr2	Mm99999051_gH
Col1a1	Mm00801666_g1
Col1a2	Mm00483888_m1
Bckdk	Mm00437777_m1
Ppia	Mm02342430_g1
Hprt	Mm03024075_m1
Gapdh	Mm99999915_g1
Itgam	Mm00434455_m1
Bckdha	Mm00476112_m1
Bckdhb	Mm01177077_m1
Bcat1	Mm00500289_m1
Bcat2	Mm00802192_m1
Dbt	Mm00501651_m1

Plasma analysis and ELISA: Whole blood samples were collected in K_2 EDTA tubes and placed on ice. Samples were then spun in a microcentrifuge at 4 °C for 10 min at 11,292 x *g*. Plasma was aliquoted into separate tubes, and ALPCO Ultrasensitive Insulin ELISA (80-INSMSU-E01, E10) was utilized according to manufacturers' instructions.

Liver triglyceride assessment: Frozen livers were pulverized on a cooled aluminum block in liquid nitrogen. Approximately 50 mg of pulverized tissue was added to a pre-weighed 2 mL lysing matrix D tube. After the tissue was added, these tubes were weighed again to determine the exact weight of the tissues. 1 mL of homogenization buffer, containing 10 mM TRIS pH 7.4, 0.9% NaCl and 0.2% Triton X100, was added to each sample. Samples were immediately homogenized using MP FastPrep 5G with the following parameters: (speed: 6, time: 40 seconds, cycles: 1). The samples were then vortexed for 20 seconds. and then transferred to 1.1 mL tubes and immediately analyzed on a Siemens Chemistry XPT clinical analyzer using Triglycerides_2 reagents.

Histopathology Evaluation: The left lateral lobe of the liver was fixed in 10% formalin, embedded in paraffin, and sectioned; 5 mm sections were mounted to positively charged slides for H&E staining. A board-certified veterinary pathologist who was familiar with the animal models graded H&E sections for hepatic lipid vacuolation (steatosis) and inflammation (inflammatory cell infiltrates) in a blinded fashion on a scale of 0-4.

Interaction proteome:

Lysis and digestion. Mouse hearts were lysed from mice that were treated with vehicle, 10 mg/kg PF-07247685 BID (described in Extended data Fig. 5), or 100 mg/kg BT2 QD for 18 days. BDK was immunoprecipitated from 5 mg of heart lysate with 15 µL BDK antibody (N=3/group) or 5 µL IgG (N=3 total). Immunoprecipitates were performed overnight, protein A magnetic beads were added, and Ips were washed 3x using lysis buffer. The beads were subsequently washed 3 additional times in PBS and resuspended in denaturing buffer (8 M urea and 0.1 M Tris, pH 8.5).

The proteins were reduced with dithiothreitol (DTT) 1 mM final concentration for 30 min at 55 °C and alkylated with iodoacetamide (55 mM final concentration) for 30 min at RT in the dark. Lys-C protease (Wako Chemicals, Richmond, VA, USA) was added at a ratio 1:100 to the total protein amount and incubated at room temperature for 2 h in a Thermomixer. Urea was diluted to 2 M with 50 mM Tris pH 8.5, and the samples were incubated with trypsin (1:50 w/w) (Pierce Biotechnology, USA) overnight at 37 °C at 41 x *g*.

Elution and desalting of peptides. The peptide mixtures were acidified with formic acid (FA) to 1% final concentration (pH <3) and centrifuged at 4000 x *g* for 5 min. Peptides were desalted in 2-plug C18 (Empore C18 extraction disks). The stage tips were sequentially conditioned with methanol (MeOH), 100% acetonitrile (ACN), 50% (vol/vol) ACN / 0.1% (vol/vol) FA, and 0.1% (vol/vol) FA. The samples were loaded onto the stage tips and centrifuged at 3000–3500 x *g* for 3 min at room temperature. After sample loading, the tips were washed twice with 0.1% FA. Peptides were eluted with 50% ACN/0.1% FA. The eluted peptides were dried in a Speed-Vac centrifuge, reconstituted in 5% ACN, 0.1% FA (vol/vol) and sonicated in a water bath sonicator for 5 min.

MS analyses. The peptides were loaded on a 50 cm column (Thermo Fisher ES903) and separated by reversed-phase chromatography using a gradient from 5% to 30% B over 2 h (Buffer A: 0.1% FA in HPLC grade water; Buffer B: 80% ACN, 0.1% FA) with a flow rate of 0.25 µL/min using an EASY-nLC 1200 system (Thermo Fisher Scientific). For interaction proteome MS data were acquired on a Q Exactive HF mass spectrometer (Thermo Scientific) using a data-dependent acquisition top 10 method, AGC target 3e6, maximum injection time of 32 msec, scan range of 375-1500 m/z and a resolution of 60K. MS/MS was performed at a resolution of 15K, AGC target 1e5, maximum injection time of 60 msec, isolation window 1.4 m/z. Dynamic exclusion was set to 20 seconds. Raw mass spectrometry data were processed using the MaxQuant software Version 1.6.10.43 (www.maxquant.org) with the Andromeda search engine integrated into MaxQuant environment. The MS/MS spectra were searched against the mouse UniProt sequence database without spliced isoforms. All MS/MS spectra were searched with the following MaxQuant parameters for peptide identification: acetyl (protein N-terminus) and methionine oxidation, were selected as variable modifications; cysteine carbamidomethylation was selected as fixed modification. A maximum of 2 missed cleavages were allowed. Peptide spectrum matches and proteins were automatically filtered to a 1% false discovery rate based on Andromeda score, peptide length, and individual peptide mass errors. Modified peptides required a minimum peptide length of at least six amino acids (AA).

Quantification. TMT reporter ion intensity values were quantified from MS2 scans using an integration tolerance of 20 ppm (Orbitrap) with the most confident centroid setting (Maxquant 1.6.10.43) for matching peptides. For interactome analysis, raw reporter ion abundance was used for further analysis. MSstatsTMT workflow starts from the peptide intensities reported in Maxquant's evidence.txt file. When a peptide and charge combination was measured multiple times in a sample, only the maximum intensity was kept. The log2 peptide intensities were median normalized assuming equal input loading of all channels. Peptide intensities were summarized to protein intensities using Tukey's median polish algorithm[2228]. MSstatsTMT builds protein-wise linear models based on these protein summaries, which was used for AP-MS proteomics analysis.

SPR binding assay:

The binding affinity and kinetics of binding were measured using Surface Plasmon Resonance based binding assay[29]. These experiments were carried out on Biacore B3000 (Cytiva Inc) and Sierra Sensors (now Bruker) MASS-1 instruments. There was no significant difference in results obtained on both these instruments. Bap-tagged BDK protein was captured on a Streptavidin coated sensor chip to achieve about 4000 to 4500 RUs of surface density. All the samples were prepared in buffer consisting of 10mM HEPES, pH 7.5, 150 mM NaCl, 2 mM $MgCl_2$, 0.005%

Tween-20 and 2% DMSO. The same buffer was used as the running buffer during the experiments. Compound samples were injected at a flow rate of 30 µL/min for 120 seconds of association time followed by at least 300 seconds of dissociation period. The compounds were tested in a concentration series consisting of at least 6 samples made with 2-fold dilution. The highest concentration was selected based on compound potency in the biochemical assay or binding affinity observed in a previous experiment. Multiple blank injections were run before and after each compound series to allow double reference subtraction during data processing and analysis. Radicicol was tested in every experiment as a positive control to assess activity of the captured protein on the surface. A DMSO curve was run during each experiment to properly correct for excluded volume. The data were processed and analyzed using Bruker Analyzer, Scrubber and Biaeval softwares to calculate binding affinities and kinetics by fitting the data to 1:1 binding model.

Supplementary References:

1. Wright, W.B. Preparation of 3-chlorothieno[3,2-b]thiophene derivatives from thiophene-2-acrylic acids. *J. Heterocycl. Chem.* **9**, 879-882 (1972).
2. Davie, J.R. *et al.* Expression and characterization of branched-chain alpha-ketoacid dehydrogenase kinase from the rat. Is it a histidine-protein kinase? *J Biol Chem* **270**, 19861-19867 (1995).
3. Machius, M., Chuang, J.L., Wynn, R.M., Tomchick, D.R. & Chuang, D.T. Structure of rat BCKD kinase: nucleotide-induced domain communication in a mitochondrial protein kinase. *Proc Natl Acad Sci U S A* **98**, 11218-11223 (2001).
4. Vonrhein, C. *et al.* Data processing and analysis with the autoPROC toolbox. *Acta Crystallogr. D. Biol. Crystallogr.* **67**, 293-302 (2011).
5. Bricogne G., B.E., Brandl M., Flensburg C., Keller P., Paciorek W., Roversi P, Sharff A., Smart O.S., Vonrhein C., Womack T.O. (Global Phasing Ltd., Cambridge, United Kingdom; 2017).
6. Emsley P, C.K. (2004).
7. Smart OS, W., TO, Sharff A, Flensburg C, Keller P, Paciorek W, Vonrhein C and Bricogne G (Global Phasing Ltd., Cambridge, United Kingdom; 2014).
8. Madhavi Sastry, G., Adzhigirey, M., Day, T., Annabhimoju, R. & Sherman, W. Protein and ligand preparation: parameters, protocols, and influence on virtual screening enrichments. *J. Comput. Aided Mol. Des.* **27**, 221-234 (2013).
9. Schrödinger (
10. Epik (New York, NY; 2021).
11. Impact (New York, NY; 2021).
12. Prime (New York, NY; 2021).
13. Jacobson, M.P., Friesner, R.A., Xiang, Z. & Honig, B. On the role of the crystal environment in determining protein side-chain conformations. *J. Mol. Biol.* **320**, 597-608 (2002).
14. Jacobson, M.P. *et al.* A hierarchical approach to all-atom protein loop prediction. *Proteins: Structure, Function, and Bioinformatics* **55**, 351-367 (2004).
15. Bowers, K.J. *et al.* in Proceedings of the ACM/IEEE Conference on Supercomputing (SC06) (Tampa, Florida.
16. Desmond (New York, NY; 2021).
17. Schrödinger (New York, NY; 2021).

18. Lu, C. *et al.* OPLS4: Improving force field accuracy on challenging regimes of chemical space. *Journal of Chemical Theory and Computation* **17**, 4291-4300 (2021).
19. Jorgensen, W.L., Chandrasekhar, J., Madura, J.D., Impey, R.W. & Klein, M.L. Comparison of simple potential functions for simulating liquid water. *The Journal of Chemical Physics* **79**, 926-935 (1983).
20. Knapp, B., Ospina, L. & Deane, C.M. Avoiding false positive conclusions in molecular simulation: The importance of replicas. *Journal of Chemical Theory and Computation* **14**, 6127-6138 (2018).
21. Vassaux, M., Wan, S., Edeling, W. & Coveney, P.V. Ensembles Are Required to Handle Aleatoric and Parametric Uncertainty in Molecular Dynamics Simulation. *J Chem Theory Comput* **17**, 5187-5197 (2021).
22. Poongavanam, V. *et al.* Linker-Dependent Folding Rationalizes PROTAC Cell Permeability. *J. Med. Chem.* **65**, 13029-13040 (2022).
23. Ball, K.A. *et al.* Conformational Dynamics of the HIV-Vif Protein Complex. *Biophys. J.* **116**, 1432-1445 (2019).
24. Kato, M., Chuang, J.L., Tso, S.C., Wynn, R.M. & Chuang, D.T. Crystal structure of pyruvate dehydrogenase kinase 3 bound to lipoyl domain 2 of human pyruvate dehydrogenase complex. *EMBO J.* **24**, 1763-1774 (2005).
25. Tso, S.C. *et al.* Structure-based design and mechanisms of allosteric inhibitors for mitochondrial branched-chain alpha-ketoacid dehydrogenase kinase. *Proc Natl Acad Sci U S A* **110**, 9728-9733 (2013).
26. Tso, S.C. *et al.* Benzothiophene carboxylate derivatives as novel allosteric inhibitors of branched-chain alpha-ketoacid dehydrogenase kinase. *J. Biol. Chem.* **289**, 20583-20593 (2014).
27. Maguolo, A. *et al.* A gain-of-function mutation on BCKDK gene and its possible pathogenic role in branched-chain amino acid metabolism. *Genes* **13**, 233 (2022).
28. Huang, T. *et al.* MSstatsTMT: Statistical detection of differentially abundant proteins in experiments with isobaric labeling and multiple mixtures. *Mol Cell Proteomics* **19**, 1706-1723 (2020).
29. Liu, S. *et al.* Structural studies identify angiotensin II receptor blocker-like compounds as branched-chain ketoacid dehydrogenase kinase inhibitors. *J Biol Chem* **299**, 102959 (2023).

Supplementary Figure 11. Western blots

pBCKDH pS292 (Bethyl A304-672A 1:1000)
total BCKDH (Yenzym 037 1:1000)
BDK (abcam ab128935 1:1000)
GAPDH (CST 14C10 1:10000)

Related to Figure 1

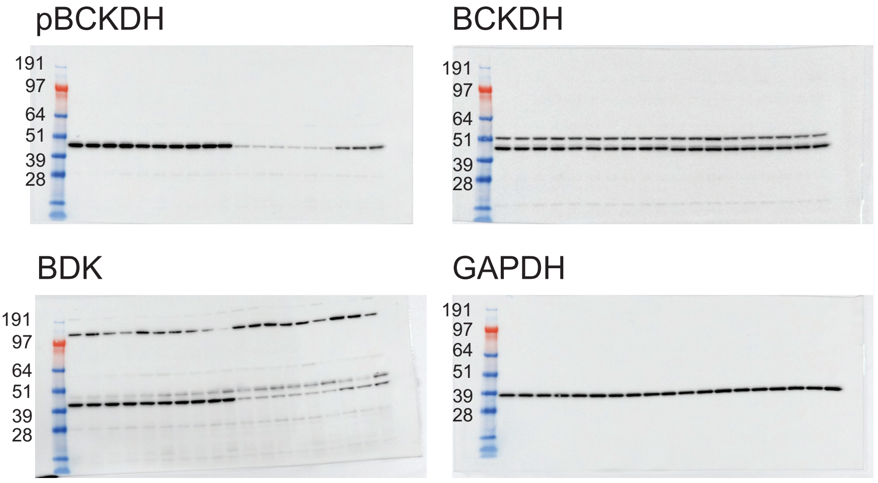

Related to Figure 6A

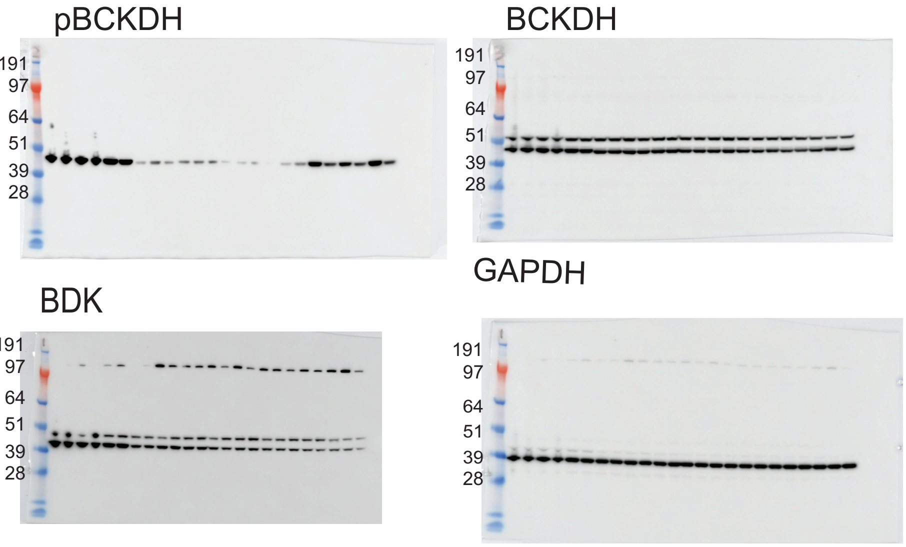

Related to Figure 6C

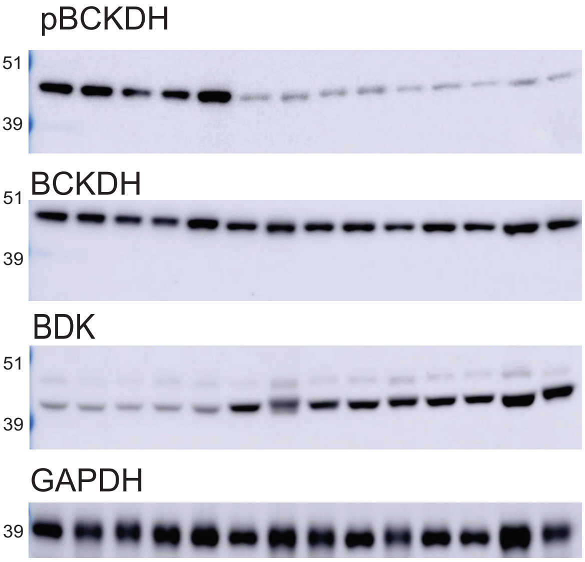

Related to Figure 6E

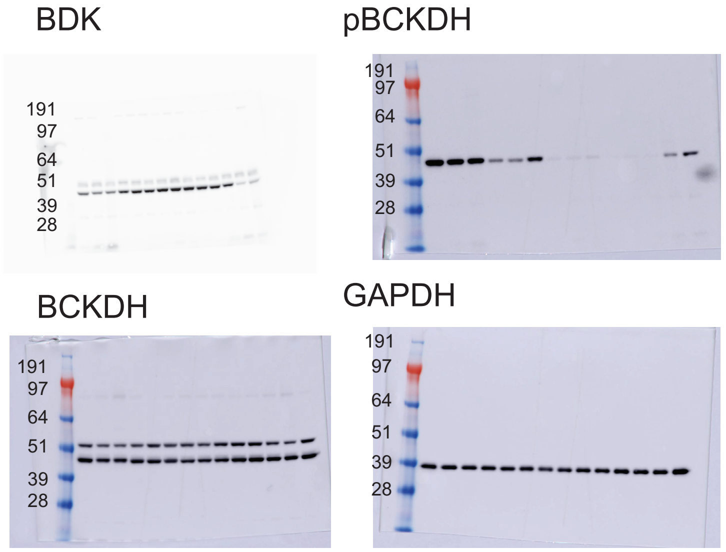

Related to Figure 7A

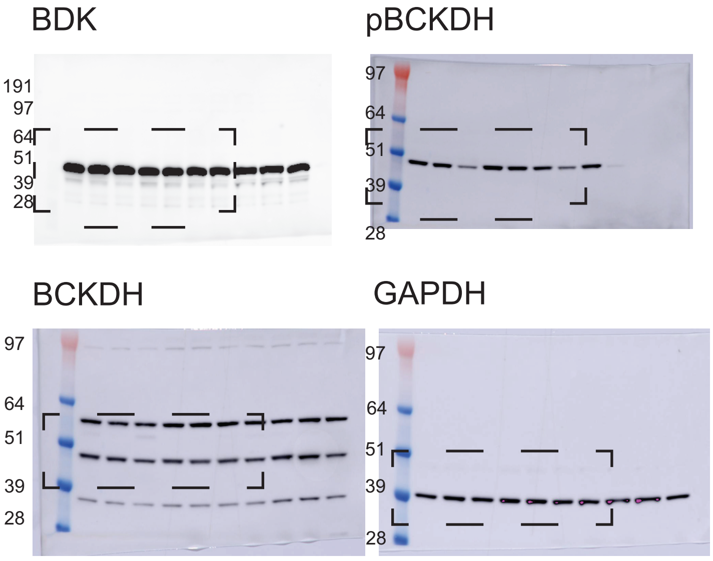

Related to Figure 7C

BDK

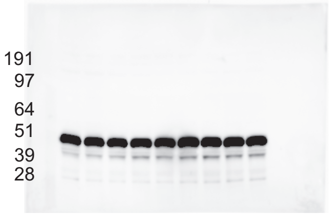

pBCKDH

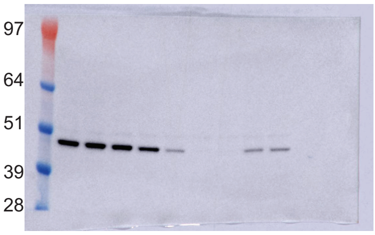

BCKDH

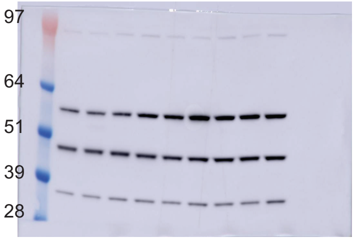

GAPDH

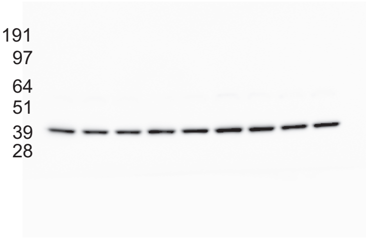

Related to Figure 8B

BDK

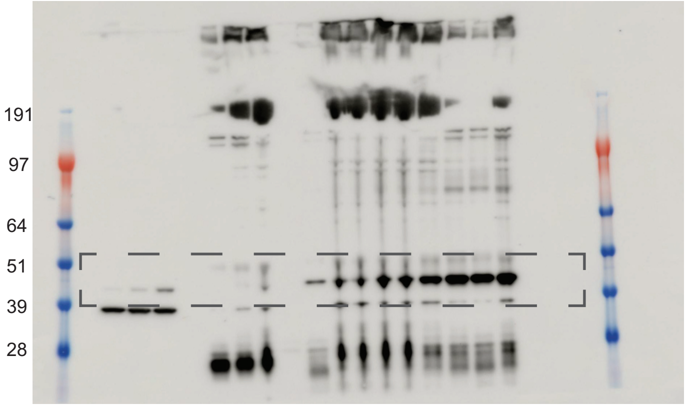

Related to Supplementary Figure 8E

pBCKDH

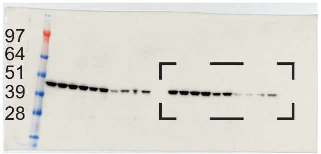

BCKDH

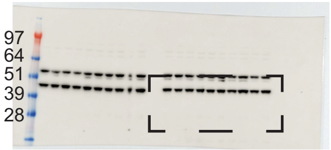

GAPDH

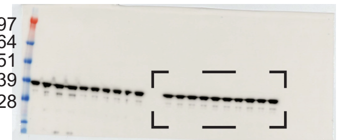

BDK

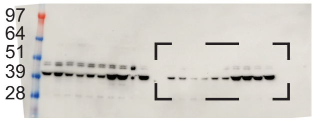

Related to Supplementary Figure 8F

pBCKDH

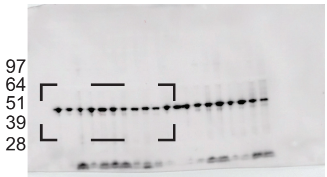

BCKDH

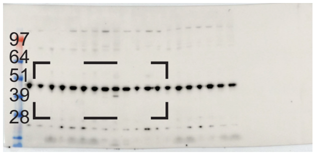

GAPDH

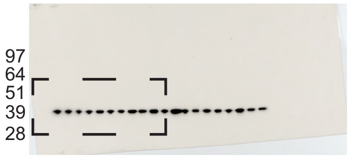

BDK

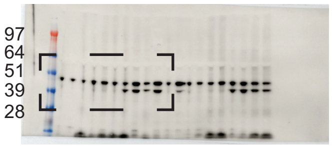

Supplementary Figure 12. 1H NMR of compound S4

Compound S4, 1H NMR (d-DMSO, 400MHz

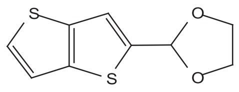

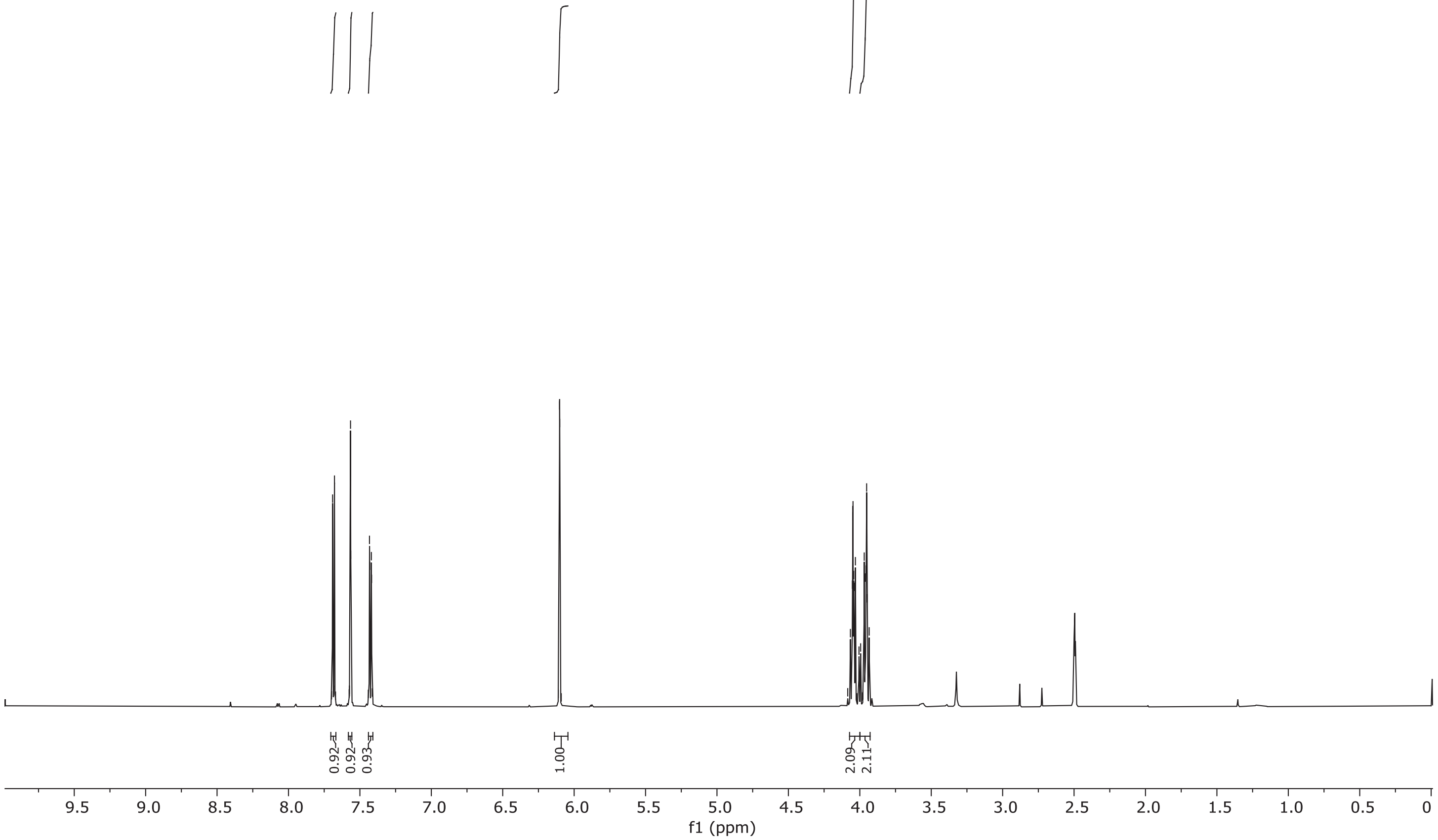

Supplementary Figure 13. HRMS of compound S4

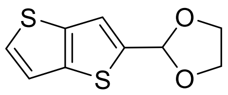

S4

Open Access HRMS Sample Report			
**Sample ID**	00110407-5465-005	**HPLC**	Agilent 1200
**Molecular Formula**	C9H8O2S2	**Mass Spectrometer**	Sciex 5600+ QToF
**Submittor**	Martinez Alsina, Luis A	**DAD**	190-400 nm, reported 270nm
**Run Date**	4/4/2023	**MS Scan**	100-2000 amu
**Analyst**	Quinn, Alandra	**Acquisition Method**	HRMS-3min gradient AQ
**Analyst's CeN**	00714673-0163	**eWB Archive Ref**	00713459-0292

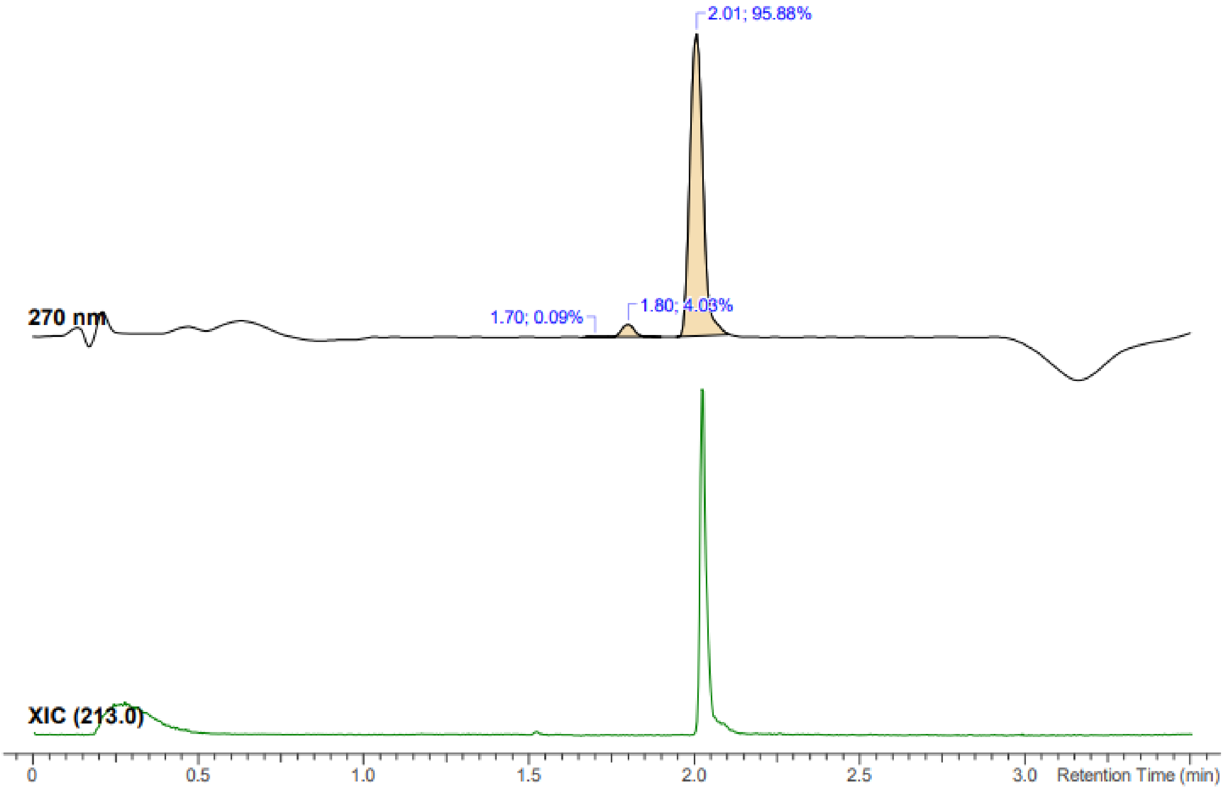

No.	Peak Name	tR (min)	Area (%)
1	unknown	1.70	0.09
2	unknown	1.80	4.03
3	C9H8O2S2	2.01	95.88

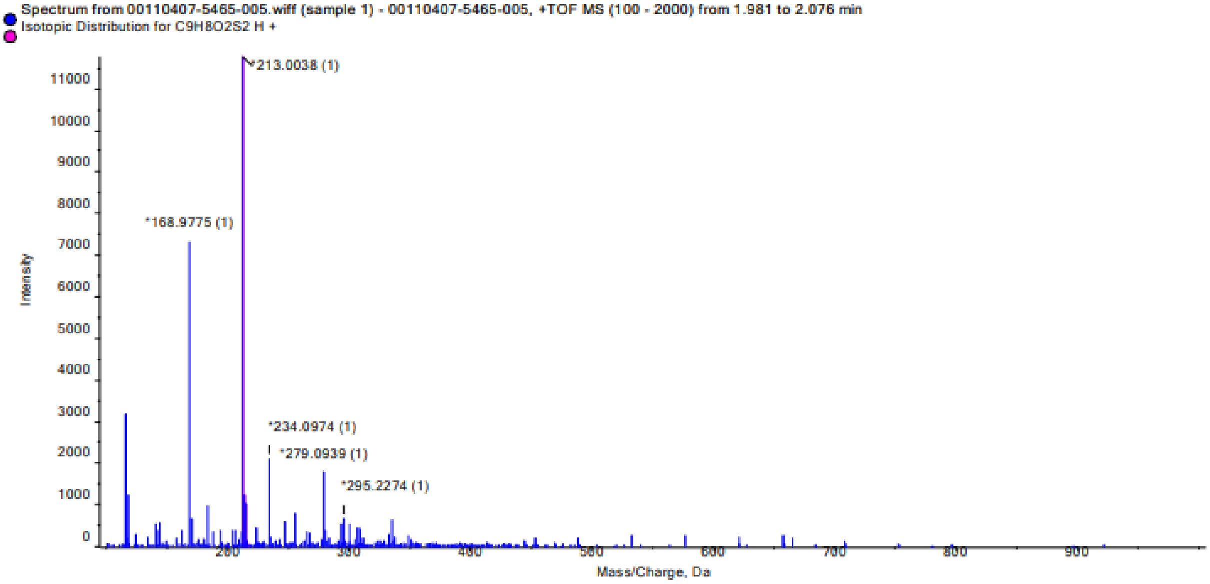

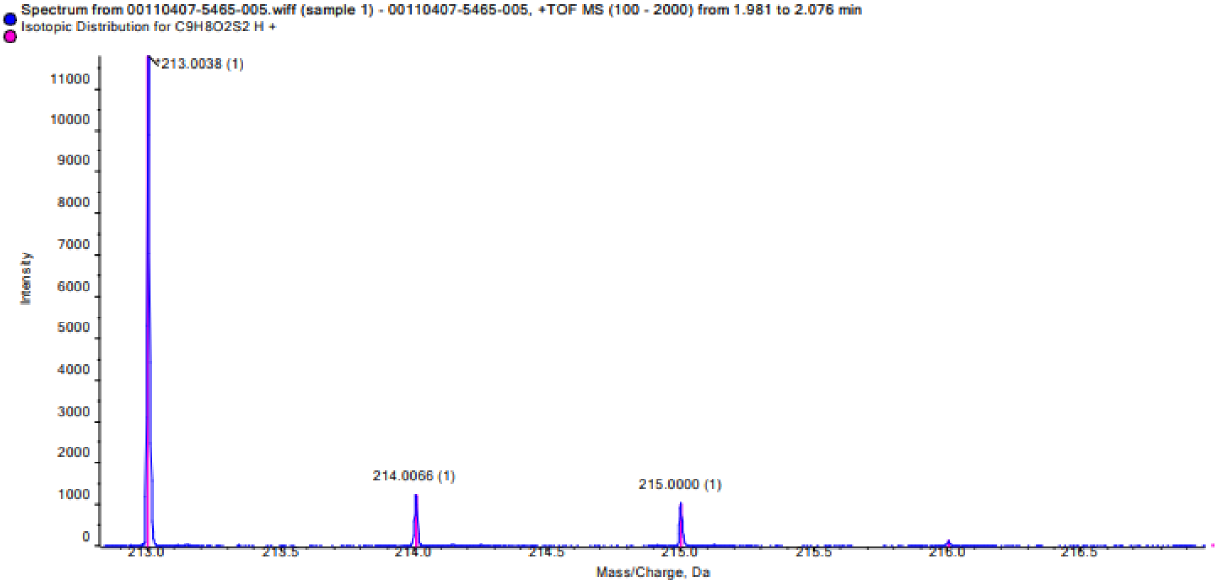

Peak at 2.01 min in UV								
Mass/Charge (Da)	Height	Relative % Height	Compound	Peak Type	Theoretical m/z	Error (ppm)	Theoretical m/z	Theoretical Intensity
213.0038	11774	100.0	C9H8O2S2	[M+H]+	213.0039	-0.1	213.0039	100.0
214.0066	1237	10.5					214.0067	11.8
215.0000	1046	8.9					215.0006	9.9
216.0037	128	1.1					216.0033	1.0

Supplementary Figure 14. HPLC of compound S4

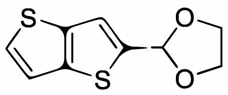

S4

Sampl. Name: 00132438-141-05

Acq. Method: D:\Chem32\HPLC-007\DATA\2019\040219\123790-040219-1820 2019-04-02 13-39-26\AM-123790-02-M1.M
Data File: D:\CHEM32\HPLC-007\DATA\2019\040219\123790-040219-1820 2019-04-02 13-39-26\00132438-141-05-.D
User Name: LN Acq. Instrument: HPLC-007
Acq. Date: 04/02/2019 18:34:37 Injection Vol.: 2.0 µl

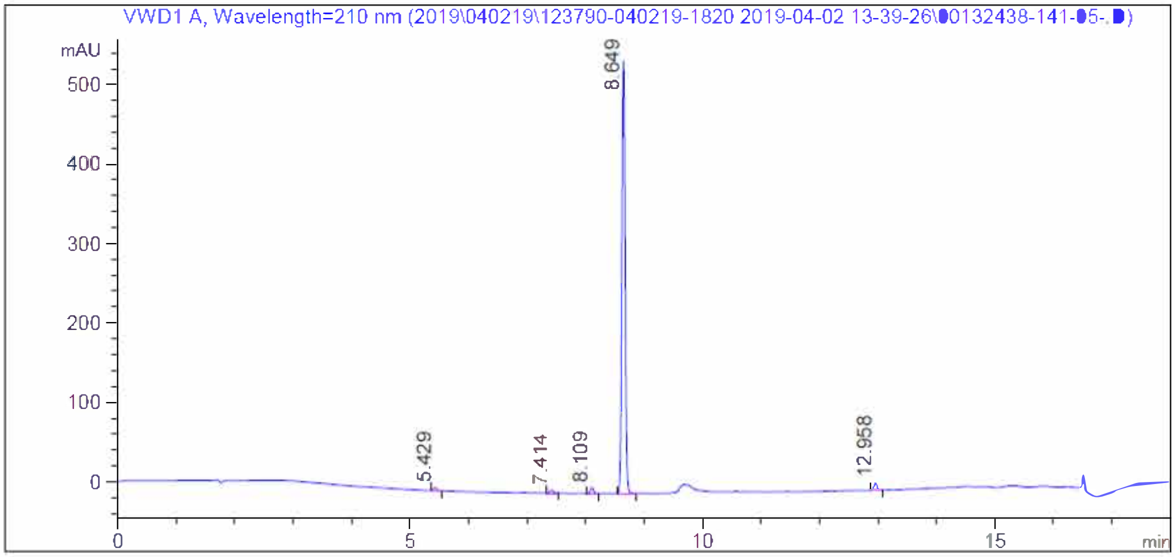

#	Meas. Re	Width	Symmetry	Height	Area	Area %
1	5.4291	0.0630	0.8142	3.8512	14.5653	0.6555
2	7.4143	0.0780	0.9469	4.1883	19.5896	0.8816
3	8.1092	0.0639	0.9313	7.0948	27.2131	1.2247
4	8.6492	0.0649	0.9675	545.4932	2123.0632	95.5439
5	12.9580	0.0679	0.9056	9.2360	37.6493	1.6943

Supplementary Figure 15. 1H NMR of compound S5

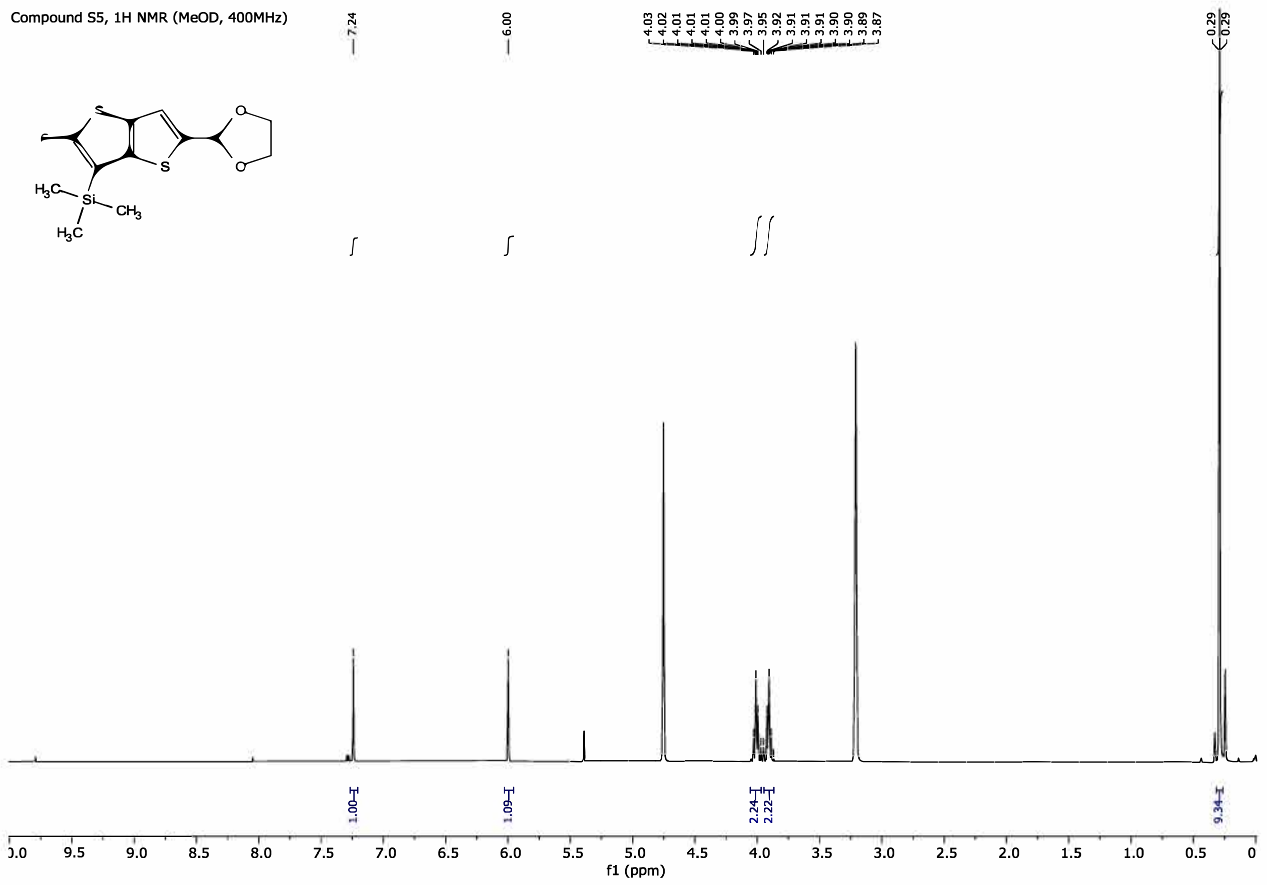

Supplementary Figure 16. 19F NMR of compound S5

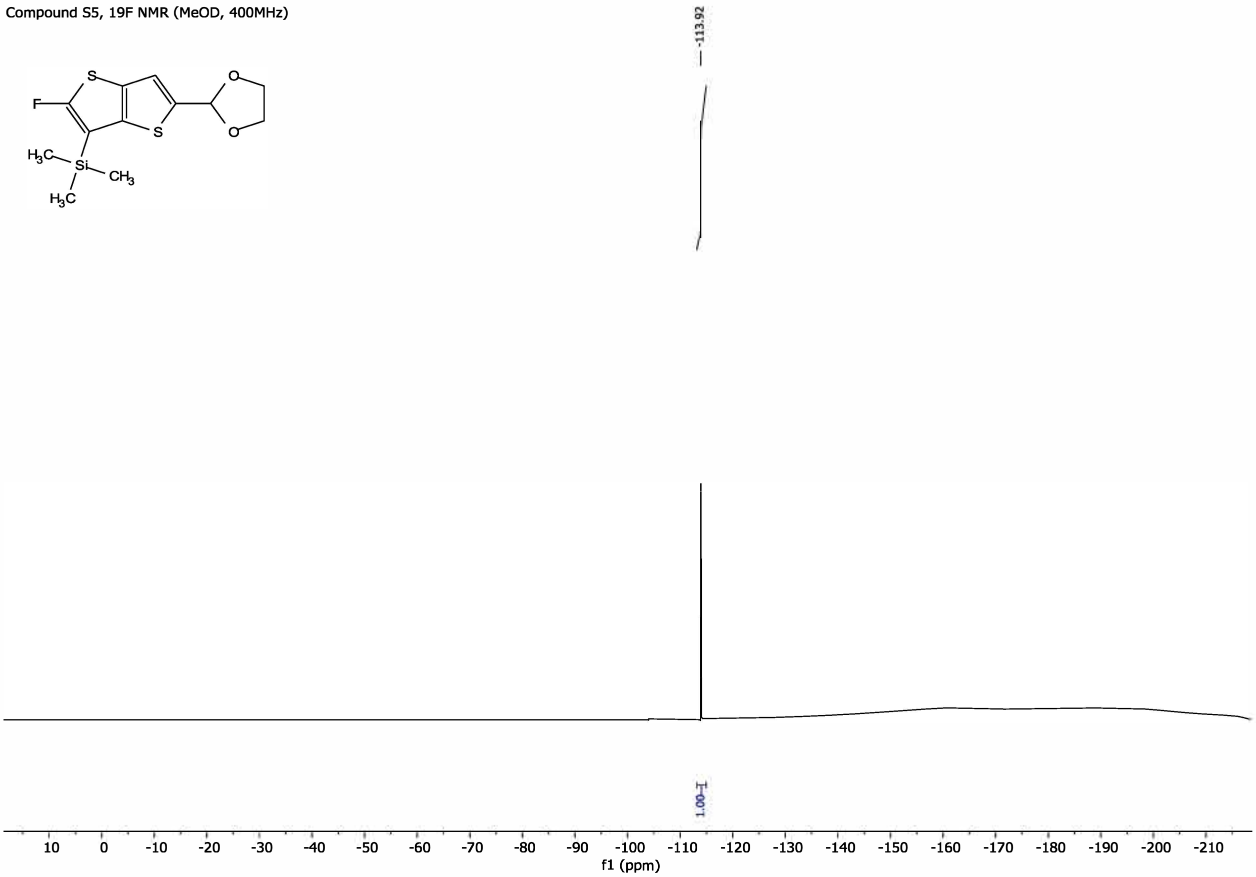

Supplementary Figure 17. 1H NMR of compound S7

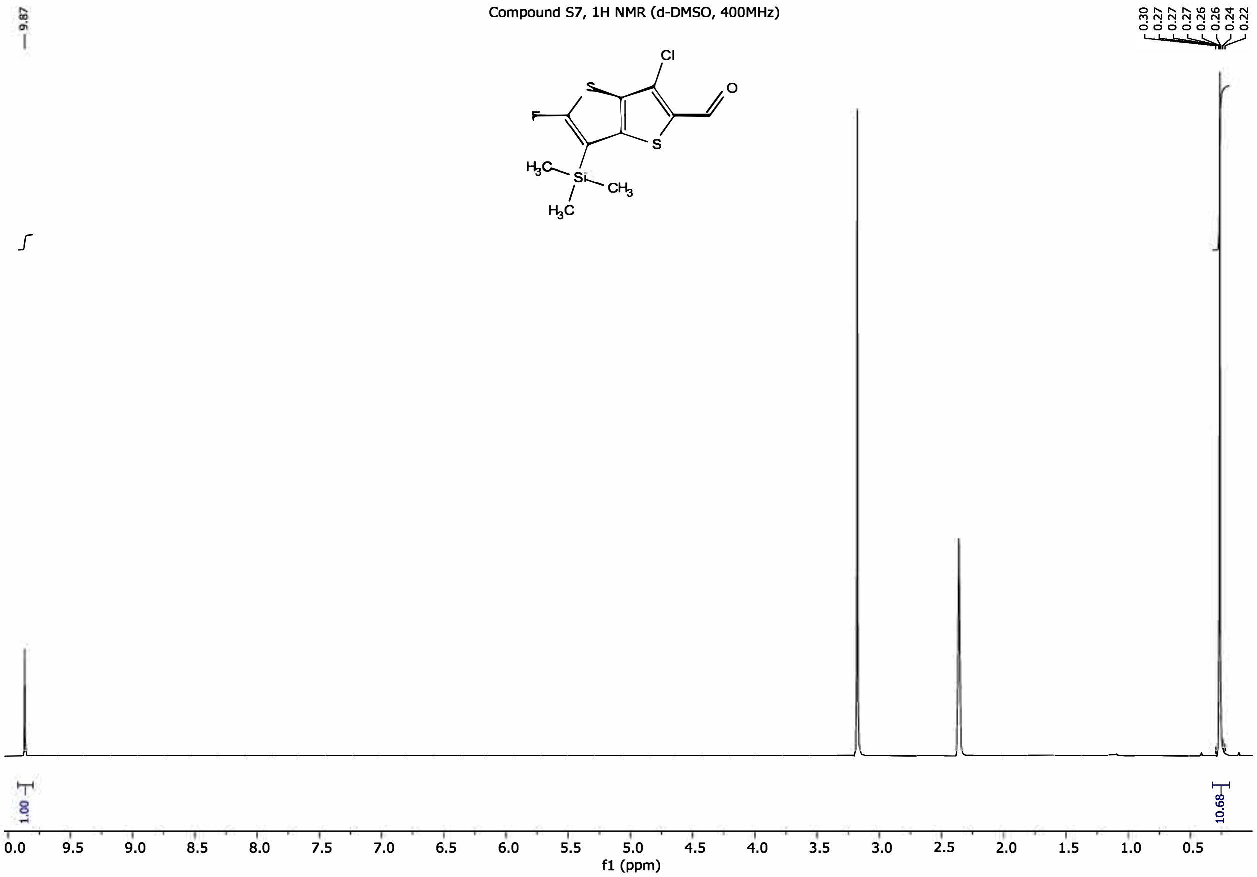

Supplementary Figure 18. 13C NMR of compound S7

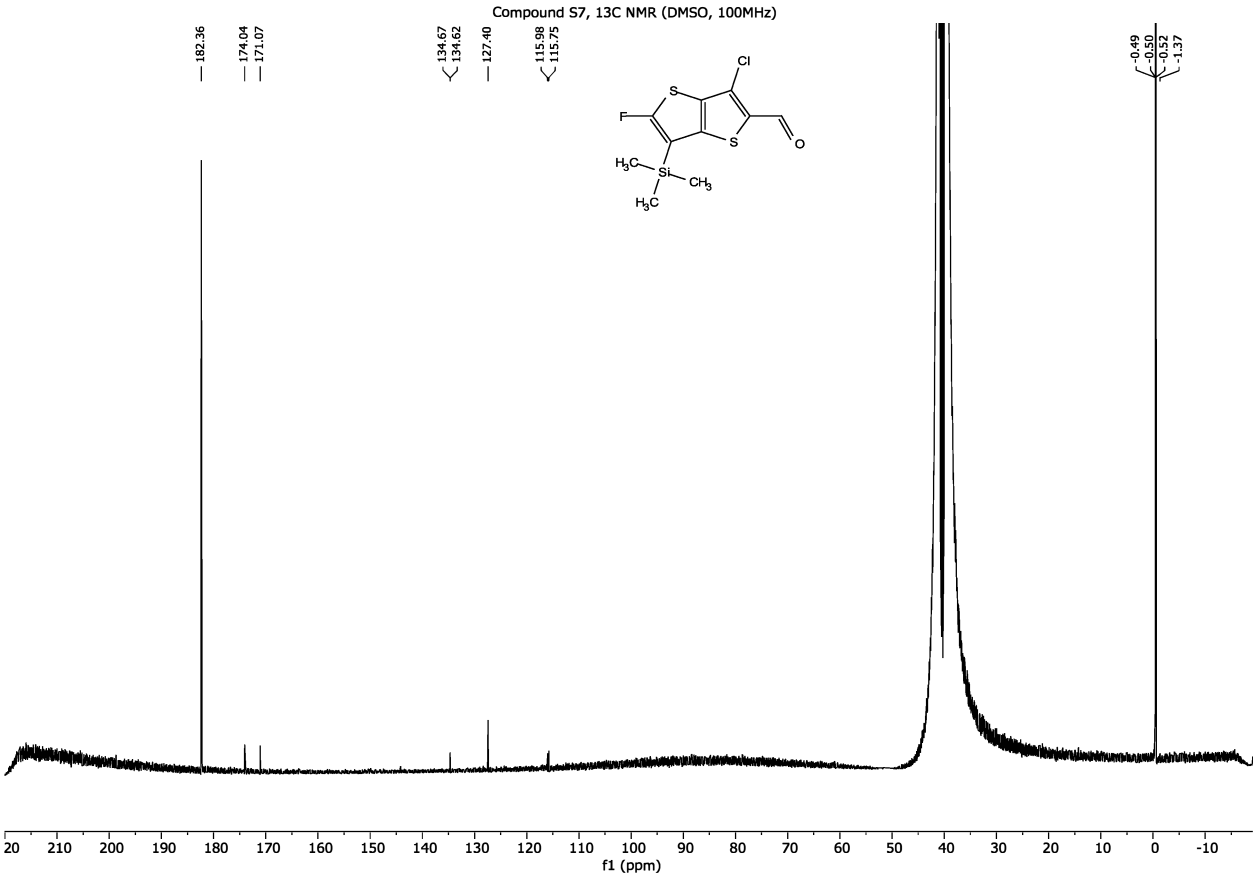

Supplementary Figure 19. 19F NMR of compound S7

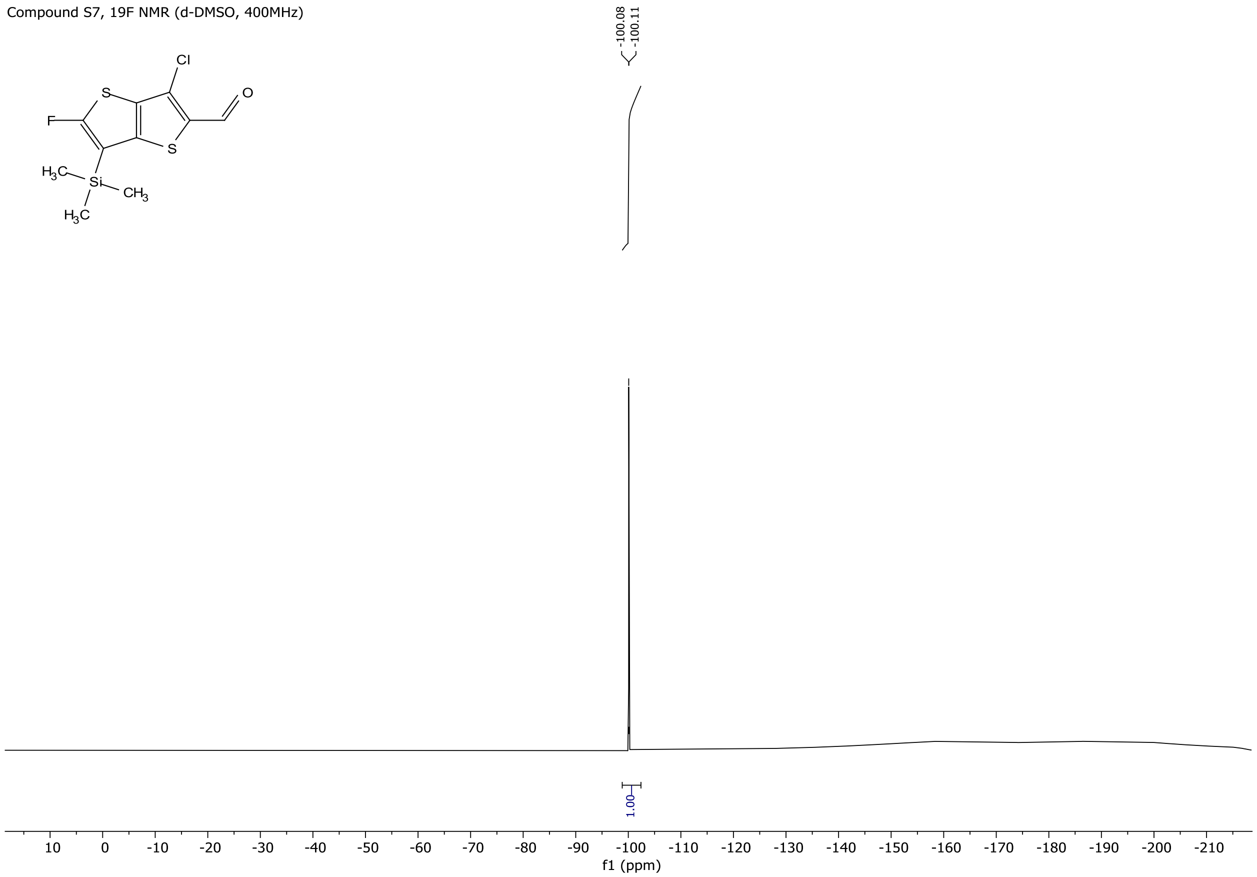

Supplementary Figure 20. HRMS of compound S7

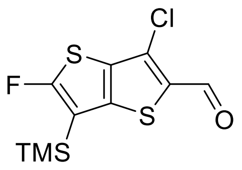

S7

Open Access HRMS Sample Report			
**Sample ID**	00110407-5465-003	**HPLC**	Agilent 1200
**Molecular Formula**	C10H10ClFOS2Si	**Mass Spectrometer**	Sciex 5600+ QToF
**Submittor**	Martinez Alsina, Luis A	**DAD**	190-400 nm, reported 270nm
**Run Date**	4/4/2023	**MS Scan**	100-2000 amu
**Analyst**	Quinn, Alandra	**Acquisition Method**	HRMS-3min gradient AQ
**Analyst's CeN**	00714673-0163	**eWB Archive Ref**	00713459-0292

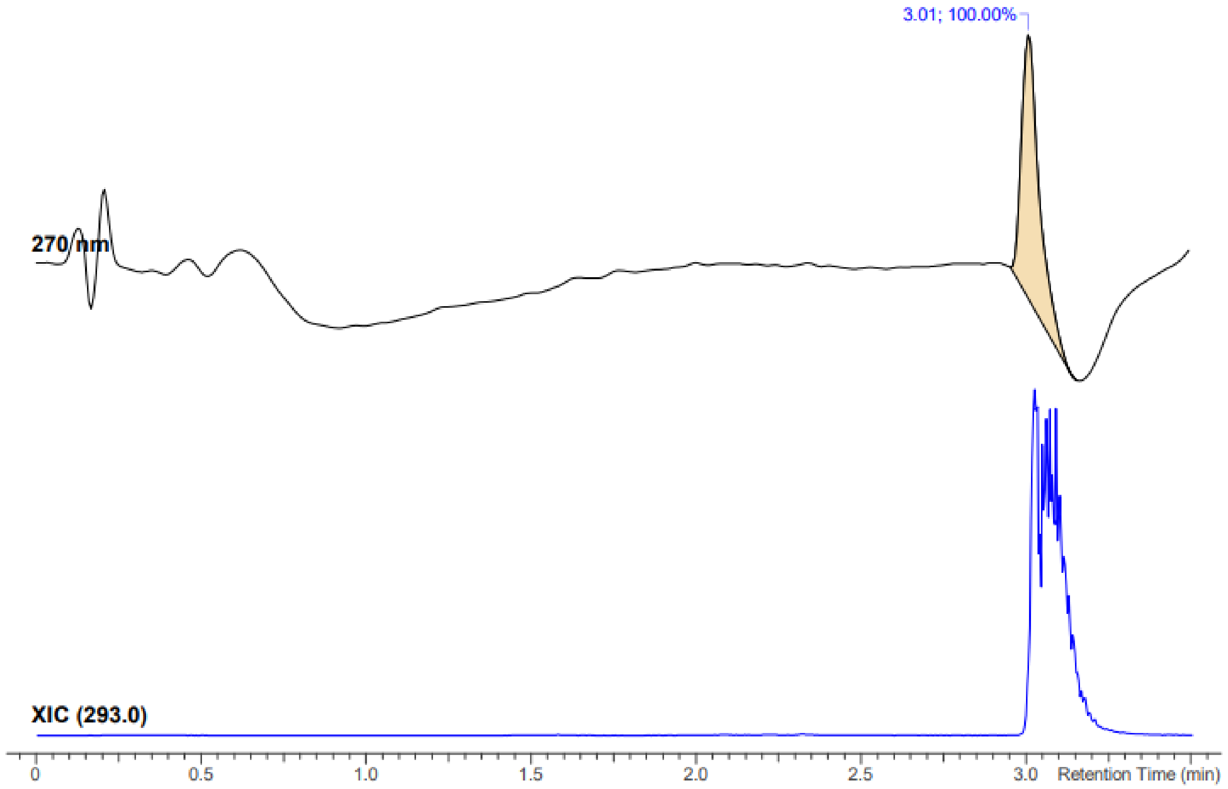

No.	Peak Name	tR (min)	Area (%)
1	C10H10ClFOS2Si	3.01	100.00

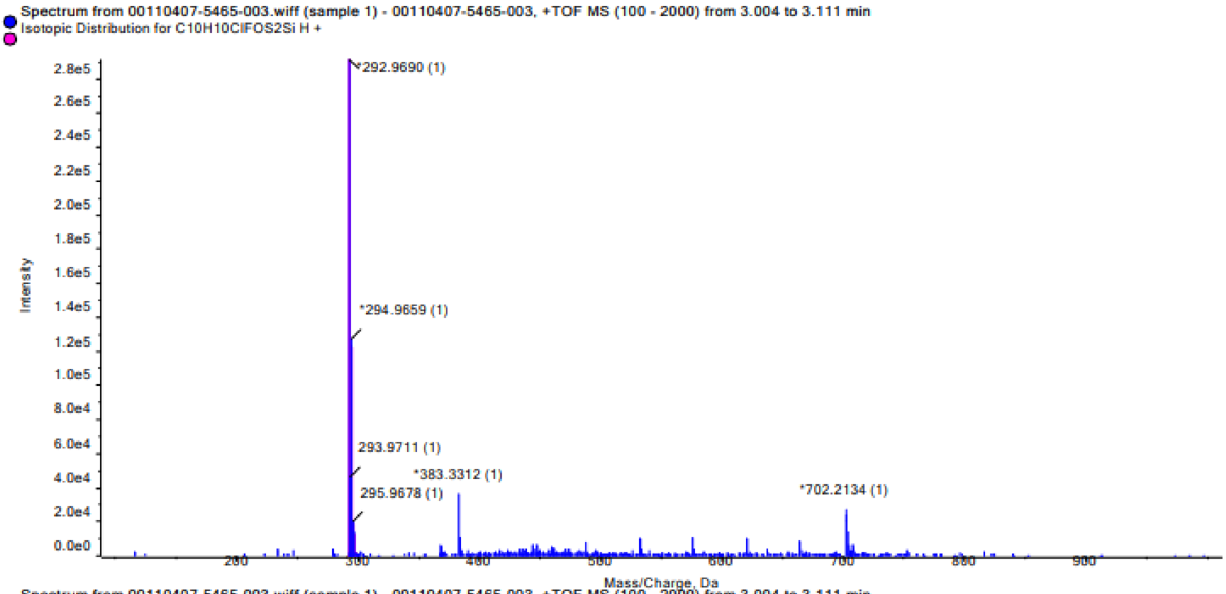

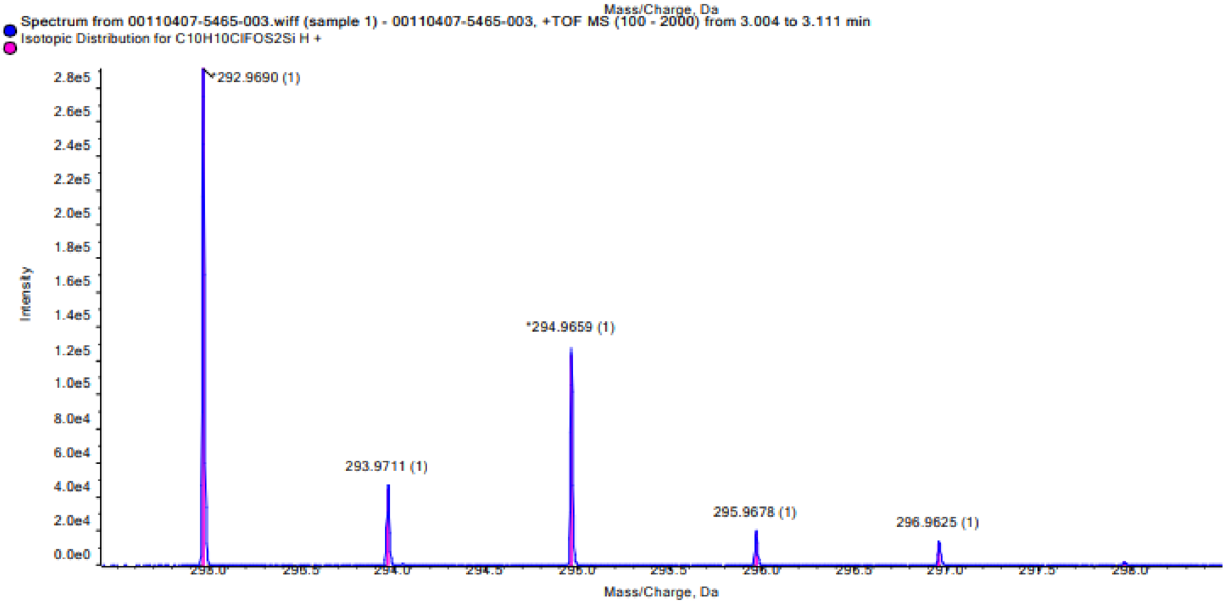

Peak at 3.01 min in UV								
Mass/Charge (Da)	Height	Relative % Height	Compound	Peak Type	Theoretical m/z	Error (ppm)	Theoretical m/z	Theoretical Intensity
292.9690	291363	100.0	C10H10ClFOS2Si	[M+H]+	292.9688	0.8	292.9688	100.0
293.9711	46906	16.1					293.9707	17.9
294.9659	127292	43.7					294.9658	45.8
295.9678	19914	6.8					295.9677	7.8
296.9625	13923	4.8					296.9629	5.1

Supplementary Figure 21. HPLC of compound S7

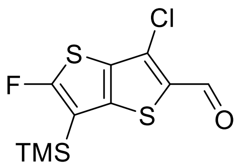

S7

```
=====================================================================
Acq. Operator   : SYSTEM                          Seq. Line :   3
Sample Operator : SYSTEM
Acq. Instrument : LC-325GF                         Location :   3
Injection Date  : 3/17/2023 4:12:37 PM                  Inj :   1
                                                Inj Volume : 5.000 µl
Different Inj Volume from Sample Entry!  Actual Inj Volume : 10.000 µl
Sequence File   : C:\Users\Public\Documents\ChemStation\1\Data\DEF_LC_2021 2023-03-17 15-41
                  -03\DEF_LC_2021.S
Method          : C:\Users\Public\Documents\ChemStation\1\Data\DEF_LC_2021 2023-03-17 15-41
                  -03\ACID_CLASSIC.M (Sequence Method)
Last changed    : 6/8/2017 9:26:22 AM by SYSTEM
Method Info     : ACID METHOD for classic look on new Chemstation software
                  Column: XBridge C18 5 micron (4.6 mm x 150 mm)
                  Flow rate: 1.500 mL/min with solvents containing 0.1% TFA
                  0-1.5 min: 5% acetonitrile/water
                  1.5-10 min: 5-100% acetonitrile water
                  10-11 min: 100% acetonitrile
                  11-12.5 min: 100-5% acetonitrile/water
```

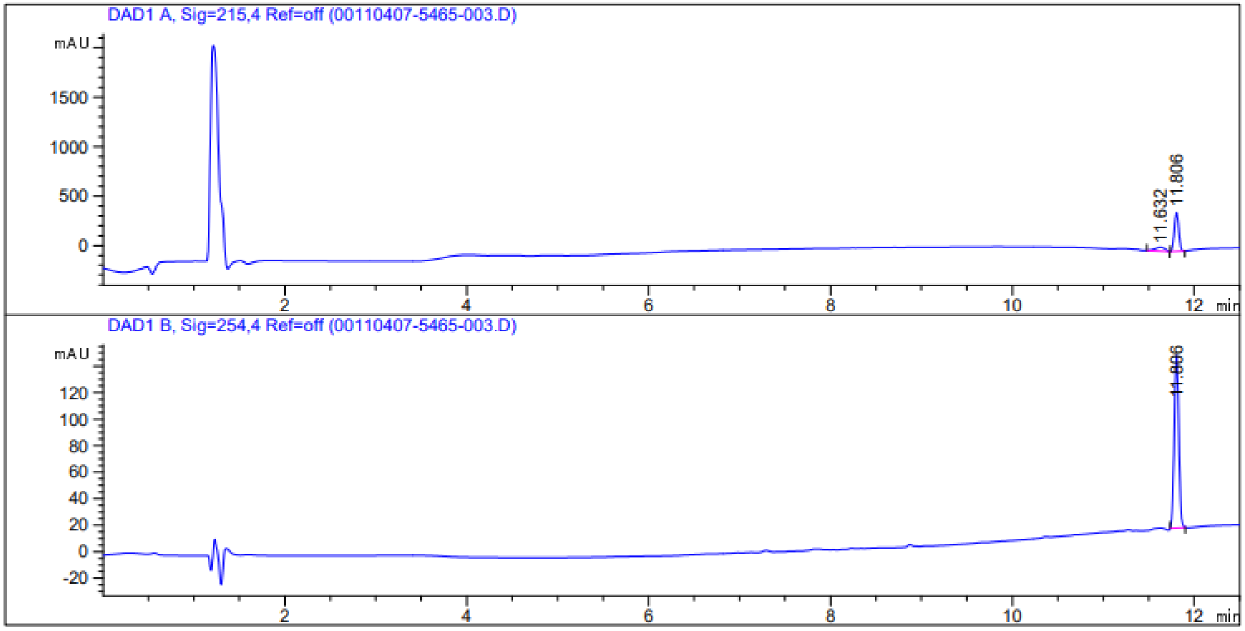


```
=====================================================================
                         Area Percent Report
=====================================================================

Sorted By             :      Signal
Multiplier            :      1.0000
Dilution              :      1.0000
Do not use Multiplier & Dilution Factor with ISTDs
```

Signal 1: DAD1 A, Sig=215,4 Ref=off

Peak #	RetTime [min]	Type	Width [min]	Area [mAU*s]	Height [mAU]	Area %
1	11.632	BB	0.1213	314.89941	42.08999	19.1677
2	11.806	BB	0.0511	1327.96643	396.49451	80.8323

Totals : 1642.86584 438.58450

Signal 2: DAD1 B, Sig=254,4 Ref=off

Peak #	RetTime [min]	Type	Width [min]	Area [mAU*s]	Height [mAU]	Area %
1	11.806	BB	0.0523	453.53329	131.35742	100.0000

Totals : 453.53329 131.35742

==

*** End of Report ***

Supplementary Figure 22. 1H NMR of compound S8

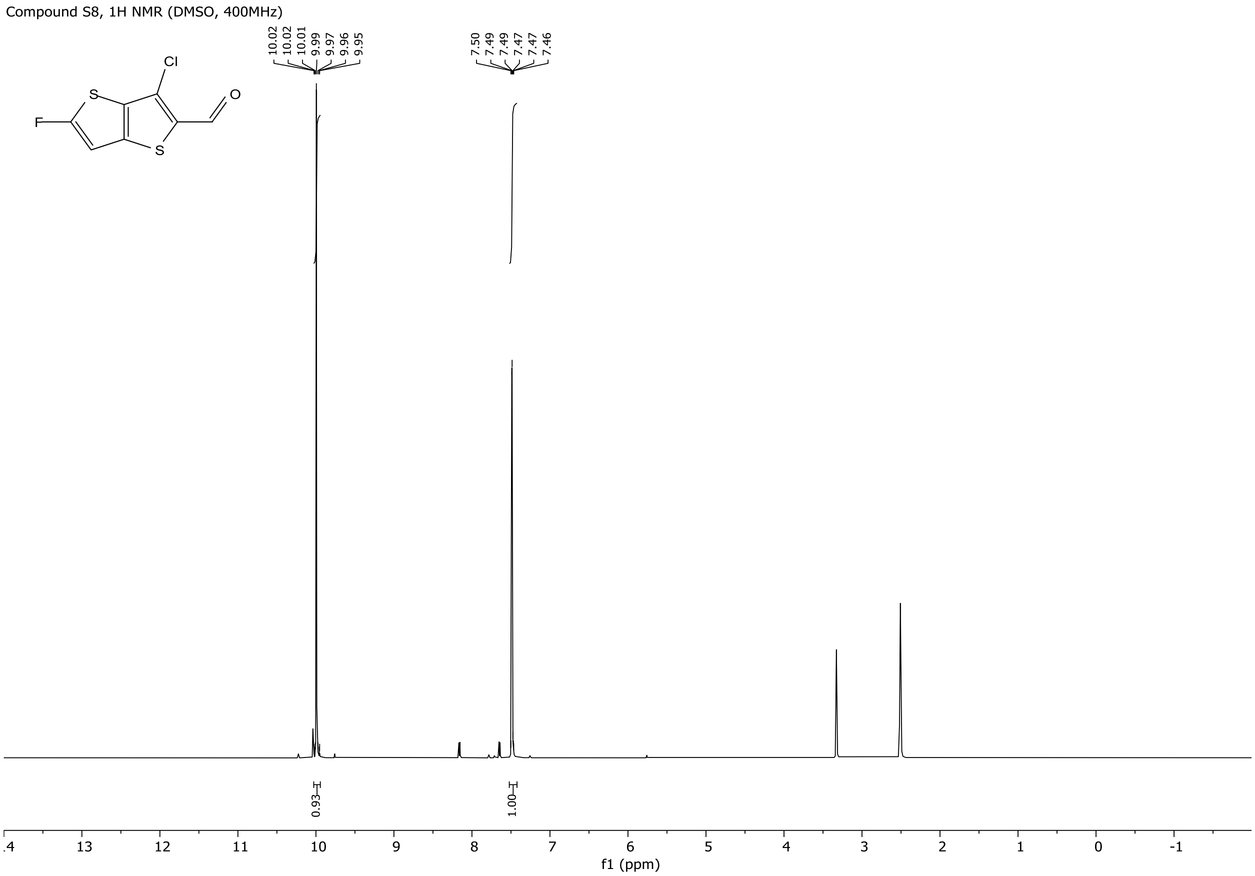

Supplementary Figure 23. 13C NMR of compound S8

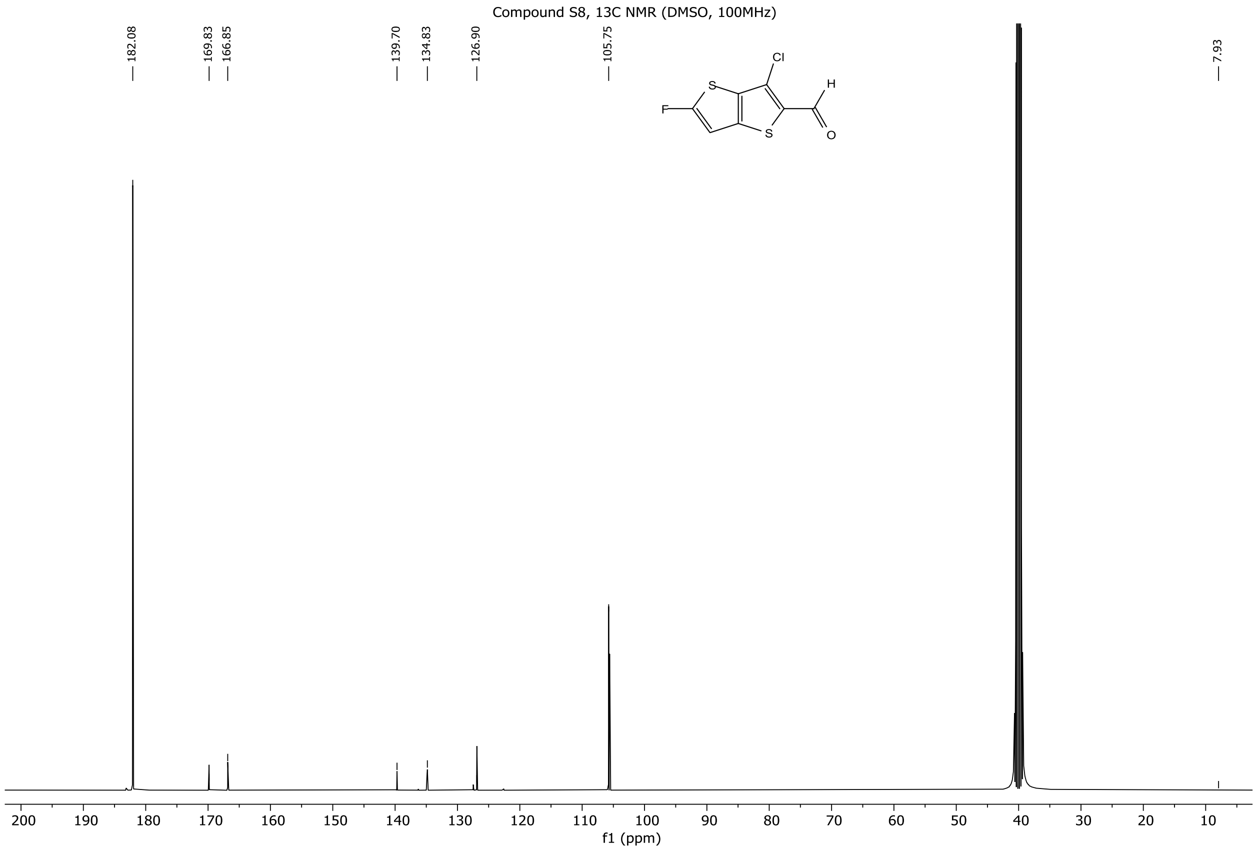

Supplementary Figure 24. 19F NMR of compound S8

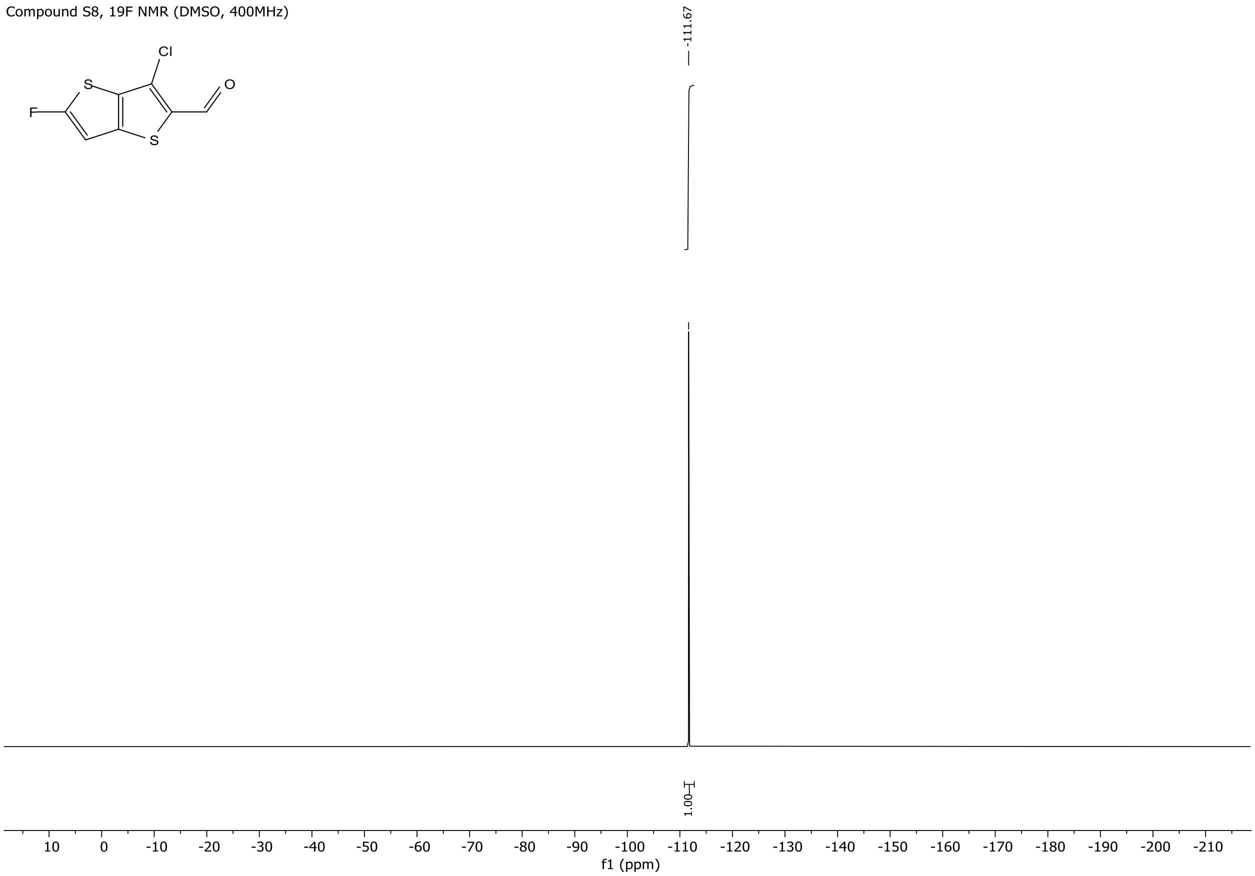

Supplementary Figure 25. HRMS of compound S8

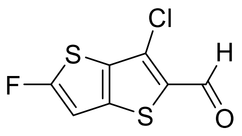

S8

Open Access HRMS Sample Report			
**Sample ID**	00110407-5465-004	**HPLC**	Agilent 1200
**Molecular Formula**	C7H2ClFOS2	**Mass Spectrometer**	Sciex 5600+ QToF
**Submittor**	Martinez Alsina, Luis A	**DAD**	190-400 nm, reported 270nm
**Run Date**	4/4/2023	**MS Scan**	100-2000 amu
**Analyst**	Quinn, Alandra	**Acquisition Method**	HRMS-3min gradient AQ
**Analyst's CeN**	00714673-0163	**eWB Archive Ref**	00713459-0292

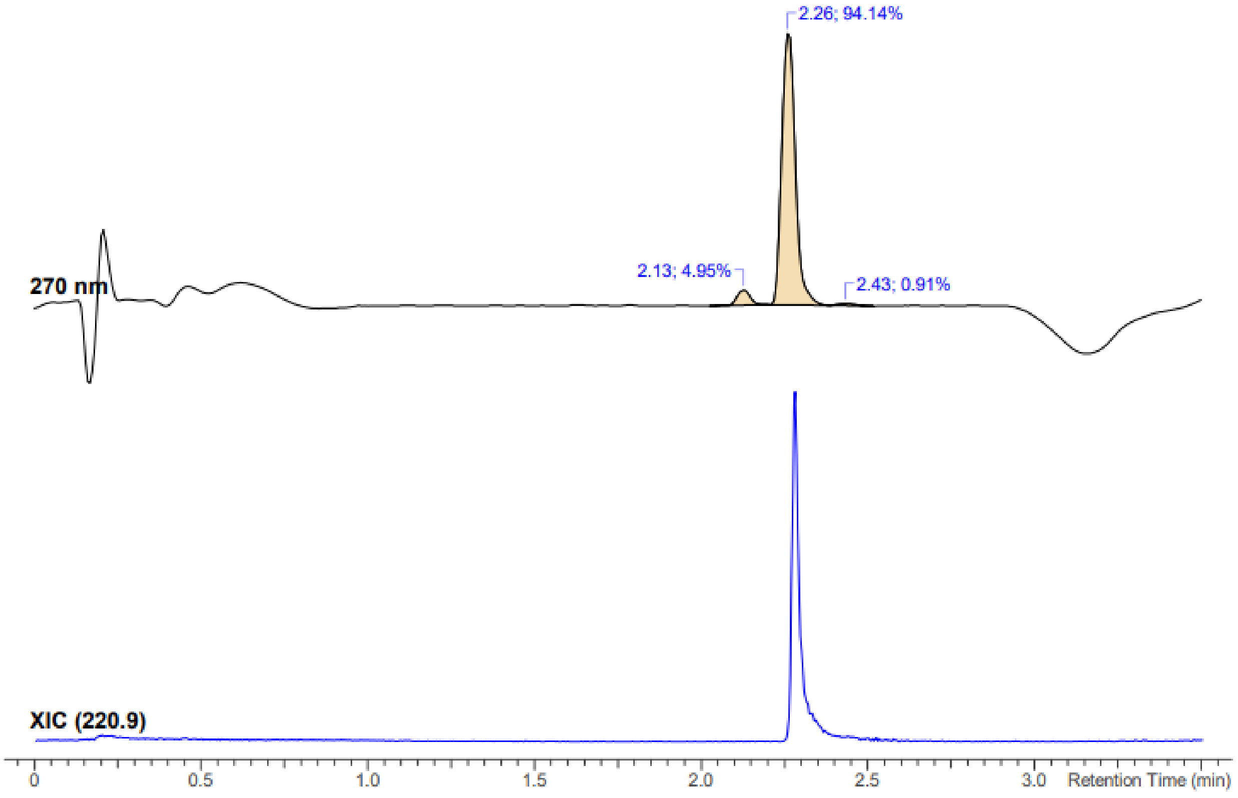

No.	Peak Name	tR (min)	Area (%)
1	unknown	2.13	4.95
2	C7H2ClFOS2	2.26	94.14
3	unknown	2.43	0.91

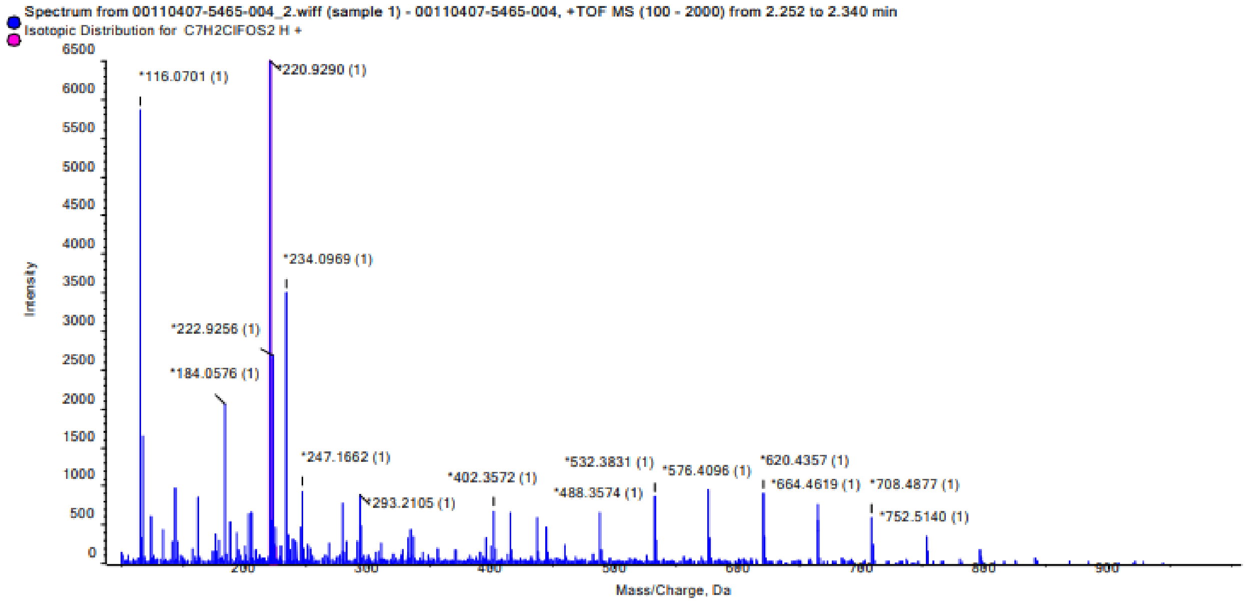

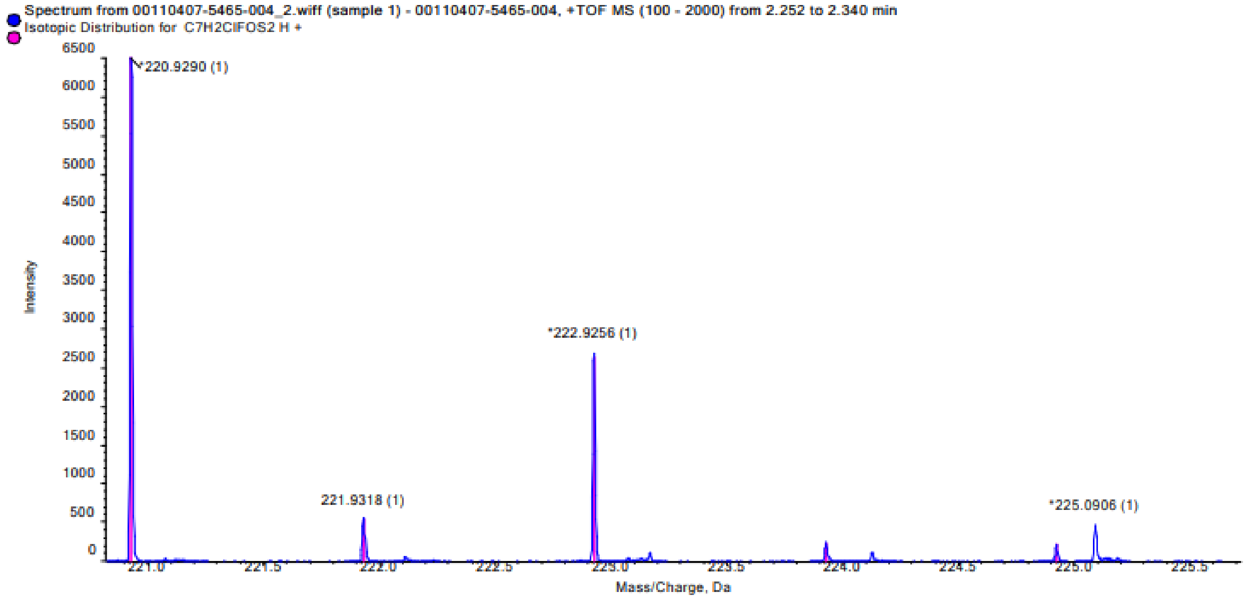

Peak at 2.26 min in UV									
Mass/Charge (Da)	Height	Relative % Height	Compound	Peak Type	Theoretical m/z	Error (ppm)	Theoretical m/z	Theoretical Intensity
220.9290	6500	100.0	C7H2ClFOS2	[M+H]+	220.9292	-1.3	220.9292	100.0
221.9318	548	8.4					221.9319	9.4
222.9256	2689	41.4					222.9261	41.4
223.9283	249	3.8					223.9289	3.8
224.9221	209	3.2					224.9227	3.3

Supplementary Figure 26. HPLC of compound S8

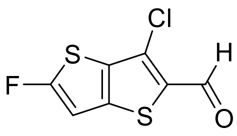

S8

```
======================================================================
Acq. Operator   : SYSTEM                          Seq. Line :   4
Sample Operator : SYSTEM
Acq. Instrument : LC-325GF                         Location :   4
Injection Date  : 3/17/2023 4:26:47 PM                  Inj :   1
                                                Inj Volume : 5.000 µl
Different Inj Volume from Sample Entry!  Actual Inj Volume : 10.000 µl
Sequence File   : C:\Users\Public\Documents\ChemStation\1\Data\DEF_LC_2021 2023-03-17 15-41
                  -03\DEF_LC_2021.S
Method          : C:\Users\Public\Documents\ChemStation\1\Data\DEF_LC_2021 2023-03-17 15-41
                  -03\ACID_CLASSIC.M (Sequence Method)
Last changed    : 6/8/2017 9:26:22 AM by SYSTEM
Method Info     : ACID METHOD for classic look on new Chemstation software
                  Column: XBridge C18 5 micron (4.6 mm x 150 mm)
                  Flow rate: 1.500 mL/min with solvents containing 0.1% TFA
                  0-1.5 min: 5% acetonitrile/water
                  1.5-10 min: 5-100% acetonitrile water
                  10-11 min: 100% acetonitrile
                  11-12.5 min: 100-5% acetonitrile/water
```

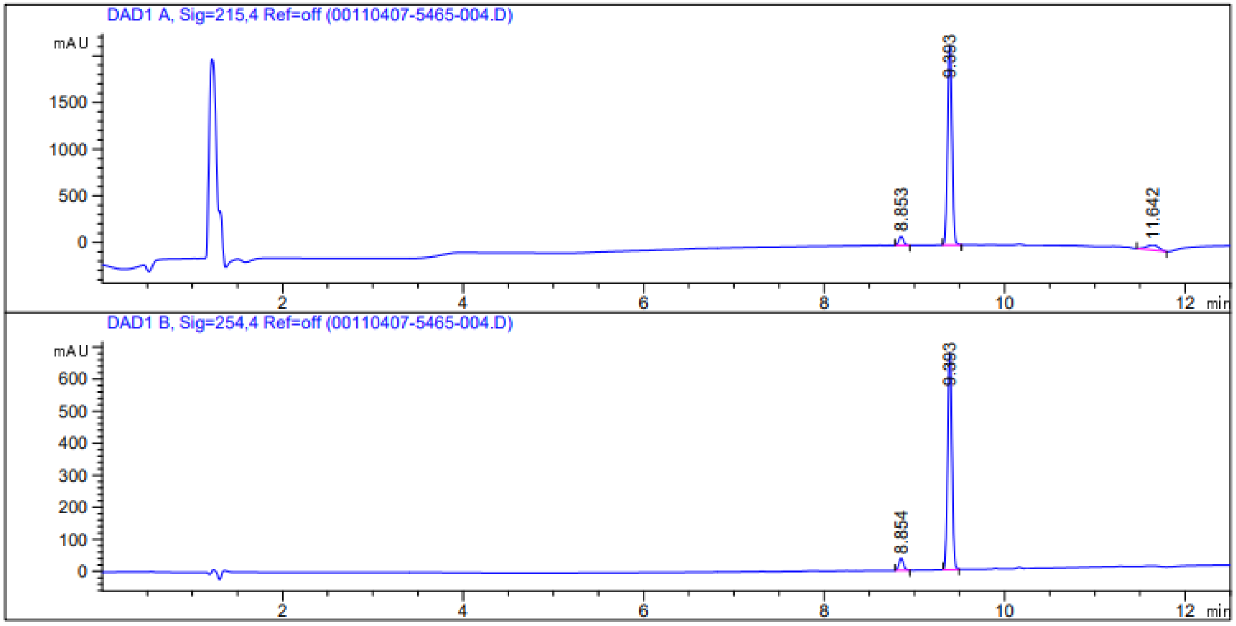


```
======================================================================
                         Area Percent Report
======================================================================

Sorted By             :      Signal
Multiplier            :      1.0000
Dilution              :      1.0000
Do not use Multiplier & Dilution Factor with ISTDs
```

Signal 1: DAD1 A, Sig=215,4 Ref=off

Peak #	RetTime [min]	Type	Width [min]	Area [mAU*s]	Height [mAU]	Area %
1	8.853	BB	0.0546	337.35281	96.83382	4.0003
2	9.393	BB	0.0570	7575.64990	2155.09009	89.8311
3	11.642	BB	0.1470	520.20831	55.69928	6.1686

Totals : 8433.21103 2307.62319

Signal 2: DAD1 B, Sig=254,4 Ref=off

Peak #	RetTime [min]	Type	Width [min]	Area [mAU*s]	Height [mAU]	Area %
1	8.854	BB	0.0550	133.53358	38.03883	5.4395
2	9.393	BB	0.0538	2321.35132	680.88263	94.5605

Totals : 2454.88490 718.92146

==

*** End of Report ***

Supplementary Figure 27. LCMS of compound S8

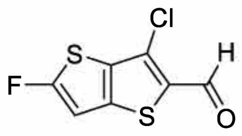

S8

MS Ionization : ESI
Instrument & Column: LCMS-AH(4-302) Chromolith. Flash RP-18e 25-2mm

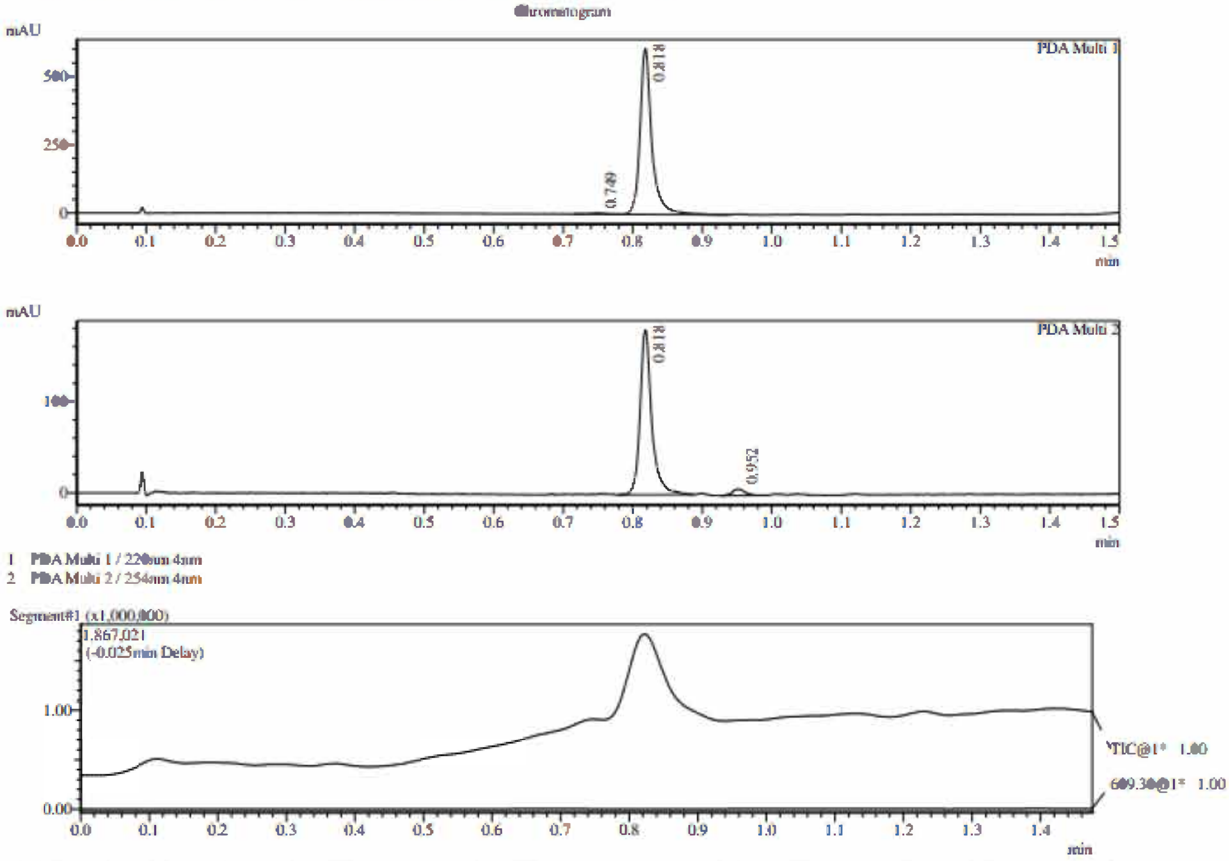

==

Integration Result

==

Ch1 220nm 4nm

Peak#	Ret. Time	Height	Height %	USP Width	Area	Area %
1	0.749	3232	0.538	0.030	4932	0.683
2	0.818	597822	99.462	0.028	717323	99.317

Ch2 254nm 4nm

Peak#	Ret. Time	Height	Height %	USP Width	Area	Area %
1	0.818	177973	96.210	0.028	210958	95.975
2	0.952	7011	3.790	0.030	8846	4.025

MS Spectrum Graph

RetTime: 0.817 DataFile: C:\DATA\1901\190115\00711155-1003-001A.lcd

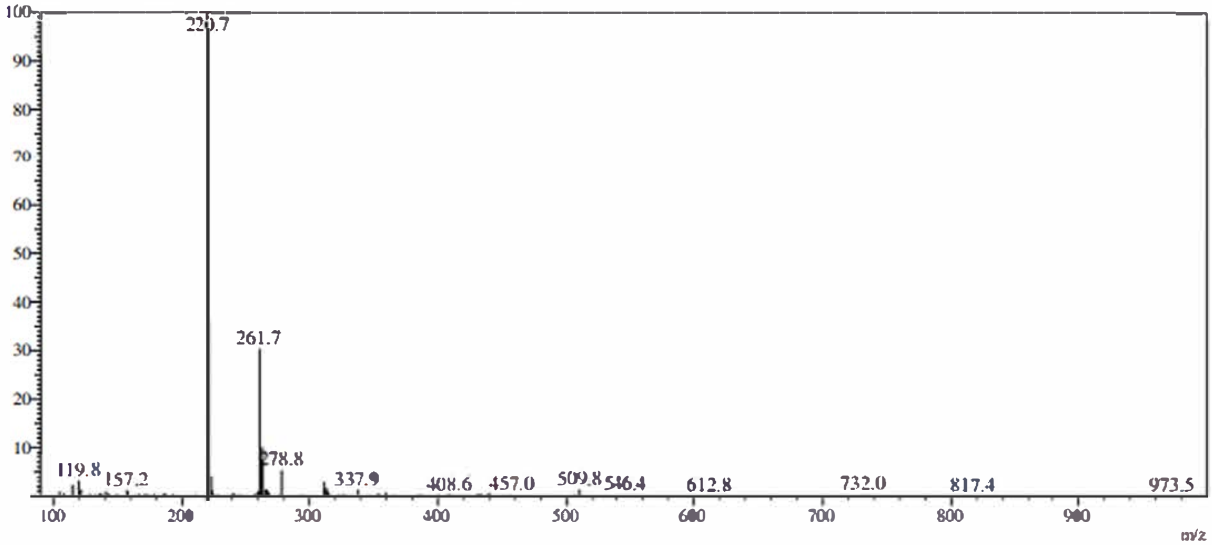

Supplementary Figure 28. 1H NMR of PF-07208254

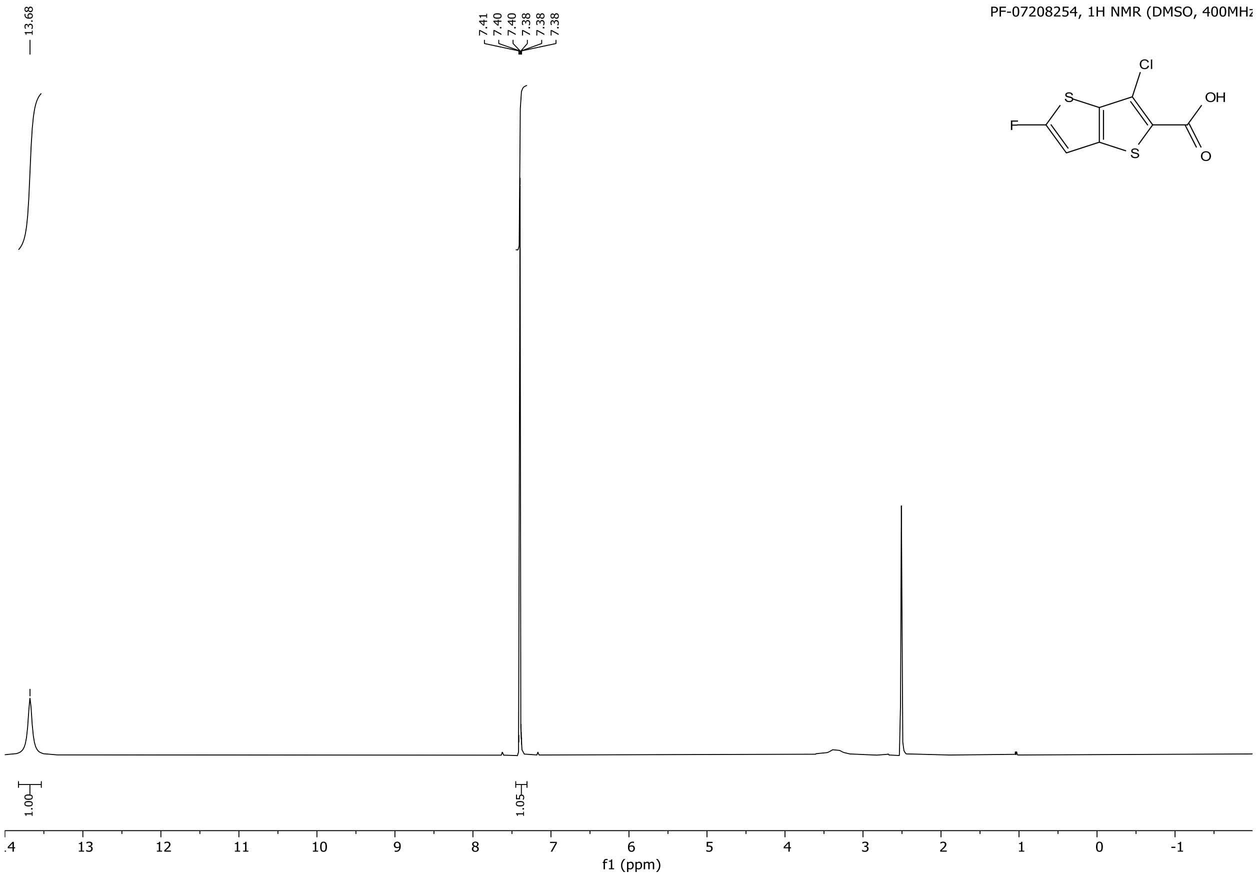

Supplementary Figure 29. 13C NMR of PF-07208254

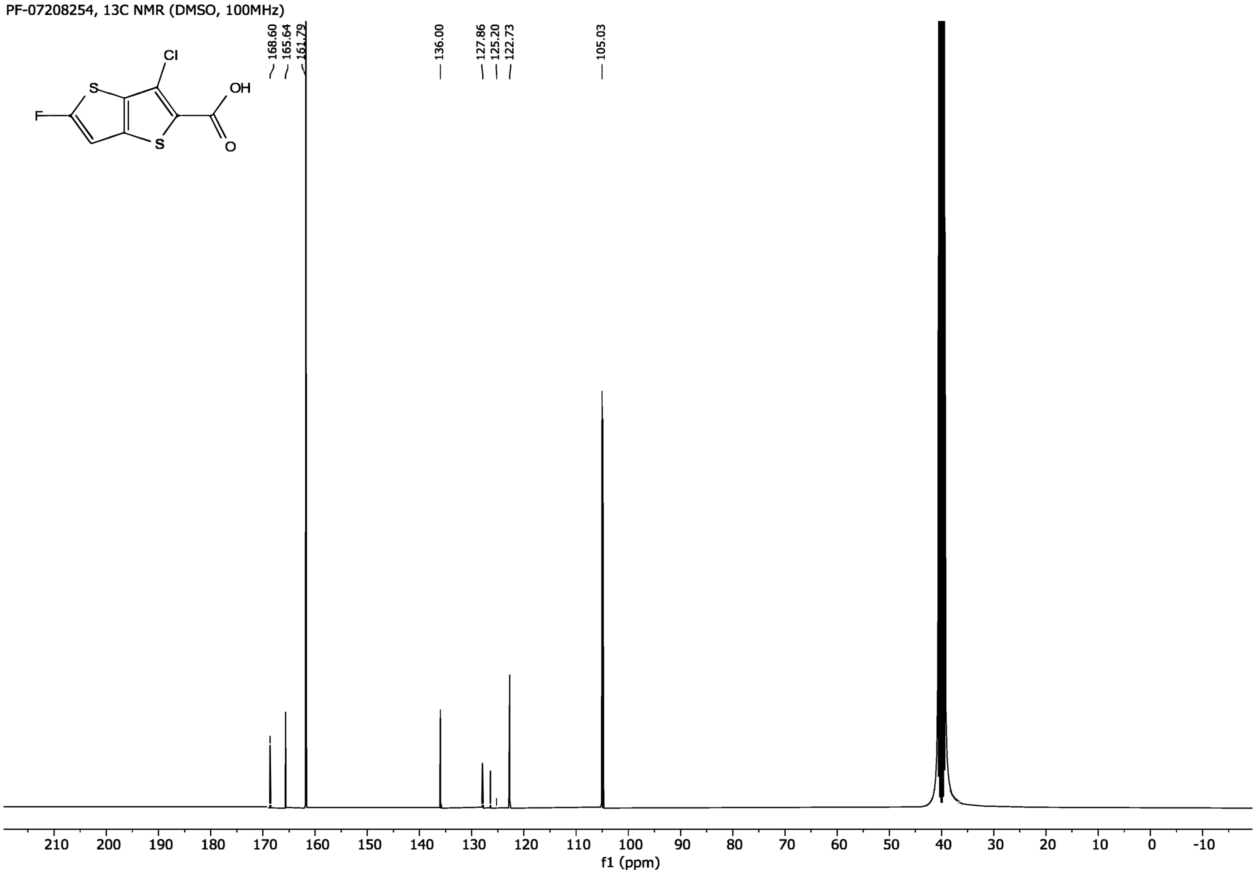

Supplementary Figure 30. 19F NMR of PF-07208254

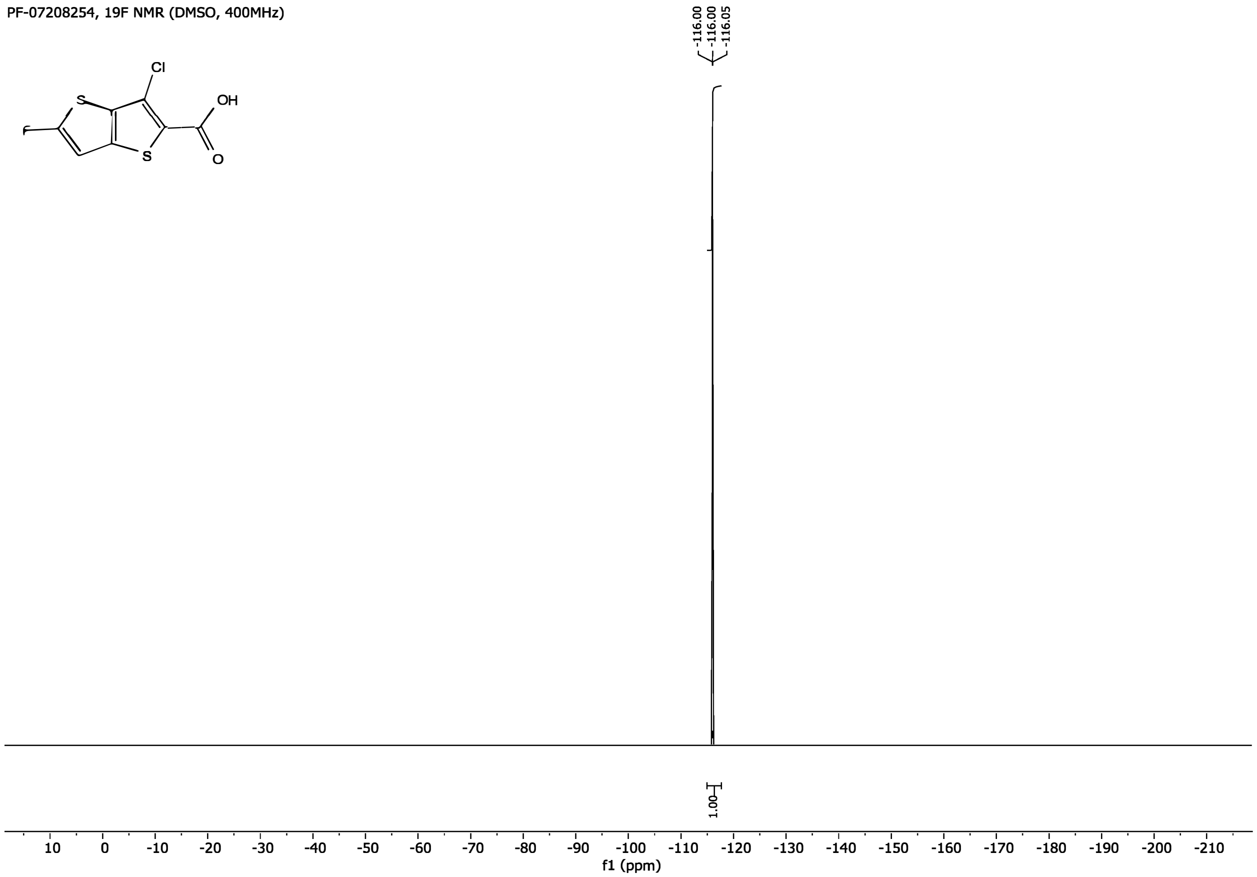

Supplementary Figure 31. HRMS of PF-07208254

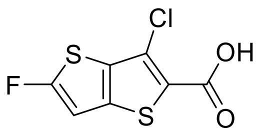

PF-07208254

Open Access HRMS Sample Report			
**Sample ID**	00110407-5465-001	**HPLC**	Agilent 1200
**Molecular Formula**	C7H2ClFO2S2	**Mass Spectrometer**	Sciex 5600+ QToF
**Submittor**	Martinez Alsina, Luis A	**DAD**	190-400 nm, reported 270nm
**Run Date**	4/4/2023	**MS Scan**	100-2000 amu
**Analyst**	Quinn, Alandra	**Acquisition Method**	HRMS-3min gradient AQ
**Analyst's CeN**	00714673-0163	**eWB Archive Ref**	00713459-0292

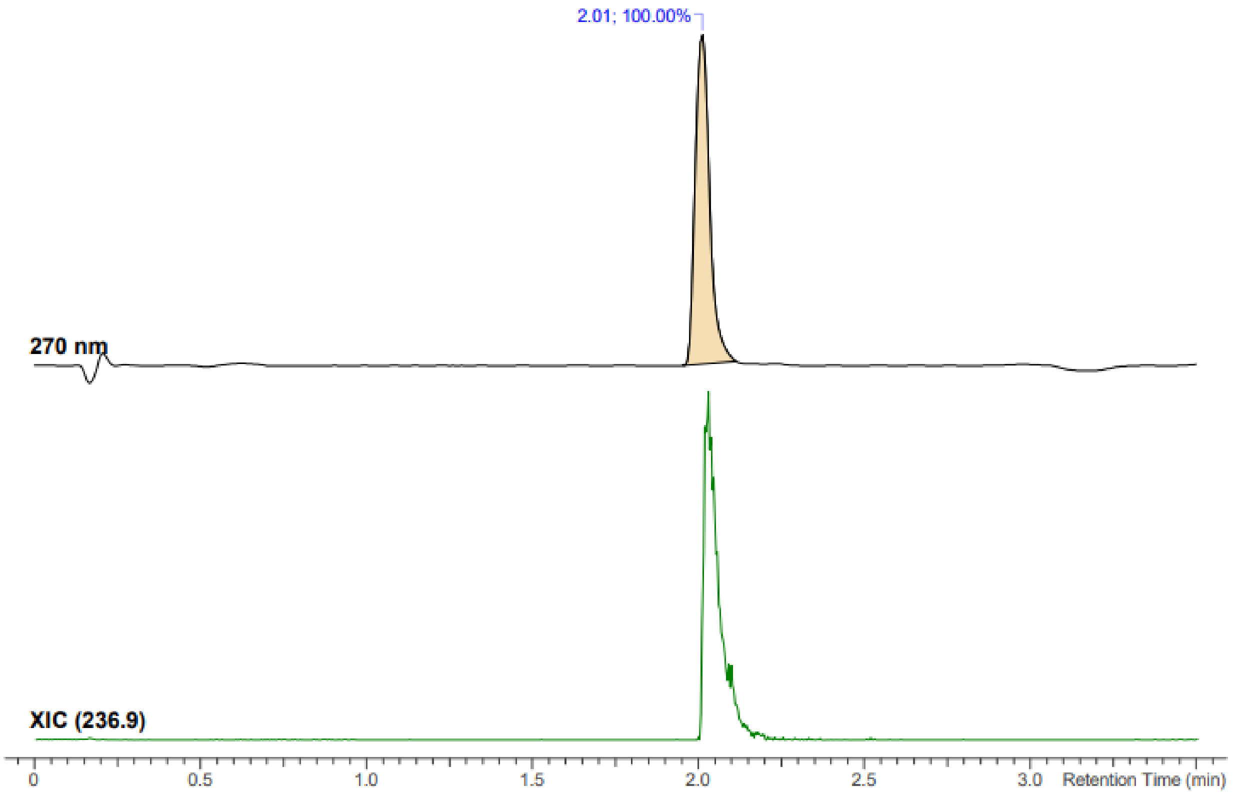

No.	Peak Name	tR (min)	Area (%)
1	C7H2ClFO2S2	2.01	100.00

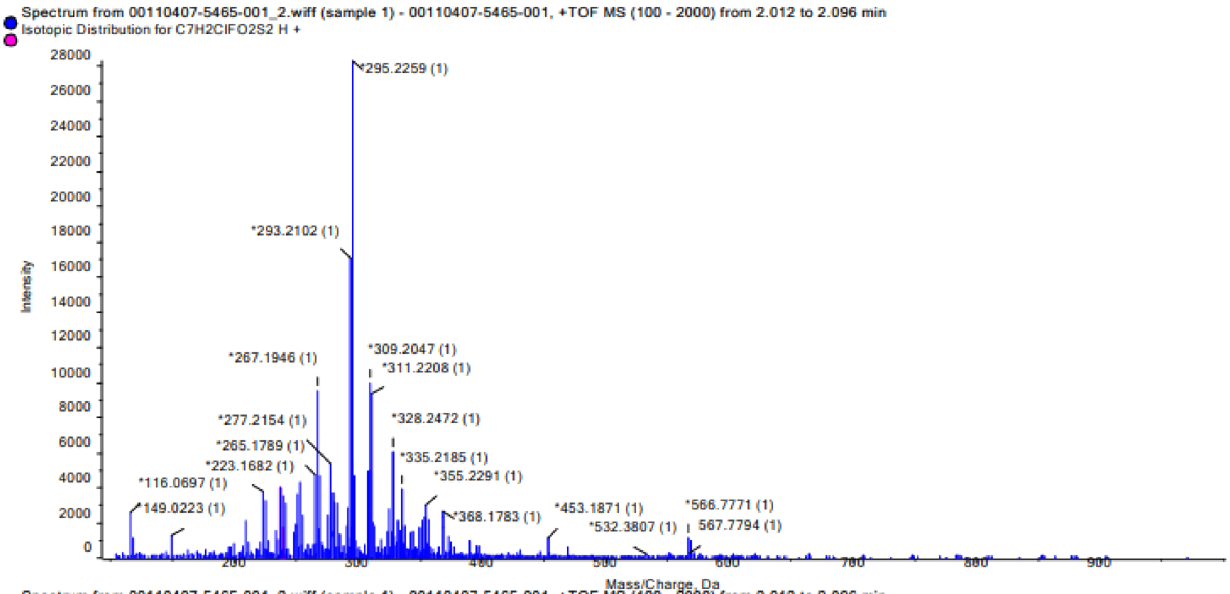

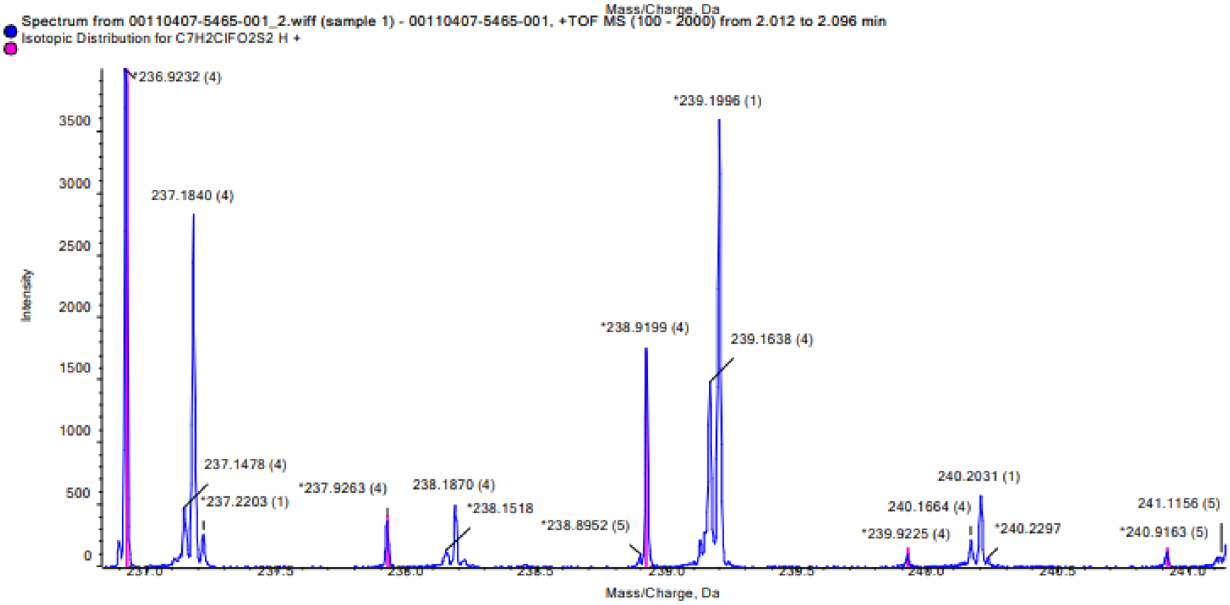

Peak at 2.01 min in UV								
Mass/Charge (Da)	Height	Relative % Height	Compound	Peak Type	Theoretical m/z	Error (ppm)	Theoretical m/z	Theoretical Intensity
236.9232	3998	100.0	C7H2ClFO2S2	[M+H]+	236.9242	-4.1	236.9242	100.0
237.9263	367	9.2					237.9269	9.5
238.9199	1760	44.0					238.9211	41.6
239.9225	99	2.5					239.9238	3.8
240.9163	116	2.9					240.9178	3.4

Supplementary Figure 32. HPLC of PF-07208254

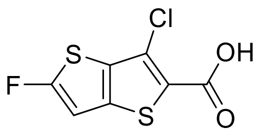

PF-07208254

connecting discovery to pharm sci

SAMPLE INFORMATION

Sample Name:	PF-07208254-00-0026	Acquired By:	LiC03
		Date Acquired:	14-Feb-20 12:40:27 PM EST
Vial:	1:A,2	Acq. Method Set:	GRO343M3_10pt3_MSA_RP18
Injection #:	1	Date Processed:	14-Feb-20 1:40:28 PM EST
Injection Volume:	1.00 ul	Processing Method:	GRO343_UPLC_PM
Run Time:	10.3 Minutes	Channel Name:	PDA 210 nm
Sample Set Name:	GRO343_CL_ETS 02142020 Run1		

Auto-Scaled Chromatogram

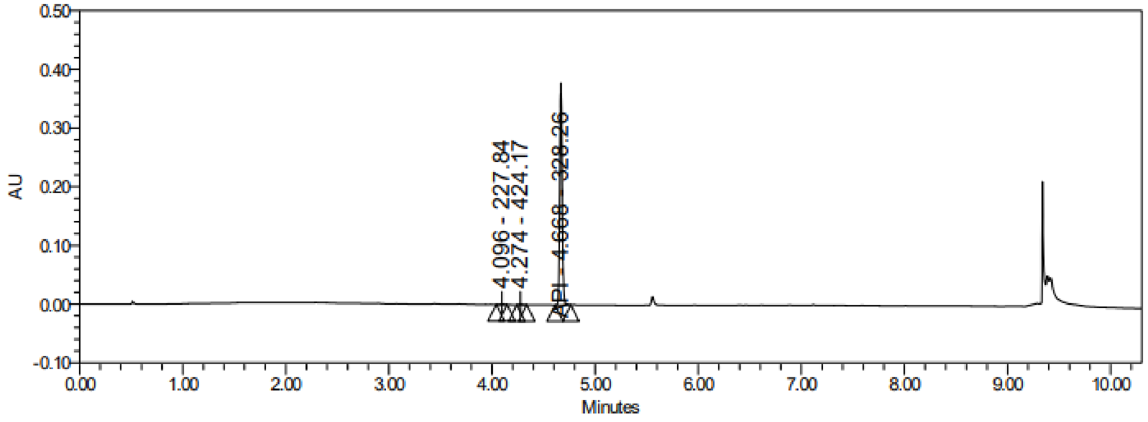

Sample Name PF-07208254-00-0026; Vial 1:A,2; Injection 1; Channel PDA Ch1 210nm@4.8nm; Date Acquired 14-Feb-20 12:40:27 PM EST

Zoomed Chromatogram

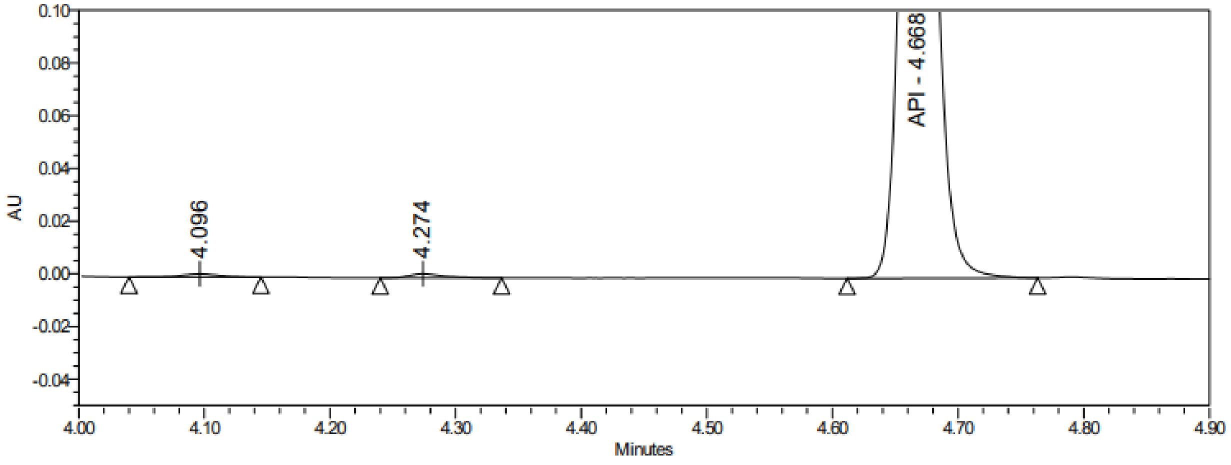

SAMPLE INFORMATION

Sample Name: PF-07208254-00-0026

Vial: 1:A,2

Injection #: 1

Injection Volume: 1.00 ul

Run Time: 10.3 Minutes

Sample Set Name: GRO343_CL_ETS 02142020 Run1

Acquired By: LiC03

Date Acquired: 14-Feb-20 12:40:27 PM EST

Acq. Method Set: GRO343M3_10pt3_MSA_RP18

Date Processed: 14-Feb-20 1:40:28 PM EST

Processing Method GRO343_UPLC_PM

Channel Name: PDA 210 nm

Area % Results

Result Id 95855

Peak Results

	Peak Name	RT	Area	% Area	Height	USP Tailing	s/n	USP s/n	USP Resolution
1		4.096	2416	0.41	1201				
2		4.274	2938	0.50	1560				
3	API	4.668	582040	99.09	360389				

Supplementary Figure 33. 1H NMR of compound S9

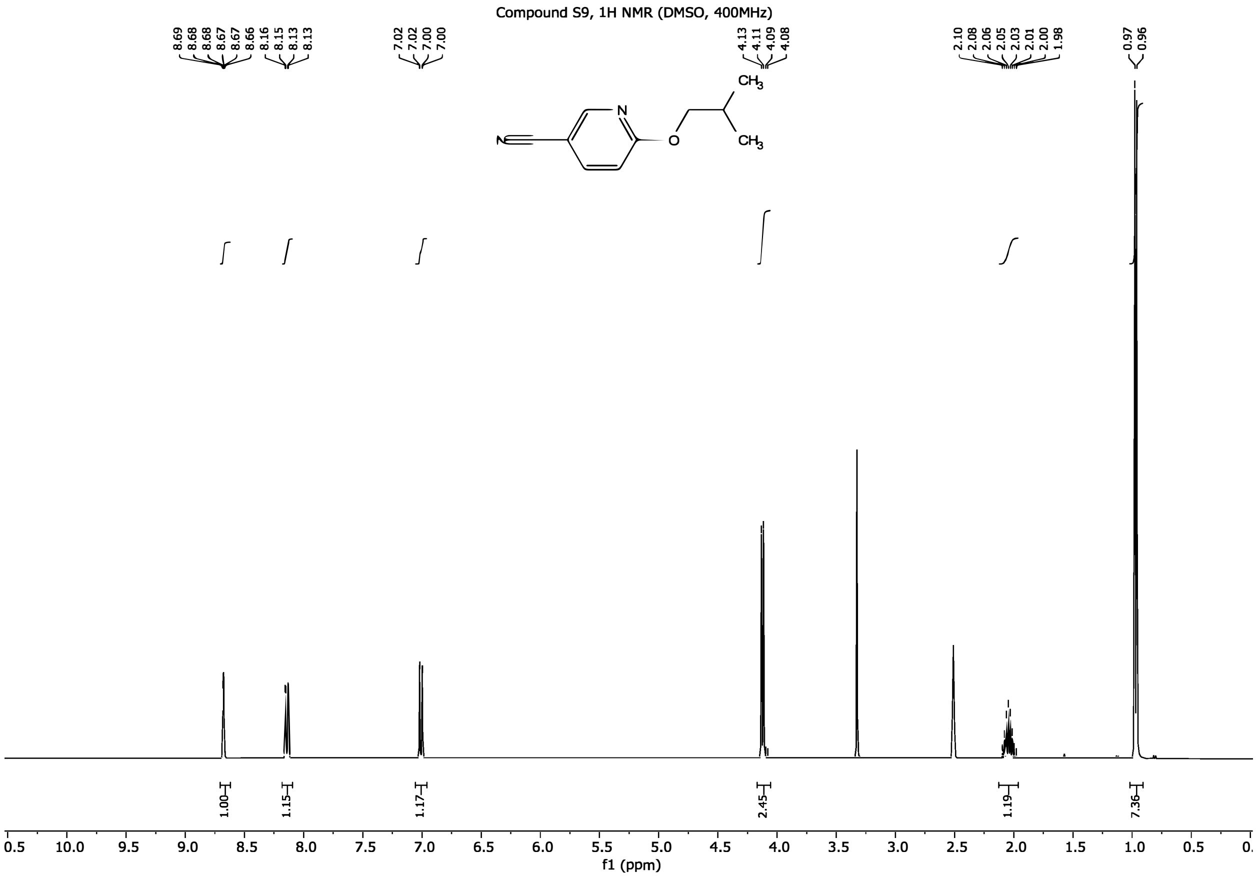

Supplementary Figure 34. 13C NMR of compound S9

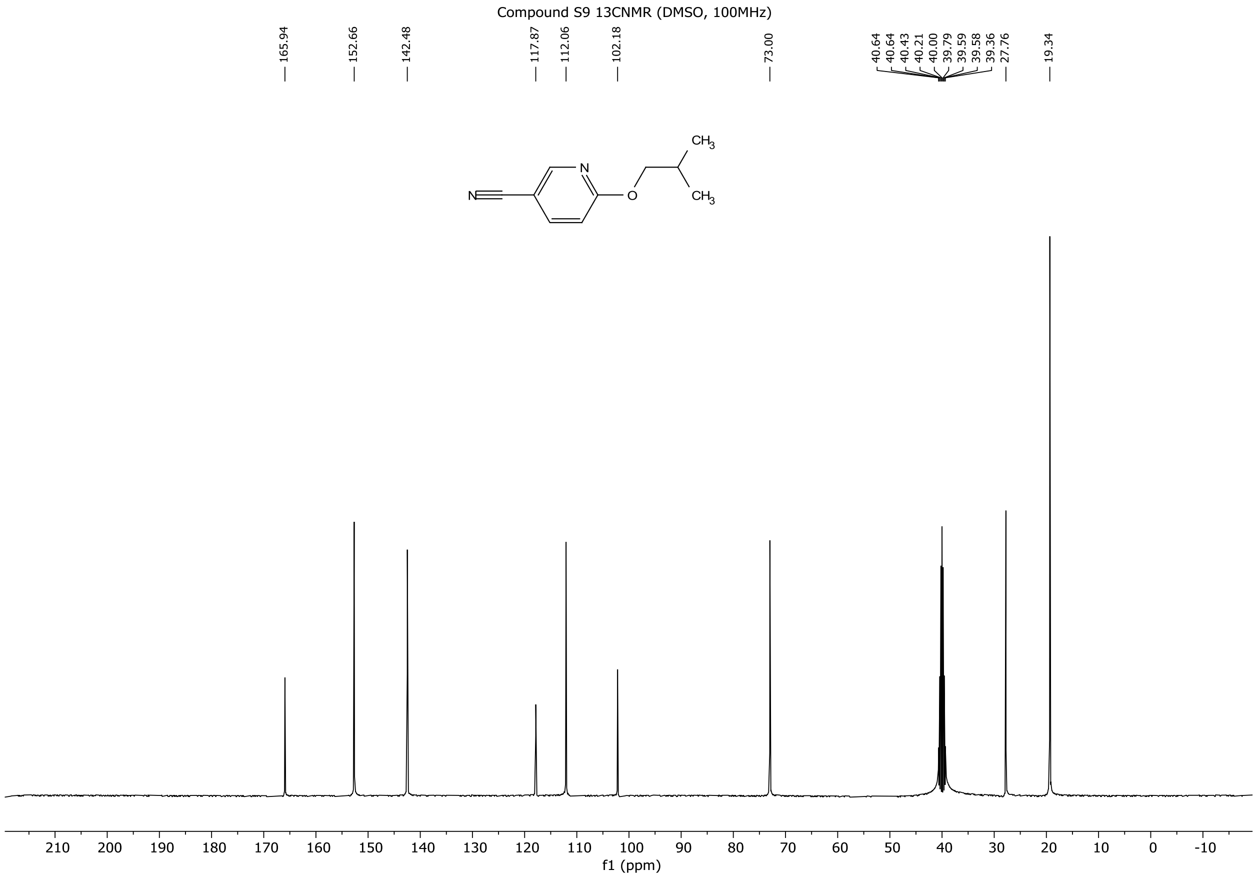

Supplementary Figure 35. HRMS of compound S9

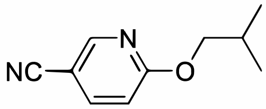

S9

Open Access HRMS Sample Report			
Sample ID	00110580-3414-006	HPLC	Agilent 1200
Molecular Formula	C10H12N2O	Mass Spectrometer	Sciex 5600+ QToF
Submittor	Martinez, Luis	DAD	190-400 nm, reported 270nm
Run Date	4/27/2023	MS Scan	100-2000 amu
Analyst	Quinn, Alandra	Acquisition Method	HRMS-3min gradient AQ
Analyst's CeN	00714673-0166	eWB Archive Ref	00713459-0294

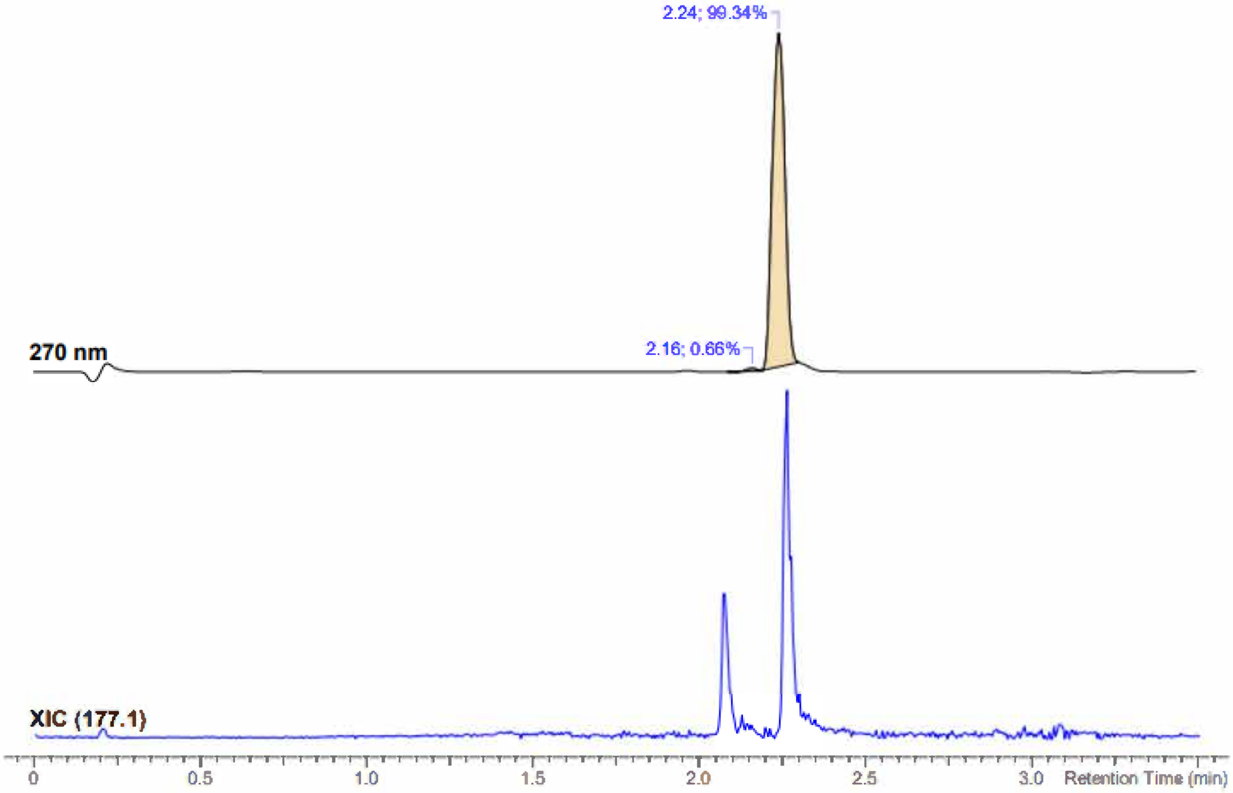

No.	Peak Name	tR (min)	Area (%)
1	unknown	2.16	0.66
2	C10H12N2O	2.24	99.34

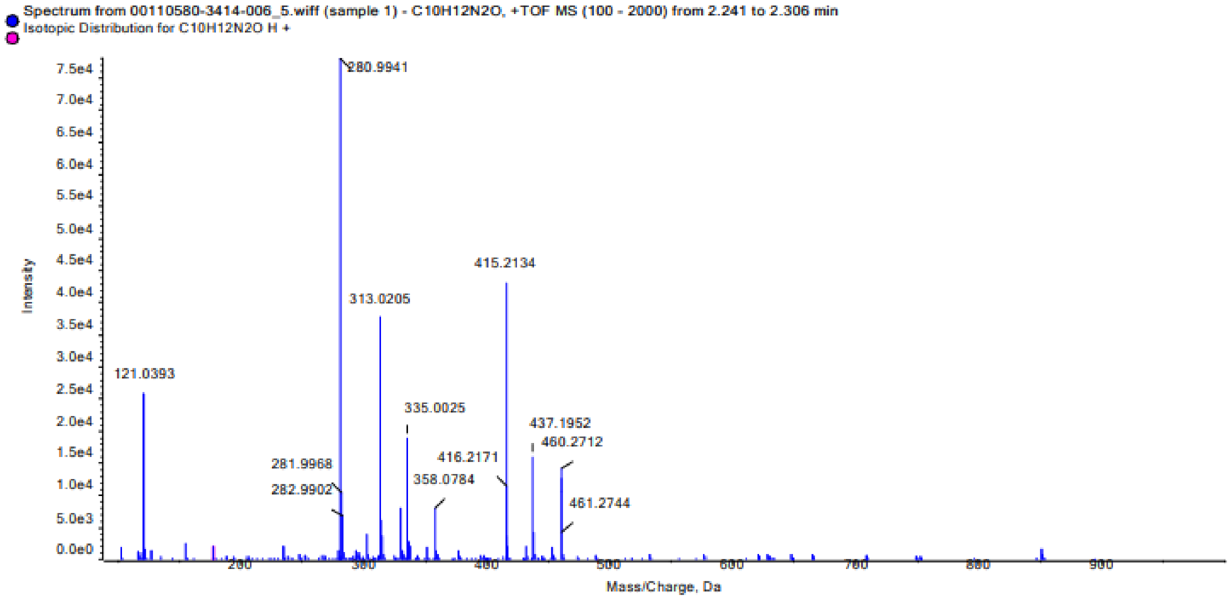

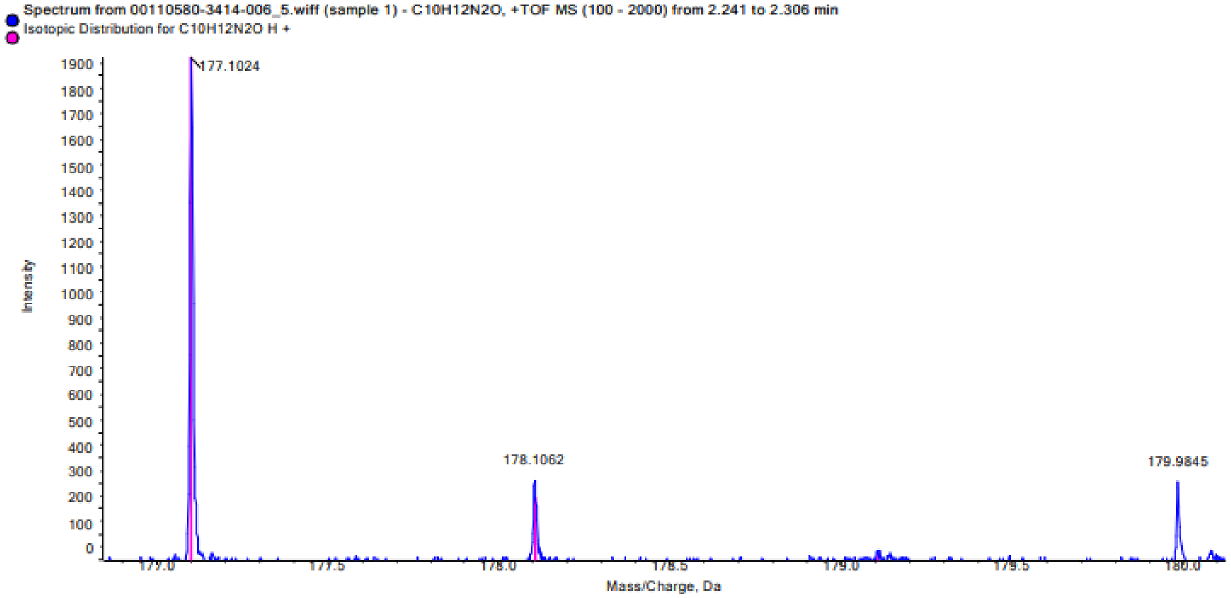

Peak at 2.24 min in UV								
Mass/Charge (Da)	Height	Relative % Height	Compound	Peak Type	Theoretical m/z	Error (ppm)	Theoretical m/z	Theoretical Intensity
177.1024	1969	100.0	C10H12N2O	[M+H]+	177.1022	0.8	177.1022	100.0
178.1062	310	15.8					178.1052	12.0
179.1076	39	2.0					179.1078	0.9

Supplementary Figure 36. HPLC of compound S9

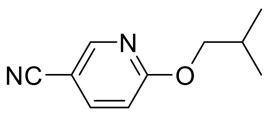

S9

```
=====================================================================
Acq. Operator   : SYSTEM                        Seq. Line :    1
Sample Operator : SYSTEM
Acq. Instrument : HPLC-233GA                     Location :    6
Injection Date  : 4/27/2023 12:34:22 PM               Inj :    1
                                               Inj Volume : 10.000 µl
Acq. Method     : C:\Users\Public\Documents\ChemStation\1\Data\def_LC_2021 2023-04-27 12-29-
                  18\ACID_GRADIENT.M
Last changed    : 6/9/2021 9:16:24 AM by SYSTEM
Analysis Method : C:\Users\Public\Documents\ChemStation\1\Data\def_LC_2021 2023-04-27 12-29-
                  18\ACID_GRADIENT.M (Sequence Method)
Last changed    : 4/28/2023 2:01:26 PM by SYSTEM
                  (modified after loading)
Method Info     : Column: XBridge C18 5 micron (4.6 mm x 150 mm)
                  Flow rate: 1.500 mL/min with solvents containing 0.1% TFA
                  0-1.5 min: 5% acetonitrile/water
                  1.5-10 min: 5-100% acetonitrile water
                  10-11 min: 100% acetonitrile
                  11-12.5 min: 100-5% acetonitrile/water
```

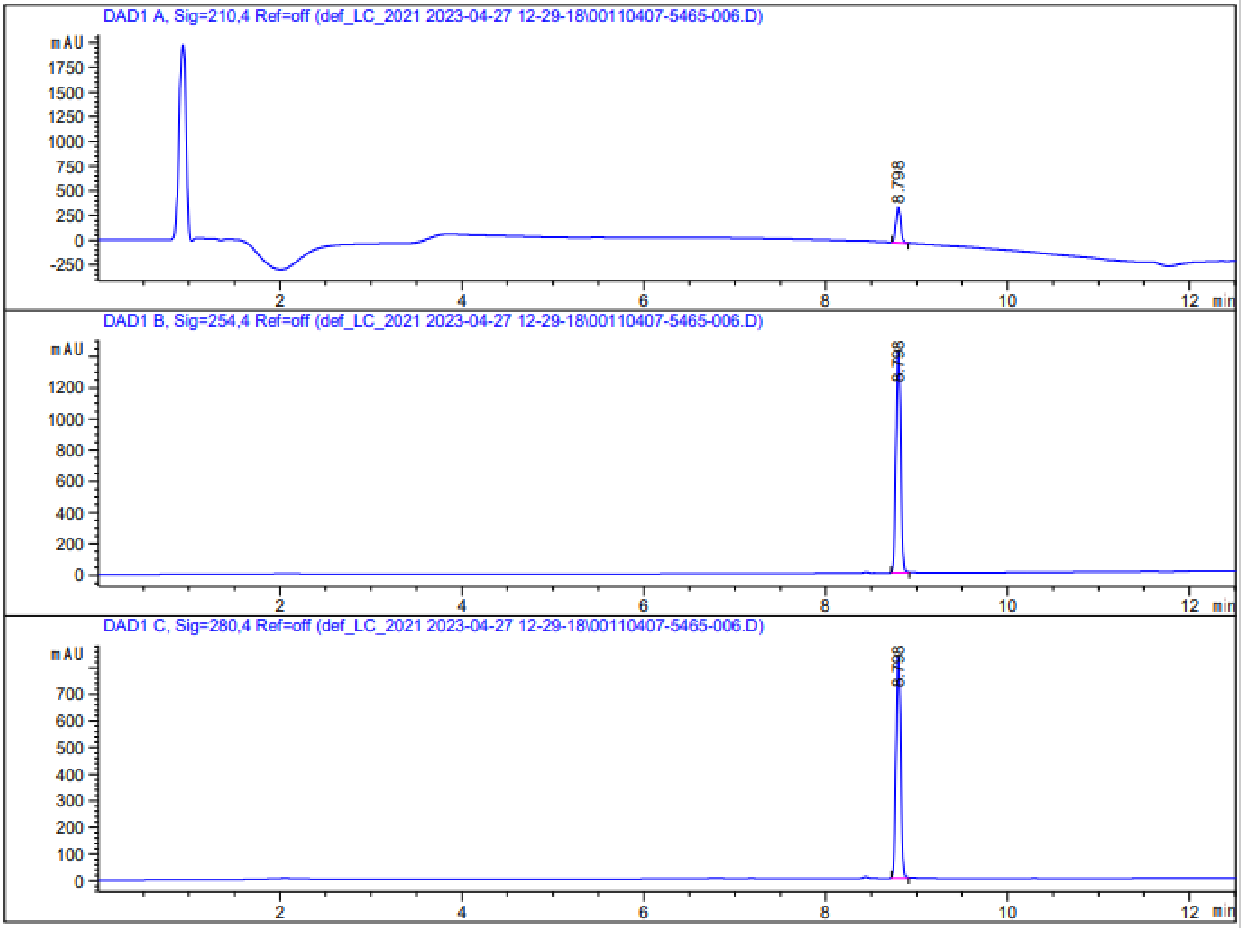

Area Percent Report

Sorted By : Signal
Multiplier : 1.0000
Dilution : 1.0000
Do not use Multiplier & Dilution Factor with ISTDs

Signal 1: DAD1 A, Sig=210,4 Ref=off

Peak #	RetTime [min]	Type	Width [min]	Area [mAU*s]	Height [mAU]	Area %
1	8.798	BB	0.0561	1291.38745	357.88132	100.0000
Totals :				1291.38745	357.88132	

Signal 2: DAD1 B, Sig=254,4 Ref=off

Peak #	RetTime [min]	Type	Width [min]	Area [mAU*s]	Height [mAU]	Area %
1	8.798	BB	0.0558	5101.04199	1424.99304	100.0000
Totals :				5101.04199	1424.99304	

Signal 3: DAD1 C, Sig=280,4 Ref=off

Peak #	RetTime [min]	Type	Width [min]	Area [mAU*s]	Height [mAU]	Area %
1	8.798	BB	0.0553	2957.12744	835.37280	100.0000
Totals :				2957.12744	835.37280	

*** End of Report ***

Supplementary Figure 37. 1H NMR of compound S10

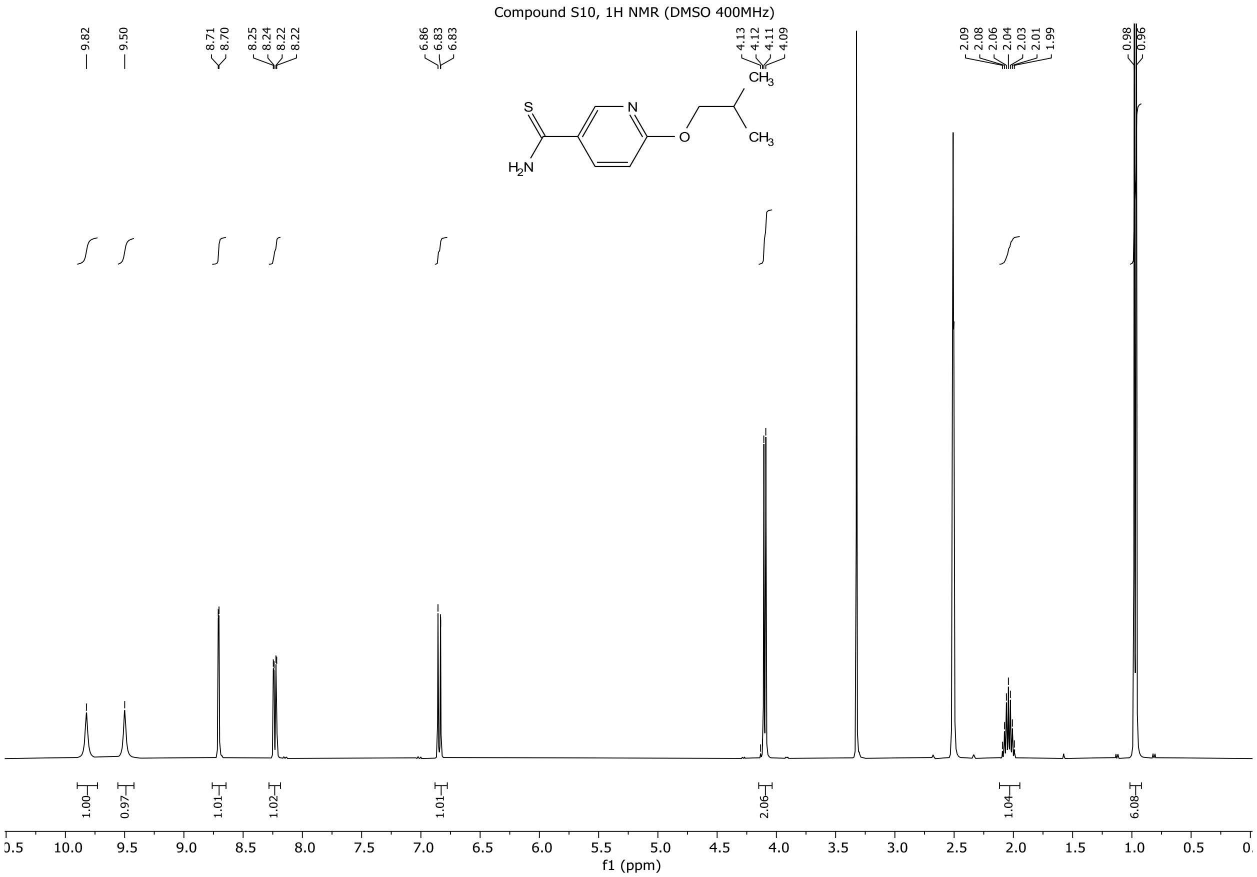

Supplementary Figure 38. 13C NMR of compound S10

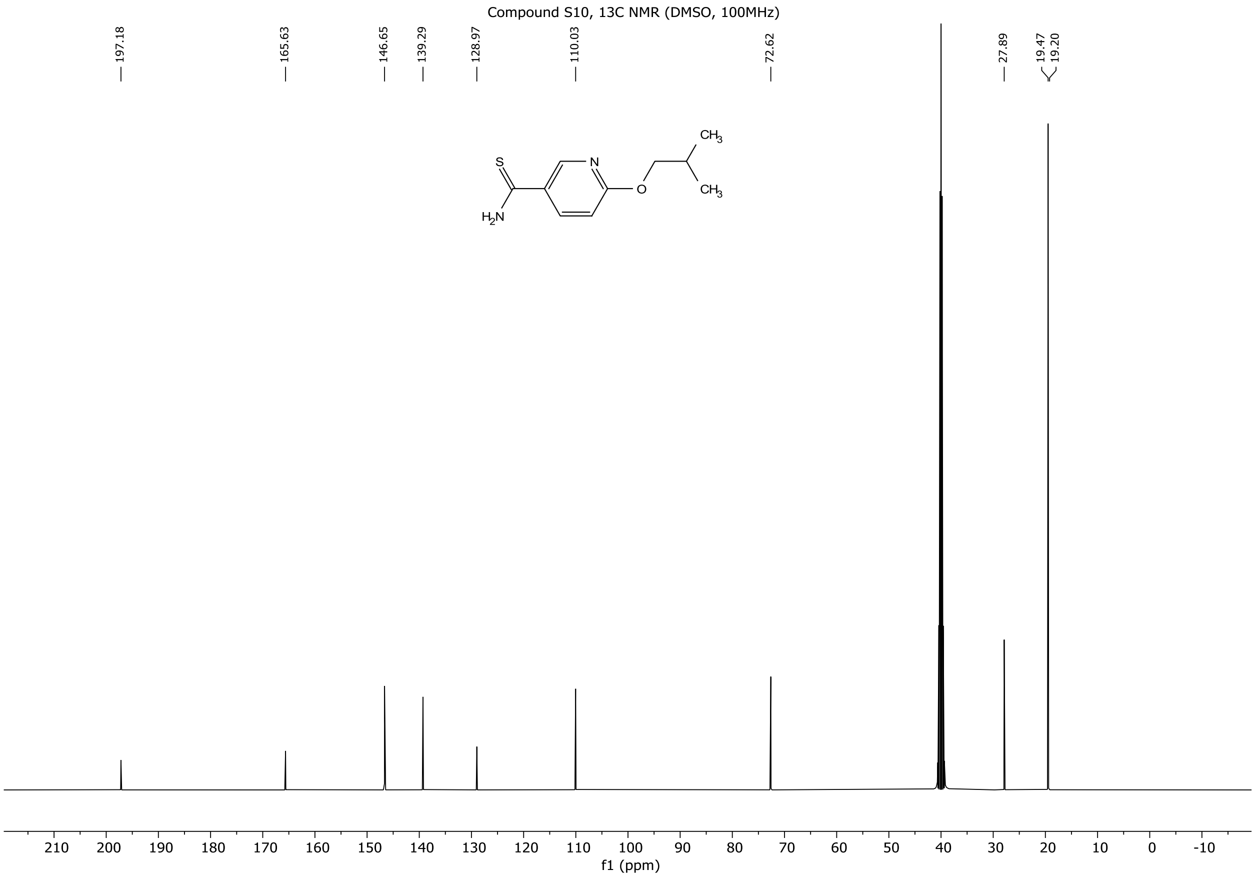

Supplementary Figure 39. HRMS of compound S10

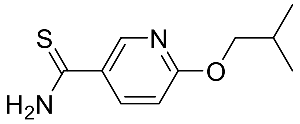

S10

Open Access HRMS Sample Report			
**Sample ID**	00110512-3609-003	**HPLC**	Agilent 1200
**Molecular Formula**	C10H14N2OS	**Mass Spectrometer**	Sciex 5600+ QToF
**Submittor**	Martinez, Luis	**DAD**	190-400 nm, reported 270nm
**Run Date**	4/27/2023	**MS Scan**	100-2000 amu
**Analyst**	Quinn, Alandra	**Acquisition Method**	HRMS-3min gradient AQ
**Analyst's CeN**	00714673-0166	**eWB Archive Ref**	00713459-0294

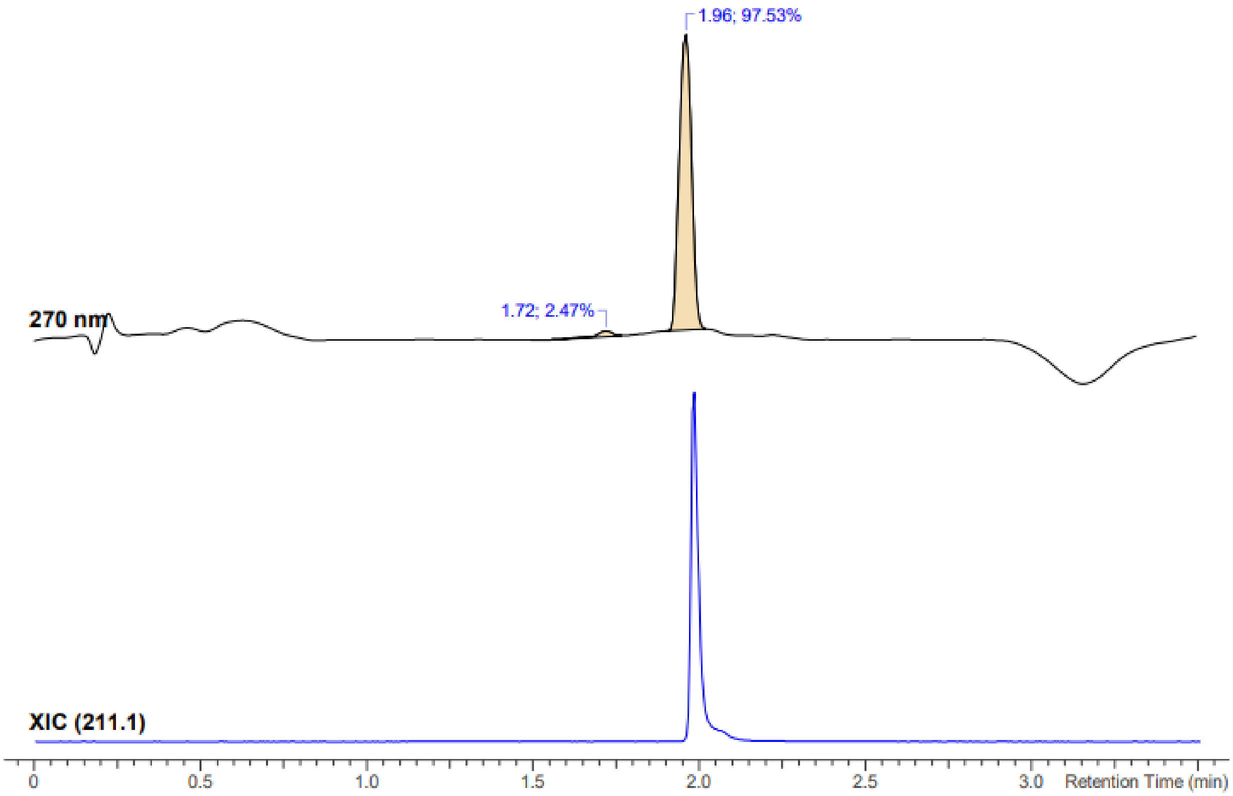

No.	Peak Name	tR (min)	Area (%)
1	unknown	1.72	2.47
2	C10H14N2OS	1.96	97.53

● **Spectrum from 00110512-3609-003.wiff (sample 1) - C10H14N2OS, +TOF MS (100 - 2000) from 1.934 to 2.027 min**
● Isotopic Distribution for C10H14N2OS H +

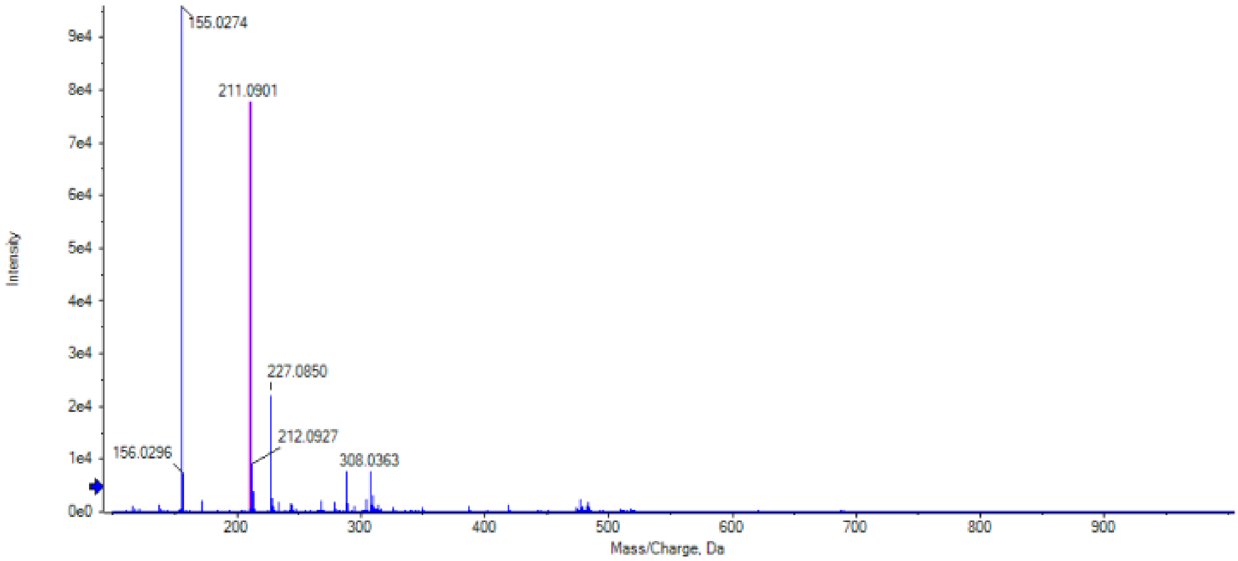

● **Spectrum from 00110512-3609-003.wiff (sample 1) - C10H14N2OS, +TOF MS (100 - 2000) from 1.934 to 2.027 min**
● Isotopic Distribution for C10H14N2OS H +

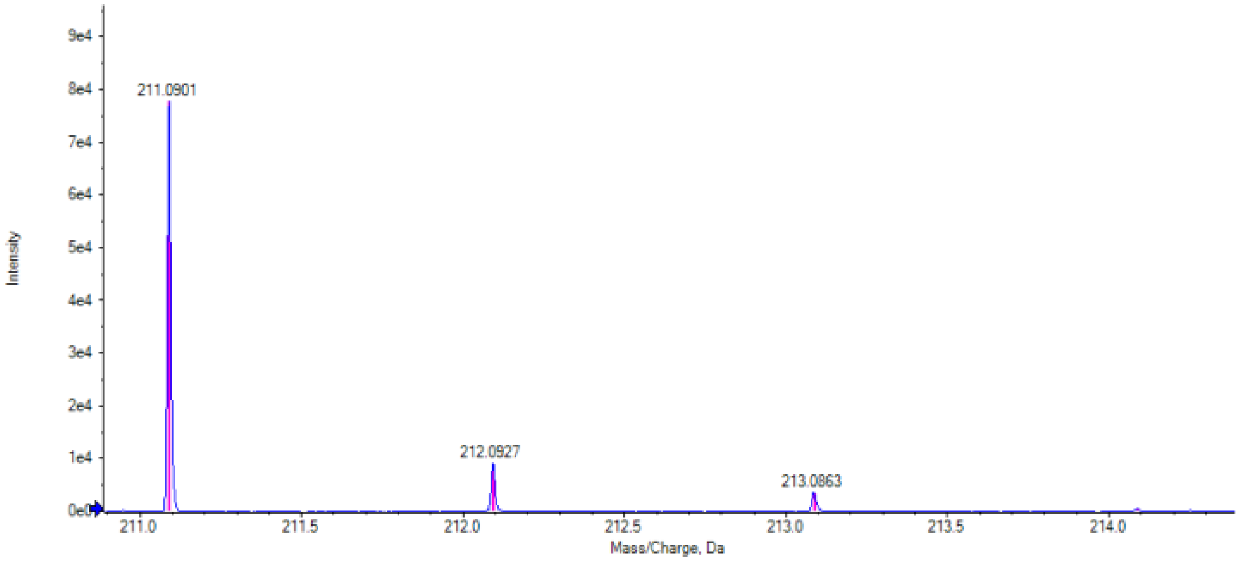

Peak at 1.96 min in UV								
Mass/Charge (Da)	Height	Relative % Height	Compound	Peak Type	Theoretical m/z	Error (ppm)	Theoretical m/z	Theoretical Intensity
211.0901	77845	100.0	C10H14N2OS	[M+H]+	211.0900	0.5	211.0900	100.0
212.0927	9088	11.7					212.0927	12.8
213.0863	3722	4.8					213.0874	5.4
214.0889	532	0.7					214.0896	0.6

Supplementary Figure 40. HPLC of compound S10

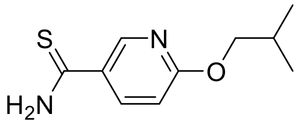

S10

```
=====================================================================
Acq. Operator   : SYSTEM                        Seq. Line :   7
Sample Operator : SYSTEM
Acq. Instrument : HPLC-233GA                     Location :   12
Injection Date  : 4/27/2023 1:59:16 PM                Inj :   1
                                               Inj Volume : 10.000 µl
Method          : C:\Users\Public\Documents\ChemStation\1\Data\def_LC_2021 2023-04-27 12-29-
                  18\ACID_GRADIENT.M (Sequence Method)
Last changed    : 6/9/2021 9:16:24 AM by SYSTEM
Method Info     : Column: XBridge C18 5 micron (4.6 mm x 150 mm)
                  Flow rate: 1.500 mL/min with solvents containing 0.1% TFA
                  0-1.5 min: 5% acetonitrile/water
                  1.5-10 min: 5-100% acetonitrile water
                  10-11 min: 100% acetonitrile
                  11-12.5 min: 100-5% acetonitrile/water
```

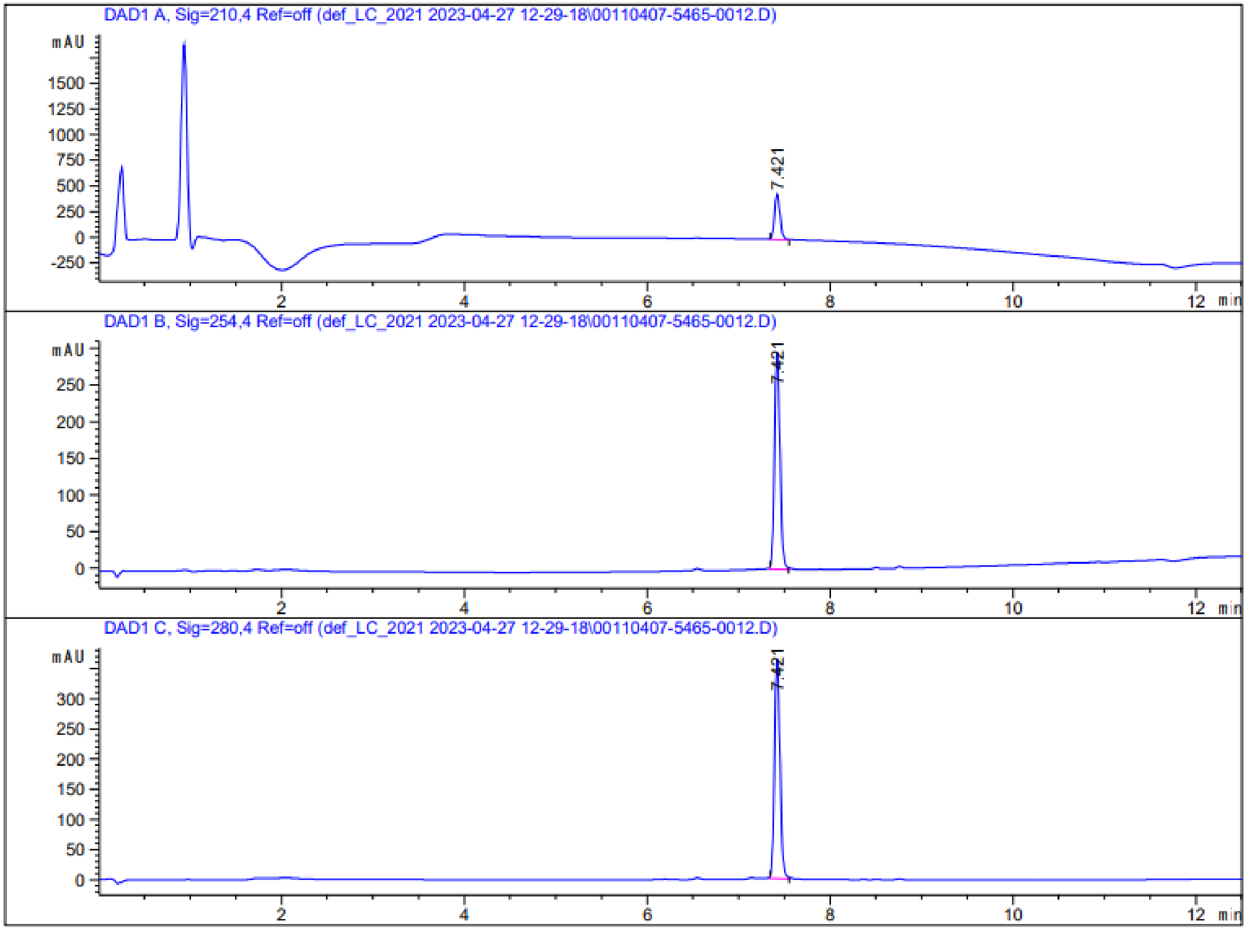

===

Area Percent Report

===

Sorted By : Signal
Multiplier : 1.0000
Dilution : 1.0000
Do not use Multiplier & Dilution Factor with ISTDs

Signal 1: DAD1 A, Sig=210,4 Ref=off

Peak #	RetTime [min]	Type	Width [min]	Area [mAU*s]	Height [mAU]	Area %
1	7.421	BB	0.0652	1861.47400	441.43124	100.0000

Totals : 1861.47400 441.43124

Signal 2: DAD1 B, Sig=254,4 Ref=off

Peak #	RetTime [min]	Type	Width [min]	Area [mAU*s]	Height [mAU]	Area %
1	7.421	BB	0.0650	1249.24487	297.22464	100.0000

Totals : 1249.24487 297.22464

Signal 3: DAD1 C, Sig=280,4 Ref=off

Peak #	RetTime [min]	Type	Width [min]	Area [mAU*s]	Height [mAU]	Area %
1	7.421	BB	0.0650	1533.97607	365.13541	100.0000

Totals : 1533.97607 365.13541

===

*** End of Report ***

Supplementary Figure 41. 1H NMR of compound S11

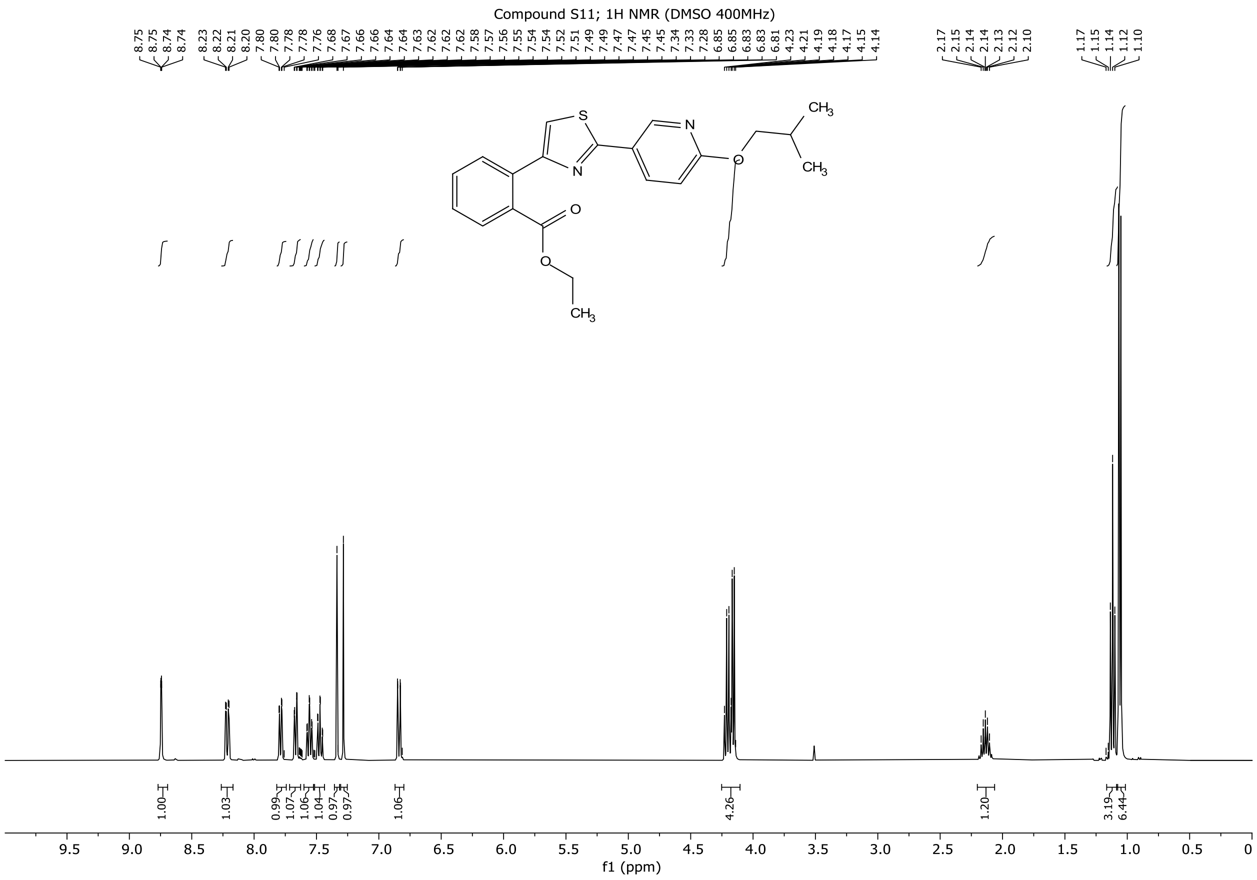

Supplementary Figure 42. HPLC of compound S11

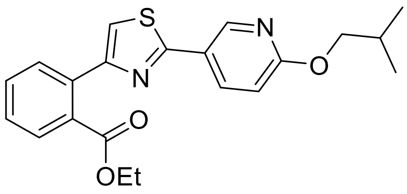

S11

```
=====================================================================
Acq. Operator   : SYSTEM                         Seq. Line :    3
Acq. Instrument : LC-235GC                        Location :    2
Injection Date  : 3/25/2019 6:52:20 AM                 Inj :    1
                                                Inj Volume : 5.0 µl
Different Inj Volume from Sample Entry!  Actual Inj Volume : 10.0 µl
Sequence File   : C:\Chem32\1\Data\THERMO\TEST 2019-03-25 06-23-17\TEST.S
Method          : C:\Chem32\1\Data\THERMO\TEST 2019-03-25 06-23-17\ACID_CLASSIC.M (Sequence
                  Method)
Last changed    : 3/25/2019 6:23:18 AM by SYSTEM
Method Info     : ACID METHOD for classic look on new Chemstation software
                  Column: XBridge C18 5 micron (4.6 mm x 150 mm)
                  Flow rate: 1.500 mL/min with solvents containing 0.1% TFA
                  0-1.5 min: 5% acetonitrile/water
                  1.5-10 min: 5-100% acetonitrile water
                  10-11 min: 100% acetonitrile
                  11-12.5 min: 100-5% acetonitrile/water

Sample Info     : 00110512-3562-004
```

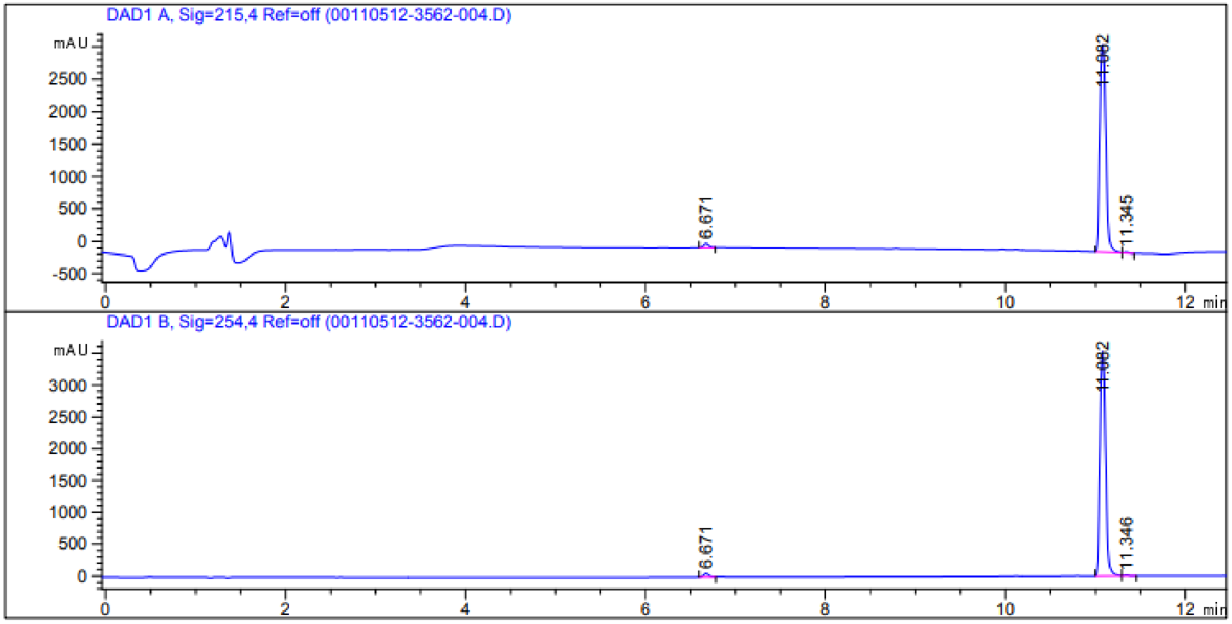


```
=====================================================================
                         Area Percent Report
=====================================================================

Sorted By             :      Signal
Multiplier            :      1.0000
Dilution              :      1.0000
Do not use Multiplier & Dilution Factor with ISTDs
```

Signal 1: DAD1 A, Sig=215,4 Ref=off

Peak #	RetTime [min]	Type	Width [min]	Area [mAU*s]	Height [mAU]	Area %
1	6.671	BB	0.0644	274.95190	63.60682	1.8365
2	11.082	BV	0.0732	1.46410e4	3209.51489	97.7920
3	11.345	VB	0.0533	55.62463	16.49937	0.3715

Totals : 1.49716e4 3289.62108

Signal 2: DAD1 B, Sig=254,4 Ref=off

Peak #	RetTime [min]	Type	Width [min]	Area [mAU*s]	Height [mAU]	Area %
1	6.671	BB	0.0644	252.87498	58.50365	1.6504
2	11.082	BB	0.0696	1.50151e4	3532.13159	97.9943
3	11.346	BB	0.0574	54.44085	14.63323	0.3553

Totals : 1.53225e4 3605.26847

==

*** End of Report ***

Supplementary Figure 43. LCMS of compound S11

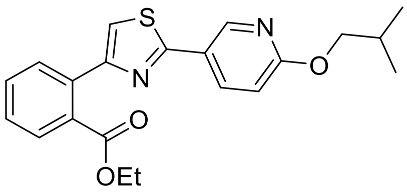

S11

MS Ionization : ESI
Instrument & Column: LCMS-AO(4-302) Chromolith. Flash RP-18e 25-2mm

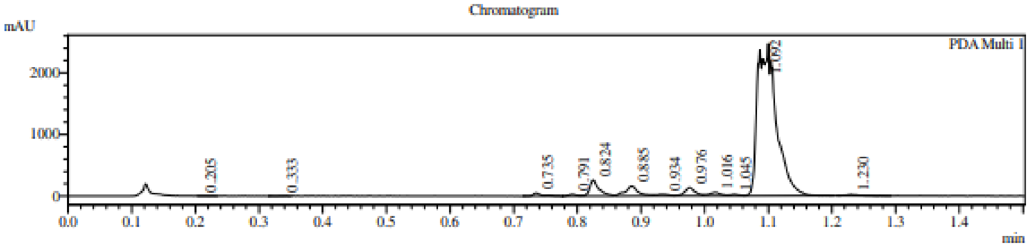

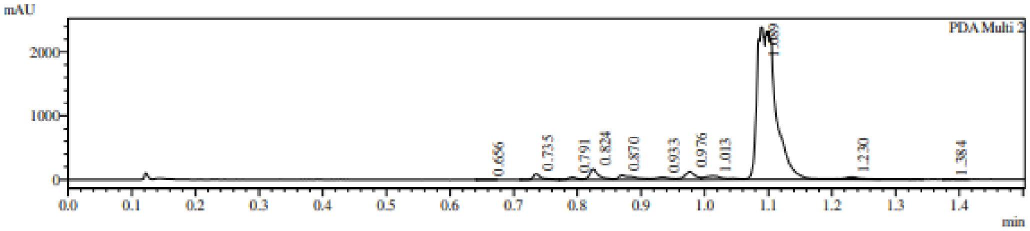

1 PDA Multi 1 / 220nm 4nm
2 PDA Multi 2 / 254nm 4nm

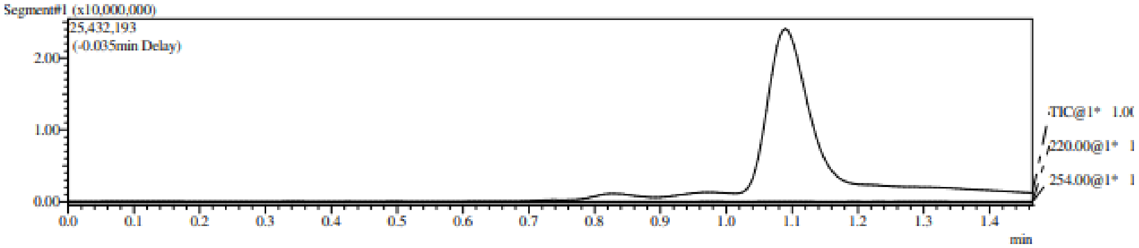

==

Integration Result

==

PDA Ch1 220nm 4nm

Peak#	Ret. Time	Height	Height %	USP Width	Area	Area %
1	0.205	2209	0.075	0.011	3089	0.052
2	0.333	2421	0.083	0.056	4759	0.080
3	0.735	51657	1.764	0.021	51542	0.864
4	0.791	30427	1.039	0.026	27472	0.461
5	0.824	251295	8.582	0.027	268431	4.501
6	0.885	159999	5.464	0.033	226778	3.802
7	0.934	27653	0.944	0.050	37216	0.624
8	0.976	132772	4.534	0.031	154079	2.583
9	1.016	54021	1.845	0.038	71389	1.197
10	1.045	23064	0.788	0.025	24651	0.413
11	1.092	2174106	74.244	0.046	5066531	84.953
12	1.230	18691	0.638	0.037	28020	0.470

PDA Ch2 254nm 4nm

Peak#	Ret. Time	Height	Height %	USP Width	Area	Area %
1	0.656	2619	0.088	0.090	3035	0.054
2	0.735	90238	3.026	0.021	79163	1.399

Peak#	Ret. Time	Height	Height %	USP Width	Area	Area %
3	0.791	37896	1.271	0.026	37006	0.654
4	0.824	161148	5.403	0.025	163388	2.888
5	0.870	65664	2.202	0.048	108551	1.919
6	0.933	37046	1.242	0.041	58674	1.037
7	0.976	120552	4.042	0.031	139263	2.461
8	1.013	53342	1.789	0.056	111290	1.967
9	1.089	2380165	79.809	0.043	4911657	86.809
10	1.230	32100	1.076	0.034	42757	0.756
11	1.384	1570	0.053	0.022	3229	0.057

RetTime: 1.090 DataFile: D:\DATA\1903\190328\00708262-1371-001.lcd

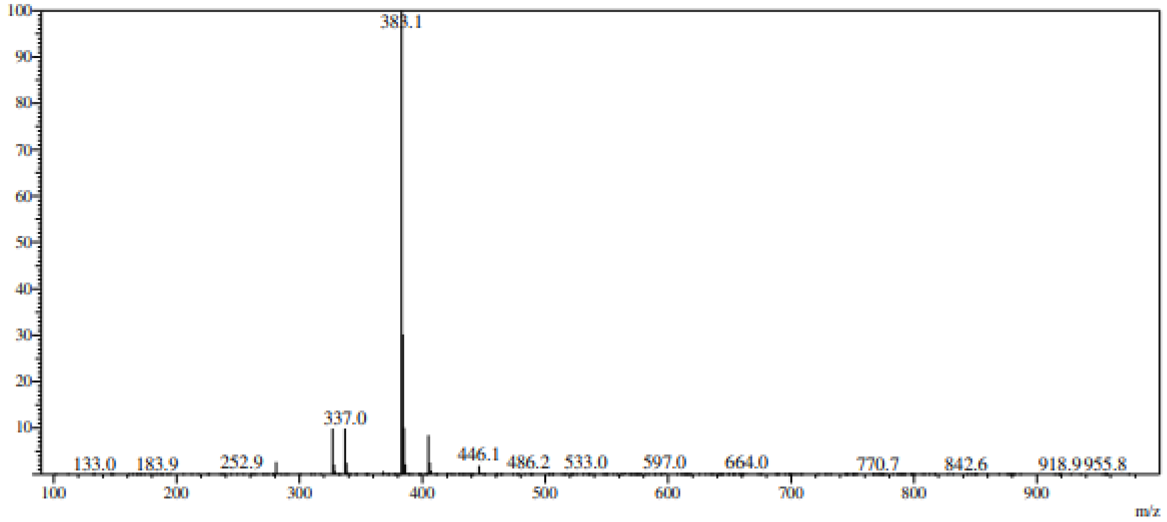

Supplementary Figure 44. 1H NMR of PF-07238025

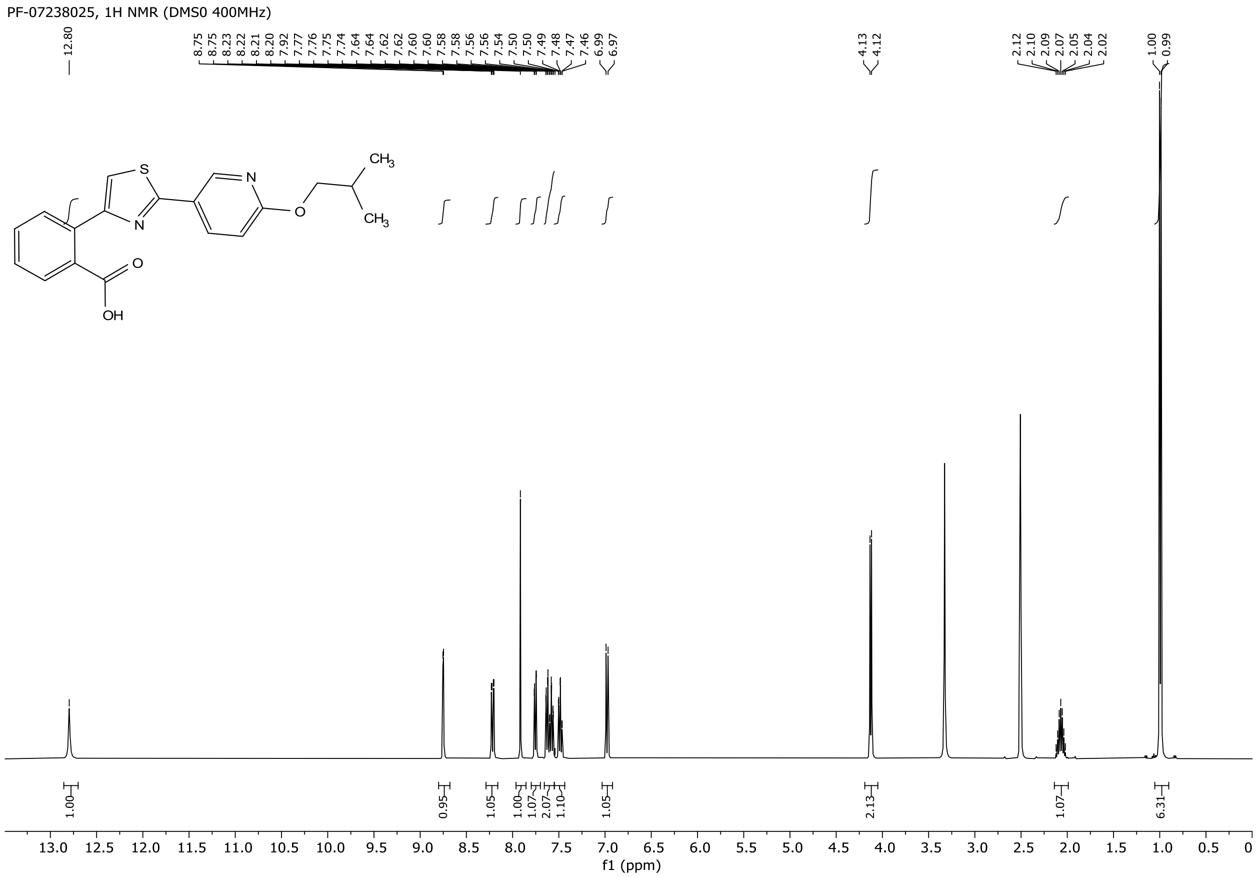

Supplementary Figure 45. 13C NMR of PF-07238025

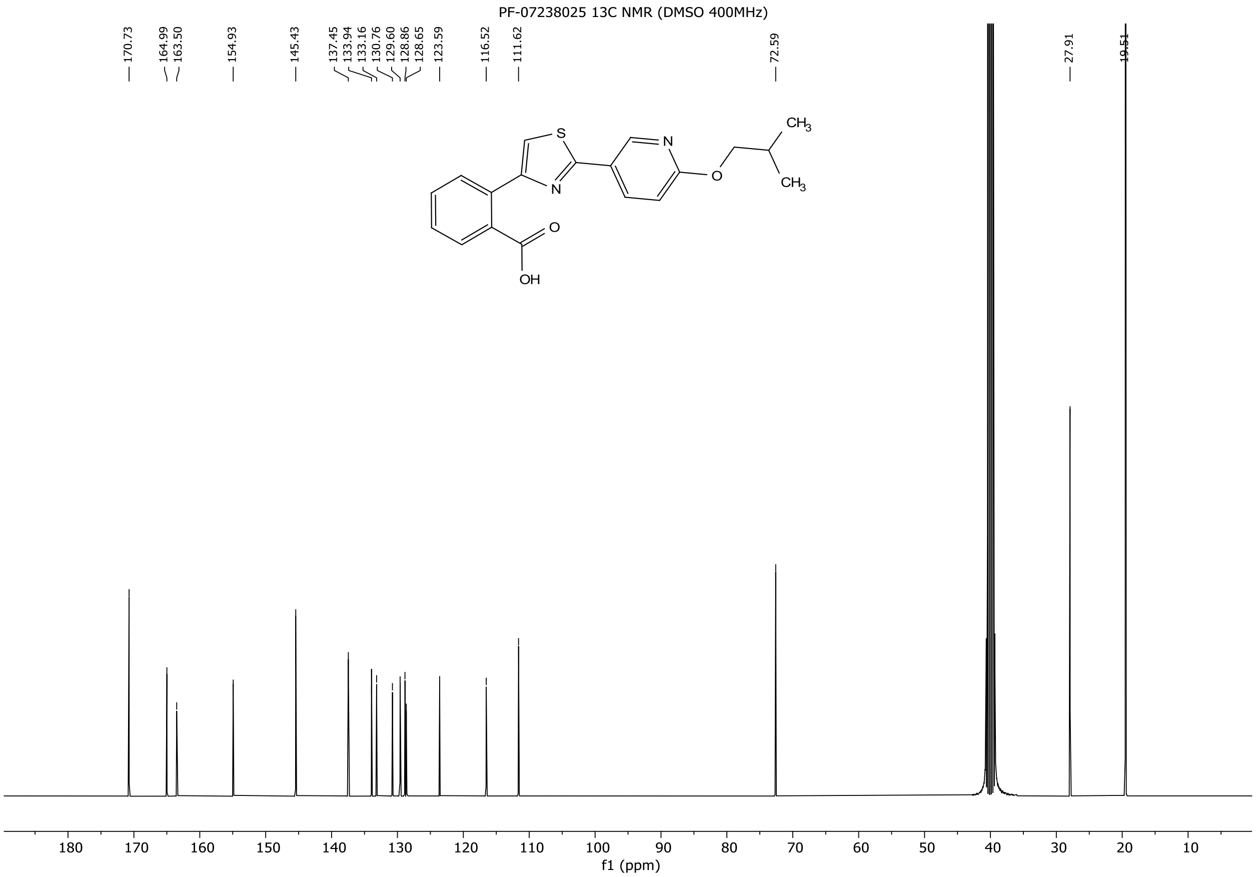

Supplementary Figure 46. HRMS of PF-07238025

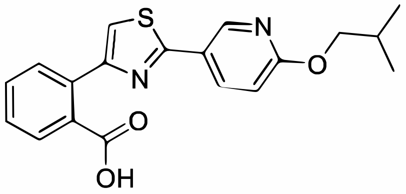

PF-07238025

Open Access HRMS Sample Report			
**Sample ID**	00110580-3430-001	**HPLC**	Agilent 1200
**Molecular Formula**	C19H18N2O3S	**Mass Spectrometer**	Sciex 5600+ QToF
**Submittor**	Martinez, Luis	**DAD**	190-400 nm, reported 270nm
**Run Date**	4/27/2023	**MS Scan**	100-2000 amu
**Analyst**	Quinn, Alandra	**Acquisition Method**	HRMS-3min gradient AQ
**Analyst's CeN**	00714673-0166	**eWB Archive Ref**	00713459-0294

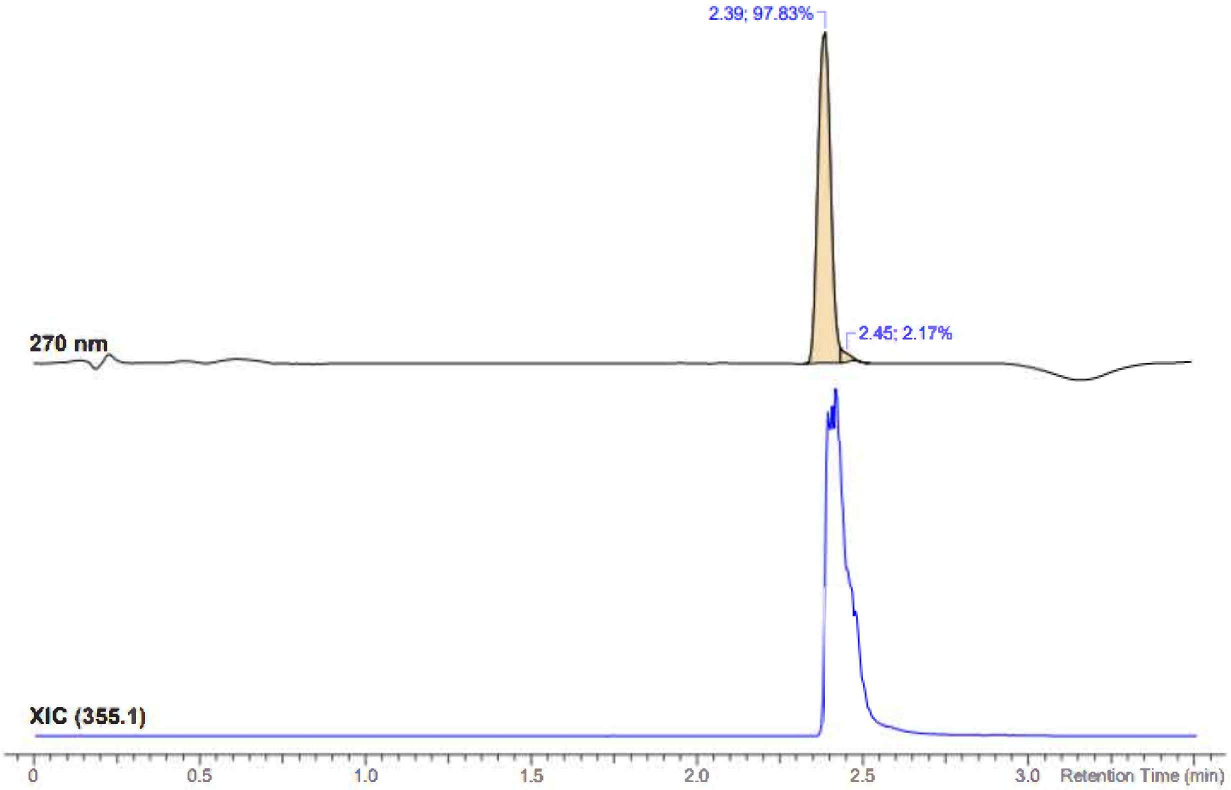

No.	Peak Name	tR (min)	Area (%)
1	C19H18N2O3S	2.39	97.83
2	unknown	2.45	2.17

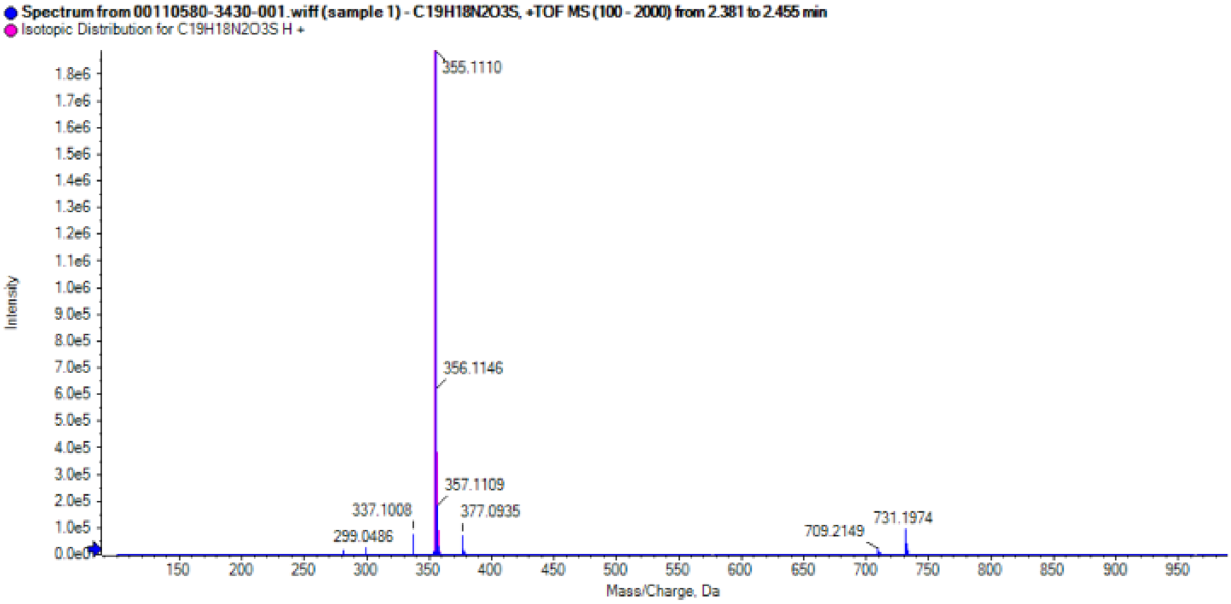

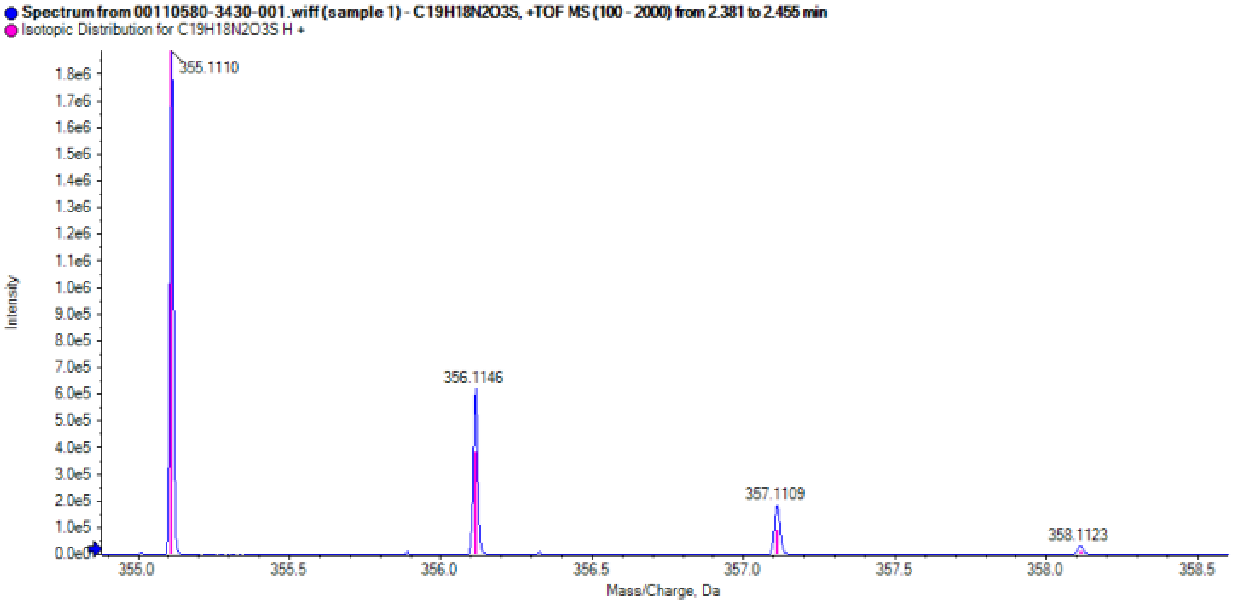

Peak at 2.39 min in UV								
Mass/Charge (Da)	Height	Relative % Height	Compound	Peak Type	Theoretical m/z	Error (ppm)	Theoretical m/z	Theoretical Intensity
355.1110	1886716	100.0	C19H18N2O3S	[M+H]+	355.1111	-0.1	355.1111	100.0
356.1146	622056	33.0					356.1141	23.0
357.1109	184260	9.8					357.1110	7.5
358.1123	32639	1.7					358.1123	1.3

Supplementary Figure 47. HPLC of PF-07238025

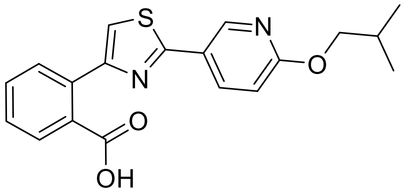

PF-07238025

```
======================================================================
Acq. Operator   : SYSTEM                        Seq. Line :   4
Sample Operator : SYSTEM
Acq. Instrument : HPLC-233GA                     Location :   9
Injection Date  : 4/27/2023 1:16:43 PM                Inj :   1
                                               Inj Volume : 10.000 µl
Acq. Method     : C:\Users\Public\Documents\ChemStation\1\Data\def_LC_2021 2023-04-27 12-29-
                  18\ACID_GRADIENT.M
Last changed    : 6/9/2021 9:16:24 AM by SYSTEM
Analysis Method : C:\Users\Public\Documents\ChemStation\1\Data\def_LC_2021 2023-04-27 12-29-
                  18\ACID_GRADIENT.M (Sequence Method)
Last changed    : 4/28/2023 2:01:26 PM by SYSTEM
                  (modified after loading)
Method Info     : Column: XBridge C18 5 micron (4.6 mm x 150 mm)
                  Flow rate: 1.500 mL/min with solvents containing 0.1% TFA
                  0-1.5 min: 5% acetonitrile/water
                  1.5-10 min: 5-100% acetonitrile water
                  10-11 min: 100% acetonitrile
                  11-12.5 min: 100-5% acetonitrile/water
```

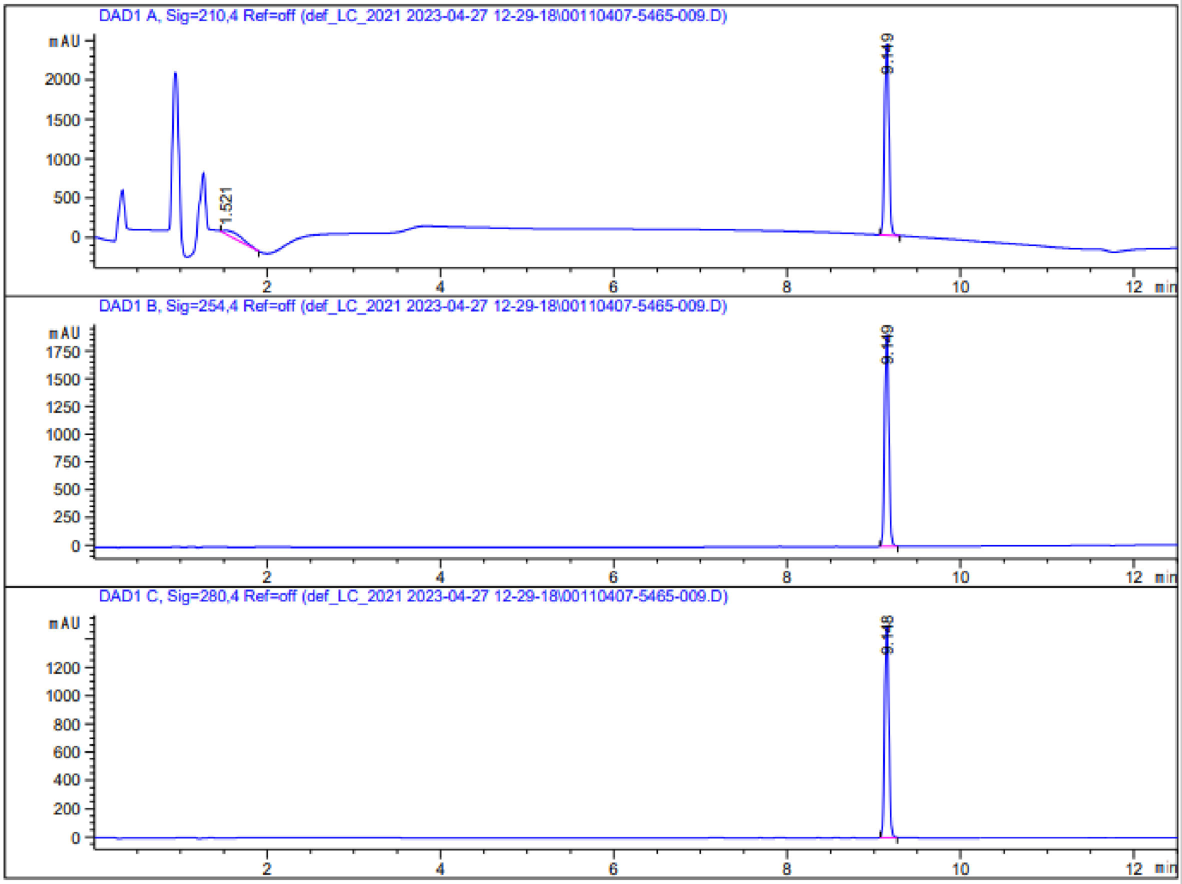

===

Area Percent Report

===

Sorted By : Signal
Multiplier : 1.0000
Dilution : 1.0000
Do not use Multiplier & Dilution Factor with ISTDs

Signal 1: DAD1 A, Sig=210,4 Ref=off

Peak #	RetTime [min]	Type	Width [min]	Area [mAU*s]	Height [mAU]	Area %
1	1.521	BB	0.3515	1026.59692	44.11615	10.7388
2	9.149	BB	0.0549	8533.05957	2434.75903	89.2612

Totals : 9559.65649 2478.87518

Signal 2: DAD1 B, Sig=254,4 Ref=off

Peak #	RetTime [min]	Type	Width [min]	Area [mAU*s]	Height [mAU]	Area %
1	9.149	BB	0.0534	6460.28711	1915.08313	100.0000

Totals : 6460.28711 1915.08313

Signal 3: DAD1 C, Sig=280,4 Ref=off

Peak #	RetTime [min]	Type	Width [min]	Area [mAU*s]	Height [mAU]	Area %
1	9.148	BB	0.0533	5073.91406	1506.82288	100.0000

Totals : 5073.91406 1506.82288

===

*** End of Report ***

Supplementary Figure 48. 1H NMR of compound S12

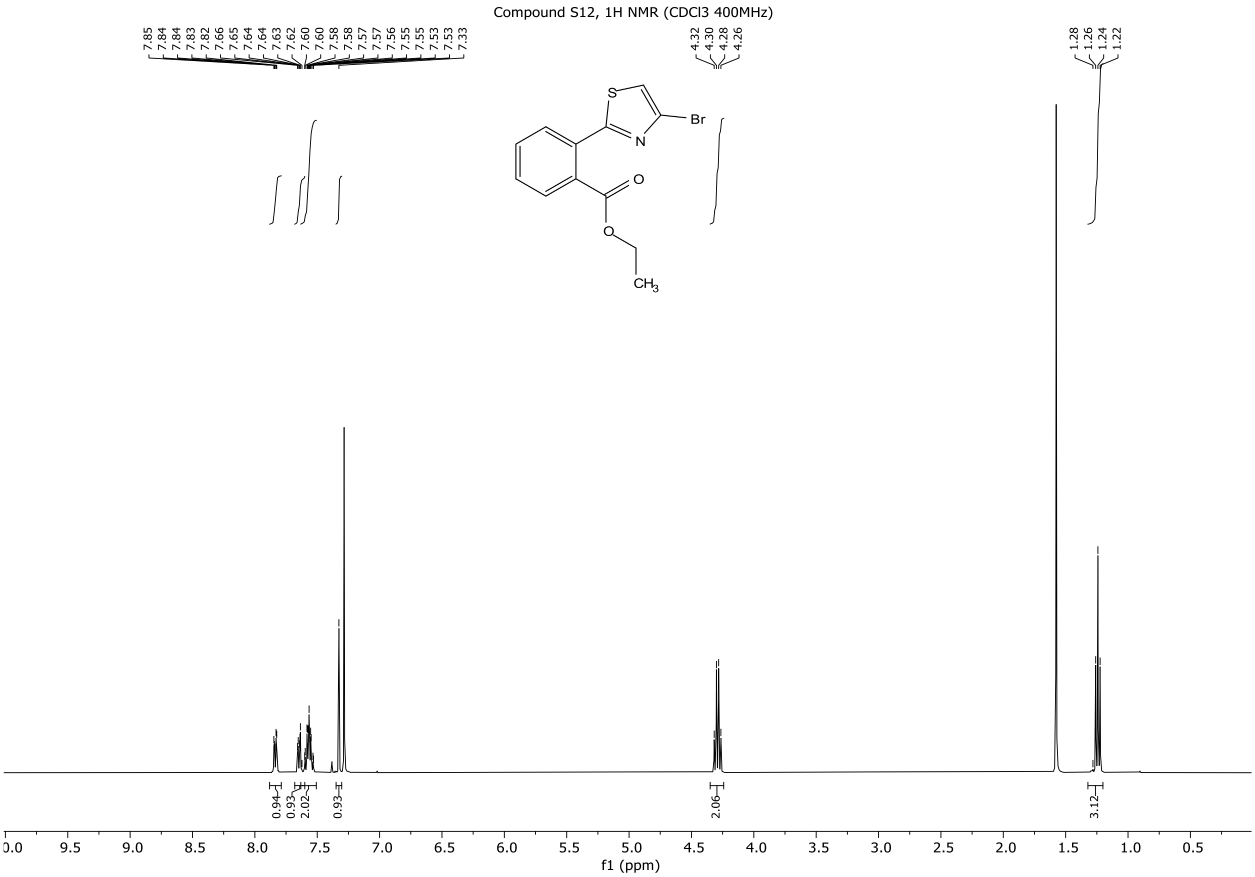

Supplementary Figure 49. 13C NMR of compound S12

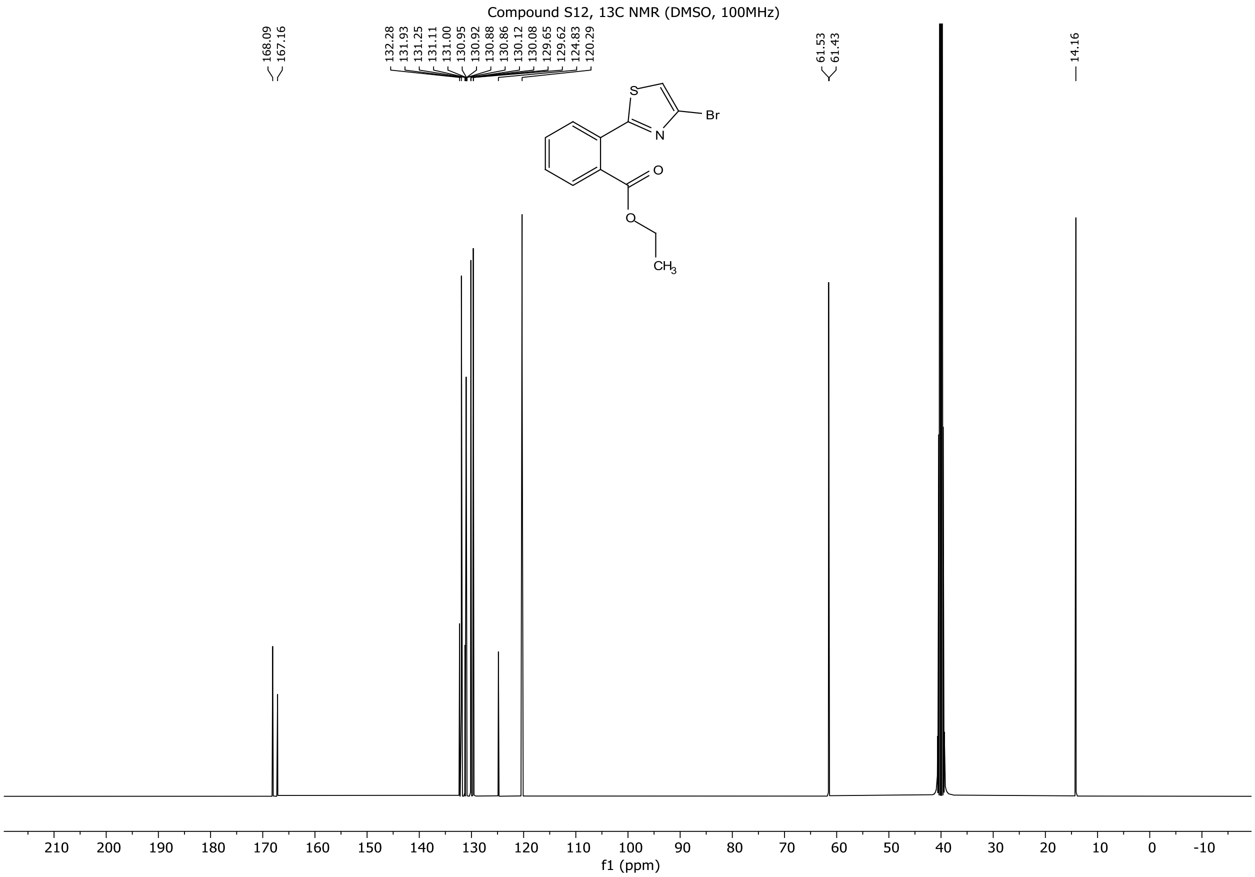

Supplementary Figure 50. HRMS of compound S12

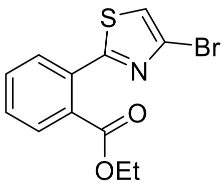

S12

Open Access HRMS Sample Report			
**Sample ID**	00711867-0388-001	**HPLC**	Agilent 1200
**Molecular Formula**	C12H10BrNO2S	**Mass Spectrometer**	Sciex 5600+ QToF
**Submittor**	Martinez, Luis	**DAD**	190-400 nm, reported 270nm
**Run Date**	4/27/2023	**MS Scan**	100-2000 amu
**Analyst**	Quinn, Alandra	**Acquisition Method**	HRMS-3min gradient AQ
**Analyst's CeN**	00714673-0166	**eWB Archive Ref**	00713459-0294

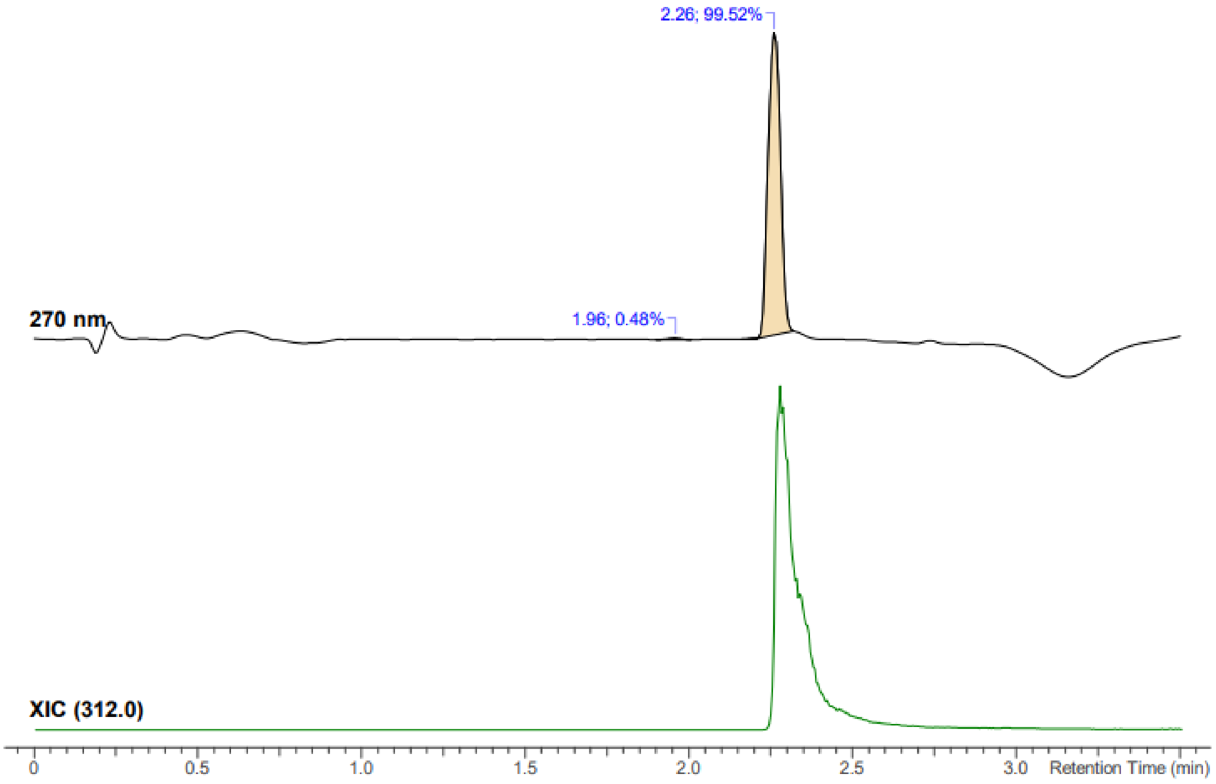

No.	Peak Name	tR (min)	Area (%)
1	unknown	1.96	0.48
2	C12H10BrNO2S	2.26	99.52

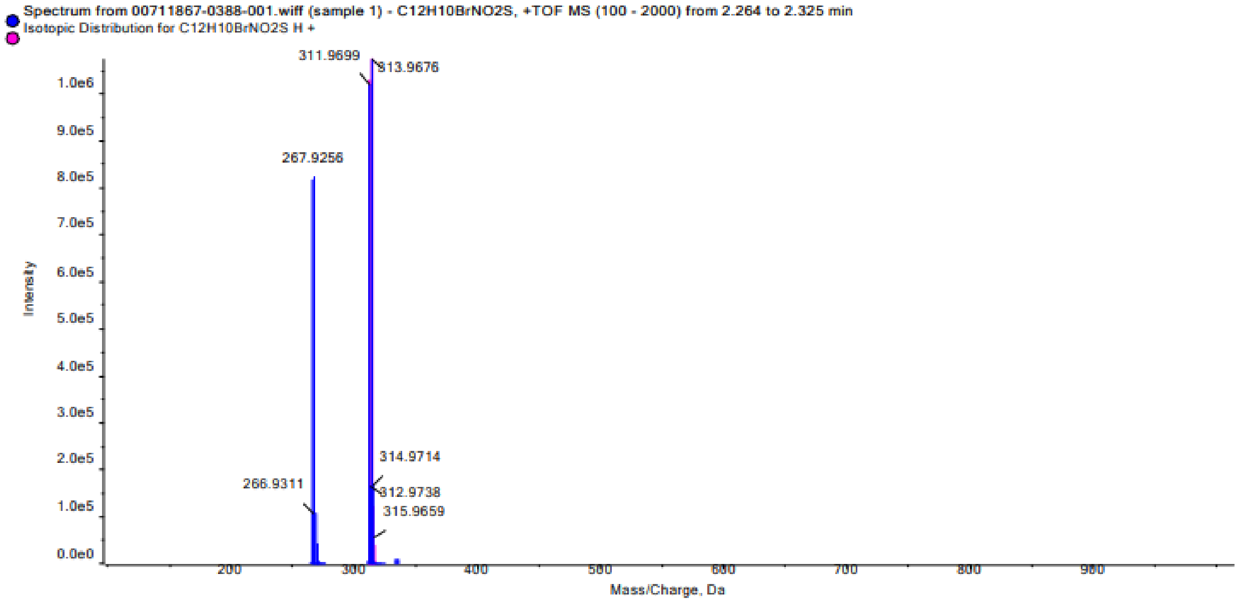

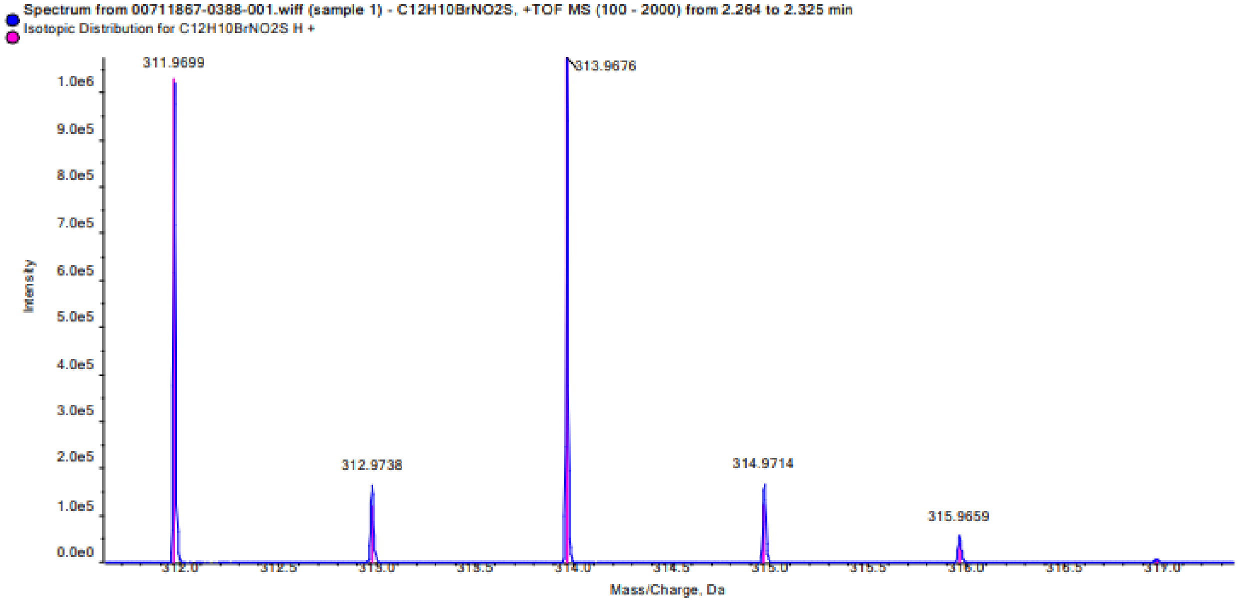

Peak at 2.26 min in UV								
Mass/Charge (Da)	Height	Relative % Height	Compound	Peak Type	Theoretical m/z	Error (ppm)	Theoretical m/z	Theoretical Intensity
311.9699	1021812	95.1	C12H10BrNO2S	[M+H]+	311.9688	3.4	311.9688	97.0
312.9738	164442	15.3					312.9719	14.3
313.9676	1073990	100.0					313.9668	100.0
314.9714	166621	15.5					314.9698	14.6
315.9659	57382	5.3					315.9650	5.6

Supplementary Figure 51. HPLC of compound S12

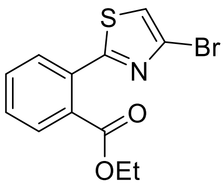

S12

```
=====================================================================
Acq. Operator   : SYSTEM                        Seq. Line :    5
Sample Operator : SYSTEM
Acq. Instrument : HPLC-233GA                     Location :   10
Injection Date  : 4/27/2023 1:30:55 PM                Inj :    1
                                               Inj Volume : 10.000 µl
Acq. Method     : C:\Users\Public\Documents\ChemStation\1\Data\def_LC_2021 2023-04-27 12-29-
                  18\ACID_GRADIENT.M
Last changed    : 6/9/2021 9:16:24 AM by SYSTEM
Analysis Method : C:\Users\Public\Documents\ChemStation\1\Data\def_LC_2021 2023-04-27 12-29-
                  18\ACID_GRADIENT.M (Sequence Method)
Last changed    : 4/28/2023 2:01:26 PM by SYSTEM
                  (modified after loading)
Method Info     : Column: XBridge C18 5 micron (4.6 mm x 150 mm)
                  Flow rate: 1.500 mL/min with solvents containing 0.1% TFA
                  0-1.5 min: 5% acetonitrile/water
                  1.5-10 min: 5-100% acetonitrile water
                  10-11 min: 100% acetonitrile
                  11-12.5 min: 100-5% acetonitrile/water
```

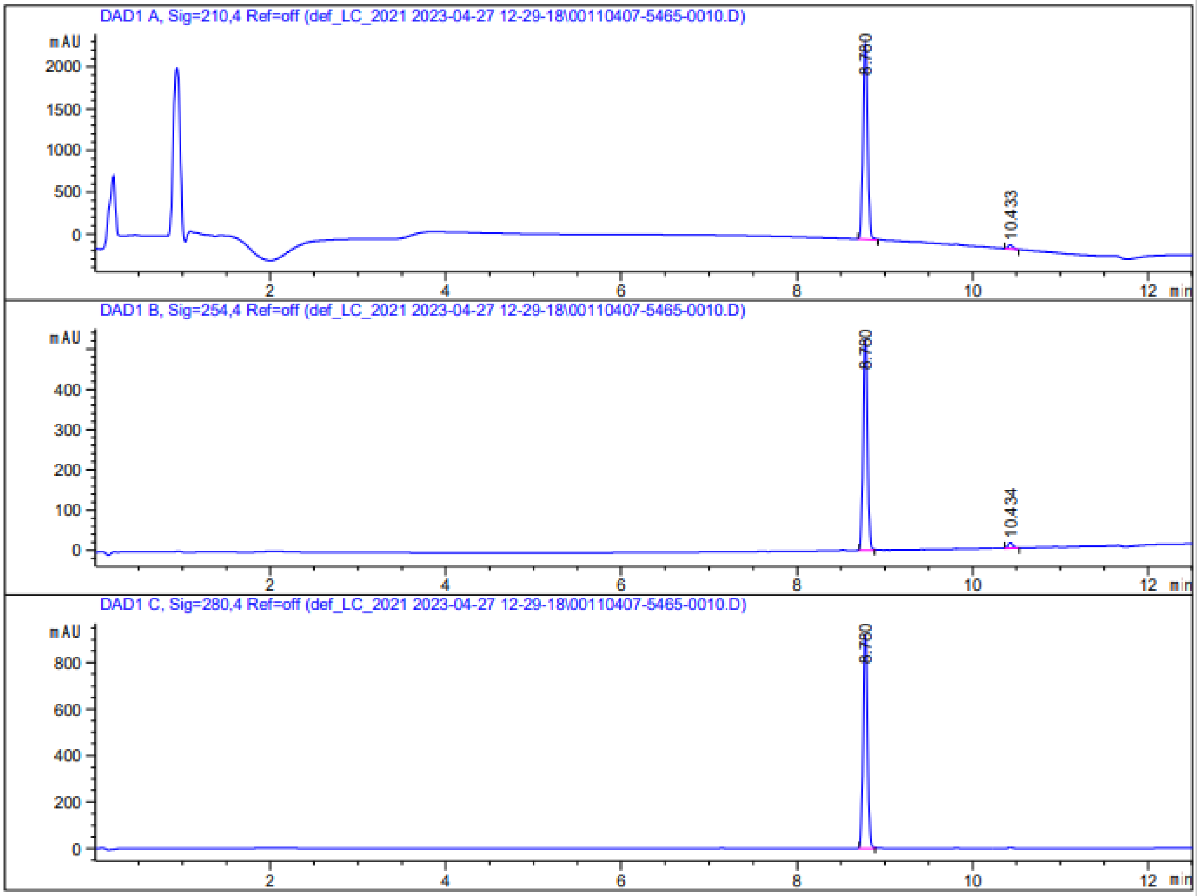

===
Area Percent Report
===

Sorted By : Signal
Multiplier : 1.0000
Dilution : 1.0000
Do not use Multiplier & Dilution Factor with ISTDs

Signal 1: DAD1 A, Sig=210,4 Ref=off

Peak #	RetTime [min]	Type	Width [min]	Area [mAU*s]	Height [mAU]	Area %
1	8.780	BB	0.0568	8567.22363	2332.77637	97.9666
2	10.433	BB	0.0537	177.82544	52.29279	2.0334

Totals : 8745.04907 2385.06916

Signal 2: DAD1 B, Sig=254,4 Ref=off

Peak #	RetTime [min]	Type	Width [min]	Area [mAU*s]	Height [mAU]	Area %
1	8.780	BB	0.0542	1805.83240	523.63135	97.4147
2	10.434	BB	0.0533	47.92553	14.21884	2.5853

Totals : 1853.75793 537.85019

Signal 3: DAD1 C, Sig=280,4 Ref=off

Peak #	RetTime [min]	Type	Width [min]	Area [mAU*s]	Height [mAU]	Area %
1	8.780	BB	0.0543	3189.18506	923.13580	100.0000

Totals : 3189.18506 923.13580

===
*** End of Report ***

Supplementary Figure 52. 1H NMR of compound S13

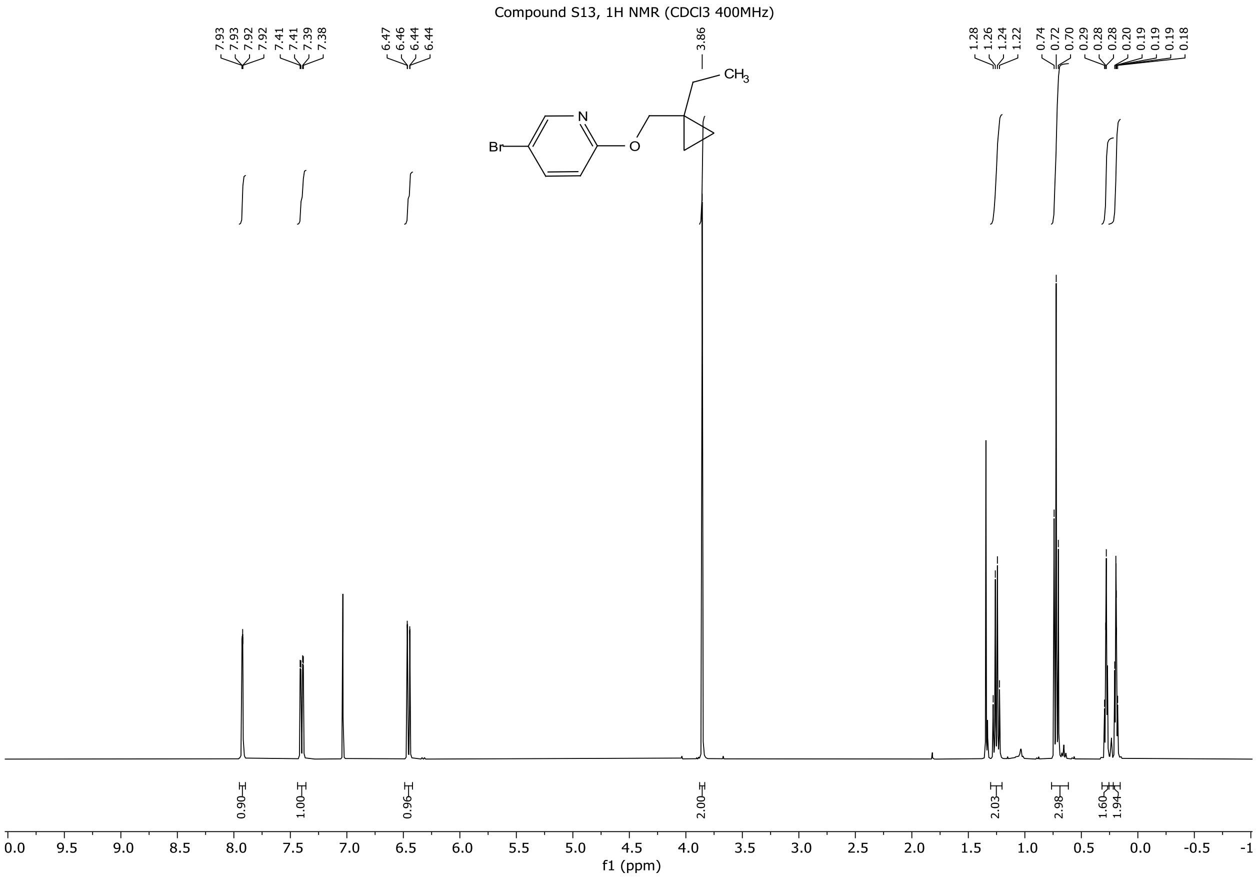

Supplementary Figure 53. 13C NMR of compound S13

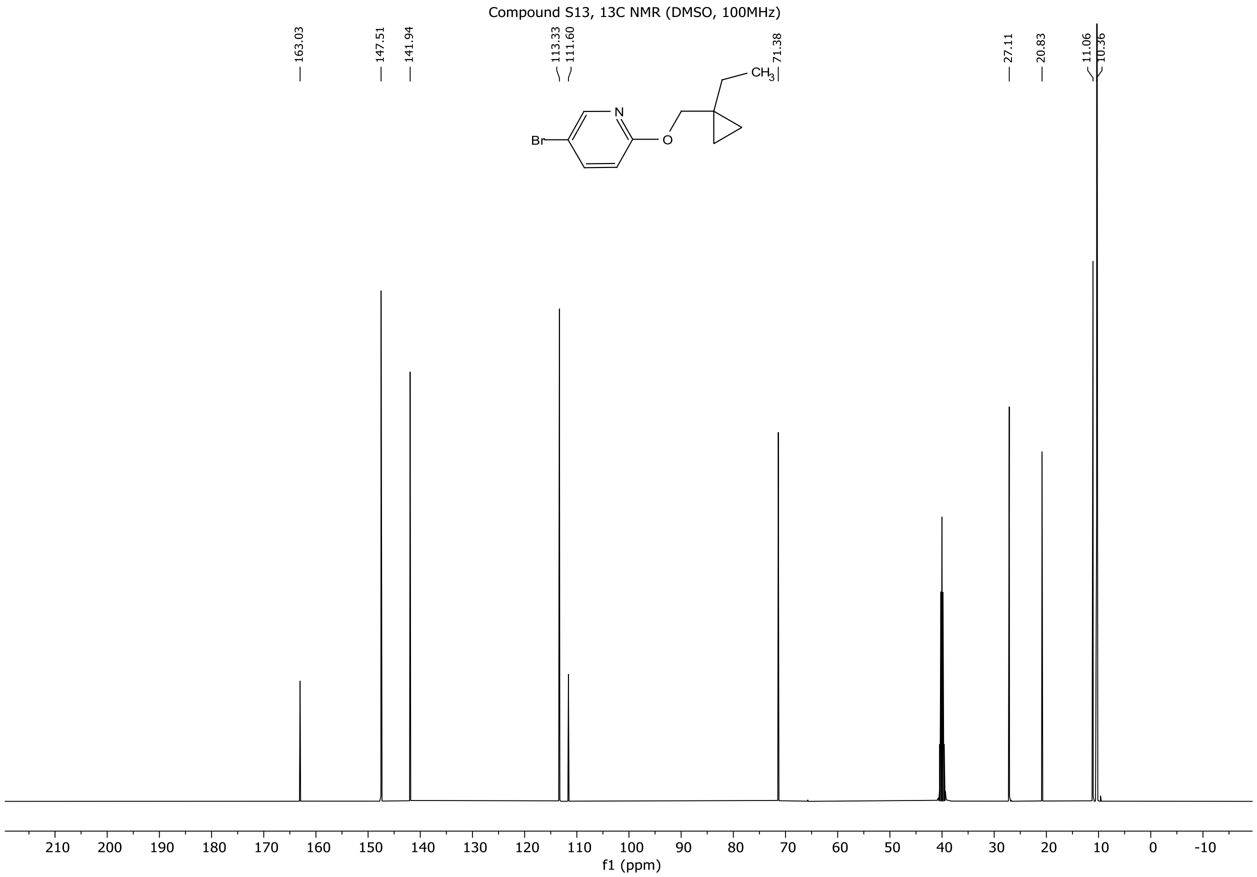

Supplementary Figure 54. HRMS of compound S13

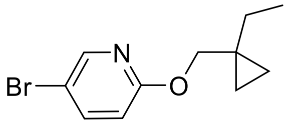

S13

Open Access HRMS Sample Report			
**Sample ID**	00180155-0098-001	**HPLC**	Agilent 1200
**Molecular Formula**	C11H14BrNO	**Mass Spectrometer**	Sciex 5600+ QToF
**Submittor**	Martinez, Luis	**DAD**	190-400 nm, reported 270nm
**Run Date**	4/27/2023	**MS Scan**	100-2000 amu
**Analyst**	Quinn, Alandra	**Acquisition Method**	HRMS-3min gradient AQ
**Analyst's CeN**	00714673-0166	**eWB Archive Ref**	00713459-0294

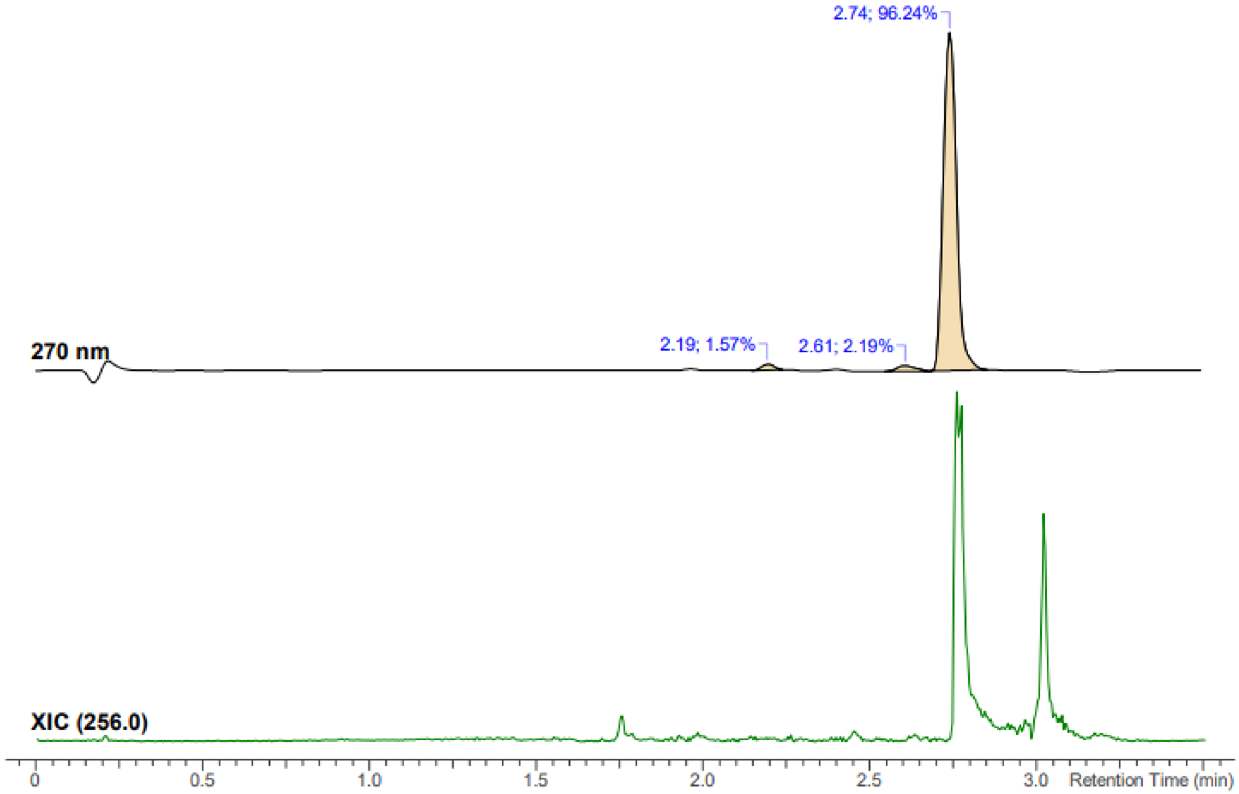

No.	Peak Name	tR (min)	Area (%)
1	unknown	2.19	1.57
2	unknown	2.61	2.19
3	C11H14BrNO	2.74	96.24

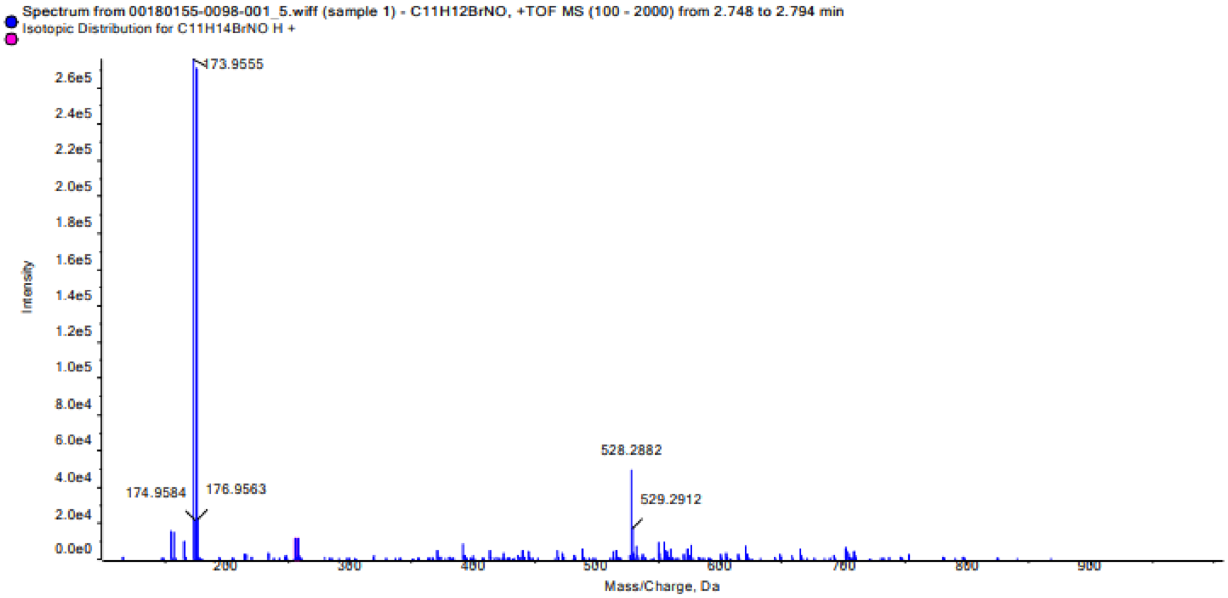

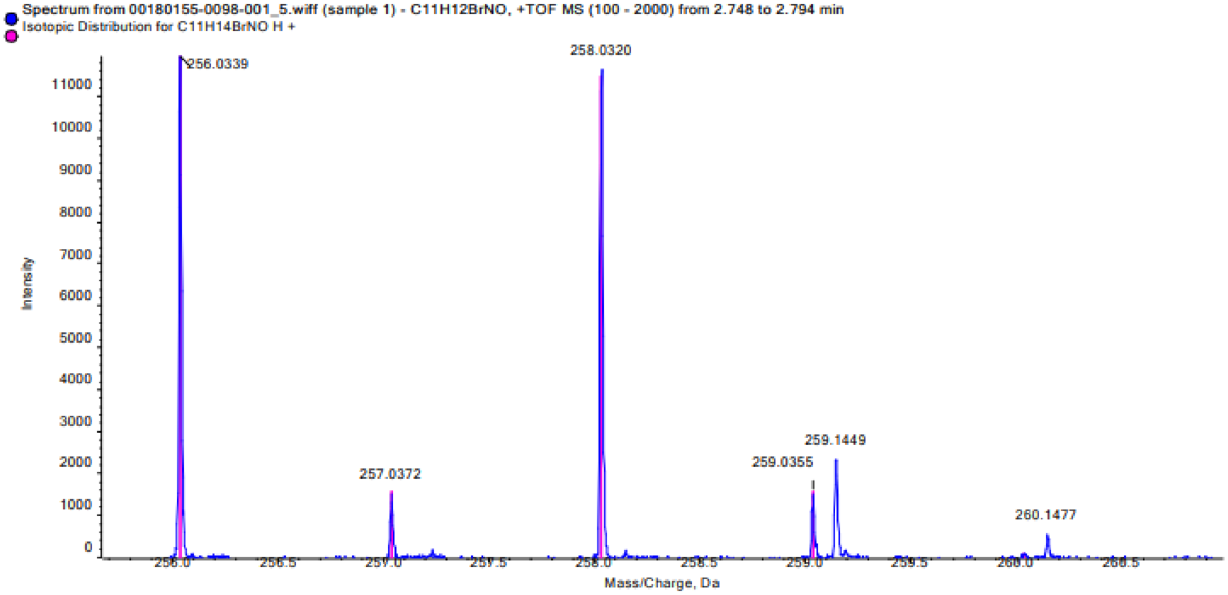

Peak at 2.74 min in UV								
Mass/Charge (Da)	Height	Relative % Height	Compound	Peak Type	Theoretical m/z	Error (ppm)	Theoretical m/z	Theoretical Intensity
256.0339	11951	100.0	C11H14BrNO	[M+H]+	256.0332	2.8	256.0332	100.0
257.0372	1510	12.6					257.0364	12.8
258.0320	11641	97.4					258.0312	98.2
259.0355	1504	12.6					259.0343	12.5
260.0388	93	0.8					258.0214	0.9

Supplementary Figure 55. HPLC of compound S13

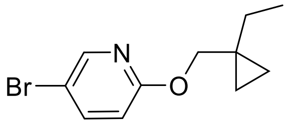

S13

```
=====================================================================
Acq. Operator   : SYSTEM                          Seq. Line :   3
Sample Operator : SYSTEM
Acq. Instrument : HPLC-233GA                       Location :   8
Injection Date  : 4/27/2023 1:02:46 PM                  Inj :   1
                                                Inj Volume : 10.000 µl
Acq. Method     : C:\Users\Public\Documents\ChemStation\1\Data\def_LC_2021 2023-04-27 12-29-
                  18\ACID_GRADIENT.M
Last changed    : 6/9/2021 9:16:24 AM by SYSTEM
Analysis Method : C:\Users\Public\Documents\ChemStation\1\Data\def_LC_2021 2023-04-27 12-29-
                  18\ACID_GRADIENT.M (Sequence Method)
Last changed    : 4/28/2023 2:01:26 PM by SYSTEM
                  (modified after loading)
Method Info     : Column: XBridge C18 5 micron (4.6 mm x 150 mm)
                  Flow rate: 1.500 mL/min with solvents containing 0.1% TFA
                  0-1.5 min: 5% acetonitrile/water
                  1.5-10 min: 5-100% acetonitrile water
                  10-11 min: 100% acetonitrile
                  11-12.5 min: 100-5% acetonitrile/water
```

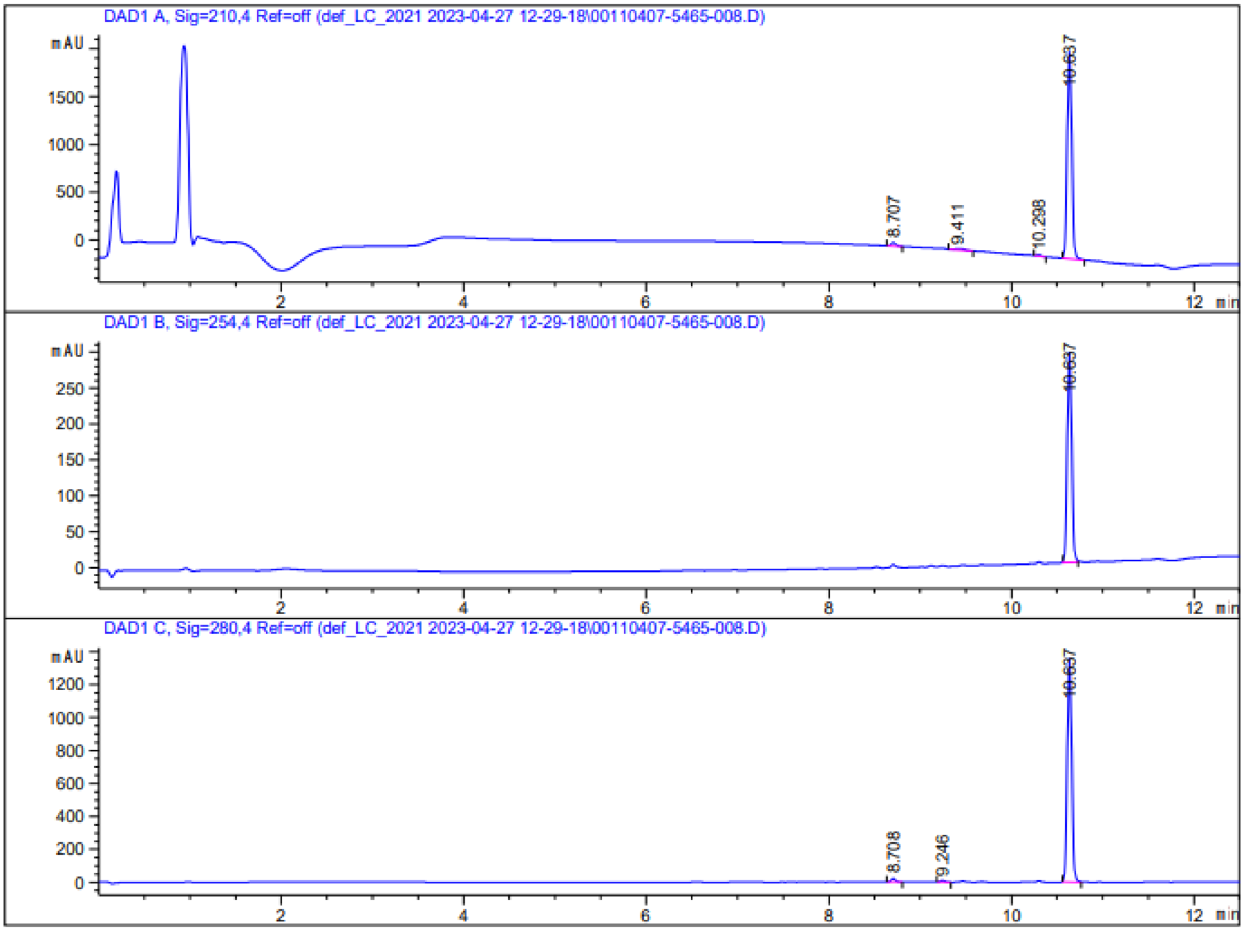

===

Area Percent Report

===

Sorted By : Signal
Multiplier : 1.0000
Dilution : 1.0000
Do not use Multiplier & Dilution Factor with ISTDs

Signal 1: DAD1 A, Sig=210,4 Ref=off

Peak #	RetTime [min]	Type	Width [min]	Area [mAU*s]	Height [mAU]	Area %
1	8.707	BB	0.0581	145.41386	38.43911	1.8333
2	9.411	BB	0.0953	91.78696	12.84769	1.1572
3	10.298	BB	0.0551	45.86970	13.01597	0.5783
4	10.637	BB	0.0550	7648.67236	2175.46094	96.4312

Totals : 7931.74288 2239.76370

Signal 2: DAD1 B, Sig=254,4 Ref=off

Peak #	RetTime [min]	Type	Width [min]	Area [mAU*s]	Height [mAU]	Area %
1	10.637	BB	0.0538	1003.27765	293.85690	100.0000

Totals : 1003.27765 293.85690

Signal 3: DAD1 C, Sig=280,4 Ref=off

Peak #	RetTime [min]	Type	Width [min]	Area [mAU*s]	Height [mAU]	Area %
1	8.708	BB	0.0569	83.47157	22.68753	1.7387
2	9.246	BB	0.0537	38.49805	11.30905	0.8019
3	10.637	BB	0.0540	4678.93945	1365.01904	97.4594

Totals : 4800.90908 1399.01562

===

*** End of Report ***

Supplementary Figure 56. 1H NMR of compound S15

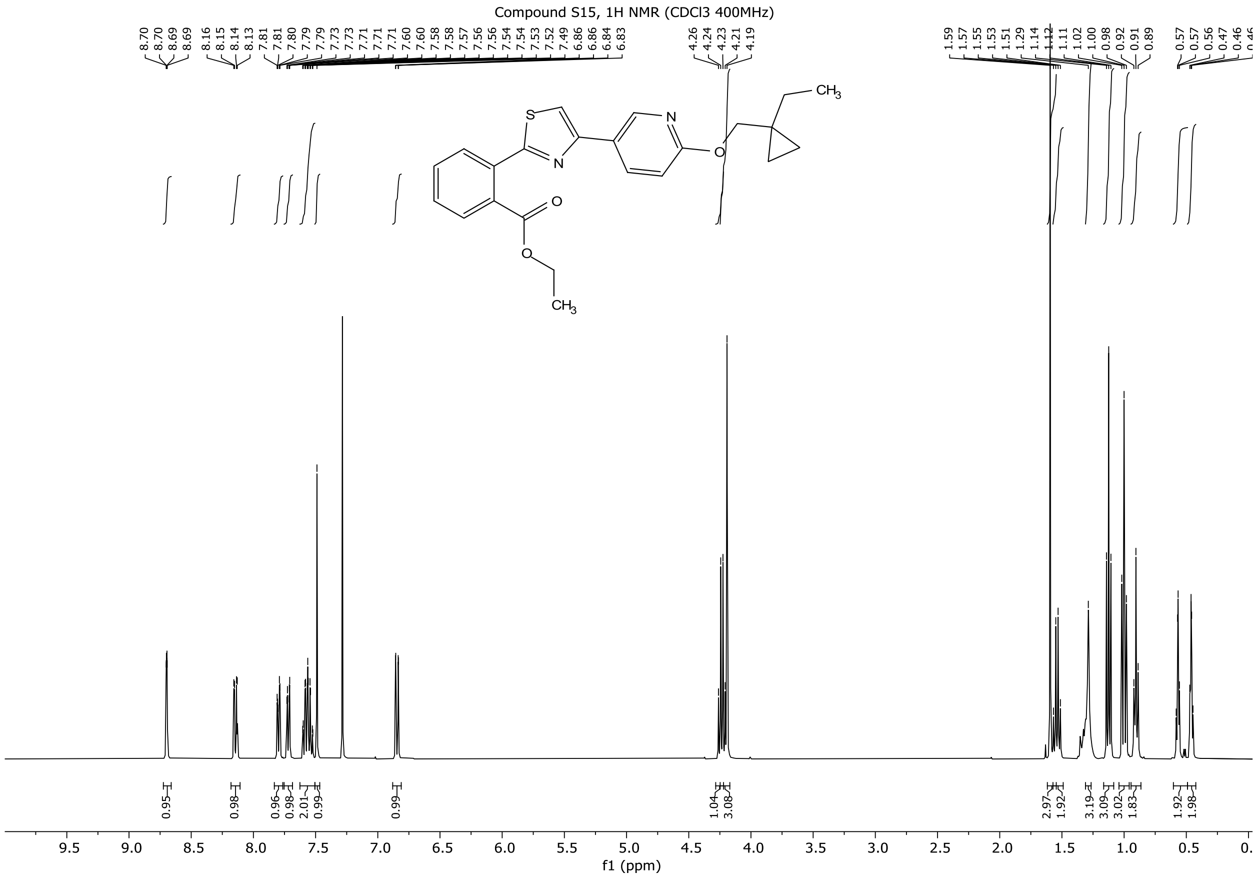

Supplementary Figure 57. LCMS of compound S15

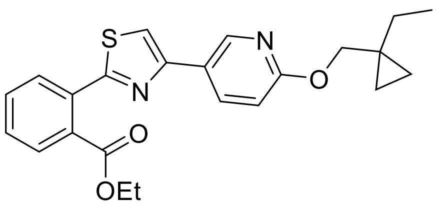

S15

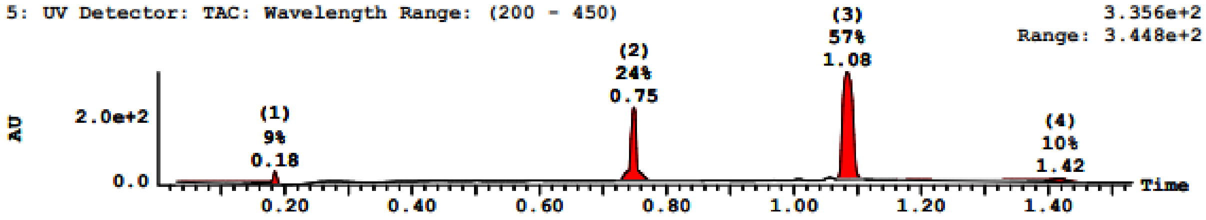

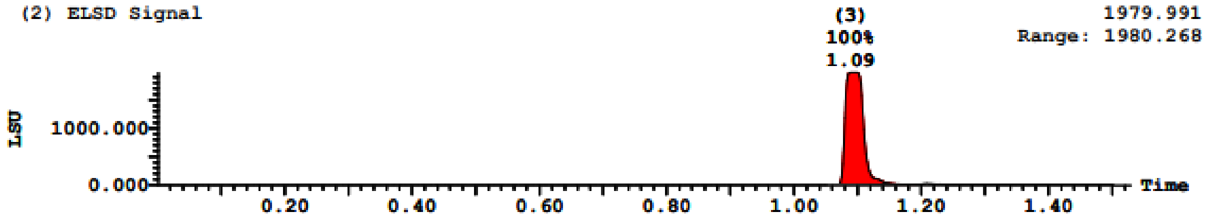

Peak ID	Time	Mass Found	AreaAbs	Area %Total
3	1.09	Not Found	65045	100.00

Peak ID	Time
1	0.18

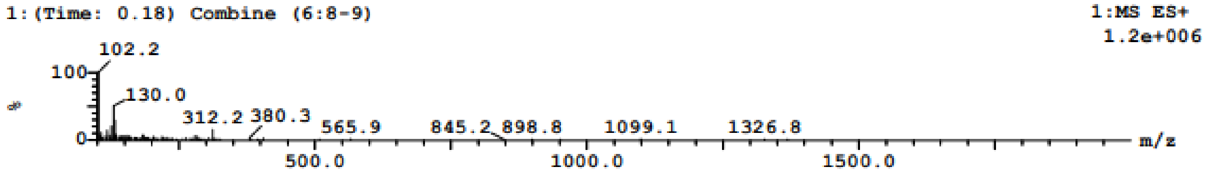

Peak ID	Time
1	0.18

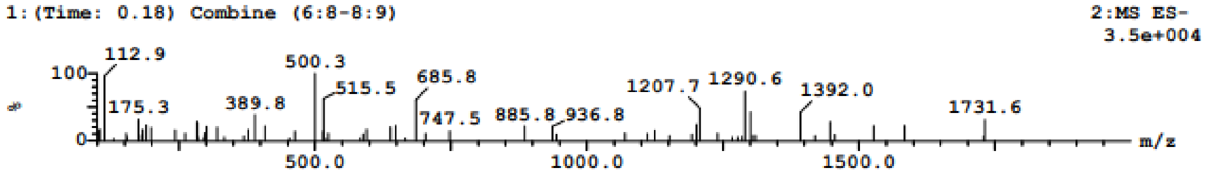

Peak ID	Time
1	0.18

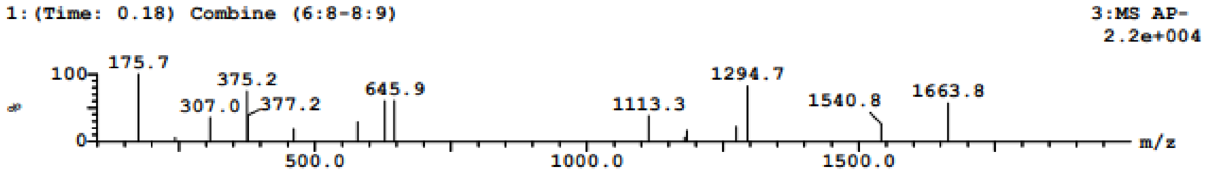

Peak ID	Time
1	0.18

1:(Time: 0.18) Combine (6:7-8:9) 4:MS AP+ 9.8e+004

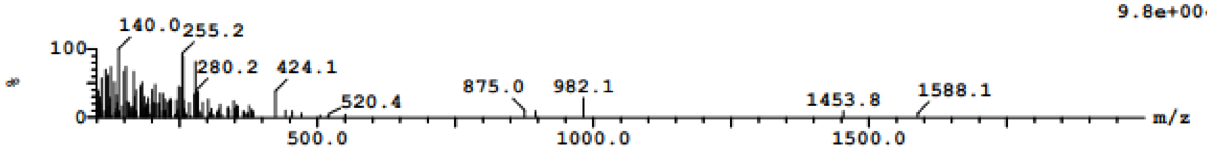

Peak ID	Time
2	0.75

2:(Time: 0.75) Combine (26:28-(24:25+32:33)) 1:MS ES+ 6.3e+006

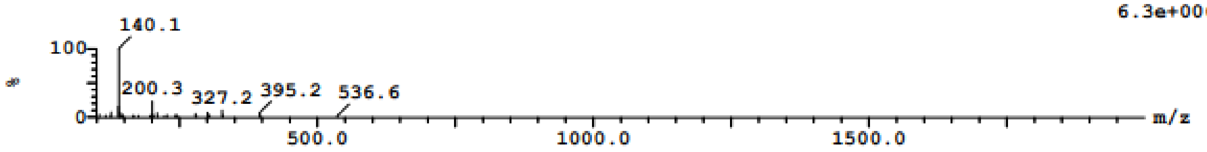

Peak ID	Time
2	0.75

2:(Time: 0.75) Combine (26:28-(24:25+31:32)) 2:MS ES- 3.5e+004

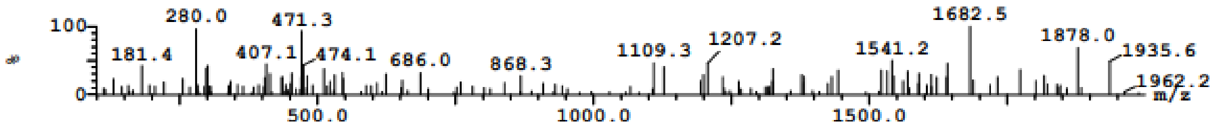

Peak ID	Time
2	0.75

2:(Time: 0.75) Combine (26:27-(24:25+31:32)) 3:MS AP- 4.5e+004

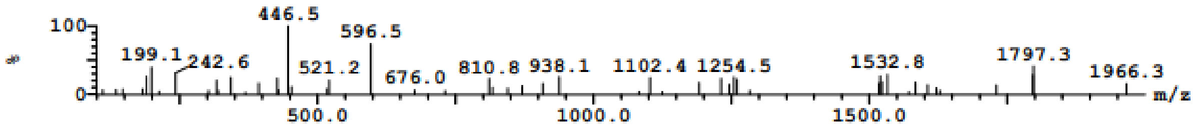

Peak ID	Time
2	0.75

2:(Time: 0.75) Combine (25:27-(24+31:32)) 4:MS AP+ 6.8e+005

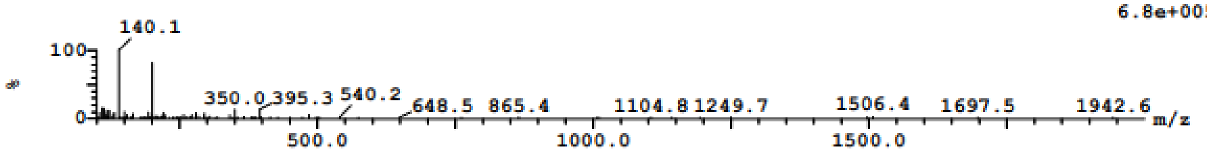

Peak ID	Time
3	1.08

3:(Time: 1.09) Combine (38:40-(36:37+44))

1:MS ES+ 4.5e+007

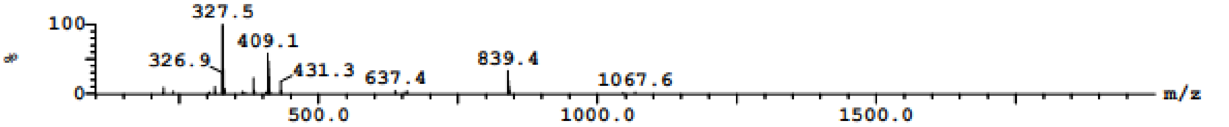

Peak ID	Time
3	1.08

3:(Time: 1.09) Combine (38:40-(36:37+43:44))

2:MS ES- 5.6e+004

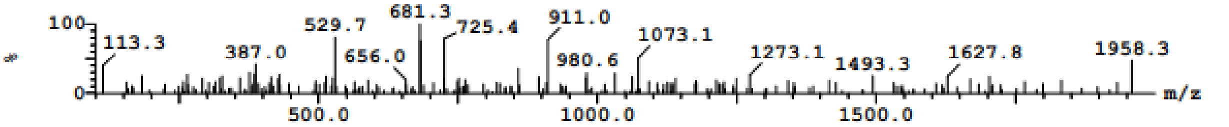

Peak ID	Time
3	1.08

3:(Time: 1.09) Combine (38:39-(36+43:44))

3:MS AP- 5.4e+004

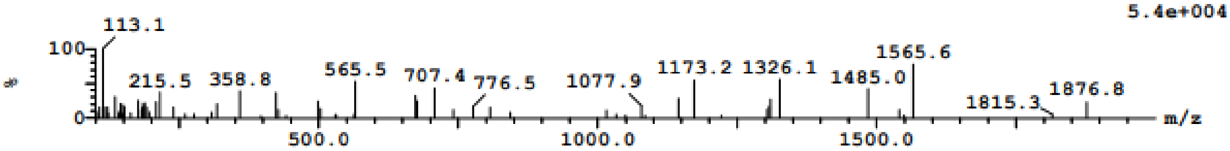

Peak ID	Time
3	1.08

3:(Time: 1.09) Combine (37:39-(35:36+43:44))

4:MS AP+ 1.7e+007

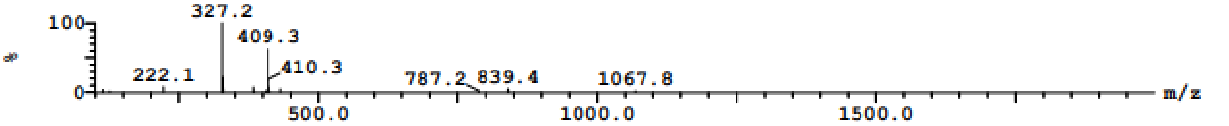

Peak ID	Time
4	1.42

4:(Time: 1.42) Combine (50:52-40:41)

1:MS ES+ 5.0e+006

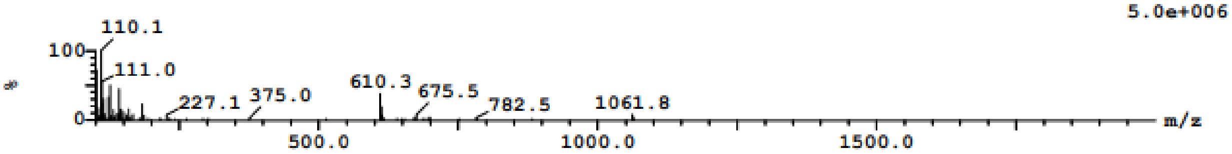

Peak ID	Time
4	1.42

4:(Time: 1.42) Combine (50:51-(40+53))

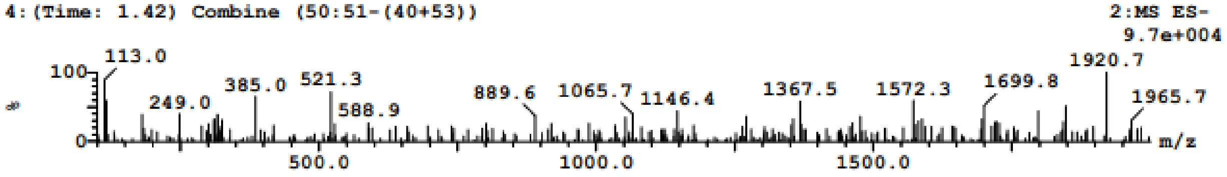

Peak ID	Time
4	1.42

4:(Time: 1.42) Combine (49:51-(39:40+53))

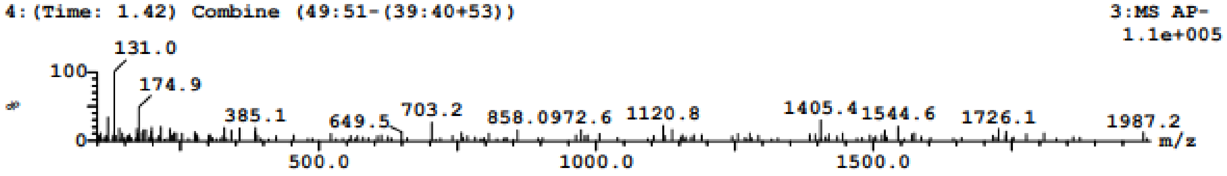

Peak ID	Time
4	1.42

4:(Time: 1.42) Combine (49:51-(39:40+52:53))

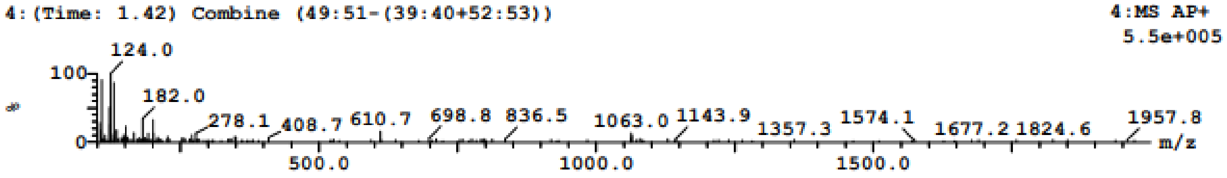

Supplementary Figure 58. 1H NMR of PF-07247685

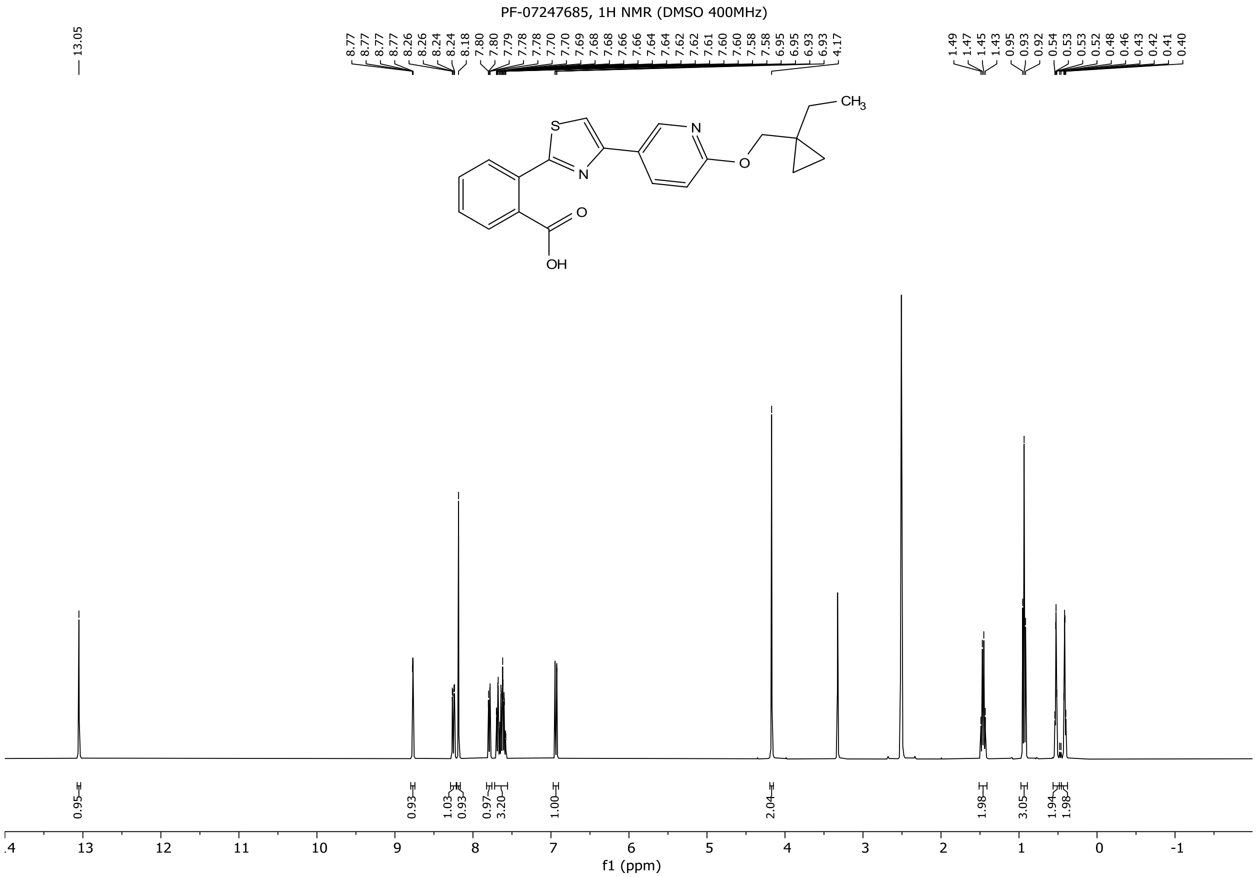

Supplementary Figure 59. 13C NMR of PF-07247685

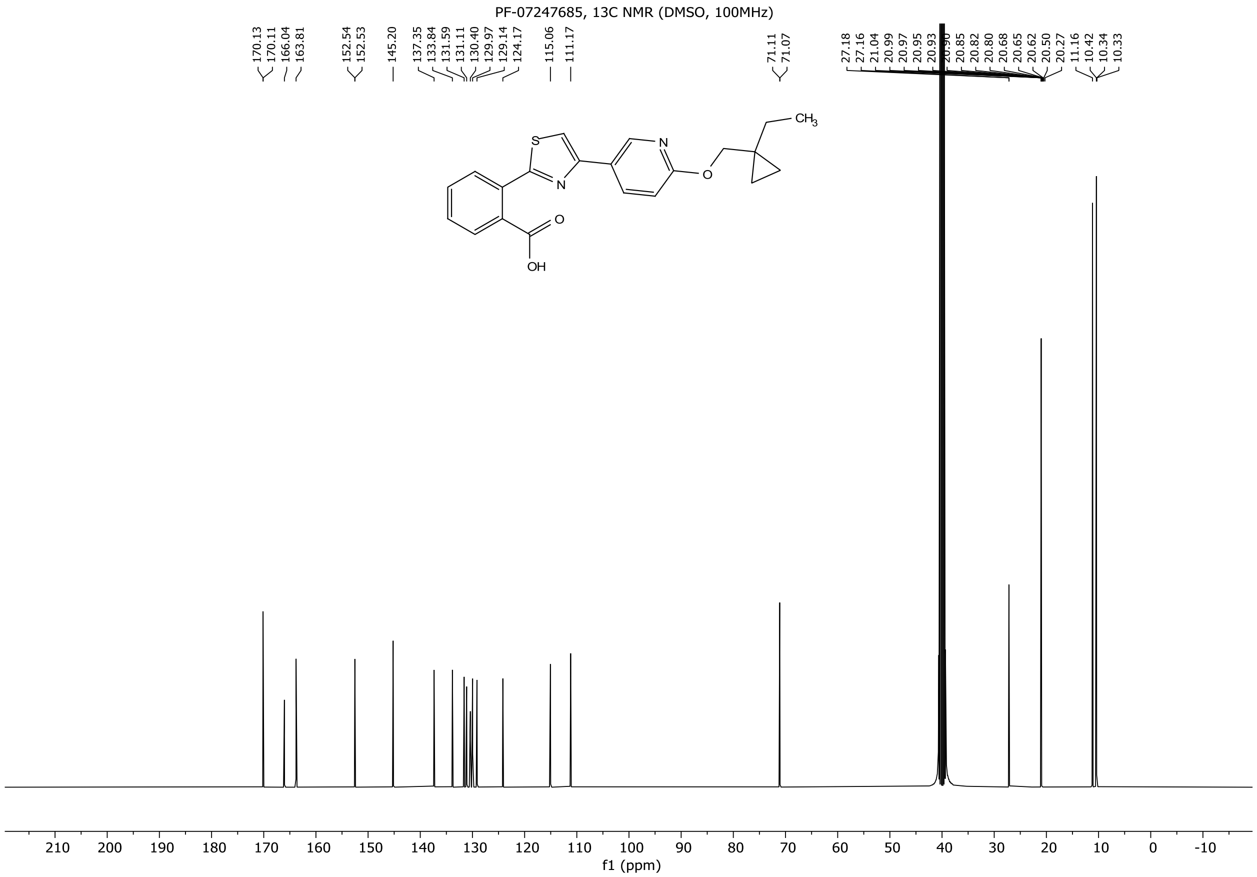

Supplementary Figure 60. HRMS of PF-07247685

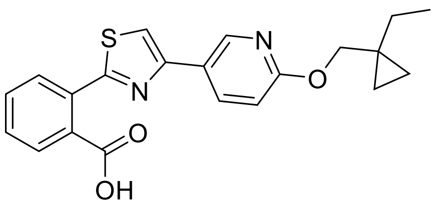

PF-07247685

Open Access HRMS Sample Report			
**Sample ID**	00712228-0051-001	**HPLC**	Agilent 1200
**Molecular Formula**	C21H20N2O3S	**Mass Spectrometer**	Sciex 5600+ QToF
**Submittor**	Martinez, Luis	**DAD**	190-400 nm, reported 270nm
**Run Date**	4/27/2023	**MS Scan**	100-2000 amu
**Analyst**	Quinn, Alandra	**Acquisition Method**	HRMS-3min gradient AQ
**Analyst's CeN**	00714673-0166	**eWB Archive Ref**	00713459-0294

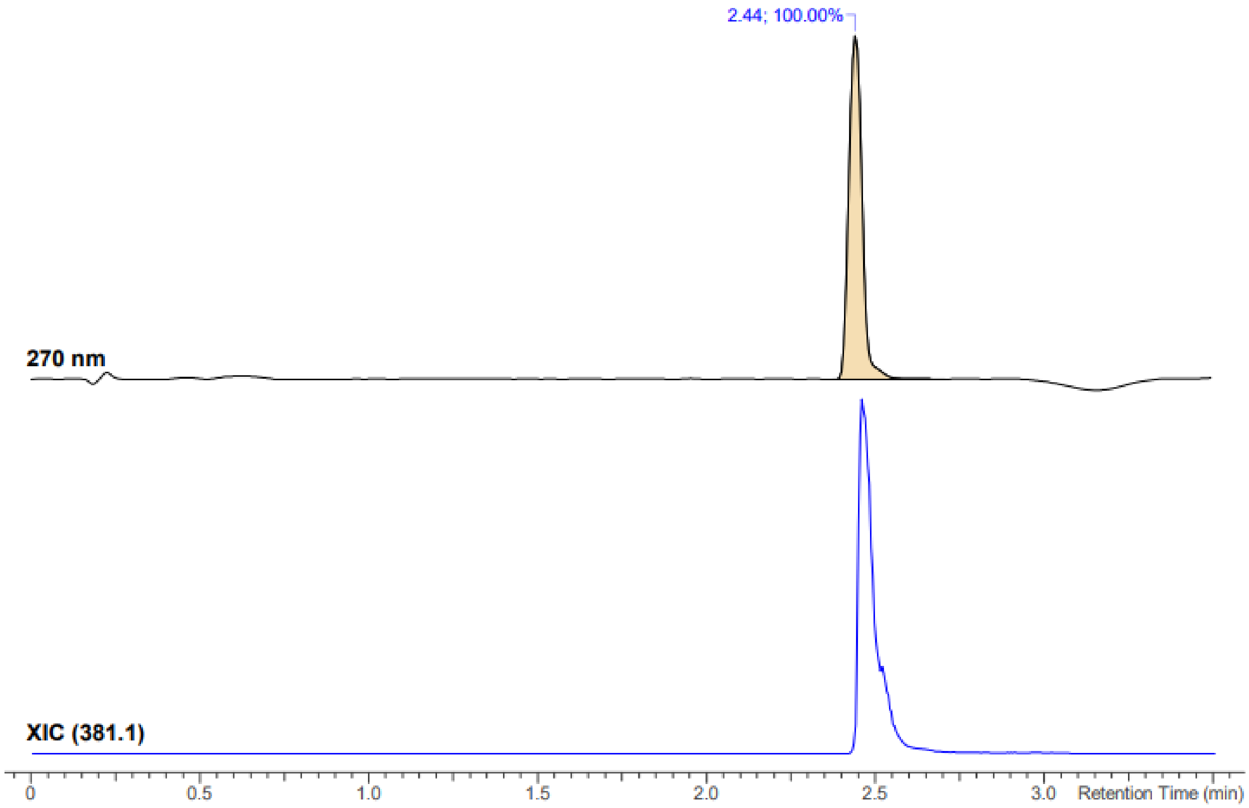

No.	Peak Name	tR (min)	Area (%)
1	C21H20N2O3S	2.44	100.00

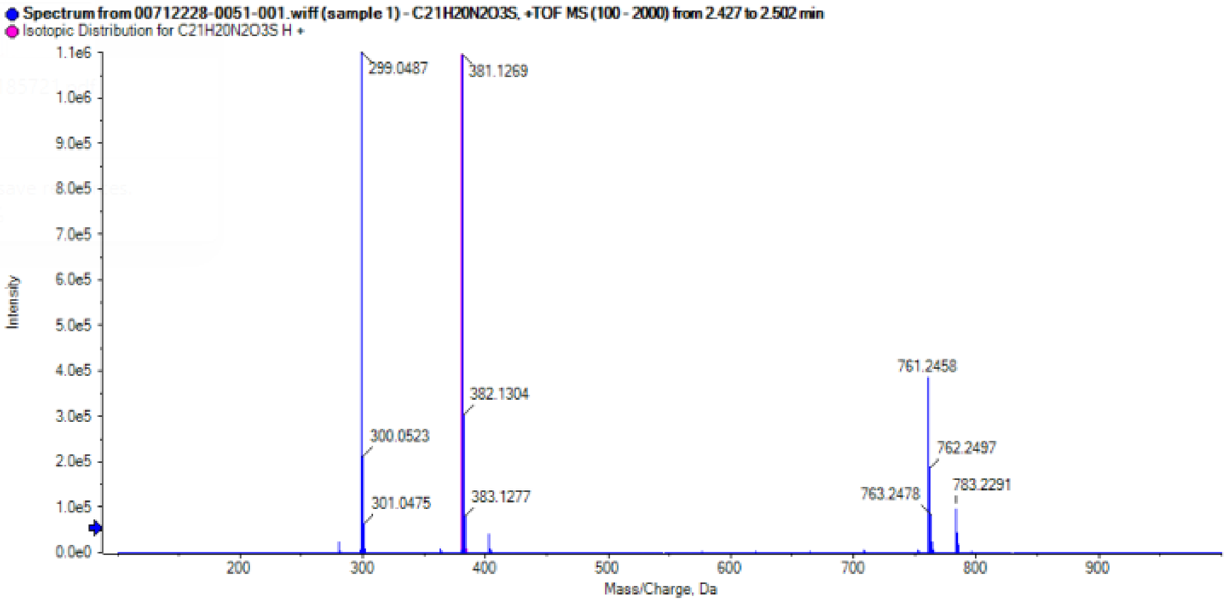

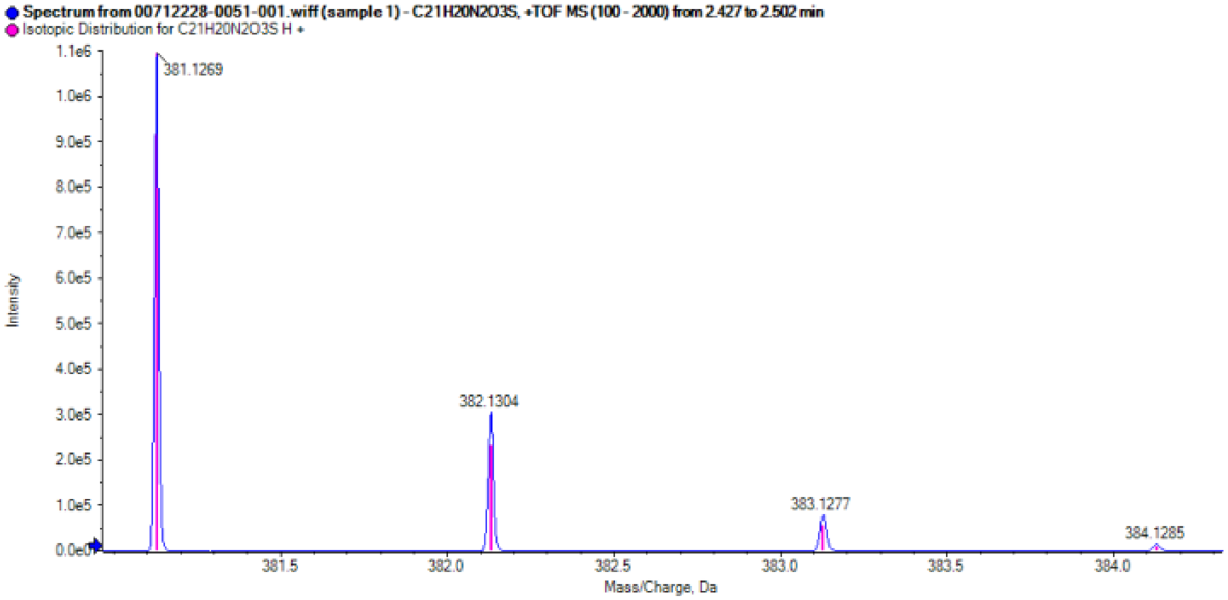

Peak at 2.44 min in UV								
Mass/Charge (Da)	Height	Relative % Height	Compound	Peak Type	Theoretical m/z	Error (ppm)	Theoretical m/z	Theoretical Intensity
381.1269	1097286	100.0	C21H20N2O3S	[M+H]+	381.1267	0.3	381.1267	100.0
382.1304	304353	27.7					382.1298	25.2
383.1277	79446	7.2					383.1271	8.1
384.1285	14392	1.3					384.1282	1.5

Supplementary Figure 61. HPLC of PF-07247685

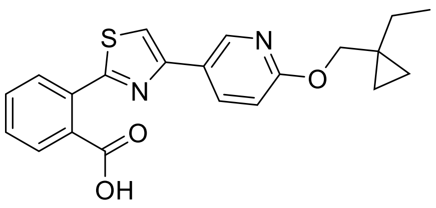

PF-07247685

```
=====================================================================
Acq. Operator   : SYSTEM                        Seq. Line :    2
Sample Operator : SYSTEM
Acq. Instrument : HPLC-233GA                     Location :    7
Injection Date  : 4/27/2023 12:48:32 PM               Inj :    1
                                               Inj Volume : 10.000 µl
Acq. Method     : C:\Users\Public\Documents\ChemStation\1\Data\def_LC_2021 2023-04-27 12-29-
                  18\ACID_GRADIENT.M
Last changed    : 6/9/2021 9:16:24 AM by SYSTEM
Analysis Method : C:\Users\Public\Documents\ChemStation\1\Data\def_LC_2021 2023-04-27 12-29-
                  18\ACID_GRADIENT.M (Sequence Method)
Last changed    : 4/28/2023 2:01:26 PM by SYSTEM
                  (modified after loading)
Method Info     : Column: XBridge C18 5 micron (4.6 mm x 150 mm)
                  Flow rate: 1.500 mL/min with solvents containing 0.1% TFA
                  0-1.5 min: 5% acetonitrile/water
                  1.5-10 min: 5-100% acetonitrile water
                  10-11 min: 100% acetonitrile
                  11-12.5 min: 100-5% acetonitrile/water
```

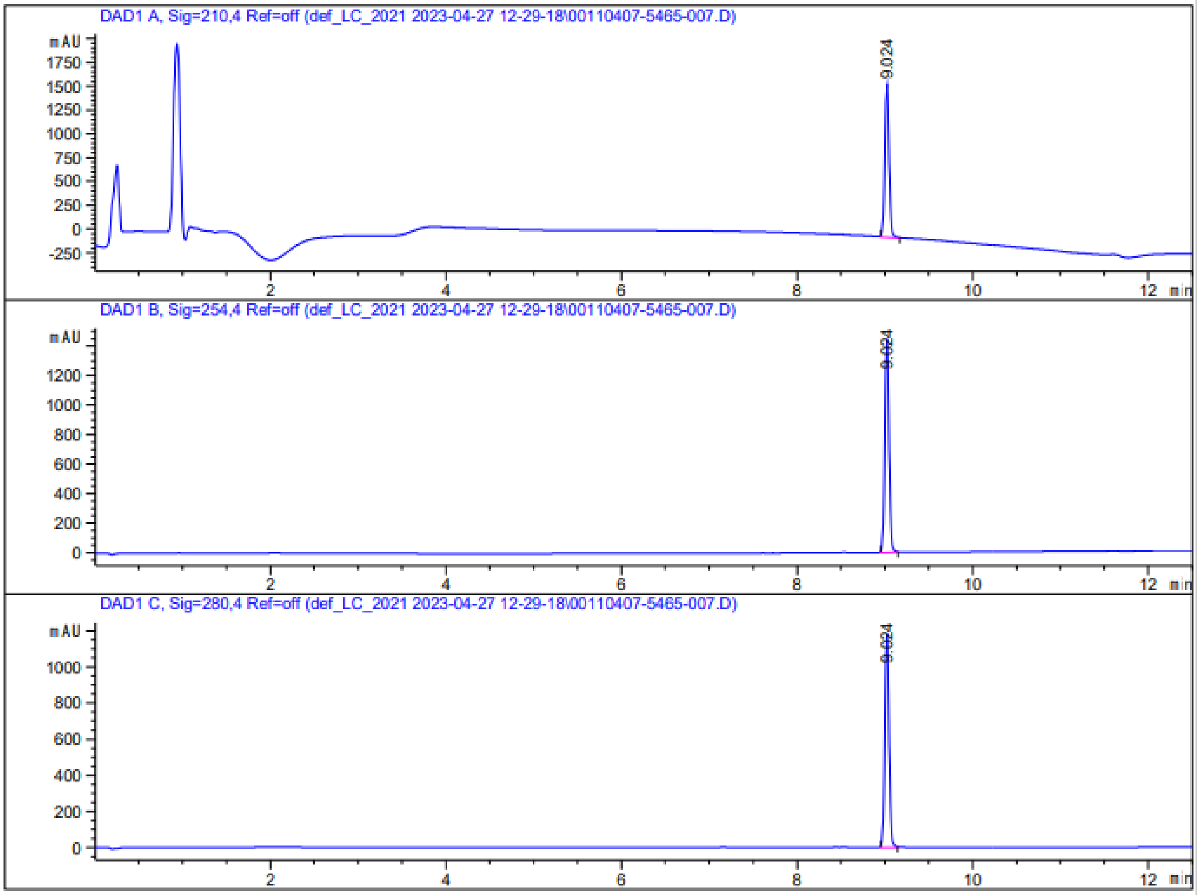

===
Area Percent Report
===

Sorted By : Signal
Multiplier : 1.0000
Dilution : 1.0000
Do not use Multiplier & Dilution Factor with ISTDs

Signal 1: DAD1 A, Sig=210,4 Ref=off

Peak #	RetTime [min]	Type	Width [min]	Area [mAU*s]	Height [mAU]	Area %
1	9.024	BB	0.0549	5616.75732	1601.76013	100.0000

Totals : 5616.75732 1601.76013

Signal 2: DAD1 B, Sig=254,4 Ref=off

Peak #	RetTime [min]	Type	Width [min]	Area [mAU*s]	Height [mAU]	Area %
1	9.024	BB	0.0546	5052.00098	1450.17712	100.0000

Totals : 5052.00098 1450.17712

Signal 3: DAD1 C, Sig=280,4 Ref=off

Peak #	RetTime [min]	Type	Width [min]	Area [mAU*s]	Height [mAU]	Area %
1	9.024	BB	0.0546	4133.79639	1187.12280	100.0000

Totals : 4133.79639 1187.12280

===
*** End of Report ***
