# Supplementary material for: Small molecule branched-chain ketoacid dehydrogenase kinase (BDK) inhibitors with opposing effects on BDK protein levels
Source: Nat Commun. 2023 Aug 9;14:4812. doi: 10.1038/s41467-023-40536-y (PMC10412597; doi:10.1038/s41467-023-40536-y)
Supplement: Supplementary file 3 — Description of Additional Supplementary Files [file 41467_2023_40536_MOESM3_ESM.docx]

**Description of Additional Supplementary Files**

**-Within Supplementary Data PDF:**

Supplementary Figures 1-10

Supplementary Tables 1-4

Supplementary Methods

Supplementary References

Supplementary Figure 11 Western Blot Images

Supplementary Figures 12-61 BDK inhibitor compound spectra images

**-Supplementary Movies:**

File name: Supplementary Movie 1

Description: Visualization of the first principal component (PC1) from essential dynamics analysis of the trajectories for BDK Apo structure

File name: Supplementary Movie 2

Description: Visualization of the first principal component (PC1) from essential dynamics analysis of the trajectories for BDK with BT2

File name: Supplementary Movie 3

Description: Visualization of the first principal component (PC1) from essential dynamics analysis of the trajectories for BDK with PF-07208254

File name: Supplementary Movie 4

Description: Visualization of the first principal component (PC1) from essential dynamics analysis of the trajectories for BDK with PF-07238025

File Name: Supplementary Movie 5

Description: Visualization of the first principal component (PC1) from essential dynamics analysis of the trajectories for BDK with PF-07247685

**-Source Data.zip**

Contains BDK interacting proteins and source data files

**-PDB reports**

D_1000269980_val-report-full_P1

D_1000270041_val-report-full_P1

D_1000270058_val-report-full_P1
